# Transcriptome-wide analyses of early immune responses in lumpfish leukocytes upon stimulation with poly(I:C)

**DOI:** 10.3389/fimmu.2023.1198211

**Published:** 2023-06-14

**Authors:** Shreesha S. Rao, Harald S. Lunde, David W. P. Dolan, Amanda K. Fond, Kjell Petersen, Gyri T. Haugland

**Affiliations:** ^1^ Department of Biological Sciences, Bergen High-Technology Centre, University of Bergen, Bergen, Norway; ^2^ Computational Biology Unit, Department of Informatics, University of Bergen, Bergen, Norway

**Keywords:** poly(I:C), lumpfish, transcriptome, DEG, omics, RIG-I signaling pathway

## Abstract

**Background:**

Both bacterial and viral diseases are a major threat to farmed fish. As the antiviral immune mechanisms in lumpfish (*Cyclopterus lumpus* L.) are poorly understood, lumpfish leukocytes were stimulated with poly(I:C), a synthetic analog of double stranded RNA, which mimic viral infections, and RNA sequencing was performed.

**Methods:**

To address this gap, we stimulated lumpfish leukocytes with poly(I:C) for 6 and 24 hours and did RNA sequencing with three parallels per timepoint. Genome guided mapping was performed to define differentially expressed genes (DEGs).

**Results:**

Immune genes were identified, and transcriptome-wide analyses of early immune responses showed that 376 and 2372 transcripts were significantly differentially expressed 6 and 24 hours post exposure (hpe) to poly(I:C), respectively. The most enriched GO terms when time had been accounted for, were immune system processes (GO:0002376) and immune response (GO:0006955). Analysis of DEGs showed that among the most highly upregulated genes were TLRs and genes belonging to the RIG-I signaling pathway, including LGP2, STING and MX, as well as IRF3 and IL12A. RIG-I was not identified, but *in silico* analyses showed that genes encoding proteins involved in pathogen recognition, cell signaling, and cytokines of the TLR and RIG-I signaling pathway are mostly conserved in lumpfish when compared to mammals and other teleost species.

**Conclusions:**

Our analyses unravel the innate immune pathways playing a major role in antiviral defense in lumpfish. The information gathered can be used in comparative studies and lay the groundwork for future functional analyses of immune and pathogenicity mechanisms. Such knowledge is also necessary for the development of immunoprophylactic measures for lumpfish, which is extensively cultivated for use as cleaner fish in the aquaculture for removal of sea lice from Atlantic salmon (*Salmo salar* L.).

## 1 Introduction

With over 32,000 species, teleost fish exhibit not only a diverse set of phenotypic and genetic traits, there are also huge variations in immune defense mechanisms (1), partly due to a third round of whole-genome duplication within Cypriniformes and Salmoniformes (1–3). Immunological studies make an important basis for the development of preventive measures, and since fish constitute the earliest evolutionary group having both innate and adaptive immunity (4), they are also highly interesting for comparative and evolutionary studies as. The host’s innate immune responses are essential for preventing the spread of pathogens during infections (5). The understanding of lower vertebrate innate immune responses to viral infection has significantly advanced in recent years, particularly regarding the role and diversity of interferons (IFNs) and interferon-induced signaling pathways (6, 7). The IFN system is activated when pattern recognition receptors (PRRs) recognize pathogen-associated molecular patterns (PAMPs) such as viral nucleic acids or glycoproteins, resulting in intracellular signaling and release of type I IFNs and activation of interferon stimulated genes (ISGs) important for antiviral immunity (8, 9). Different classes of PRRs, including Retinoic acid inducible gene (RIG)-I-like receptors (RLRs), Toll-like receptors (TLRs) nucleotide-binding oligomerization domain (NOD)-like receptors (NLR) and cGAS-like receptors (cGLRs), can detect the molecular patterns of various viral particles (10–13).

While the TLRs are membrane-bound receptors (except the soluble version of TLR5 in lower vertebrates), cGLRs, NLRs and RLRs are cytoplasmatic receptors. RLRs, which includes RIG-I, melanoma differentiation-associated protein 5 (MDA5), and laboratory of genetics and pathology 2 (LGP2), can detect RNA virus infection in most cell types by detecting viral RNA (14). RIG-I and MDA5 both recognize viral dsRNA, but the nature of the RNA species recognized are different (15). RIG-I has highest affinity for short dsRNA which is tri-phosphorylated at the 5’end, while MDA5 binds mainly to long dsRNA (16). Interestingly, RIG-I has been identified in fish species belonging to Cypriniformes and Salmoniformes (14, 17), but not in modern teleosts belonging to the Perciformes (18, 19). MDA5 and RIG-I initiate antiviral activity *via* interaction with the signaling adaptor MAVS (mitochondrial antiviral-signaling protein) and the transcription regulators IRF-3 and NFκB, resulting in type I IFN and IFN-stimulated gene (ISG) expression (20, 21). LGP2, which lack CARD domains, is a modulator of RIG-I- and MDA5-mediated antiviral responses, but the function of LGPs in the immune response is controversial (22, 23). Stimulator of IFN genes (STING) is adaptor protein for cGAS but is also involved in RIG-I mediated responses in fish (24). TLR3, 7, 8 and 9 are known to recognize viral RNA and DNA (22), and which signaling *via* IRF3 and IRF7 through the adaptor molecules MyD88 and TRIF (25).

In addition to PRRs, TRIM25 (Tripartite Motif Containing 25), a RING-finger E3 ubiquitin ligase, is essential for RIG-I mediated antiviral activity (10, 26), and there are numerous ISG which are also involved in the process. The use of poly(I:C)to study antiviral immune mechanisms is commonly used, and the studies have shown that genes linked to viral immunity are upregulated when poly(I:C)is exposed with fish, fish leukocytes or fish cell lines (16, 27, 28). According to functional studies and the discovery of fish genes involved in the antiviral response, the IFN antiviral system is diverse among vertebrates (29–31). By producing cytokines, PRRs that recognize PAMPs ensure that the immune response elicited is specific to the invading pathogen, in contrast to e.g. complement factors, which identify potential pathogens, encourage host cells to become more active phagocytically, and eliminate invader microbes. To date, it has discovered that fish type I IFNs have seven subgroups and are further classified into three groups, including Group I (IFNa, IFNd, IFNe and IFNh), Group II (IFNb and IFNc) and Group III (IFNf) (9, 32–35). In several fish species, these receptors have been shown to be upregulated in response to poly(I:C) stimulation (23, 36, 37). In lumpfish, IFNc, IFNd and IFNh have been described (our unpublished data). In the present study, we have performed transcriptome-wide analyses of head kidney leukocytes (HKLs) exposed to poly(I:C).

## 2 Materials and methods

Under Norwegian law, raising fish in normal, ideal conditions is not subject to ethical review (FOR 1996- 01- 15 no. 23)

### 2.1 Fish and rearing conditions

Farmed lumpfish (*C. lumpus* L.) (weight 332.4 ± 63 g, length 17.6 cm ± 0.97 cm) were obtained from a commercial breeder in Sogn & Fjordane County, Norway (Fjord Forsk Sogn AS). During quarantine, the fish were screened for pathogens before being reared in a 500L tank at the Bergen High-Technology Centre’s Aquatic and Industrial Laboratory (ILAB) under normal rearing conditions with a 12h light: 12h dark light regime. The temperature of the outlet water was 8°C, the salinity was 34 PSU, and the minimum oxygen saturation was 77%. The fish were fed with Amber Neptune commercial dry feed (1.5mm) at 2% bodyweight.

The head kidney was isolated from healthy lumpfish and homogenized as described in our previous study (38). Briefly, the leukocytes were separated using discontinuous Percoll gradients a described previously (38, 39). Isolated leukocytes were resuspended in L-15+ medium ((L-15 media without L-Glutamine adjusted to 370 mOsm by adding 5% (v/v) of a solution consisting of 0.41 M NaCl 0.33 M NaHCO3 and 0.66 5 (w/v) D-glucose) supplemented with 100 μg/mL genatamicin (Lonza Biowhittaker Verviers, Belgium), 10 U/mL heparin (Lonza Biowhittaker Verviers, Belgium) and 15 mM HEPES (Sigma-Aldrich, St. louis, USA). Using a CASY TT Cell Counter (Innovatis AG, Reutlingen, Germany), the number of cells, cell viability, and aggregation factor were measured. Leukocytes from each of the 15 fish as distributed into four samples (stimulated samples which were exposed to poly(I:C) for 6 and 24 hours, and non-stimulated controls for the two time points) in 24 well plates (NUNC). Poly(I:C) (100 µg mL^-1^) in a total volume of 0.5 mL were added to the stimulated samples, while for non-exposed cells, L-15+ medium was added instead of poly(I:C). Following incubation at 15°C for 6 and 24 hours, the plates were centrifuged for 10 minutes at 200 x g. The supernatants were removed and lysis buffer containing β-mercaptoethanol was added to each well. The lysates were stored at -80°C before RNA isolation.

### 2.2 RNA isolation

GeneElute Mammalian Total RNA miniprep kit (Sigma) was used to isolate total RNA in accordance with the manufacturer’s instructions. Samples were treated with DNase I (Sigma) to eliminate any lingering traces of genomic DNA. One RNA sample for the RNA sequencing was made by combining total RNA (1 µg) from five fish. Three parallels were set up for RNA sequencing for each time point. After being cleaned with RNA clean & concentrator-5 (Zymo Research) in accordance with the manufacturer’s instructions, the RNA was tested for quality in an Agilent 2100 bioanalyzer. The RIN values varied from 8.5 to 10.(https://www.illumina.com/products/by-type/sequencing-kits/library-prep-kits/truseq-stranded-mrna.html)

### 2.3 RNA sequencing, transcriptome assembly and annotation.

The TruSeq Stranded mRNA sample Preparation kit (Illumina^®^) was used by the Norwegian High Troughput Sequencing Centre to create sequencing libraries using dual indexing on a Perkin Elmer Sciclone NGSx liquid handler system. Final libraries were checked for size and adapter contamination using a standard sensitivity Fragment analyzer NGS kit, and library concentration using qPCR with Kapa Library quantification kit for Illumina (Kapa Biosciences). Paired RNA sequencing was performed on an Illumina HiSeq4000.

### 2.4 Transcriptome-wide bioinformatic analyses.

On average 24383597 (SE 906038) read pairs per sample passed the quality control steps, these reads were then aligned to the *Cyclopterus lumpus* genome, fCycLum1.pri, from Ensemble Release 109 (ensembl.org) using STAR 2.7.10 (40) and the with an average unique alignment rate of 70.2%. Gene counts were obtained using HTSeq 2.0 (41) using the *C. lumpus* gene annotations from Ensembl Release 109. Differentially expressed genes (DEGs) were determined using DESeq2 (42) between exposed and non-exposed samples at both 6 and 24 hours separately as well between all exposed and non-exposed samples using DESeq2’s functions to account for multi-factor model design. RNA sequencing reads and gene counts have been submitted to Array Express under accession number E-MTAB-12884

Gene Ontology overrepresentation was carried out on genes determined to show significant difference between sample groups (adjusted p-value of 0.05 or less) with clusterProfiler (43) and Lumpfish ontology data available from Ensembl. This process was carried out on lists of significantly upregulated genes, significantly downregulated genes and lists combining both up and down regulated genes.

### 2.5 Synteny and phylogenetic analysis

Genomicus v108.01, NCBI and Ensembl were used for genomic synteny analysis. This study compared fish genomes from a common ancestor, where two fish (Lumpfish and Stickleback) genomes from the Actinopterygii (386 Mya) clade, two from chordeta (Zebrafish, Atlantic Salmon) clade and a primate species (human) were used. The phylogenetic analyses were performed as described in our previous study (2), using IQ-TREE with automatic model selection followed by 1000 ultrafast bootstraps (2, 44).

## 3 Results

### 3.1 Differentially expressed genes

**Figure 1:**
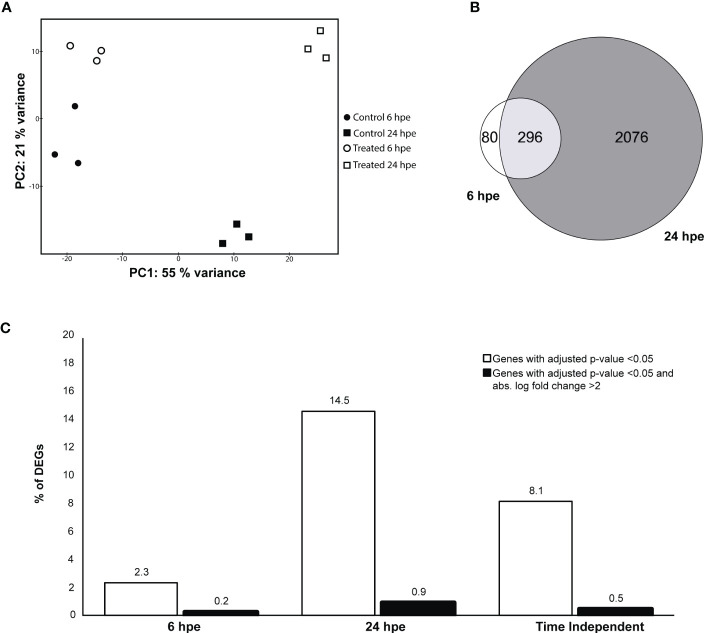
Differential gene expression (DEG) analysis 6 and 24 hours post exposure (hpe) to poly(I:C) **(A)** Principal component analysis. PC1 is time and PC2 is treatment. Black circles are non-treated controls, 6 hpe, white circles are treated samples 6 hpe, white squared are non-treated controls 24h, and black squared are treated sample 24hpe. **(B)** Venn diagram showing the number of DEGs at the different time point. Only those that were statistically significant are shown. White=6 hpe, black=24 hpe and dark grey=genes that were significantly regulated at both time points. **(C)** Percentage of DEGs that were significantly regulated (p-value<0.05) at 6 hpe and 24 hpe are shown in black bars. Percentages of statistically significantly regulated (p-value<0.05) DEG with an absolute log fold change >2. The percentage of DEGs that were significantly regulated (p-value<0.05) in a time independent manner with an absolute log fold change >2 was also plotted.

Transcriptome-wide analyses at two different time points, 6 and 24 hpe to poly(I:C) was performed to gain more information about antiviral immune mechanisms in lumpfish. Principal component analysis (**Figure 1A**) demonstrated a substantial distinction between exposed and non-exposed samples at both time points. The effects of poly(I:C) on gene expression were assessed by performing a differential gene expression (DEG) analysis at the two time points compared to unexposed controls. It was observed that the immune response was stronger and more extensive at 24 hours post-exposure (hpe) as compared to 6 hpe. Additionally, the Venn diagram indicates that 296 genes were detected as significantly differentially expressed at both time points (**Figure 1B**). “Differential expression analysis between test and control samples at 6hpe and 24hpe indicated that 376 (2.3%) 2372 (14.5%) of genes displayed significantly different expression with an adjusted p-value (Benjamini & Hochberg) of less than 0.05 respectively. Of these 310 in the 6hpe test and 1872 in the 24hpe test had a log2 fold change of greater than 1 or less than -1 (ie greater than a doubling or more than a halving in the test group verses the control). The time independent test returned 1324 (8.1%) significantly differently expressed genes of which 520 had a log2 fold change of greater than 1 or less than -1.” (**Figure 1C**). A list of all DEGs is shown in **Suppl. Table 1**.

### 3.2 Global differential gene expression analysis upon polyI:C exposure

**Figure 2:**
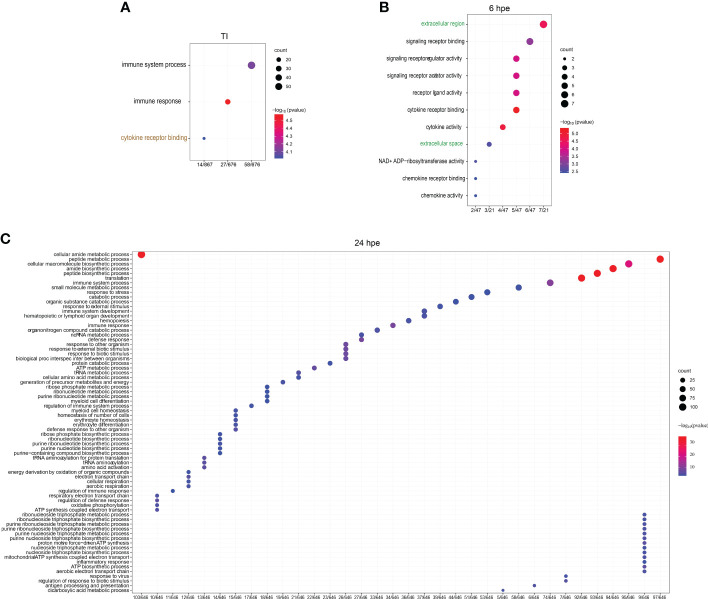
GO overrepresentation analysis of lumpfish leukocytes post poly(I:C) exposure. Semantic plots of significantly regulated genes (log fold change >2 and p-value <0.001) enriched GO terms at **(A)** TI **(B)** 6 hpe and **(C)** 24 hpe. GO terms with black fonts belong to BP, brown font to MF and green fonts to CC. Enrichment p-values are plotted from red and blue, where blue is the smallest p-value and red the biggest p-value. The size of the circles correlates to the semantic size of the GO terms.

To identify significant GO terms in different categories, a GO overrepresentation analysis was performed on the significantly differently expressed genes using the complete gene list from each differential expression test as universes (16198, 16324 and 16316 genes for 6 hpe, 24 hpe and TI). For TI, three GO terms were statistically significant, two within biological function (BP) and one within molecular function (MF): Regulation of immune system processes (GO:0002376) containing 58/676 genes, immune response (GO:0006955) containing 27/676 genes, and cytokine receptor binding (GO:0005126) containing 14/867 genes (**Figure 2A**, **Suppl. Table 2**).These three GO terms included 32 unique sub GO terms which were identified as upregulated, including the terms “lymphocyte activation” and “response to virus” (data not shown). There were no downregulated GO-terms in the TI-set. Furthermore, the GO overrepresentation analysis revealed that at 6 hpe, 11 GO terms, were significantly upregulated (**Figure 2B**). namely extracellular region, and extracellular space, as well as cytokine activity were significantly upregulated. Among these, the cytokine receptor binding had the lowest p-value (0.0004) (**Figure 2B**). At 24 hpe, the overrepresented GO terms within biological processes (BP) included “immune system process”, “immune response”, “response to external biotic stimulus”, “response to other organism”, “response to biotic stimulus”, and “defense response” (**Figure 2C**). GO term belonging to CC and MF is shown in **Suppl. Figure 1**.

**Figure 3:**
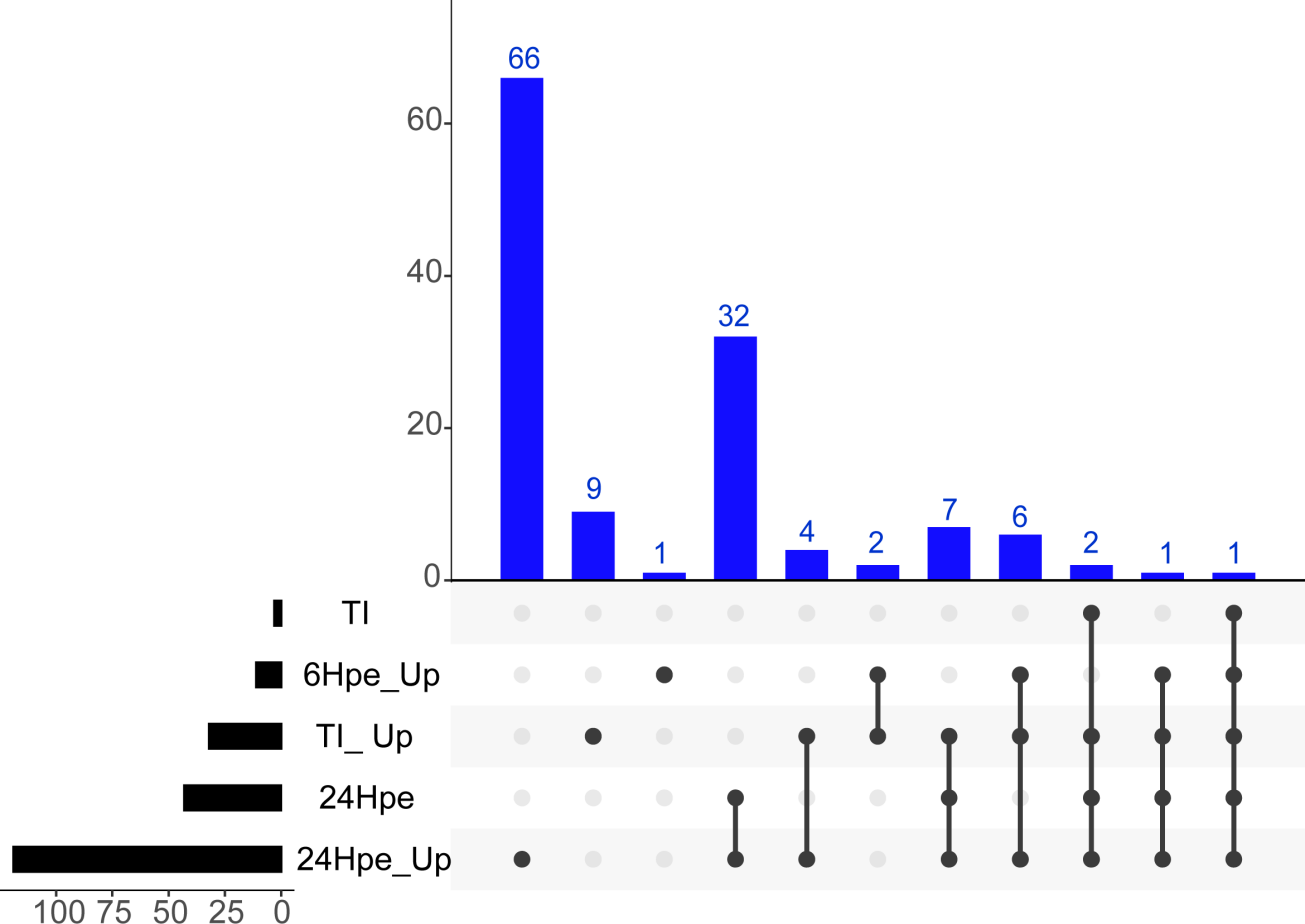
Upset plot of intersections between sets of overrepresented GO-terms of (log fold change >2 and p-value <0.001) lumpfish leukocytes post polyi:c exposure. The bar chart on the left indicates the total number of up-regulated Enriched GO-terms for each analysis group. The blue bar chart indicates the intersection size between each set of Enriched GO-term (s). Dark connected dots on the bottom panel indicate which substrates are considered for each intersection.

**Figure 4:**
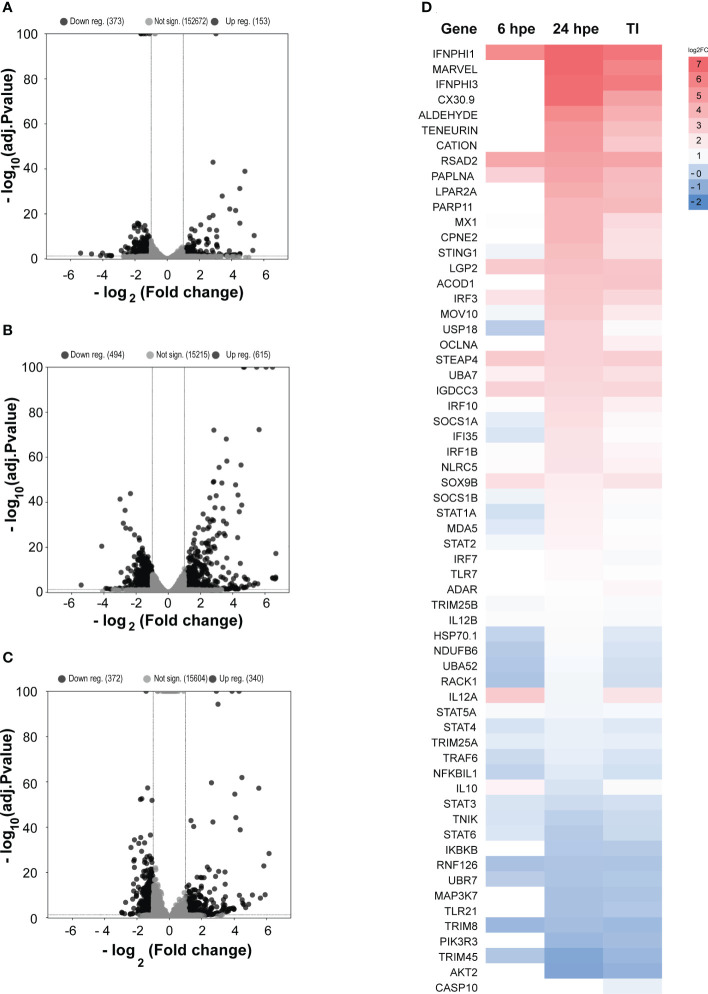
Volcano plot of DEGs, significantly regulated genes at 6 hpe are shown as black dots. Non-significantly regulated genes are shown as grey dots. **(A)** Volcano plot of DEGs 6 hpe. **(B)** Volcano plot of DEGs 24 hpe. **(C)** Volcano plot of DEGs Time independent group. **(D)** Differential gene expression analyses of highly upregulated genes in both time points (6 hours and 24 hours) and TI groups. Only those that are statistically significant regulated (adj. p-value<0.05) are shown. The color gradient represents highly upregulated (Dark Red) to highly downregulated (dark blue) genes. The genes are sorted by fold regulation at 24 hpe followed by TI group. Explanations for the abbreviations are given in **Supplementary Table 4**.

Furthermore, we conducted a comprehensive analysis to identify unique features that distinguish the GO Terms between the two time points and the TI-analysis in different clusters using the UpSetR package (www.bioinformatics.com.cn/srplot) (**Figure 3**). Our analysis revealed that immune system processes were expressed in all five groups, whereas cytokine receptors were observed in four of them. The GO terms included in each of the subcategories (bars) are listed in **Supplemental Table 3**. To get further insight into single genes that were significantly regulated during 6 and 24 hpe to poly(I:C), as well as in the TI group, log2 fold change (log2FC) were plotted against log10 p value. As shown in **Figure 3**, the immune response was stronger and more extensive at 24 hpe compared to 6 hpe (**Figure 4A, B**). In the TI group, 340 versus 372 genes were upregulated and downregulated, respectively (**Figure 4C**). The genes that exhibited the most significant differential expression were associated with immune responses, and a visual representation of the top 62 regulated genes at the 24-hour time point can be seen in **Figure 4D**. Two of the three highest DEGs at 24 hpe and in the TI group, were type I IFNs, IFNphi1 and IFNphi3, which had a log2FC of 6.67 and 6.48, respectively. IFNphi1 was also the most highly upregulated gene at 6 hpe (Log2FC 5.4). Briefly, the differential expression of the RLR family receptor genes, LGP2 and MDA5, was observed in our study. Upregulation of both genes was noted after 24 hours post exposure, with Log2FC of 3.15 (LGP2) and 1.42 (MDA5) compared to 6Hpe, which exhibited Log2 fold change of 2.84 (LGP2) and 0.25 (MDA5). Furthermore, differential expression of TLR family genes was detected, with TLR7a showing upregulation of Log2FC1.12 and TLR21 exhibiting downregulation of Log2 fold change -0.78 at 24 hours post exposure, whereas no expression was observed at 6 hpe. We had previously characterized the TLR signaling pathway in lumpfish (45), but the RLR pathway, which plays a crucial role in detecting viral particles, was not explored. As LGP2 and MDA5 were identified among the DEGs and highly expressed after poly I:C exposure, we further investigated these RIG-I receptors and the RIG-I pathway. Meanwhile, upregulation of NLRC5 was also observed, with a Log2 fold change ranging from 0.97 to 1.81-fold, respectively, from 6 hours post exposure to 24Hpe (**Figure 4D**).

### 3.3 Phylogeny and synteny analyses of RLRs

**Figure 5:**
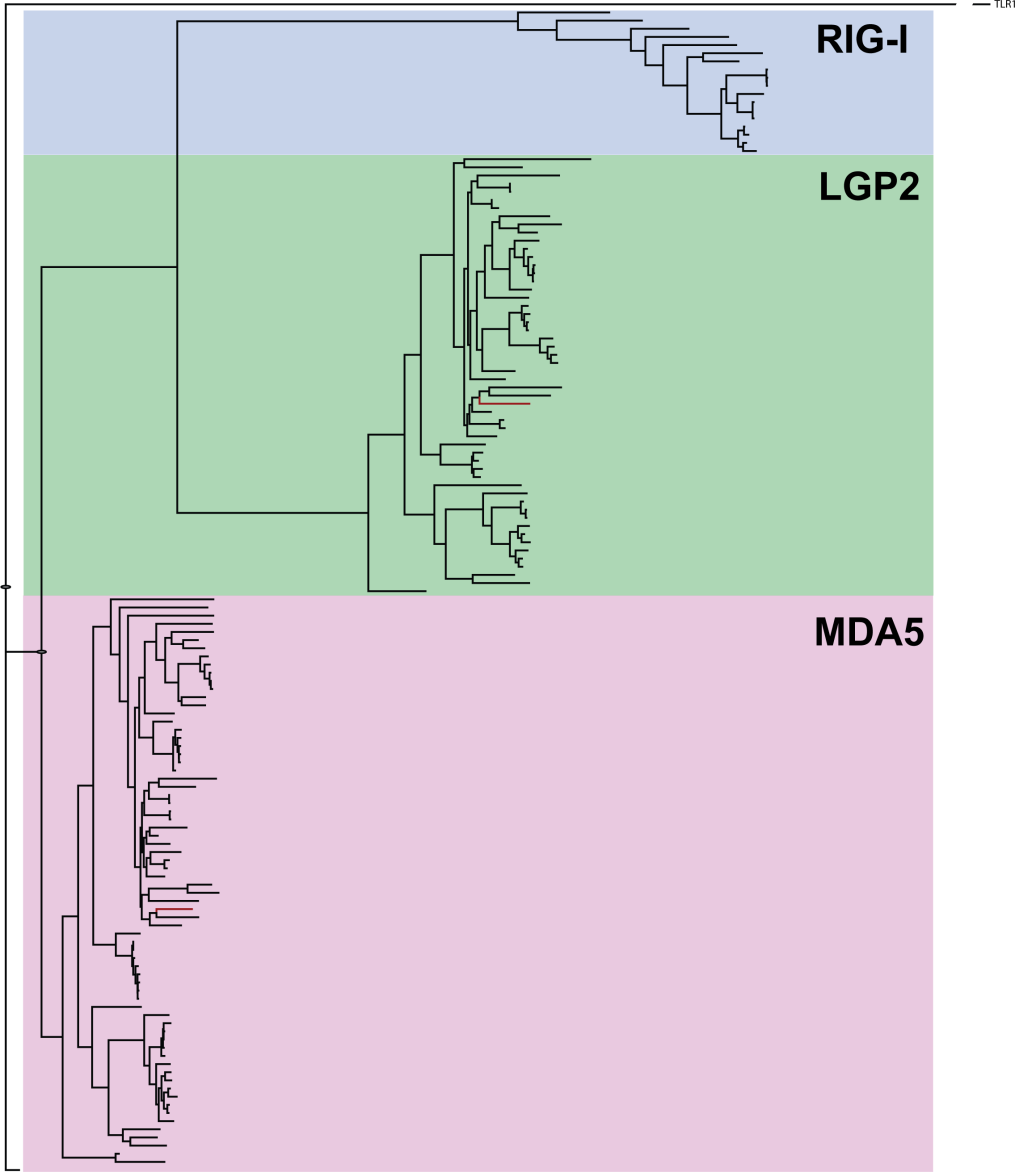
Phylogenetic tree of RIG-1 related genes, using full-length sequences from public databases. The genes are classified into three subgroups, with LGP2 and MDA5 highlighted in red letters as identified in the lumpfish transcriptome. The **Supplemental Figure 1** and **Supplemental Table 5** contains the full species names and accession numbers of the sequences included in the Figure.

A phylogenetic tree was constructed to investigate the relationships among the RLR related gene family (**Figure 5**, **Supplemental Figure 1**, **Supplemental Table 5**). All full-length sequences of RIG-1, LGP2 and MDA5 from mammals and teleost available in NCBI were included in addition to lumpfish sequences. All full-length sequence hits with adequate quality from a BLAST search were used in the study using the lumpfish sequences as query sequences. The RLR-related gene sequences were analyzed phylogenetically, and it was observed that the LGP2 and MDA5 lumpfish candidates were clustered in their respective groups (**Figure 5**). The RIG-1 clade was found to be more like LGP2 gene clade and contained sequences only from salmoniformes and cyprinoformes. RIG-I was not identified in lumpfish. MDA5 was classified as a separate clade with sequences from all fish groups. Lumpfish MDA5 clustered most closely to *Notothenia coriiceos* and *Epinephelus coioides* which both, as *C. lumpus*, belong to Perciformes.

**Figure 6:**
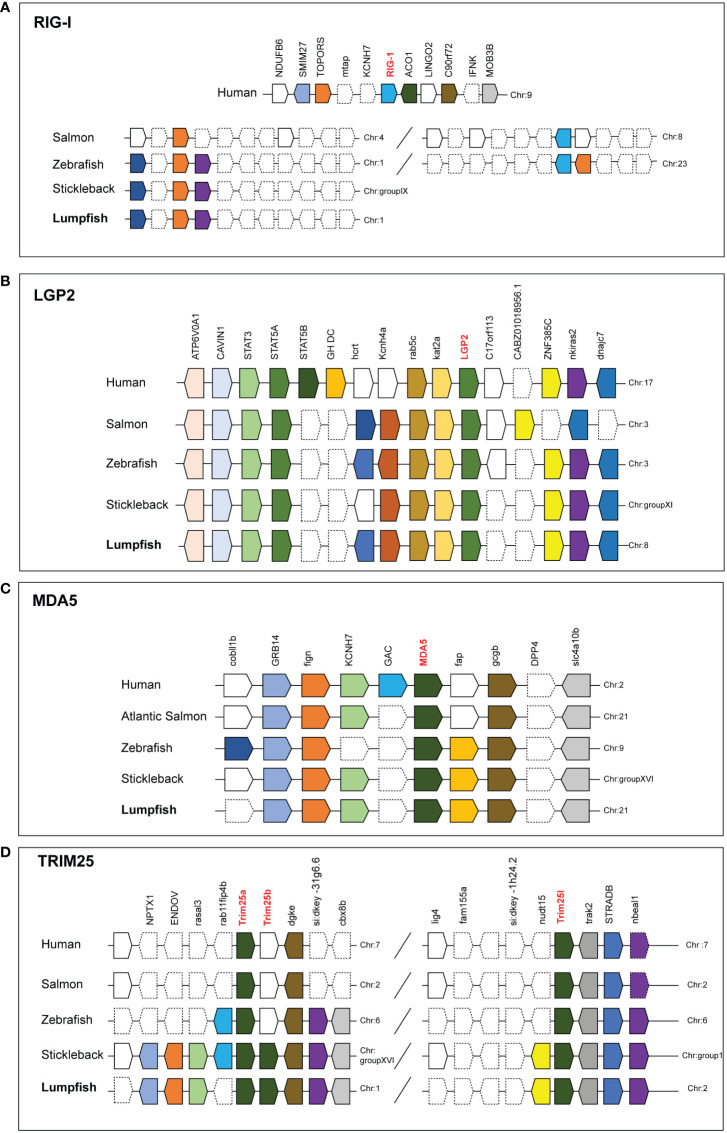
Conserved synteny of human, Atlantic salmon, zebrafish, Stickleback and Lumpfish RIG-1related genes **(A)** RIG-1 **(B)** LGP2 **(C)** MDA5 and **(D)** TRIM25like. Synteny maps comparing RIG-1related genes constructed using the Genomicus Browser (www.genomicus.bio.ens.psl.eu) and BLAST search against genome of organism. Gene symbols are described according to NCBI database. The bar lengths are not proportional to the distances between genes. Dotted lines represent the omitted genes on the chromosome/scaffold. The direction of the arrows indicates the gene orientation.

Synteny analyses of RIG-I showed that it is not conserved among humans and teleosts, shown by Atlantic salmon and zebrafish which are two species where RIG-I has been described. In the two fish species, TOPORS and RIG-I are at different chromosomes, while in human these sequences are closely located. RIG-I was not found in lumpfish or stickleback, which is a closely related species to lumpfish, but the TOPORS gene is conserved. (**Figure 6A**). The synteny of LGP2 and MDA5 are highly conserved in humans and teleost. LGP2 are located next to kat2a and rab5c (**Figure 6B**), while MDA5 are in a cluster with GRB14 and gcgb (**Figure 6C**).

### 3.4 RIG-I signaling pathway in HKLs are activated upon exposure to poly(I:C)

**Figure 7:**
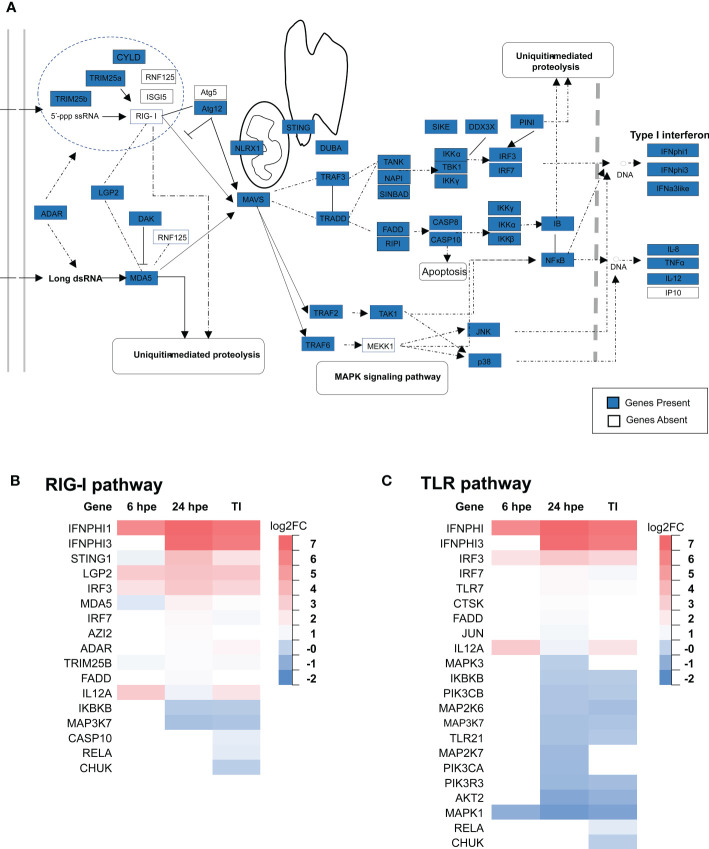
An overview of the RLR and TLR receptor signaling pathway in lumpfish. **(A)** The molecules in the RIG-1 related signaling pathway identified in lumpfish are shown with sky blue boxes, those that are not yet identified are shown in white. The figure is modified from KEGG map04622 Differential gene expression analyses of members of the RLR **(B)** and TLR **(C)** pathways at 6 hours, 24 hours post exposure (hpe) and TI group are showed above. Only those that are statistically significant regulated (p-value<0.05) are shown.

Transcriptome and genome mining showed that lumpfish had most of the components of the RIG-I pathway (**Table 1**). Except for the missing RIG-I (**Figure 5**, **6A**), the only genes not found are Atg5, RNF125, MEKK1 and IP10 (**Figure 7A**). Candidate sequences were found for ISG15, based on synteny and sequence similarity to ISG15 from other fish species. Furthermore, DEG analyses of the RIG-I pathway were performed, showing that the most highly upregulated genes were LGP2, STING1 and IRF3 (**Figure 7B**). IKKβ and TAK1 were the most highly downregulated genes.

**Table 1:** Verified genes belonging to the RIG-I pathway in lumpfish (KEGG map04622).

ENSAMBL	KEGG ID	ABBREVIATION	Name
ENSCLMG00005019134	K00863	DAK/TKFC	Triose/Dihydroxyacetone Kinase / Fad-Amp Lyase (Cyclizing)
ENSCLMG00005001158	K02372	FADD	Fas-Associated Death Domain Protein
ENSCLMG00005017167	K02580	NF-kBp105	Nuclear Factor NF-Kappa-B P105 Subunit
ENSCLMG00005001554	K02861	RIPK1	Receptor-Interacting Serine/Threonine-Protein Kinase 1
ENSCLMG00005022360	K03171	TRADD	Tumor Necrosis Factor Receptor Type 1-Associated Death Domain Protein
ENSCLMG00005004208	K03173	TRAF2	Tnf Receptor-Associated Factor -2
ENSCLMG00005015744	K03174	TRAF3	Tnf Receptor-Associated Factor -3
ENSCLMG00005000677	K03175	TRAF6	Tnf Receptor-Associated Factor -6
ENSCLMG00005014902	K04398	CASP8	Caspase 8
ENSCLMG00005020302	K04400	CASP10	Caspase 10
ENSCLMG00005017854	K04427	TAK1	Mitogen-Activated Protein Kinase Kinase Kinase 7
–	K04441	P38	P38 Map Kinase
ENSCLMG00005001245	K04441	nlk1	Nemo-Like Kinase, Type -1
ENSCLMG00005001823	K04441	mapk4	Mitogen-Activated Protein Kinase -4
ENSCLMG00005002098	K04441	mapk8a	Mitogen-Activated Protein Kinase -8
ENSCLMG00005002361	K04441	mapk7	Mitogen-Activated Protein Kinase -7
ENSCLMG00005002363	K04441	mapk10	Mitogen-Activated Protein Kinase -10
ENSCLMG00005003286	K04441	mapk6	Mitogen-Activated Protein Kinase -6
ENSCLMG00005003545	K04441	nlk2	Nemo-Like Kinase, Type -2
ENSCLMG00005004068	K04441	mapk15	Mitogen-Activated Protein Kinase -15
ENSCLMG00005005454	K04441	mapk12a	Mitogen-Activated Protein Kinase -12a
ENSCLMG00005005860	K04441	mapk3	Mitogen-Activated Protein Kinase -3
ENSCLMG00005006513	K04441	mapk14b	Mitogen-Activated Protein Kinase -14b
ENSCLMG00005007582	K04441	zgc:171775	Zgc:171775
ENSCLMG00005009478	K04441	mapk9	Mitogen-Activated Protein Kinase- 9
ENSCLMG00005010485	K04441	mapk13	Mitogen-Activated Protein Kinase -13
ENSCLMG00005010686	K04441	gipc1	GIPC PDZ Domain Containing Family, Member 1
ENSCLMG00005011533	K04441	MAPK14	Mitogen-Activated Protein Kinase -14a
ENSCLMG00005015694	K04441	mapk1	Mitogen-Activated Protein Kinase -1
ENSCLMG00005020114	K04441	mapk11	Mitogen-Activated Protein Kinase -11
ENSCLMG00005021393	K04441	mapk12b	Mitogen-Activated Protein Kinase -12b
ENSCLMG00005022014	K04441	mapk8b	Mitogen-Activated Protein Kinase -8b
ENSCLMG00005002340	K04467	IKKa	Inhibitor Of Nuclear Factor Kappa-B Kinase Subunit Alpha
ENSCLMG00005013267	K04734	IkBa	NF-Kappa-B Inhibitor Alpha
ENSCLMG00005003299	K04735	NF-kBp65	Transcription Factor P65
ENSCLMG00005018688	K05410	TBK1	Tank-Binding Kinase 1
ENSCLMG00005002258	K05411	IRF3	Interferon Regulatory Factor 3
ENSCLMG00005004829	K05425	IL-12b	Interleukin 12b
ENSCLMG00005012066	K07209	IKKb	Inhibitor Of Nuclear Factor Kappa-B Kinase Subunit Beta
ENSCLMG00005021114	K07210	IKKg	Inhibitor Of Nuclear Factor Kappa-B Kinase Subunit Gamma
ENSCLMG00005021145	K08336	ATG12	Ubiquitin-Like Protein Atg12
ENSCLMG00005018569	K08601	CYLD	Ubiquitin Carboxyl-Terminal Hydrolase Cyld
ENSCLMG00005000688	K09578	PIN1	Peptidyl-Prolyl Cis-Trans Isomerase Nima-Interacting 1
ENSCLMG00005000452	K10030	IL-8	Interleukin 8
ENSCLMG00005004085	K10652	TRIM25a	Tripartite Motif-Containing Protein 25
ENSCLMG00005004082	K10652	TRIM25b	E3 ubiquitin ligase Tripartite Motif-Containing Protein 25/ Riplet
ENSCLMG00005003340	K10652	TRIM25-like	Tripartite Motif-Containing Protein 25 like
ENSCLMG00005003514	K11594	DDX3X	Atp-Dependent Rna Helicase Ddx3x
LOC117736832/33/35/60	K12159	ISG15	Ubiquitin Cross-Reactive Protein
NA	K12170	RNF125	E3 Ubiquitin-Protein Ligase Rnf125
ENSCLMG00005003426	K12647	MDA5	Interferon-Induced Helicase C Domain-Containing Protein 1
ENSCLMG00005015335	K12649	LGP2	Atp-Dependent Rna Helicase Dhx58
ENSCLMG00005003770	K12650	TANK	Traf Family Member-Associated Nf-Kappa-B Activator
ENSCLMG00005003531	K12653	NLRX1	Nlr Family Member X1
ENSCLMG00005019234	K12656	SIKE	Suppressor Of Ikk-Epsilon
ENSCLMG00005014897	K12648	IPS-1/MAVS	Mitochondrial Antiviral-Signaling Protein
ENSCLMG00005006159	K12651	NAP1	5-Azacytidine-Induced Protein 2
NA	K12652	SINTBAD/ TBKBP1	Tank-Binding Kinase 1-Binding Protein 1
ENSCLMG00005019424	K12654	MITA/STING	Stimulator Of Interferon Response Cgamp Interactor 1
ENSCLMG00005000565	K12655	DUBA	Otu Domain-Containing Protein 5
ENSCLMG00005009384	K12656	IKKe	Suppressor Of Ikk-Epsilon
ENSCLMG00005001707	K12968	ADAR	Double-Stranded Rna-Specific Adenosine Deaminase

Several TRIM genes were identified lumpfish, such as TRIM8, TRIM45, TRIM62, TRIM16, TRIM36, TRIM26, and TRIM25 paralogs (**Figure 4D**). Through synteny analysis of the TRIM25 gene, it was discovered that two copies of TRIM25 (TRIM25a and TRIM25-like) are conserved in humans, Atlantic salmon, and zebrafish (**Figure 6D**). Interestingly, lumpfish and stickleback have an extra copy of TRIM25a (termed TRIM25b). The TRIM25 paralogs in humans, Atlantic salmon, and zebrafish are conserved and located on the same chromosome as DGKE, STRADB, TRIM25a, and TRIM25 Like genes. In contrast, stickleback, and lumpfish, which have three copies of TRIM25 genes, were found on two different chromosomes. In stickleback, TRIM25a, TRIM25b, and DGK are conserved in chromosome group XVI, while TRIM25like and STRADB are conserved in chromosome group I. In lumpfish, TRIM25a and TRIM25Like are conserved in chromosome 1, while TRIM25b and STRADB are conserved in chromosome 2 (**Figure 6D**).

Amongst all TRIM genes in lumpfish, TRIM25b and TRIM25a both demonstrated an increase in differential expression from 6Hpe to 24Hpe. TRIM25b showed a Log2 fold change of 0.64 at 6Hpe, which increased to 0.82 at 24Hpe, while TRIM25a exhibited a Log2 fold change of 0.31 at 6Hpe, which increased to 0.43 at 24Hpe. In contrast, TRIM8 and TRIM45 were both downregulated, with TRIM8 showing an increase in downregulation of differential expression from 6Hpe to 24Hpe. Specifically, TRIM8 demonstrated a Log2 fold change of -1.07 at 6Hpe, which increased to -0.86 at 24Hpe. TRIM45, on the other hand, demonstrated a decrease in differential expression from 6Hpe to 24Hpe, with a Log2 fold change of -0.61 at 6Hpe, which decreased to -1.52 at 24Hpe (**Figure 4D**).

### 3.5 TLRs and TLR signaling

**Figure 8:**
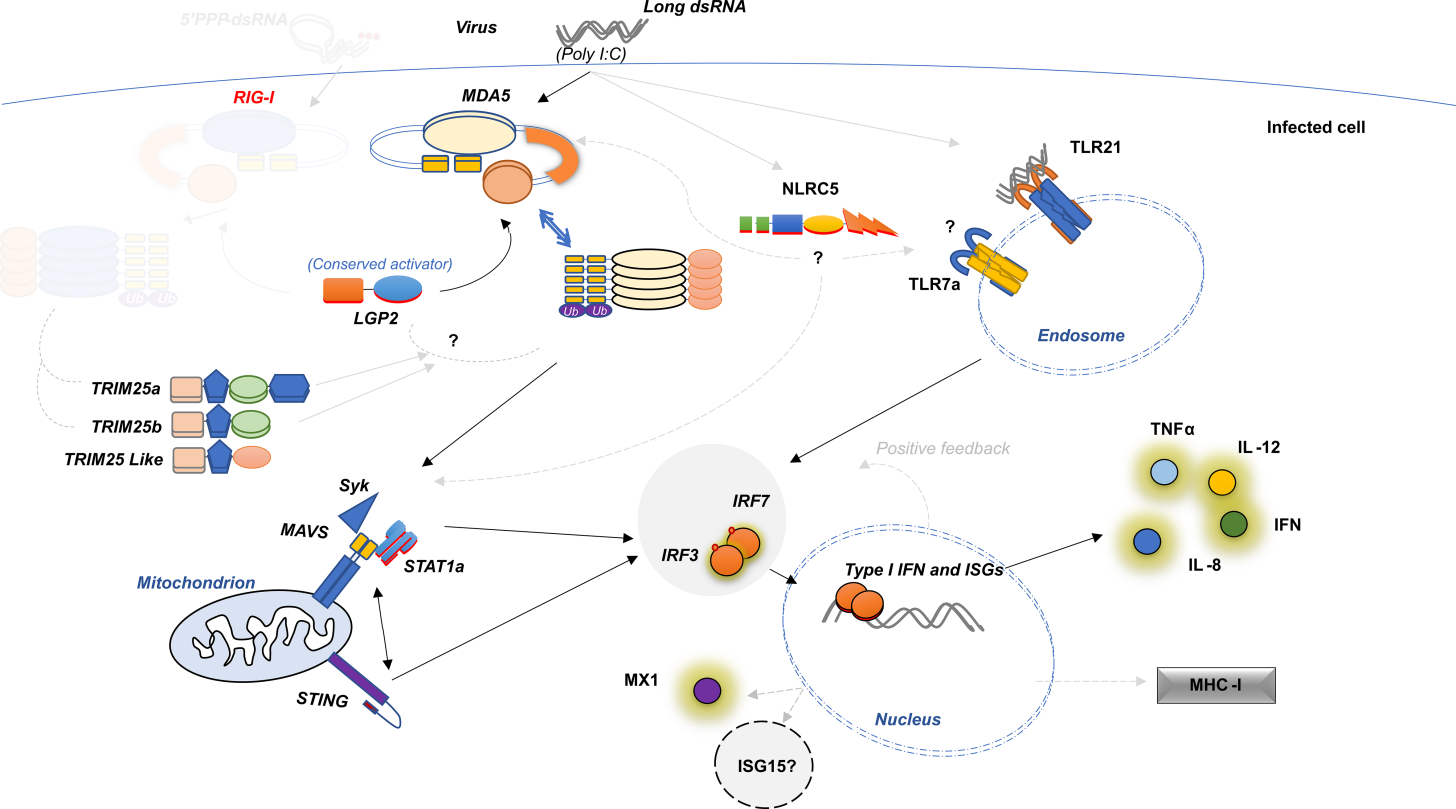
Schematic Diagram Showing Structure-Based Model for anti-viral mechanisms in lumpfish. Dark line represents the hypothesis explained in the present study, grey dotted lines represent possible influence which are explained elsewhere.

In previous study, several TLR genes in lumpfish have been identified (45), which is consistent with previous research on perciforms (46–48). Upon exposure to poly(I:C), TLR7a was upregulated, with a differential expression increase of 1.12 at 24 hours post-exposure, while TLR7b was not expressed (**Figure 7C**). Both TLR21 and TLR22 were identified in lumpfish, with TLR21 significantly downregulated at 24 hours post-exposure (with a -0.78 Log2Fold change), but not at 6 hours post-exposure, and TLR22 was not differentially expressed throughout the experiment (**Figure 7C**). IRF3 and IRF7 are crucial transcription factors that are involved in the innate immune response pathways and are produced by both TLR and RLR signaling. In the current study, it was observed that IRF3 showed the highest level of upregulation in a time-independent manner (**Figure 8**), with a 2.46 log2fold change, indicating its importance in the immune response. Specifically, IRF3 showed an increase in upregulation from 1.94 log2fold change at 6 hours post-exposure to 2.97 log2fold change. In contrast, IRF7 was not differentially expressed at the initial time point of poly(I:C) exposure, but later showed upregulation to a 1.13 log2fold change.

## 4 Discussions

In recent years, viral infections have caused diseases in lumpfish (49, 50). There are, however, not currently available cell line(s) for propagation of those viruses. Thus, to a synthetic PAMP, PolyI:C, was used to mimic viral infection and explore antiviral immune mechanisms in lumpfish. A large portion of the published data on fish immune response to viral infection come from studies on fish model organisms, such as Zebrafish and Japanese Medaka, or species that are important for the aquaculture industry such as salmonid fish and tilapia (51). Recent advancement in high throughput sequencing, both RNA sequencing and whole genome sequencing of numerous species, including lumpfish (45, 52), have given us valuable tools for species specific, as well as comparative and evolutionary, studies. In several species, polyI:C consistently induces a rapid and strong interferon response (17, 53). In the case of epithelioma papulosum cyprini (EPC) cells or conditioned medium from polyI:C-treated cells, there was a significant increase in Mx protein levels following polyI:C treatments (54). The ability of poly(I:C)to effectively induce the expression of the genes gig and vig/viperin *in vitro* suggests that it is a good virus mimic (6). For *in vivo* experiments, poly(I:C) is usually administered to fish *via* intraperitoneal (i.p.) injection. In a zebrafish model, the activation of ifn and mx genes in the head kidney was analyzed after i.p. injection of polyI:C, and the peak expression was observed 48 hours later (6).

By annotating various sequence variations, one can reveal the genome-wide evolution of orthologous protein-coding genes and functional gene duplicates that are retained. Genome annotation techniques include analysis, comparison, estimation, and precision which are employed to extract structural and functional information from raw data (55). To improve the annotation of the lumpfish genome, it is crucial to perform a comprehensive global analysis of DEGs and compare coding regions among well studied species. To identify DEGs through RNAseq, a lower threshold level was used to reduce the number of gene hits, with a significant threshold of twofold difference set at the lower end of the threshold spectrum. Furthermore, a time-independent analysis was performed to assess the impact of polyI:C on lumpfish leukocytes for a comprehensive understanding of its overall influence. The study used about 23,000(aprox.) predicted genes in the current lumpfish genome/transcriptome (biomart/ https://www.ensembl.org). The predicted coding genes were searched against existing databases. As expected, most of the differentially expressed transcripts were linked to the RLR and TLR pathways and hence these pathways were further analyzed.

Through annotation in lumpfish, multiple TLRs were identified, including TLR1, TLR2, TLR3, TLR5 (membrane-bound and soluble), TLR7, TLR8, TLR9, TLR13, TLR14, TLR18, TLR21, TLR22, and TLR28. Notably, two TLR7 paralogs, TLR7 a/b, were also discovered, which aligns with our previous studies on TLR in lumpfish (45), and other perciforms (46–48). TLR3, TLR7/8, and TLR9 are intracellular viral nucleic-acid-sensing receptors localized in the endosomes, while most other TLRs are found on the cell membrane (56–58). While TLR7 are highly expressed in immune cells like dendritic cells and B cells and macrophages, TLR3 are expressed primarily in fibroblasts and epithelial cells, and thus their overexpression’s are presumably not observed in *in-vitro* experiments (59–61). We discovered that exposing lumpfish leukocytes to PolyI:C increased TLR expression and enhance the innate immune response, with a significant upregulation of TLR7a and downregulation of TLR21, but no significant changes in TLR7b or other TLR genes were observed.

A classical TLR7 contains a leucine-rich repeat (LRR) ectodomain that recognizes ligands and a cytoplasmic Toll/interleukin-1 receptor (TIR) domain that carries out downstream signal transduction. However, TLR7b in lumpfish does not possess either of these domains, leading to the possibility that it may be a pseudogene, like those found in trout’s and Atlantic salmon (62, 63). TLR7 can activate a variety of signaling cascades, ultimately leading to the production of pro-inflammatory cytokines and IFNs, making them a potential candidate for use as a vaccine adjuvant (64). Nonetheless, the precise functions of these paralogs on an individual basis remain ambiguous. TLR21 belongs to the TLR11 superfamily and is found primarily in non-mammalian species. In fish, it is divided into two clusters: TLR11/TLR13/TLR21 and TLR22/TLR23 (45, 65, 66). TLR21 is thought to function in both avian and fish species in a manner like mammalian TLR9, recognizing microbial DNA as a peril signal and activating downstream innate and adaptive immune responses (67, 68). Both TLR21 and TLR22 from this super-family were observed in lumpfish in our study, and TLR21 was significantly regulated 24 hours after polyIC exposure.

The RLR family is composed of three cytoplasmic receptors: RIG-I, MDA5, and LGP2 (69). RIG-I and MDA5 identify viral RNA species and initiate interferon signaling (70). LGP2 lacks a signaling domain and it may function as a positive or negative regulator of the other two (16, 71, 72). The RIG-I-like helicase family to have evolved from a common ancestor comprised of genes encoding various core functional domains (14). Diversification of core functional domains may be important in terms of functional divergence in viral PAMP recognition (16). When viral nucleic acids (or glycoproteins) are detected by pathogen-associated molecular patterns (PAMPs), it results in intracellular signaling and the release of IFN and activation of interferon-stimulated genes (ISGs), which play a vital role in antiviral immunity (9). The identification of lumpfish LGP2 and MDA5 as intracellular RNA sensors that initiate the signal through MAVS as a mechanism of early detection and activation of interferon-stimulated genes (ISGs) (**Figure 8**) aligns with earlier findings in Olive flounder (73). Early detection and response play a vital role in combating viral infections, and RIG-I is regulated by the ubiquitination of three ligases, such as TRIM25 and/or RING finger protein (Riplet) (74, 75),, we have chosen them for synteny analysis in the present study along with RLR genes.

The N-terminal regions of proteins in the TRIM family contain a RING domain, one or more B-box domains, and a coiled-coil domain (TRIM/RBCC) (75). TRIM25a positively regulates MDA5, MAVS, and TRAF3, leading to the activation of nf-kB, and interacts with Zinc finger protein (76). Studies have shown that TRIM25a actively participates in the regulation of antiviral immune responses during viral infections, as evidenced by its transcription in response to interferon stimulation (75). Unlike in human, zebrafish or in Atlantic salmon, the lumpfish genome has an extra TRIM25 paralog called TRIM25b (E3ligase/ISG15) which may act as an E3 ligase by conjugating ubiquitin and ISG15 (6), but the species specific activity of the protein is yet to be confirmed. Interestingly, ISG15 in humans stabilizes USP18, a negative regulator of the type I IFN receptor (77). However, in lumpfish, only TRIM25a has complete domains to be fully functional like its mammalian counterpart, making it the likely functional orthologue (**Figure 8**).

These findings suggest that viruses may develop the capacity to inhibit ubiquitin ligases to evade innate immune responses. However, it is also interesting to see that in mammals it is evident that Riplet (mediates C-terminal domain) and TRIM4 (mediates 2CARDs) are among some of the ubiquitin ligases involved in K3-linked polyubiquitination and activation of RIG-I (78–80). Though TRIM4 and RIPLET share an N-terminal RING domain and a C-terminal SPRY domain to TRIM25, RIPLET does not belong to the TRIM family because it lacks B-box domains. However, TRIM4 belongs to the same subfamily as TRIM25, which can also target RIG-I polyubiquitination. On the other hand, TRIM4 and TRIM25 ubiquitin ligases target the 2CARDs similarly, they may be capable of compensating for one another’s inadequacies in terms of replacement (78). It was recently confirmed in mammals (mouse and human cell lines) that TRIM25 does not affect the RIG-I dependent IFN response, wherein it is the Riplet that play a major role in RIG-I dependent IFN activation (75, 81, 82). Taking this into account, the current study confirms that in the absence of the RIG-I gene, Riplet activity was constrained and TRIM25 was highly regulated, with potential downstream effects yet to be investigated. In a study conducted to understand the cross talk between type I IFNs induced IL-27 it was observed that TRIM25 induction requires the influence from both transcription factors STAT1 and STAT3 supported by interleukin-27 (IL-27) activation (83). Together, these findings highlight the critical roles played by the three ubiquitin ligases TRIM25, TRIM4, and Riplet in viral particle detections as well as the potential for these genes to interact with other pathways, such as the JAK/STAT pathway. This may offer a novel theory for the mechanisms underlying viral detection in lumpfish.

From an evolutionary perspective, it is plausible that a fish species possessing a greater number of copies of the selectively advantageous haplotype may exhibit a diminished cytokine response to infection, while concurrently displaying an enhanced ability to restrict viral growth (84). This highlights the significance of examining gene evolutionary synteny as a crucial basis for investigating the characterization of immune-related genes in fish, which may yield insights for further functional studies. It has been observed before that genes lacking homology are typically shorter than those with conserved homologues that have undergone significant modifications (16). This connection has been interpreted as evidence that young genes can develop independently from short open reading frames (69). However, this is due to a bias brought on by short genes’ faster rates of evolution. If this is the case, it might help to explain why some short genes that seem to be young evolve more quickly. The case is observed in the present study with the absence of RIG-1 gene in lumpfish, meanwhile multiple paralogs of TRIM25 and TLR7 are observed (**Figures 6A, D**). This is also a case in birds where, ducks limit viral replication by early cytokine expression, while chickens lack RIG-I and some signaling pathway modulators, which delays interferon response and increases viral replication (26, 85, 86).

Homologous genes have similar evolutionary histories, indicating that they evolved from a common ancestor (87). Various sources, such as gene duplication, exon shuffling, and gene fusion, contribute to the creation of new genes (88, 89). Conserved genes are crucial in the basic biology of organisms, while non-conserved genes are responsible for their unique traits (26). Understanding the importance of non-conserved genes can improve our understanding of evolution by exploring previously unexplored areas. Homologous gene groups can be studied to understand their evolutionary history and intraspecies divergence. The conserved synteny of lumpfish allows us to evaluate the contribution of complete divergence to their gene pool.

## 5 Conclusion

The lumpfish is an important species in aquaculture as it helps control sea lice in salmon farming. However, viral and bacterial infections have threatened lumpfish production. To better understand the immune system of this important species, the present study utilized poly(I:C), a synthetic dsRNA, as a virus mimic. Our data demonstrated rapid and strong responses of components in the RLR and TLR signaling pathway. The RLR family of cytoplasmic receptors, including LGP2, MDA5 and paralogs of TRIM25 genes initiate MAVS and STING stimulations in release of IRF3 and IRF7 leading to interferon signaling. The absence of the RIG-1 gene in lumpfish raises the possibility of a correlation between the RLR and TLR pathways, shedding light on the unique immune response of this species. These findings can be instrumental in developing effective therapies and strategies to combat viral diseases in lumpfish. Further understanding of the lumpfish immune system and their response to virus/viral particle exposure at the individual gene level is crucial. Thus, the identification of immune genes, transcriptome-wide mapping of signaling pathways, and early immune responses described in this study provide a valuable foundation for developing more efficient immune prophylactic measures and evaluating the efficacy of different prophylactic strategies.

## Data availability statement

The datasets presented in this study can be found in online repositories. The names of the repository/repositories and accession number(s) can be found below: E-MTAB-12884 (Array Express).

## Ethics statement

Ethical review and approval was not required for the animal study because The study was performed with leukocytes isolated from healthy lumpfish.

## Author contributions

GH performed *in vitro* stimulation and RNA isolation. SR, HL, AF and GH performed analysis. DD and KP performed pre-processing and data analysis of transcriptome data. SR and GH wrote the initial draft. All authors contributed to the article and approved the submitted version.
