# Corrigendum: Transcriptome-wide analyses of early immune responses in lumpfish leukocytes upon stimulation with poly(I:C)

**DOI:** 10.3389/fimmu.2024.1452608

**Published:** 2024-06-28

**Authors:** Shreesha S. Rao, Harald S. Lunde, David W. P. Dolan, Amanda K. Fond, Kjell Petersen, Gyri T. Haugland

**Affiliations:** ^1^ Department of Biological Sciences, Bergen High-Technology Centre, University of Bergen, Bergen, Norway; ^2^ Computational Biology Unit, Department of Informatics, University of Bergen, Bergen, Norway

**Keywords:** poly(I:C), lumpfish, transcriptome, DEG, omics, RIG-I signaling pathway

In the published article, there was an error in the **Acknowledgment** section. In the original publication, we would like to elaborate on the funding received, as some information was missed. The correct statement appears below.

**ACKNOWLEDGMENTS**

Authors thank SEAS (Shaping European Research Leaders for Marine Sustainability) programme and University of Bergen for financial and administrative support for the project. The sequencing service was provided by the Norwegian Sequencing Centre (www.sequencing.uio.no) a national technology platform hosted by the University of Oslo and supported by the “Functional Genomics” and “Infrastructure” programs of the Research Council of Norway and the Southeastern Regional Health Authorities. The SEAS project has received funding from the European Union’s Horizon 2020 research and innovation programme under the Marie Skłodowska-Curie grant agreement No 101034309.

The authors apologize for this error and state that this does not change the scientific conclusions of the article in any way. The original article has been updated.

