# Supplementary figures and images for: Transcriptome-wide analyses of early immune responses in lumpfish leukocytes upon stimulation with poly(I:C)
Source: Front Immunol. 2023 Jun 14;14:1198211. doi: 10.3389/fimmu.2023.1198211 (PMC10300353; doi:10.3389/fimmu.2023.1198211)

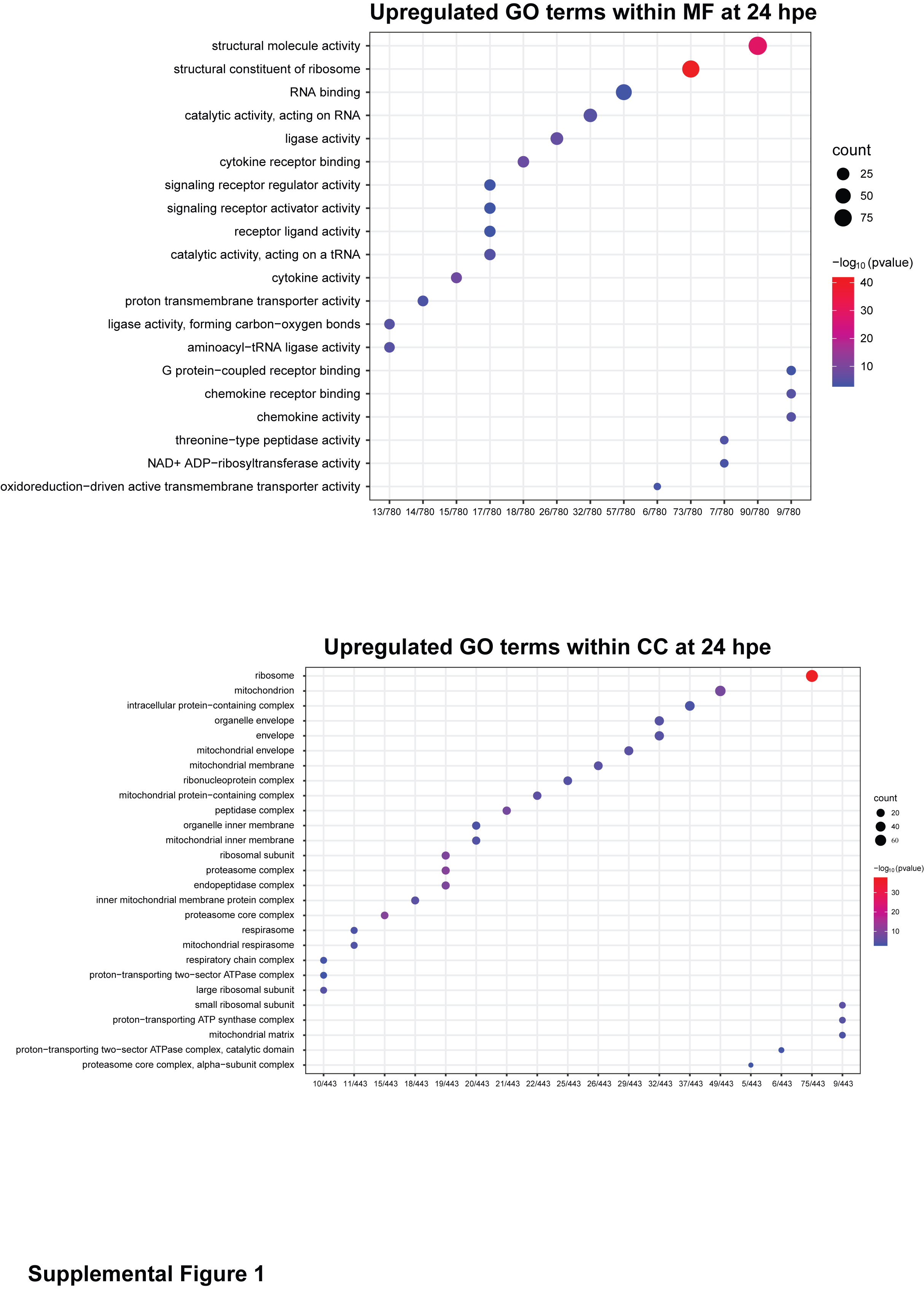

Supplement: Supplementary file 1 [file Image_1.tif]
