# Supplementary material for: Transcriptome-wide analyses of early immune responses in lumpfish leukocytes upon stimulation with poly(I:C)
Source: Front Immunol. 2023 Jun 14;14:1198211. doi: 10.3389/fimmu.2023.1198211 (PMC10300353; doi:10.3389/fimmu.2023.1198211)
Supplement: Supplementary file 3 [file Table_1.docx]

**Supplemental Table 1.** Overview of significantly regulated genes at 6,24 hpe and TI *

**Most significantly regulated genes at 6 hpe**

| **ID** | **log2FoldChange** | **P_value (adj)** |
| --- | --- | --- |
| ENSCLMG00005012324 | 5.400159136 | 2.17E-08 |
| ENSCLMG00005001377 | 4.821094105 | 8.35E-36 |
| ENSCLMG00005011066 | 4.5107894 | 1.85E-13 |
| ENSCLMG00005018145 | 4.505708807 | 2.52E-28 |
| ENSCLMG00005019686 | 4.250685483 | 7.31E-19 |
| ENSCLMG00005001410 | 3.885574814 | 1.86E-19 |
| ENSCLMG00005000798 | 3.856992028 | 0.013557505 |
| ENSCLMG00005015980 | 3.414264105 | 4.25E-25 |
| ENSCLMG00005003581 | 3.11640342 | 0.000561517 |
| ENSCLMG00005010671 | 3.075160655 | 6.23E-05 |
| ENSCLMG00005014628 | 3.073258306 | 6.35E-08 |
| ENSCLMG00005011114 | 3.072451252 | 0.00013831 |
| ENSCLMG00005018843 | 3.066666264 | 1.39E-10 |
| ENSCLMG00005011528 | -2.851369332 | 0.010400754 |
| ENSCLMG00005004874 | 2.845843774 | 9.40E-17 |
| ENSCLMG00005015335 | 2.843158578 | 1.57E-39 |
| ENSCLMG00005011538 | 2.797078376 | 0.009280486 |
| ENSCLMG00005017999 | 2.625353956 | 6.50E-11 |
| ENSCLMG00005011543 | 2.613393248 | 7.88E-16 |
| ENSCLMG00005003866 | -2.593082863 | 0.000451326 |
| ENSCLMG00005011922 | 2.549620997 | 1.09E-05 |
| ENSCLMG00005005864 | 2.468228905 | 5.98E-07 |
| ENSCLMG00005008569 | -2.463782131 | 0.0015259 |
| ENSCLMG00005014809 | 2.448419543 | 6.25E-05 |
| ENSCLMG00005010362 | 2.44028775 | 5.24E-07 |
| ENSCLMG00005013626 | 2.425125035 | 0.02199913 |
| ENSCLMG00005006297 | -2.392270163 | 0.001381324 |
| ENSCLMG00005012028 | -2.271076056 | 6.83E-08 |
| ENSCLMG00005019403 | -2.146636185 | 0.015654965 |
| ENSCLMG00005003496 | -2.123402348 | 0.002483831 |
| ENSCLMG00005013890 | 2.082413083 | 7.62E-13 |
| ENSCLMG00005010384 | -2.070470929 | 0.001365445 |
| ENSCLMG00005015653 | 2.068058565 | 0.012624342 |
| ENSCLMG00005012087 | -2.063314042 | 0.019634051 |
| ENSCLMG00005006306 | -2.054068852 | 7.26E-06 |
| ENSCLMG00005002230 | -2.050223641 | 1.65E-12 |
| ENSCLMG00005018963 | -2.048806149 | 0.001204386 |
| ENSCLMG00005000035 | -2.044407693 | 1.45E-11 |
| ENSCLMG00005000948 | -2.038474821 | 1.93E-05 |
| ENSCLMG00005000029 | -2.027250883 | 6.87E-06 |
| ENSCLMG00005013429 | -1.990151177 | 0.014028519 |
| ENSCLMG00005001818 | 1.979297299 | 3.10E-06 |
| ENSCLMG00005005204 | -1.962768671 | 6.47E-05 |
| ENSCLMG00005020274 | -1.960740995 | 6.74E-07 |
| ENSCLMG00005000447 | -1.947224709 | 3.26E-05 |
| ENSCLMG00005002258 | 1.935216628 | 1.03E-05 |
| ENSCLMG00005006299 | -1.926507753 | 0.001361749 |
| ENSCLMG00005016528 | -1.915668997 | 0.0133412 |
| ENSCLMG00005012191 | -1.908757165 | 0.01917751 |
| ENSCLMG00005018745 | 1.908736622 | 0.033573311 |
| ENSCLMG00005008800 | 1.894983092 | 4.43E-06 |
| ENSCLMG00005004519 | -1.846955596 | 1.85E-13 |
| ENSCLMG00005017209 | -1.841123279 | 1.30E-05 |
| ENSCLMG00005022511 | -1.839720806 | 1.67E-05 |
| ENSCLMG00005017401 | -1.816717072 | 3.09E-10 |
| ENSCLMG00005010209 | -1.811797243 | 0.039323679 |
| ENSCLMG00005013248 | -1.810133089 | 3.44E-13 |
| ENSCLMG00005008834 | -1.789020138 | 1.42E-12 |
| ENSCLMG00005010794 | -1.756628689 | 3.44E-13 |
| ENSCLMG00005018126 | -1.741035874 | 1.26E-05 |
| ENSCLMG00005013592 | -1.732979058 | 0.016309114 |
| ENSCLMG00005010553 | -1.722248076 | 0.001413318 |
| ENSCLMG00005011923 | 1.720321606 | 1.72E-10 |
| ENSCLMG00005014745 | -1.717687176 | 1.19E-05 |
| ENSCLMG00005009734 | -1.70899229 | 0.00027687 |
| ENSCLMG00005009114 | -1.707532195 | 0.000849604 |
| ENSCLMG00005016649 | 1.702168239 | 0.049767218 |
| ENSCLMG00005002642 | -1.692268844 | 0.014410559 |
| ENSCLMG00005006790 | 1.692159994 | 0.007787214 |
| ENSCLMG00005007909 | -1.680726151 | 0.033065813 |
| ENSCLMG00005002394 | -1.680575257 | 0.000267206 |
| ENSCLMG00005013464 | 1.666422932 | 0.000109746 |
| ENSCLMG00005011840 | -1.652949372 | 0.015737079 |
| ENSCLMG00005006624 | -1.650976087 | 7.02E-12 |
| ENSCLMG00005011306 | -1.649696614 | 0.005235888 |
| ENSCLMG00005001707 | 1.649537258 | 0.00013831 |
| ENSCLMG00005016339 | -1.64944022 | 0.014410559 |
| ENSCLMG00005001168 | -1.643417552 | 0.005633737 |
| ENSCLMG00005018034 | -1.628864729 | 0.010400754 |
| ENSCLMG00005009885 | -1.622480478 | 0.003333449 |
| ENSCLMG00005002493 | 1.620555772 | 2.40E-05 |
| ENSCLMG00005005697 | -1.619731014 | 0.043825012 |
| ENSCLMG00005016423 | -1.619102506 | 0.018340148 |
| ENSCLMG00005015366 | 1.607663406 | 0.003524917 |
| ENSCLMG00005006548 | 1.607279991 | 1.79E-06 |
| ENSCLMG00005016777 | -1.593588716 | 2.36E-06 |
| ENSCLMG00005019355 | -1.580530567 | 3.85E-05 |
| ENSCLMG00005006686 | 1.580057125 | 0.000372099 |
| ENSCLMG00005019162 | -1.57525362 | 0.001015171 |
| ENSCLMG00005005289 | -1.57257594 | 0.001076427 |
| ENSCLMG00005000659 | -1.5697137 | 0.034726065 |
| ENSCLMG00005010703 | -1.568690025 | 0.0032874 |
| ENSCLMG00005007821 | -1.557843302 | 0.036375247 |
| ENSCLMG00005008930 | -1.55732257 | 9.78E-08 |
| ENSCLMG00005005469 | -1.557247253 | 0.01917751 |
| ENSCLMG00005003201 | 1.553043252 | 0.002639309 |
| ENSCLMG00005004828 | 1.541062869 | 0.007787214 |
| ENSCLMG00005008894 | -1.53623332 | 5.31E-05 |
| ENSCLMG00005004422 | -1.532278942 | 0.007954288 |
| ENSCLMG00005006149 | -1.532222364 | 0.034230404 |
| ENSCLMG00005022526 | -1.527296799 | 0.014028519 |
| ENSCLMG00005002412 | -1.516427682 | 0.002327221 |
| ENSCLMG00005018619 | -1.515295903 | 0.004623988 |
| ENSCLMG00005020206 | 1.514565086 | 3.91E-11 |
| ENSCLMG00005002325 | -1.512983708 | 0.037815948 |
| ENSCLMG00005020602 | -1.491466195 | 2.50E-07 |
| ENSCLMG00005004947 | 1.488797858 | 0.001107685 |
| ENSCLMG00005017224 | 1.482846873 | 0.006811507 |
| ENSCLMG00005010468 | 1.482790071 | 0.009000083 |
| ENSCLMG00005006897 | -1.480593196 | 0.023020106 |
| ENSCLMG00005022205 | 1.47380311 | 0.000683009 |
| ENSCLMG00005013100 | -1.459530552 | 3.10E-06 |
| ENSCLMG00005018853 | -1.450981729 | 0.018974523 |
| ENSCLMG00005004419 | -1.447497426 | 0.005366785 |
| ENSCLMG00005001142 | 1.44062685 | 3.10E-07 |
| ENSCLMG00005010501 | -1.438907484 | 0.008336675 |
| ENSCLMG00005002059 | -1.435863329 | 0.000716932 |
| ENSCLMG00005005949 | -1.435845885 | 0.017250419 |
| ENSCLMG00005005837 | 1.427020407 | 0.024909643 |
| ENSCLMG00005006154 | -1.425448513 | 0.008854316 |
| ENSCLMG00005020820 | 1.425189704 | 0.026320463 |
| ENSCLMG00005018626 | -1.4220374 | 1.11E-10 |
| ENSCLMG00005014178 | -1.418801863 | 0.046230823 |
| ENSCLMG00005002153 | 1.412570662 | 3.22E-08 |
| ENSCLMG00005020627 | -1.407725373 | 1.57E-06 |
| ENSCLMG00005014474 | -1.406954968 | 2.36E-06 |
| ENSCLMG00005013254 | -1.404490894 | 2.88E-05 |
| ENSCLMG00005021035 | -1.400929546 | 0.000849604 |
| ENSCLMG00005008998 | -1.398103797 | 0.003046109 |
| ENSCLMG00005020366 | -1.395926789 | 0.032939351 |
| ENSCLMG00005014967 | -1.393536349 | 4.04E-05 |
| ENSCLMG00005005269 | -1.383787154 | 0.005064245 |
| ENSCLMG00005022745 | -1.383683107 | 1.48E-12 |
| ENSCLMG00005015473 | 1.381192211 | 0.045256435 |
| ENSCLMG00005015692 | -1.380365677 | 2.87E-06 |
| ENSCLMG00005000867 | -1.379661161 | 0.012087336 |
| ENSCLMG00005012163 | -1.37535789 | 4.32E-05 |
| ENSCLMG00005005640 | -1.372027427 | 0.015737079 |
| ENSCLMG00005013128 | -1.366023778 | 3.69E-07 |
| ENSCLMG00005014295 | -1.364755862 | 0.005843931 |
| ENSCLMG00005007092 | -1.359825842 | 0.006379571 |
| ENSCLMG00005007620 | -1.339777865 | 1.10E-06 |
| ENSCLMG00005012561 | -1.338349128 | 0.005714179 |
| ENSCLMG00005013909 | -1.331256544 | 1.48E-06 |
| ENSCLMG00005011775 | -1.327959889 | 1.20E-05 |
| ENSCLMG00005015696 | -1.326783045 | 1.94E-06 |
| ENSCLMG00005000389 | -1.326059999 | 0.04770502 |
| ENSCLMG00005017861 | -1.320189197 | 0.000279393 |
| ENSCLMG00005019356 | -1.317937924 | 0.018880143 |
| ENSCLMG00005015694 | -1.310579255 | 0.005339744 |
| ENSCLMG00005011867 | -1.30982513 | 1.46E-10 |
| ENSCLMG00005003609 | 1.308553027 | 6.85E-05 |
| ENSCLMG00005010499 | -1.305254005 | 0.015594889 |
| ENSCLMG00005021558 | -1.303290347 | 4.04E-05 |
| ENSCLMG00005006676 | -1.297029858 | 0.004544875 |
| ENSCLMG00005009886 | -1.29589931 | 0.001027888 |
| ENSCLMG00005001398 | 1.295298392 | 0.00073896 |
| ENSCLMG00005001543 | -1.286091338 | 1.79E-06 |
| ENSCLMG00005005943 | -1.283432199 | 0.039636088 |
| ENSCLMG00005006574 | -1.272367733 | 0.014564431 |
| ENSCLMG00005004992 | -1.272034131 | 0.002054687 |
| ENSCLMG00005009468 | -1.270658734 | 0.001488315 |
| ENSCLMG00005021303 | -1.262692601 | 9.56E-05 |
| ENSCLMG00005004984 | -1.260611549 | 0.000818946 |
| ENSCLMG00005014658 | -1.260122095 | 0.00525717 |
| ENSCLMG00005008966 | -1.258128931 | 0.029862458 |
| ENSCLMG00005014881 | -1.255083221 | 0.007787214 |
| ENSCLMG00005008216 | -1.254263609 | 0.028235209 |
| ENSCLMG00005009997 | -1.253663499 | 0.002097299 |
| ENSCLMG00005008246 | -1.253260752 | 6.83E-08 |
| ENSCLMG00005010786 | -1.247459365 | 0.029109297 |
| ENSCLMG00005009954 | -1.241568462 | 0.003083431 |
| ENSCLMG00005021375 | -1.238974457 | 0.006556002 |
| ENSCLMG00005009629 | -1.231453153 | 0.025994787 |
| ENSCLMG00005020652 | -1.230789346 | 4.24E-06 |
| ENSCLMG00005006912 | -1.229780635 | 1.10E-06 |
| ENSCLMG00005009896 | -1.227519296 | 0.003316057 |
| ENSCLMG00005004070 | -1.222416768 | 0.023020106 |
| ENSCLMG00005022529 | -1.21825195 | 0.001663912 |
| ENSCLMG00005015621 | -1.217552435 | 0.004406506 |
| ENSCLMG00005020164 | 1.213135622 | 0.003104027 |
| ENSCLMG00005006494 | -1.210019904 | 1.23E-08 |
| ENSCLMG00005016223 | -1.208769683 | 0.001042757 |
| ENSCLMG00005002615 | -1.206779515 | 0.003287197 |
| ENSCLMG00005020640 | -1.204664309 | 0.00013831 |
| ENSCLMG00005019063 | -1.203336828 | 0.004544875 |
| ENSCLMG00005010698 | -1.200638597 | 6.73E-05 |
| ENSCLMG00005000260 | 1.200606722 | 0.014028519 |
| ENSCLMG00005013856 | -1.199759748 | 0.002435073 |
| ENSCLMG00005011313 | -1.192402995 | 0.002263314 |
| ENSCLMG00005020589 | -1.189719767 | 0.000787196 |
| ENSCLMG00005000713 | -1.188771668 | 1.30E-05 |
| ENSCLMG00005019998 | -1.183091634 | 0.002811848 |
| ENSCLMG00005010949 | -1.182230358 | 0.005434103 |
| ENSCLMG00005003292 | -1.178529348 | 0.047907034 |
| ENSCLMG00005010180 | 1.178393377 | 0.000684571 |
| ENSCLMG00005012944 | 1.178254463 | 0.001471382 |
| ENSCLMG00005014420 | -1.176287001 | 0.000101182 |
| ENSCLMG00005000049 | -1.175857301 | 1.55E-05 |
| ENSCLMG00005011563 | 1.1735748 | 0.021166508 |
| ENSCLMG00005017643 | -1.173284709 | 0.000225588 |
| ENSCLMG00005012284 | -1.172262663 | 0.004544875 |
| ENSCLMG00005019259 | -1.172134599 | 0.007675595 |
| ENSCLMG00005017832 | -1.171769657 | 0.00506713 |
| ENSCLMG00005016592 | -1.170210993 | 1.15E-06 |
| ENSCLMG00005009999 | -1.167389689 | 0.035499145 |
| ENSCLMG00005015395 | -1.166107279 | 0.004544875 |
| ENSCLMG00005001940 | -1.163954733 | 0.001639876 |
| ENSCLMG00005010662 | -1.163953277 | 0.00148679 |
| ENSCLMG00005020755 | -1.16303701 | 0.019838508 |
| ENSCLMG00005004892 | -1.161504511 | 1.15E-06 |
| ENSCLMG00005020358 | -1.157973742 | 7.41E-06 |
| ENSCLMG00005005185 | -1.157785528 | 6.18E-06 |
| ENSCLMG00005016396 | -1.153966312 | 0.001004178 |
| ENSCLMG00005007373 | -1.151197614 | 0.007792864 |
| ENSCLMG00005016330 | -1.149926543 | 3.58E-07 |
| ENSCLMG00005011137 | -1.14900184 | 0.000115868 |
| ENSCLMG00005022214 | -1.148018542 | 0.00203445 |
| ENSCLMG00005007940 | -1.144419764 | 0.001204386 |
| ENSCLMG00005019354 | -1.14274132 | 0.000420529 |
| ENSCLMG00005018052 | -1.142662357 | 0.004773755 |
| ENSCLMG00005000632 | -1.142351055 | 0.028781503 |
| ENSCLMG00005007179 | -1.141478312 | 0.022837707 |
| ENSCLMG00005008809 | -1.140227016 | 0.0032874 |
| ENSCLMG00005006128 | -1.13887751 | 0.000147506 |
| ENSCLMG00005016475 | -1.129287496 | 0.000372099 |
| ENSCLMG00005010877 | -1.128065583 | 0.026435215 |
| ENSCLMG00005018936 | -1.12805343 | 0.000117975 |
| ENSCLMG00005007799 | -1.126106969 | 0.013064522 |
| ENSCLMG00005008432 | -1.123561631 | 6.72E-06 |
| ENSCLMG00005008952 | -1.118803514 | 0.026320463 |
| ENSCLMG00005015809 | -1.117009457 | 0.0012593 |
| ENSCLMG00005010280 | -1.116564058 | 0.00243396 |
| ENSCLMG00005002524 | -1.11519118 | 6.33E-08 |
| ENSCLMG00005022038 | -1.114505385 | 0.001156591 |
| ENSCLMG00005009333 | -1.113902246 | 0.015283272 |
| ENSCLMG00005021644 | -1.107433276 | 0.013038339 |
| ENSCLMG00005013521 | -1.104491833 | 0.001221004 |
| ENSCLMG00005011261 | -1.10316782 | 0.044018875 |
| ENSCLMG00005014806 | -1.096933785 | 0.000276332 |
| ENSCLMG00005020742 | -1.095167475 | 0.000355847 |
| ENSCLMG00005020888 | 1.092420495 | 0.00735357 |
| ENSCLMG00005009787 | -1.091221814 | 0.005434103 |
| ENSCLMG00005018788 | -1.090584548 | 0.000499933 |
| ENSCLMG00005021113 | -1.089388543 | 0.00291605 |
| ENSCLMG00005010390 | -1.084812336 | 0.008107061 |
| ENSCLMG00005003287 | -1.079344721 | 5.47E-05 |
| ENSCLMG00005013566 | -1.076798306 | 0.005339744 |
| ENSCLMG00005018392 | -1.07513585 | 0.00687943 |
| ENSCLMG00005004093 | 1.073103768 | 0.005102265 |
| ENSCLMG00005020007 | -1.072228592 | 3.15E-05 |
| ENSCLMG00005013868 | -1.071105257 | 0.037584693 |
| ENSCLMG00005021756 | -1.067415844 | 0.000524428 |
| ENSCLMG00005011182 | -1.066695784 | 0.00169893 |
| ENSCLMG00005004176 | -1.063641047 | 0.003104027 |
| ENSCLMG00005012663 | 1.056717419 | 0.039685773 |
| ENSCLMG00005015532 | -1.05617188 | 0.00487529 |
| ENSCLMG00005018353 | -1.04963426 | 0.000526008 |
| ENSCLMG00005015892 | -1.044052411 | 2.37E-05 |
| ENSCLMG00005017049 | -1.041919267 | 0.006346641 |
| ENSCLMG00005000455 | -1.041849553 | 0.001045535 |
| ENSCLMG00005014445 | -1.039619817 | 0.041113627 |
| ENSCLMG00005010420 | -1.039284297 | 0.041269558 |
| ENSCLMG00005009639 | -1.032883146 | 0.007954288 |
| ENSCLMG00005015649 | -1.028430075 | 2.55E-05 |
| ENSCLMG00005013256 | -1.027564243 | 0.023927911 |
| ENSCLMG00005014257 | -1.027422512 | 0.005323859 |
| ENSCLMG00005004298 | -1.027413131 | 0.009911478 |
| ENSCLMG00005010042 | -1.027284964 | 0.001063741 |
| ENSCLMG00005013721 | -1.026839539 | 0.039636088 |
| ENSCLMG00005006988 | -1.026707327 | 0.00013831 |
| ENSCLMG00005016912 | -1.025055934 | 0.031509354 |
| ENSCLMG00005004122 | -1.024490911 | 0.000223224 |
| ENSCLMG00005000559 | -1.022053709 | 0.003869522 |
| ENSCLMG00005014559 | -1.02034056 | 0.018016015 |
| ENSCLMG00005008252 | -1.010786338 | 0.003316057 |
| ENSCLMG00005010144 | -1.008618172 | 0.027710178 |
| ENSCLMG00005003079 | -1.006631259 | 0.00073896 |
| ENSCLMG00005015934 | -0.998080004 | 1.94E-06 |
| ENSCLMG00005017410 | -0.996516349 | 0.00066042 |
| ENSCLMG00005002729 | -0.993282766 | 0.000853587 |
| ENSCLMG00005013273 | -0.992737462 | 0.004274489 |
| ENSCLMG00005012986 | -0.992562427 | 2.59E-06 |
| ENSCLMG00005008242 | -0.989615265 | 0.035756814 |
| ENSCLMG00005021898 | -0.986360218 | 0.000499933 |
| ENSCLMG00005019050 | 0.985018724 | 0.021315837 |
| ENSCLMG00005014625 | -0.979196411 | 0.047224265 |
| ENSCLMG00005018555 | -0.977318217 | 1.53E-05 |
| ENSCLMG00005005193 | -0.974201379 | 0.017320346 |
| ENSCLMG00005020519 | -0.973158104 | 0.031973904 |
| ENSCLMG00005020722 | -0.971663158 | 5.55E-05 |
| ENSCLMG00005004927 | -0.967818467 | 0.000337627 |
| ENSCLMG00005000057 | -0.967344316 | 0.007792864 |
| ENSCLMG00005002492 | -0.967301546 | 0.027690793 |
| ENSCLMG00005019376 | -0.966077803 | 0.000320203 |
| ENSCLMG00005020721 | -0.965286266 | 5.76E-05 |
| ENSCLMG00005012892 | -0.965281182 | 0.009375032 |
| ENSCLMG00005018831 | -0.963463808 | 0.013038339 |
| ENSCLMG00005020747 | -0.96127331 | 0.008257938 |
| ENSCLMG00005004009 | -0.957926154 | 0.029646784 |
| ENSCLMG00005018786 | 0.957097006 | 0.019704129 |
| ENSCLMG00005000214 | -0.954037511 | 0.004433435 |
| ENSCLMG00005000892 | -0.950430284 | 0.033573311 |
| ENSCLMG00005006748 | -0.943351169 | 0.000388946 |
| ENSCLMG00005021761 | -0.938805917 | 7.24E-05 |
| ENSCLMG00005017576 | -0.937099652 | 0.04787907 |
| ENSCLMG00005007194 | -0.935358479 | 0.02102859 |
| ENSCLMG00005006041 | -0.934847134 | 0.004595677 |
| ENSCLMG00005021226 | -0.934520724 | 0.041890317 |
| ENSCLMG00005005800 | 0.933846672 | 0.012354677 |
| ENSCLMG00005006687 | 0.931585878 | 0.000563915 |
| ENSCLMG00005001786 | -0.928684958 | 0.017616243 |
| ENSCLMG00005002226 | -0.921098821 | 0.02992687 |
| ENSCLMG00005000496 | -0.92107261 | 0.005355962 |
| ENSCLMG00005003088 | -0.91957246 | 0.004274489 |
| ENSCLMG00005009597 | -0.905750306 | 0.00013831 |
| ENSCLMG00005007562 | -0.897446906 | 0.001063741 |
| ENSCLMG00005007056 | -0.893809803 | 0.004274489 |
| ENSCLMG00005007189 | 0.887827115 | 0.003688986 |
| ENSCLMG00005013703 | -0.887540989 | 0.01917751 |
| ENSCLMG00005021887 | -0.8862697 | 0.003735348 |
| ENSCLMG00005003770 | 0.885670959 | 0.029344206 |
| ENSCLMG00005016127 | -0.884994008 | 0.02199913 |
| ENSCLMG00005019945 | -0.882457884 | 0.00227608 |
| ENSCLMG00005007123 | 0.867935296 | 0.014575837 |
| ENSCLMG00005009993 | -0.867782805 | 0.012087336 |
| ENSCLMG00005012423 | -0.867577706 | 0.037641031 |
| ENSCLMG00005000053 | -0.865420849 | 0.020925299 |
| ENSCLMG00005000010 | -0.86539392 | 0.017320346 |
| ENSCLMG00005021559 | -0.862672023 | 0.002893794 |
| ENSCLMG00005005835 | -0.859182268 | 0.034211652 |
| ENSCLMG00005018910 | -0.855465302 | 0.000337301 |
| ENSCLMG00005002464 | -0.850971049 | 0.017329814 |
| ENSCLMG00005019716 | 0.849081787 | 0.001710932 |
| ENSCLMG00005002037 | -0.846267284 | 0.018880143 |
| ENSCLMG00005005202 | -0.843866594 | 0.008107061 |
| ENSCLMG00005002316 | -0.843757409 | 0.027589038 |
| ENSCLMG00005009666 | -0.839978768 | 0.01917751 |
| ENSCLMG00005012454 | -0.835217077 | 0.049958746 |
| ENSCLMG00005018971 | -0.833393363 | 0.036096612 |
| ENSCLMG00005016638 | -0.833149943 | 0.04663314 |
| ENSCLMG00005005506 | -0.826624035 | 0.023428902 |
| ENSCLMG00005020220 | -0.814366215 | 0.023547912 |
| ENSCLMG00005005184 | -0.799785191 | 0.004274489 |
| ENSCLMG00005010941 | -0.798400692 | 0.007153638 |
| ENSCLMG00005012453 | -0.796622234 | 0.03150344 |
| ENSCLMG00005020393 | -0.794809553 | 0.031236225 |
| ENSCLMG00005006613 | -0.793023394 | 0.02925845 |
| ENSCLMG00005022618 | -0.789564542 | 0.024150696 |
| ENSCLMG00005007178 | -0.789416108 | 0.002483831 |
| ENSCLMG00005003598 | -0.788604255 | 0.023737845 |
| ENSCLMG00005008919 | -0.78825966 | 0.013667282 |
| ENSCLMG00005002717 | -0.782421762 | 0.007153638 |
| ENSCLMG00005001490 | -0.781933556 | 0.024909643 |
| ENSCLMG00005010618 | -0.77643161 | 0.018770937 |
| ENSCLMG00005008781 | 0.770005468 | 0.025994787 |
| ENSCLMG00005021470 | 0.769076394 | 0.025994787 |
| ENSCLMG00005003956 | -0.757473089 | 0.01917751 |
| ENSCLMG00005005379 | -0.75163672 | 0.035745315 |
| ENSCLMG00005005884 | -0.74450957 | 0.037797959 |
| ENSCLMG00005006540 | -0.742298457 | 0.003046109 |
| ENSCLMG00005016228 | -0.741718923 | 0.036096612 |
| ENSCLMG00005009083 | -0.736101394 | 0.023840688 |
| ENSCLMG00005012790 | -0.724265974 | 0.044018875 |
| ENSCLMG00005005602 | -0.723704895 | 0.037873172 |
| ENSCLMG00005015237 | -0.723680458 | 0.013788404 |
| ENSCLMG00005016950 | -0.709847131 | 0.036375247 |
| ENSCLMG00005013270 | -0.70103509 | 0.004544875 |
| ENSCLMG00005006320 | -0.679251 | 0.030239689 |
| ENSCLMG00005010219 | -0.674599654 | 0.011304941 |
| ENSCLMG00005011236 | -0.673966991 | 0.020482486 |
| ENSCLMG00005001084 | -0.665142672 | 0.031973904 |
| ENSCLMG00005009925 | -0.649578027 | 0.009280486 |
| ENSCLMG00005013032 | 0.648573874 | 0.015737079 |
| ENSCLMG00005011156 | -0.647874603 | 0.048530726 |
| ENSCLMG00005008767 | -0.645802142 | 0.041762921 |

**Most significantly regulated genes at 24 hpe**

| **ID** | **log2FoldChange** | **P_value(adj)** |
| --- | --- | --- |
| ENSCLMG00005010047 | -4.134874072 | 7.66E-19 |
| ENSCLMG00005008908 | -2.996805598 | 2.97E-39 |
| ENSCLMG00005005309 | -2.93307504 | 6.64E-08 |
| ENSCLMG00005005053 | -2.795731764 | 9.02E-29 |
| ENSCLMG00005008461 | -2.706632144 | 0.000434053 |
| ENSCLMG00005017816 | -2.68650398 | 2.33E-34 |
| ENSCLMG00005021738 | -2.672741192 | 0.00035394 |
| ENSCLMG00005006306 | -2.641139341 | 1.05E-26 |
| ENSCLMG00005006299 | -2.539066411 | 6.85E-06 |
| ENSCLMG00005010703 | -2.501446641 | 1.52E-09 |
| ENSCLMG00005023042 | -2.470744285 | 9.70E-05 |
| ENSCLMG00005022781 | -2.427080623 | 0.000517319 |
| ENSCLMG00005008865 | -2.414127603 | 0.02281298 |
| ENSCLMG00005012282 | -2.412229851 | 0.000130484 |
| ENSCLMG00005000029 | -2.3629385 | 2.52E-26 |
| ENSCLMG00005020274 | -2.351889582 | 1.27E-41 |
| ENSCLMG00005000035 | -2.319227936 | 1.83E-08 |
| ENSCLMG00005019230 | -2.302224237 | 0.004765663 |
| ENSCLMG00005015707 | -2.293264004 | 0.00473458 |
| ENSCLMG00005019589 | -2.127204852 | 7.24E-11 |
| ENSCLMG00005000447 | -2.113019534 | 2.04E-14 |
| ENSCLMG00005012915 | -2.102215592 | 0.000467649 |
| ENSCLMG00005006912 | -2.100128483 | 9.06E-24 |
| ENSCLMG00005006358 | -2.086885848 | 5.15E-07 |
| ENSCLMG00005013429 | -2.07449763 | 0.000331135 |
| ENSCLMG00005012028 | -2.038005973 | 4.09E-08 |
| ENSCLMG00005015621 | -2.027296059 | 5.97E-11 |
| ENSCLMG00005002325 | -2.021883821 | 2.63E-05 |
| ENSCLMG00005013572 | -1.992939724 | 0.006115637 |
| ENSCLMG00005018283 | -1.989877643 | 3.22E-07 |
| ENSCLMG00005001888 | -1.986345778 | 4.20E-05 |
| ENSCLMG00005008240 | -1.980367655 | 0.000399012 |
| ENSCLMG00005015693 | -1.980303162 | 4.49E-09 |
| ENSCLMG00005019007 | -1.925391887 | 0.003196258 |
| ENSCLMG00005005186 | -1.923221614 | 3.12E-07 |
| ENSCLMG00005003866 | -1.901345094 | 0.00147897 |
| ENSCLMG00005005851 | -1.900887763 | 0.004510686 |
| ENSCLMG00005017156 | -1.900731157 | 0.000687009 |
| ENSCLMG00005008016 | -1.898967231 | 3.71E-13 |
| ENSCLMG00005018052 | -1.894767447 | 5.68E-09 |
| ENSCLMG00005015694 | -1.877166851 | 6.76E-13 |
| ENSCLMG00005017830 | -1.871178736 | 1.36E-07 |
| ENSCLMG00005013072 | -1.865684894 | 0.002966966 |
| ENSCLMG00005021455 | -1.850640339 | 0.001886728 |
| ENSCLMG00005013592 | -1.845280639 | 2.60E-08 |
| ENSCLMG00005010499 | -1.841753051 | 2.20E-13 |
| ENSCLMG00005020224 | -1.838224642 | 5.32E-06 |
| ENSCLMG00005017458 | -1.834201594 | 0.003178988 |
| ENSCLMG00005022980 | -1.830027697 | 0.037714438 |
| ENSCLMG00005018520 | -1.826483005 | 0.006377328 |
| ENSCLMG00005000774 | -1.821025893 | 7.75E-16 |
| ENSCLMG00005010794 | -1.820202077 | 1.92E-22 |
| ENSCLMG00005005640 | -1.813907765 | 6.96E-16 |
| ENSCLMG00005011799 | -1.813707878 | 3.49E-15 |
| ENSCLMG00005021888 | -1.812911598 | 2.13E-08 |
| ENSCLMG00005021452 | -1.812616521 | 9.87E-08 |
| ENSCLMG00005022380 | -1.801775522 | 5.86E-16 |
| ENSCLMG00005000701 | -1.800408231 | 0.000789124 |
| ENSCLMG00005010420 | -1.791336949 | 1.58E-21 |
| ENSCLMG00005000948 | -1.78544087 | 9.44E-09 |
| ENSCLMG00005017832 | -1.781255967 | 5.99E-06 |
| ENSCLMG00005002505 | -1.781015574 | 0.000161197 |
| ENSCLMG00005014967 | -1.775793354 | 4.22E-16 |
| ENSCLMG00005017511 | -1.769811915 | 0.001273897 |
| ENSCLMG00005017633 | -1.758905614 | 0.006344965 |
| ENSCLMG00005013254 | -1.734925283 | 1.09E-12 |
| ENSCLMG00005002230 | -1.72966695 | 4.75E-13 |
| ENSCLMG00005009999 | -1.722746209 | 7.96E-07 |
| ENSCLMG00005017002 | -1.722437079 | 0.013771146 |
| ENSCLMG00005009629 | -1.721696538 | 1.53E-13 |
| ENSCLMG00005017781 | -1.721036155 | 0.015286559 |
| ENSCLMG00005008569 | -1.715644913 | 5.81E-06 |
| ENSCLMG00005001743 | -1.707374122 | 0.002083328 |
| ENSCLMG00005016731 | -1.702982017 | 2.44E-05 |
| ENSCLMG00005017049 | -1.699374453 | 5.70E-14 |
| ENSCLMG00005018707 | -1.696822463 | 4.74E-07 |
| ENSCLMG00005015696 | -1.696464109 | 2.27E-15 |
| ENSCLMG00005004519 | -1.691448461 | 3.76E-13 |
| ENSCLMG00005004349 | -1.690540938 | 1.57E-13 |
| ENSCLMG00005003300 | -1.671073094 | 0.011312348 |
| ENSCLMG00005020188 | -1.663936852 | 8.31E-11 |
| ENSCLMG00005007968 | -1.66392448 | 5.33E-05 |
| ENSCLMG00005017289 | -1.653928733 | 0.000907821 |
| ENSCLMG00005007235 | -1.650526189 | 0.002888106 |
| ENSCLMG00005007179 | -1.645648707 | 2.42E-08 |
| ENSCLMG00005000720 | -1.643785276 | 6.31E-12 |
| ENSCLMG00005004136 | -1.637550613 | 0.016424823 |
| ENSCLMG00005000847 | -1.635220842 | 1.97E-11 |
| ENSCLMG00005020488 | -1.623279657 | 3.58E-17 |
| ENSCLMG00005013248 | -1.619550296 | 1.87E-11 |
| ENSCLMG00005021035 | -1.618727992 | 1.43E-14 |
| ENSCLMG00005020075 | -1.612576975 | 3.84E-05 |
| ENSCLMG00005011306 | -1.612201329 | 5.46E-06 |
| ENSCLMG00005008834 | -1.612099875 | 4.65E-18 |
| ENSCLMG00005012129 | -1.599719342 | 0.001477495 |
| ENSCLMG00005009954 | -1.587902037 | 2.72E-09 |
| ENSCLMG00005019273 | -1.577022039 | 0.024050173 |
| ENSCLMG00005010949 | -1.574552157 | 5.86E-16 |
| ENSCLMG00005003055 | -1.57097175 | 0.014102187 |
| ENSCLMG00005020268 | -1.566327698 | 0.000699807 |
| ENSCLMG00005016290 | -1.562213042 | 0.002205647 |
| ENSCLMG00005005378 | -1.561428223 | 0.00057077 |
| ENSCLMG00005016269 | -1.552621017 | 1.64E-08 |
| ENSCLMG00005016876 | -1.552153557 | 9.52E-06 |
| ENSCLMG00005006474 | -1.549416303 | 4.70E-12 |
| ENSCLMG00005001331 | -1.545338287 | 5.57E-09 |
| ENSCLMG00005006235 | -1.536116703 | 0.031268532 |
| ENSCLMG00005012987 | -1.536114406 | 0.038407286 |
| ENSCLMG00005007504 | -1.529425324 | 1.47E-05 |
| ENSCLMG00005015699 | -1.529257366 | 0.022396067 |
| ENSCLMG00005008854 | -1.526674378 | 0.000229066 |
| ENSCLMG00005014826 | -1.525907832 | 4.09E-13 |
| ENSCLMG00005020627 | -1.522674599 | 2.01E-08 |
| ENSCLMG00005000712 | -1.518925567 | 3.08E-07 |
| ENSCLMG00005015654 | -1.515223139 | 0.006996461 |
| ENSCLMG00005019627 | -1.511196706 | 8.50E-06 |
| ENSCLMG00005004298 | -1.508652375 | 5.80E-10 |
| ENSCLMG00005010463 | -1.50635073 | 2.98E-07 |
| ENSCLMG00005008930 | -1.503759172 | 1.99E-13 |
| ENSCLMG00005011441 | -1.502054536 | 8.30E-06 |
| ENSCLMG00005018490 | -1.497961628 | 0.041878131 |
| ENSCLMG00005010501 | -1.496326683 | 2.91E-10 |
| ENSCLMG00005018392 | -1.492807994 | 3.20E-13 |
| ENSCLMG00005016312 | -1.490907253 | 0.000107931 |
| ENSCLMG00005004954 | -1.486093397 | 0.019073666 |
| ENSCLMG00005012191 | -1.482112829 | 0.000194005 |
| ENSCLMG00005009633 | -1.476122609 | 0.000685226 |
| ENSCLMG00005007622 | -1.471179172 | 1.04E-13 |
| ENSCLMG00005013189 | -1.470671822 | 0.000216612 |
| ENSCLMG00005018428 | -1.46587077 | 8.61E-08 |
| ENSCLMG00005013257 | -1.459315 | 4.95E-12 |
| ENSCLMG00005021414 | -1.45663471 | 1.81E-14 |
| ENSCLMG00005007168 | -1.455872067 | 0.001337575 |
| ENSCLMG00005002619 | -1.454721588 | 1.38E-12 |
| ENSCLMG00005013868 | -1.45079901 | 2.32E-12 |
| ENSCLMG00005014178 | -1.445474701 | 0.000387204 |
| ENSCLMG00005002013 | -1.441848112 | 0.000133919 |
| ENSCLMG00005007620 | -1.437839623 | 1.07E-09 |
| ENSCLMG00005018260 | -1.434722464 | 0.005769864 |
| ENSCLMG00005001087 | -1.434109702 | 3.38E-08 |
| ENSCLMG00005016164 | -1.433850448 | 0.045055339 |
| ENSCLMG00005016592 | -1.433339904 | 1.80E-09 |
| ENSCLMG00005019766 | -1.431802066 | 3.72E-13 |
| ENSCLMG00005010553 | -1.42850956 | 0.001174144 |
| ENSCLMG00005022562 | -1.425539328 | 0.001559202 |
| ENSCLMG00005021984 | -1.423102218 | 1.44E-06 |
| ENSCLMG00005006624 | -1.408914225 | 1.38E-09 |
| ENSCLMG00005014474 | -1.405386419 | 9.88E-11 |
| ENSCLMG00005013380 | -1.402757734 | 4.46E-06 |
| ENSCLMG00005018616 | -1.401119484 | 0.02596385 |
| ENSCLMG00005019895 | -1.400223043 | 1.51E-10 |
| ENSCLMG00005008314 | -1.397975956 | 0.000643548 |
| ENSCLMG00005015785 | -1.39311477 | 1.64E-10 |
| ENSCLMG00005009787 | -1.385723672 | 1.09E-06 |
| ENSCLMG00005019259 | -1.37771616 | 6.98E-06 |
| ENSCLMG00005014559 | -1.372912065 | 1.35E-06 |
| ENSCLMG00005007546 | -1.365217978 | 2.89E-09 |
| ENSCLMG00005001895 | -1.358062984 | 0.005530642 |
| ENSCLMG00005007373 | -1.351482359 | 1.72E-13 |
| ENSCLMG00005002060 | -1.348653335 | 7.36E-06 |
| ENSCLMG00005022682 | -1.347800695 | 7.33E-07 |
| ENSCLMG00005008446 | -1.344019701 | 5.30E-07 |
| ENSCLMG00005001907 | -1.338096583 | 4.60E-05 |
| ENSCLMG00005012454 | -1.335423752 | 7.73E-08 |
| ENSCLMG00005019216 | -1.334983276 | 3.60E-08 |
| ENSCLMG00005018936 | -1.334046206 | 2.60E-08 |
| ENSCLMG00005009333 | -1.333109537 | 2.80E-05 |
| ENSCLMG00005009187 | -1.332537153 | 0.034760015 |
| ENSCLMG00005011573 | -1.33203483 | 1.09E-06 |
| ENSCLMG00005002260 | -1.330821463 | 0.006637706 |
| ENSCLMG00005017287 | -1.328057201 | 4.62E-13 |
| ENSCLMG00005021303 | -1.324741729 | 2.54E-07 |
| ENSCLMG00005001046 | -1.324730247 | 0.047220523 |
| ENSCLMG00005012847 | -1.321234056 | 6.68E-07 |
| ENSCLMG00005003801 | -1.320372684 | 0.009449045 |
| ENSCLMG00005006211 | -1.318930733 | 0.012797673 |
| ENSCLMG00005003088 | -1.317774688 | 4.29E-05 |
| ENSCLMG00005002684 | -1.314927253 | 9.31E-05 |
| ENSCLMG00005019162 | -1.311022352 | 8.56E-07 |
| ENSCLMG00005000848 | -1.309754628 | 1.01E-10 |
| ENSCLMG00005021113 | -1.309299736 | 1.23E-13 |
| ENSCLMG00005010662 | -1.306768315 | 5.01E-09 |
| ENSCLMG00005022745 | -1.302872199 | 5.77E-13 |
| ENSCLMG00005000496 | -1.298039268 | 1.19E-06 |
| ENSCLMG00005021886 | -1.295062609 | 1.04E-09 |
| ENSCLMG00005008590 | -1.292975207 | 0.00011153 |
| ENSCLMG00005005642 | -1.29231503 | 0.000404167 |
| ENSCLMG00005021501 | -1.288860482 | 5.40E-05 |
| ENSCLMG00005001218 | -1.287805512 | 4.42E-08 |
| ENSCLMG00005014286 | -1.286228818 | 5.67E-08 |
| ENSCLMG00005020358 | -1.285941221 | 2.06E-09 |
| ENSCLMG00005020313 | -1.285927769 | 0.015070621 |
| ENSCLMG00005012628 | -1.284509831 | 0.000813791 |
| ENSCLMG00005014743 | -1.284410023 | 0.000553779 |
| ENSCLMG00005004835 | -1.28266692 | 2.28E-06 |
| ENSCLMG00005018626 | -1.280957398 | 2.08E-07 |
| ENSCLMG00005001904 | -1.279278226 | 0.001183963 |
| ENSCLMG00005019731 | -1.277057615 | 0.030360922 |
| ENSCLMG00005007201 | -1.276694195 | 0.045779089 |
| ENSCLMG00005005026 | -1.274121594 | 3.29E-08 |
| ENSCLMG00005017865 | -1.272722477 | 0.001258856 |
| ENSCLMG00005020256 | -1.271543434 | 6.71E-08 |
| ENSCLMG00005010027 | -1.269545303 | 0.000199102 |
| ENSCLMG00005015806 | -1.264997825 | 0.001572276 |
| ENSCLMG00005005904 | -1.26456748 | 0.035860192 |
| ENSCLMG00005011547 | -1.262120364 | 2.24E-14 |
| ENSCLMG00005005781 | -1.261056025 | 0.003789213 |
| ENSCLMG00005011691 | -1.260099926 | 0.047809775 |
| ENSCLMG00005010384 | -1.259256738 | 3.37E-05 |
| ENSCLMG00005020277 | -1.257088732 | 1.02E-05 |
| ENSCLMG00005003304 | -1.255656653 | 5.93E-10 |
| ENSCLMG00005000047 | -1.254804916 | 2.14E-05 |
| ENSCLMG00005013580 | -1.254460576 | 9.66E-09 |
| ENSCLMG00005007092 | -1.254128431 | 1.11E-05 |
| ENSCLMG00005014881 | -1.25194493 | 4.11E-07 |
| ENSCLMG00005005941 | -1.251438011 | 0.003471639 |
| ENSCLMG00005017598 | -1.25121224 | 0.000107073 |
| ENSCLMG00005016135 | -1.25038282 | 0.007832066 |
| ENSCLMG00005010500 | -1.24774827 | 4.43E-10 |
| ENSCLMG00005022462 | -1.246056041 | 0.001345324 |
| ENSCLMG00005022870 | -1.245809272 | 2.76E-07 |
| ENSCLMG00005019645 | -1.244708727 | 0.02599214 |
| ENSCLMG00005021596 | -1.243888307 | 0.013628986 |
| ENSCLMG00005003437 | -1.243199913 | 1.03E-07 |
| ENSCLMG00005012822 | -1.2429949 | 0.000858675 |
| ENSCLMG00005006748 | -1.24151448 | 1.67E-08 |
| ENSCLMG00005017410 | -1.238669777 | 1.23E-06 |
| ENSCLMG00005015108 | -1.237144392 | 0.006344965 |
| ENSCLMG00005003219 | -1.232853074 | 1.23E-06 |
| ENSCLMG00005013101 | -1.229035241 | 3.08E-07 |
| ENSCLMG00005014589 | -1.227071333 | 2.31E-06 |
| ENSCLMG00005006609 | -1.224732293 | 1.20E-07 |
| ENSCLMG00005019545 | -1.224348087 | 3.41E-09 |
| ENSCLMG00005015809 | -1.223469642 | 1.31E-07 |
| ENSCLMG00005012202 | -1.220798754 | 0.004029877 |
| ENSCLMG00005017601 | -1.220521903 | 8.08E-09 |
| ENSCLMG00005011632 | -1.219526929 | 7.65E-12 |
| ENSCLMG00005021794 | -1.219370904 | 0.00119212 |
| ENSCLMG00005022098 | -1.218365177 | 0.000656786 |
| ENSCLMG00005020721 | -1.217409753 | 1.35E-09 |
| ENSCLMG00005022401 | -1.217286195 | 0.0212134 |
| ENSCLMG00005021644 | -1.213930481 | 0.00182283 |
| ENSCLMG00005007162 | -1.212227761 | 3.75E-05 |
| ENSCLMG00005006372 | -1.210517849 | 5.16E-12 |
| ENSCLMG00005000776 | -1.205260138 | 3.17E-08 |
| ENSCLMG00005018848 | -1.204495107 | 2.73E-06 |
| ENSCLMG00005015326 | -1.202748651 | 2.94E-09 |
| ENSCLMG00005014745 | -1.201763361 | 2.52E-06 |
| ENSCLMG00005010618 | -1.201755734 | 8.35E-08 |
| ENSCLMG00005014625 | -1.19966052 | 2.77E-07 |
| ENSCLMG00005000113 | -1.197793059 | 6.48E-06 |
| ENSCLMG00005000644 | -1.196314592 | 0.000394677 |
| ENSCLMG00005010978 | -1.195257101 | 1.92E-07 |
| ENSCLMG00005000306 | -1.194883848 | 0.005067734 |
| ENSCLMG00005006294 | -1.194513076 | 0.049179973 |
| ENSCLMG00005016330 | -1.194493059 | 1.34E-10 |
| ENSCLMG00005018353 | -1.191178556 | 0.000348271 |
| ENSCLMG00005016698 | -1.185726204 | 1.15E-08 |
| ENSCLMG00005022038 | -1.185466501 | 7.06E-10 |
| ENSCLMG00005021892 | -1.184437544 | 1.38E-06 |
| ENSCLMG00005015336 | -1.181946278 | 0.001639189 |
| ENSCLMG00005008432 | -1.180143112 | 8.13E-09 |
| ENSCLMG00005021893 | -1.177035879 | 9.27E-10 |
| ENSCLMG00005020037 | -1.176264334 | 0.000172543 |
| ENSCLMG00005021199 | -1.174969543 | 6.41E-07 |
| ENSCLMG00005020591 | -1.174479432 | 0.005902518 |
| ENSCLMG00005011182 | -1.170728499 | 1.28E-08 |
| ENSCLMG00005013512 | -1.168682846 | 2.37E-07 |
| ENSCLMG00005013100 | -1.168249805 | 1.29E-09 |
| ENSCLMG00005020767 | -1.167560707 | 4.77E-05 |
| ENSCLMG00005022823 | -1.16511536 | 0.035860192 |
| ENSCLMG00005006230 | -1.163025517 | 0.000582583 |
| ENSCLMG00005021226 | -1.161056231 | 1.48E-08 |
| ENSCLMG00005006229 | -1.160757699 | 2.91E-05 |
| ENSCLMG00005014434 | -1.160422932 | 7.12E-06 |
| ENSCLMG00005014045 | -1.159866468 | 0.014764535 |
| ENSCLMG00005007491 | -1.159752964 | 0.007079558 |
| ENSCLMG00005009925 | -1.157112776 | 8.06E-08 |
| ENSCLMG00005021420 | -1.155466828 | 0.005131664 |
| ENSCLMG00005010338 | -1.155305885 | 0.005402853 |
| ENSCLMG00005004702 | -1.153704032 | 2.17E-06 |
| ENSCLMG00005000049 | -1.149003128 | 8.34E-06 |
| ENSCLMG00005015601 | -1.148941308 | 1.94E-10 |
| ENSCLMG00005016228 | -1.146069535 | 8.15E-10 |
| ENSCLMG00005011618 | -1.144427866 | 0.041769184 |
| ENSCLMG00005006870 | -1.144225624 | 4.88E-06 |
| ENSCLMG00005017209 | -1.141103343 | 3.49E-06 |
| ENSCLMG00005007875 | -1.140780628 | 0.000258299 |
| ENSCLMG00005004931 | -1.139001697 | 2.09E-07 |
| ENSCLMG00005014242 | -1.134939263 | 0.000127537 |
| ENSCLMG00005021375 | -1.133536814 | 0.009146662 |
| ENSCLMG00005020640 | -1.131847597 | 0.000778682 |
| ENSCLMG00005015202 | -1.131600109 | 1.76E-05 |
| ENSCLMG00005015649 | -1.130031282 | 1.32E-08 |
| ENSCLMG00005021558 | -1.129747643 | 3.04E-07 |
| ENSCLMG00005000053 | -1.127842712 | 0.001423871 |
| ENSCLMG00005005204 | -1.126996962 | 9.37E-11 |
| ENSCLMG00005005289 | -1.125667566 | 1.91E-06 |
| ENSCLMG00005006584 | -1.124416302 | 1.44E-07 |
| ENSCLMG00005009468 | -1.124399867 | 0.000238136 |
| ENSCLMG00005007821 | -1.122276066 | 0.001327995 |
| ENSCLMG00005018788 | -1.120890345 | 7.17E-09 |
| ENSCLMG00005006128 | -1.119938696 | 2.06E-05 |
| ENSCLMG00005015348 | -1.117550776 | 0.010932327 |
| ENSCLMG00005017132 | -1.116860946 | 0.000760948 |
| ENSCLMG00005015598 | -1.116636926 | 2.87E-06 |
| ENSCLMG00005010704 | -1.116418662 | 0.000137012 |
| ENSCLMG00005018235 | -1.115379955 | 1.18E-06 |
| ENSCLMG00005010569 | -1.11533583 | 4.73E-05 |
| ENSCLMG00005015892 | -1.115305165 | 3.81E-08 |
| ENSCLMG00005009181 | -1.11483781 | 0.002089333 |
| ENSCLMG00005001732 | -1.114775416 | 0.005794378 |
| ENSCLMG00005023113 | -1.11222176 | 0.000602698 |
| ENSCLMG00005020265 | -1.111966028 | 3.38E-08 |
| ENSCLMG00005002492 | -1.109418395 | 0.000300917 |
| ENSCLMG00005006123 | -1.107693804 | 9.23E-06 |
| ENSCLMG00005000214 | -1.107347636 | 1.23E-06 |
| ENSCLMG00005017401 | -1.101645847 | 0.003529837 |
| ENSCLMG00005008209 | -1.098935213 | 2.34E-06 |
| ENSCLMG00005014806 | -1.098838269 | 2.02E-10 |
| ENSCLMG00005012453 | -1.097775891 | 2.51E-09 |
| ENSCLMG00005012790 | -1.097514117 | 5.14E-08 |
| ENSCLMG00005009727 | -1.097242344 | 4.93E-05 |
| ENSCLMG00005009928 | -1.096913784 | 0.00019124 |
| ENSCLMG00005022868 | -1.08908062 | 0.012355119 |
| ENSCLMG00005009083 | -1.087104861 | 4.53E-08 |
| ENSCLMG00005008704 | -1.086880917 | 4.75E-05 |
| ENSCLMG00005004984 | -1.086079483 | 0.001249974 |
| ENSCLMG00005011085 | -1.086064646 | 0.003227666 |
| ENSCLMG00005005996 | -1.085603007 | 0.000201708 |
| ENSCLMG00005000168 | -1.085321698 | 2.92E-05 |
| ENSCLMG00005009721 | -1.0850514 | 1.96E-06 |
| ENSCLMG00005015239 | -1.08432726 | 0.00347471 |
| ENSCLMG00005017579 | -1.083132157 | 0.0002971 |
| ENSCLMG00005015645 | -1.082937313 | 0.01287969 |
| ENSCLMG00005020589 | -1.082488021 | 5.92E-06 |
| ENSCLMG00005019489 | -1.080093032 | 0.002413161 |
| ENSCLMG00005012892 | -1.078791336 | 9.58E-06 |
| ENSCLMG00005002571 | -1.078170665 | 7.64E-05 |
| ENSCLMG00005015459 | -1.077560908 | 0.01998149 |
| ENSCLMG00005003596 | -1.074570304 | 0.002250801 |
| ENSCLMG00005018555 | -1.072867809 | 1.76E-06 |
| ENSCLMG00005022457 | -1.072645777 | 2.60E-05 |
| ENSCLMG00005003514 | -1.072300531 | 5.25E-10 |
| ENSCLMG00005005154 | -1.070555731 | 7.87E-06 |
| ENSCLMG00005016113 | -1.068899392 | 0.013966149 |
| ENSCLMG00005010757 | -1.067121407 | 0.00050009 |
| ENSCLMG00005012120 | -1.067067466 | 0.000452084 |
| ENSCLMG00005007707 | -1.064568042 | 8.91E-06 |
| ENSCLMG00005018200 | -1.064433608 | 4.22E-06 |
| ENSCLMG00005010042 | -1.063668258 | 7.29E-06 |
| ENSCLMG00005005105 | -1.063310863 | 1.98E-06 |
| ENSCLMG00005010544 | -1.062351595 | 1.81E-06 |
| ENSCLMG00005021289 | -1.061037807 | 0.026855348 |
| ENSCLMG00005008894 | -1.060659624 | 4.48E-06 |
| ENSCLMG00005022603 | -1.05990081 | 0.003471639 |
| ENSCLMG00005000745 | -1.059726825 | 9.85E-05 |
| ENSCLMG00005002037 | -1.059523115 | 3.58E-05 |
| ENSCLMG00005012561 | -1.058087733 | 0.014217891 |
| ENSCLMG00005022223 | -1.057099725 | 0.014384458 |
| ENSCLMG00005019530 | -1.056673458 | 2.16E-08 |
| ENSCLMG00005004090 | -1.055402649 | 5.01E-06 |
| ENSCLMG00005016777 | -1.055170048 | 9.19E-06 |
| ENSCLMG00005019945 | -1.05452762 | 9.97E-06 |
| ENSCLMG00005001940 | -1.054495413 | 0.001120766 |
| ENSCLMG00005000892 | -1.052552565 | 0.00011615 |
| ENSCLMG00005017915 | -1.052528294 | 1.25E-07 |
| ENSCLMG00005013128 | -1.051250196 | 2.89E-06 |
| ENSCLMG00005002464 | -1.048651598 | 4.21E-08 |
| ENSCLMG00005010826 | -1.048219079 | 0.021518392 |
| ENSCLMG00005016223 | -1.048044051 | 3.13E-07 |
| ENSCLMG00005006540 | -1.047141215 | 4.22E-07 |
| ENSCLMG00005019998 | -1.046514929 | 3.06E-05 |
| ENSCLMG00005007680 | -1.042689322 | 0.000281371 |
| ENSCLMG00005021186 | -1.042571307 | 0.014197954 |
| ENSCLMG00005020889 | -1.042210287 | 0.002463816 |
| ENSCLMG00005019354 | -1.040022648 | 0.000245721 |
| ENSCLMG00005022481 | -1.039586688 | 0.020199081 |
| ENSCLMG00005013521 | -1.039361835 | 9.23E-06 |
| ENSCLMG00005008246 | -1.038530049 | 2.70E-06 |
| ENSCLMG00005012273 | -1.035990584 | 0.000420175 |
| ENSCLMG00005006463 | -1.035879521 | 8.96E-09 |
| ENSCLMG00005021019 | -1.035598365 | 4.04E-07 |
| ENSCLMG00005002580 | -1.035197119 | 0.014142103 |
| ENSCLMG00005014219 | -1.034512607 | 0.000612824 |
| ENSCLMG00005022220 | -1.033599195 | 0.000127824 |
| ENSCLMG00005020220 | -1.033529794 | 4.73E-08 |
| ENSCLMG00005009594 | -1.032924114 | 2.75E-08 |
| ENSCLMG00005007691 | -1.032051235 | 0.001307527 |
| ENSCLMG00005002029 | -1.031545313 | 0.000831427 |
| ENSCLMG00005009114 | -1.031395828 | 1.22E-05 |
| ENSCLMG00005006036 | -1.031341815 | 2.66E-05 |
| ENSCLMG00005002524 | -1.030225089 | 2.50E-10 |
| ENSCLMG00005007934 | -1.028214987 | 3.44E-05 |
| ENSCLMG00005012398 | -1.028147872 | 0.012954978 |
| ENSCLMG00005010717 | -1.02694666 | 6.46E-05 |
| ENSCLMG00005005193 | -1.026788846 | 0.000740168 |
| ENSCLMG00005017382 | -1.025923288 | 0.006720316 |
| ENSCLMG00005003852 | -1.023395033 | 0.001739709 |
| ENSCLMG00005018975 | -1.020783967 | 0.03958419 |
| ENSCLMG00005011927 | -1.02056802 | 2.80E-05 |
| ENSCLMG00005019315 | -1.019534829 | 8.15E-07 |
| ENSCLMG00005006041 | -1.017803021 | 1.38E-06 |
| ENSCLMG00005022067 | -1.01534703 | 6.25E-08 |
| ENSCLMG00005022086 | -1.014633016 | 2.56E-06 |
| ENSCLMG00005012990 | -1.011519173 | 0.000440951 |
| ENSCLMG00005015839 | -1.007536307 | 8.12E-05 |
| ENSCLMG00005004176 | -1.003772119 | 0.001073443 |
| ENSCLMG00005006149 | -1.002996864 | 0.016149246 |
| ENSCLMG00005006706 | -1.002977928 | 6.57E-07 |
| ENSCLMG00005005360 | -1.002816923 | 1.39E-06 |
| ENSCLMG00005006261 | -1.000093091 | 0.000241728 |
| ENSCLMG00005002226 | -0.9993103 | 0.001153342 |
| ENSCLMG00005002434 | -0.998487045 | 0.002599812 |
| ENSCLMG00005022474 | -0.997352578 | 8.55E-05 |
| ENSCLMG00005013063 | -0.997289124 | 0.00153637 |
| ENSCLMG00005010461 | -0.994809956 | 8.55E-05 |
| ENSCLMG00005015569 | -0.994707494 | 0.001317808 |
| ENSCLMG00005020346 | -0.994364211 | 5.28E-05 |
| ENSCLMG00005015692 | -0.994259598 | 7.33E-07 |
| ENSCLMG00005003459 | -0.993725075 | 2.49E-06 |
| ENSCLMG00005010736 | -0.992815573 | 0.00390584 |
| ENSCLMG00005023077 | -0.992798902 | 0.002311395 |
| ENSCLMG00005020772 | -0.992493288 | 0.000423484 |
| ENSCLMG00005019698 | -0.989610278 | 0.001433871 |
| ENSCLMG00005005841 | -0.988521582 | 1.33E-05 |
| ENSCLMG00005011137 | -0.988401733 | 0.001669244 |
| ENSCLMG00005010339 | -0.988120316 | 0.00687839 |
| ENSCLMG00005006805 | -0.985742044 | 1.18E-06 |
| ENSCLMG00005016956 | -0.9853931 | 0.006737324 |
| ENSCLMG00005018870 | -0.98535661 | 0.00493636 |
| ENSCLMG00005003477 | -0.985324977 | 7.79E-07 |
| ENSCLMG00005011438 | -0.984897894 | 0.000371627 |
| ENSCLMG00005017328 | -0.984621594 | 0.000290923 |
| ENSCLMG00005013311 | -0.984386962 | 0.006889405 |
| ENSCLMG00005018201 | -0.983925368 | 0.04262881 |
| ENSCLMG00005014798 | -0.980945123 | 0.000112916 |
| ENSCLMG00005000682 | -0.979845179 | 1.88E-05 |
| ENSCLMG00005001117 | -0.978908786 | 1.39E-05 |
| ENSCLMG00005004422 | -0.978805534 | 0.022642736 |
| ENSCLMG00005017576 | -0.977086256 | 4.46E-06 |
| ENSCLMG00005021498 | -0.976145208 | 1.11E-05 |
| ENSCLMG00005022810 | -0.975562508 | 0.000231337 |
| ENSCLMG00005010411 | -0.972803873 | 0.001629213 |
| ENSCLMG00005004240 | -0.971354217 | 0.001816274 |
| ENSCLMG00005008589 | -0.971053294 | 0.043655355 |
| ENSCLMG00005019110 | -0.970418968 | 0.00309789 |
| ENSCLMG00005013333 | -0.970311843 | 0.010306606 |
| ENSCLMG00005020804 | -0.969717733 | 0.007148024 |
| ENSCLMG00005012538 | -0.969446034 | 1.07E-06 |
| ENSCLMG00005022065 | -0.965914781 | 9.81E-07 |
| ENSCLMG00005003305 | -0.965573207 | 0.002028313 |
| ENSCLMG00005007203 | -0.965426531 | 7.75E-06 |
| ENSCLMG00005013656 | -0.965309419 | 0.00063095 |
| ENSCLMG00005022529 | -0.964837615 | 0.001916161 |
| ENSCLMG00005000140 | -0.964615104 | 0.010623302 |
| ENSCLMG00005000277 | -0.962666324 | 0.000142702 |
| ENSCLMG00005004992 | -0.962236646 | 3.08E-05 |
| ENSCLMG00005012941 | -0.961684578 | 0.035631665 |
| ENSCLMG00005007563 | -0.961455769 | 2.24E-05 |
| ENSCLMG00005021702 | -0.961006386 | 0.003900715 |
| ENSCLMG00005009439 | -0.960927847 | 0.007753474 |
| ENSCLMG00005012864 | -0.960579615 | 5.79E-06 |
| ENSCLMG00005018136 | -0.960374909 | 0.002827478 |
| ENSCLMG00005022960 | -0.960305791 | 0.002502882 |
| ENSCLMG00005008919 | -0.958637515 | 9.58E-06 |
| ENSCLMG00005005269 | -0.958247608 | 0.009592264 |
| ENSCLMG00005003633 | -0.957766523 | 3.24E-05 |
| ENSCLMG00005013008 | -0.955797126 | 0.005947982 |
| ENSCLMG00005002001 | -0.954248703 | 0.002468621 |
| ENSCLMG00005014730 | -0.954065167 | 1.13E-06 |
| ENSCLMG00005019734 | -0.953013988 | 0.003781359 |
| ENSCLMG00005023009 | -0.952407491 | 4.59E-05 |
| ENSCLMG00005016803 | -0.951493149 | 0.002588029 |
| ENSCLMG00005013703 | -0.951194431 | 1.82E-05 |
| ENSCLMG00005023017 | -0.95035294 | 0.000517319 |
| ENSCLMG00005002316 | -0.949857249 | 0.000249057 |
| ENSCLMG00005021472 | -0.949571631 | 0.000629975 |
| ENSCLMG00005013909 | -0.948295755 | 0.000293162 |
| ENSCLMG00005013856 | -0.946296513 | 5.58E-05 |
| ENSCLMG00005020602 | -0.946191969 | 3.26E-07 |
| ENSCLMG00005017981 | -0.944090017 | 0.001704102 |
| ENSCLMG00005018246 | -0.943167593 | 0.041115018 |
| ENSCLMG00005005184 | -0.942215305 | 0.000132655 |
| ENSCLMG00005002259 | -0.937008723 | 0.005503276 |
| ENSCLMG00005014951 | -0.934812329 | 0.002019838 |
| ENSCLMG00005013240 | -0.934597547 | 0.003005711 |
| ENSCLMG00005007562 | -0.934235439 | 3.99E-05 |
| ENSCLMG00005015872 | -0.933583817 | 0.000104265 |
| ENSCLMG00005021887 | -0.932352528 | 3.42E-05 |
| ENSCLMG00005000010 | -0.932063132 | 6.64E-05 |
| ENSCLMG00005003033 | -0.931774036 | 0.000104243 |
| ENSCLMG00005022591 | -0.930871519 | 0.000153112 |
| ENSCLMG00005000844 | -0.92804718 | 0.014106159 |
| ENSCLMG00005008196 | -0.926278453 | 0.000317097 |
| ENSCLMG00005001084 | -0.925981169 | 1.44E-05 |
| ENSCLMG00005000713 | -0.924856643 | 7.61E-05 |
| ENSCLMG00005009974 | -0.924491002 | 6.30E-05 |
| ENSCLMG00005015225 | -0.923992379 | 1.92E-06 |
| ENSCLMG00005000614 | -0.923617342 | 0.001515006 |
| ENSCLMG00005016293 | -0.920283866 | 0.011962644 |
| ENSCLMG00005003956 | -0.919084543 | 1.24E-06 |
| ENSCLMG00005010906 | -0.918888321 | 0.010598905 |
| ENSCLMG00005004587 | -0.915274886 | 0.010776896 |
| ENSCLMG00005020774 | -0.915103772 | 0.000740168 |
| ENSCLMG00005018202 | -0.914750185 | 0.015591482 |
| ENSCLMG00005014343 | -0.914493804 | 0.008430424 |
| ENSCLMG00005020490 | -0.912492464 | 0.000403884 |
| ENSCLMG00005000865 | -0.911947445 | 0.008204046 |
| ENSCLMG00005022475 | -0.911857269 | 0.000689763 |
| ENSCLMG00005004894 | -0.910787318 | 0.001598468 |
| ENSCLMG00005015231 | -0.910327684 | 0.00020655 |
| ENSCLMG00005020747 | -0.909868701 | 0.000216732 |
| ENSCLMG00005001020 | -0.909815928 | 8.61E-07 |
| ENSCLMG00005018542 | -0.909359041 | 0.013966149 |
| ENSCLMG00005012219 | -0.907575649 | 6.95E-05 |
| ENSCLMG00005006988 | -0.907534798 | 0.00031036 |
| ENSCLMG00005016063 | -0.907326187 | 0.001373763 |
| ENSCLMG00005015575 | -0.90657639 | 0.031575983 |
| ENSCLMG00005012163 | -0.905538423 | 0.008702588 |
| ENSCLMG00005020192 | -0.904485048 | 7.73E-05 |
| ENSCLMG00005009618 | -0.903183048 | 0.001771366 |
| ENSCLMG00005017375 | -0.900422788 | 0.000283695 |
| ENSCLMG00005018853 | -0.899233068 | 0.01477859 |
| ENSCLMG00005010428 | -0.897760092 | 0.043730824 |
| ENSCLMG00005005809 | -0.897486644 | 0.000857173 |
| ENSCLMG00005000415 | -0.896767851 | 4.59E-05 |
| ENSCLMG00005008998 | -0.896250335 | 0.000248543 |
| ENSCLMG00005007056 | -0.896068927 | 7.30E-05 |
| ENSCLMG00005003850 | -0.895013097 | 0.003457479 |
| ENSCLMG00005020419 | -0.894857929 | 0.003603635 |
| ENSCLMG00005007101 | -0.893203208 | 0.000811506 |
| ENSCLMG00005020325 | -0.893193415 | 0.000177625 |
| ENSCLMG00005014945 | -0.893143798 | 0.000690164 |
| ENSCLMG00005004892 | -0.891911526 | 0.00043231 |
| ENSCLMG00005020754 | -0.891829894 | 0.001486204 |
| ENSCLMG00005020789 | -0.891253614 | 0.003789438 |
| ENSCLMG00005013256 | -0.890929733 | 0.008535936 |
| ENSCLMG00005015367 | -0.889443181 | 1.90E-05 |
| ENSCLMG00005013704 | -0.887505915 | 0.015591482 |
| ENSCLMG00005005949 | -0.883901345 | 0.030899596 |
| ENSCLMG00005002729 | -0.881386253 | 0.000244083 |
| ENSCLMG00005005301 | -0.880296125 | 0.021344487 |
| ENSCLMG00005004008 | -0.879322788 | 0.01538473 |
| ENSCLMG00005013273 | -0.879203829 | 0.000237815 |
| ENSCLMG00005000221 | -0.878665943 | 3.49E-06 |
| ENSCLMG00005018426 | -0.878459194 | 0.009913404 |
| ENSCLMG00005012685 | -0.877442591 | 0.000926297 |
| ENSCLMG00005001924 | -0.877296474 | 0.011375496 |
| ENSCLMG00005016950 | -0.87567525 | 0.000957457 |
| ENSCLMG00005021259 | -0.87546363 | 0.010545033 |
| ENSCLMG00005003045 | -0.873083358 | 1.94E-05 |
| ENSCLMG00005006930 | -0.872613988 | 0.020048571 |
| ENSCLMG00005014451 | -0.869215238 | 0.011901194 |
| ENSCLMG00005001490 | -0.86759324 | 0.0059401 |
| ENSCLMG00005019355 | -0.867442832 | 0.008507429 |
| ENSCLMG00005010919 | -0.866483556 | 0.022040997 |
| ENSCLMG00005021625 | -0.86453169 | 0.004282168 |
| ENSCLMG00005008370 | -0.86273607 | 0.018365251 |
| ENSCLMG00005016249 | -0.861826812 | 0.000587739 |
| ENSCLMG00005021756 | -0.861599286 | 5.15E-05 |
| ENSCLMG00005004420 | -0.860074834 | 0.004880243 |
| ENSCLMG00005011867 | -0.859782044 | 2.70E-06 |
| ENSCLMG00005010796 | -0.859548996 | 0.037904346 |
| ENSCLMG00005018122 | -0.85918943 | 0.000259256 |
| ENSCLMG00005009734 | -0.858945458 | 0.010307913 |
| ENSCLMG00005011308 | -0.858310345 | 0.008211869 |
| ENSCLMG00005008365 | -0.855889344 | 2.22E-05 |
| ENSCLMG00005011310 | -0.855010368 | 0.001947273 |
| ENSCLMG00005007178 | -0.854764774 | 8.75E-05 |
| ENSCLMG00005020652 | -0.854010344 | 2.14E-05 |
| ENSCLMG00005022511 | -0.853302301 | 0.030381754 |
| ENSCLMG00005015718 | -0.852586906 | 8.27E-05 |
| ENSCLMG00005004995 | -0.851266771 | 0.000900128 |
| ENSCLMG00005006136 | -0.85028356 | 2.63E-05 |
| ENSCLMG00005002437 | -0.848986412 | 0.023542294 |
| ENSCLMG00005010425 | -0.848916994 | 0.018396957 |
| ENSCLMG00005007405 | -0.848755717 | 0.00089246 |
| ENSCLMG00005017684 | -0.848594026 | 0.011819033 |
| ENSCLMG00005011813 | -0.848395007 | 0.005503276 |
| ENSCLMG00005002061 | -0.848285281 | 0.009841374 |
| ENSCLMG00005007971 | -0.848011918 | 1.12E-05 |
| ENSCLMG00005012867 | -0.846882022 | 0.000350997 |
| ENSCLMG00005000559 | -0.846869806 | 2.56E-05 |
| ENSCLMG00005002999 | -0.846776067 | 0.000140533 |
| ENSCLMG00005002615 | -0.843586852 | 0.001089796 |
| ENSCLMG00005017734 | -0.842215063 | 0.000190995 |
| ENSCLMG00005020739 | -0.841533313 | 0.008691625 |
| ENSCLMG00005016246 | -0.838082717 | 0.001621806 |
| ENSCLMG00005005202 | -0.837510399 | 0.004092278 |
| ENSCLMG00005007354 | -0.836837761 | 0.000199102 |
| ENSCLMG00005002136 | -0.83640305 | 0.004007699 |
| ENSCLMG00005015733 | -0.835640158 | 0.004014893 |
| ENSCLMG00005020460 | -0.834832437 | 0.000444939 |
| ENSCLMG00005021559 | -0.834434697 | 1.84E-06 |
| ENSCLMG00005006402 | -0.833624697 | 0.006820607 |
| ENSCLMG00005017528 | -0.833519168 | 0.000576043 |
| ENSCLMG00005010680 | -0.83329148 | 0.008296611 |
| ENSCLMG00005003079 | -0.832105623 | 0.010307913 |
| ENSCLMG00005001543 | -0.831610329 | 0.001372989 |
| ENSCLMG00005022427 | -0.831019786 | 0.010121619 |
| ENSCLMG00005013807 | -0.830373522 | 0.007938726 |
| ENSCLMG00005017475 | -0.830146502 | 0.000261704 |
| ENSCLMG00005019677 | -0.829707059 | 0.003196258 |
| ENSCLMG00005014996 | -0.828338423 | 0.000293808 |
| ENSCLMG00005007097 | -0.827292231 | 4.80E-05 |
| ENSCLMG00005001882 | -0.826218403 | 8.01E-05 |
| ENSCLMG00005015395 | -0.824805267 | 0.023190447 |
| ENSCLMG00005016638 | -0.824591993 | 0.000516015 |
| ENSCLMG00005005000 | -0.823070917 | 8.64E-05 |
| ENSCLMG00005019490 | -0.822738134 | 0.002966966 |
| ENSCLMG00005011497 | -0.822237002 | 0.000211001 |
| ENSCLMG00005007960 | -0.822058138 | 1.07E-05 |
| ENSCLMG00005016927 | -0.821141835 | 1.23E-05 |
| ENSCLMG00005010409 | -0.820334444 | 0.011070702 |
| ENSCLMG00005016778 | -0.820150566 | 0.016513301 |
| ENSCLMG00005013270 | -0.819451583 | 9.43E-05 |
| ENSCLMG00005008002 | -0.818920118 | 0.017087758 |
| ENSCLMG00005011840 | -0.818646684 | 0.028050953 |
| ENSCLMG00005006391 | -0.816885405 | 0.036941047 |
| ENSCLMG00005014467 | -0.816551799 | 0.01610654 |
| ENSCLMG00005005087 | -0.815861781 | 0.00079899 |
| ENSCLMG00005019881 | -0.815641671 | 0.011705132 |
| ENSCLMG00005009886 | -0.814493218 | 0.000217919 |
| ENSCLMG00005021898 | -0.814250137 | 0.008165911 |
| ENSCLMG00005000659 | -0.813881972 | 0.001302111 |
| ENSCLMG00005011427 | -0.813830036 | 2.18E-05 |
| ENSCLMG00005021749 | -0.813148574 | 0.000121873 |
| ENSCLMG00005017173 | -0.812951849 | 0.000293974 |
| ENSCLMG00005021654 | -0.812807822 | 0.023414384 |
| ENSCLMG00005001874 | -0.811341867 | 6.76E-06 |
| ENSCLMG00005008848 | -0.811226634 | 0.026206333 |
| ENSCLMG00005022323 | -0.810484787 | 0.000217052 |
| ENSCLMG00005022214 | -0.809515486 | 0.00135331 |
| ENSCLMG00005012164 | -0.808844698 | 0.00571734 |
| ENSCLMG00005008767 | -0.807334218 | 0.001438521 |
| ENSCLMG00005004512 | -0.806300925 | 6.95E-05 |
| ENSCLMG00005006078 | -0.806163031 | 0.000172543 |
| ENSCLMG00005001835 | -0.805072947 | 0.000515581 |
| ENSCLMG00005000043 | -0.804953593 | 6.17E-06 |
| ENSCLMG00005016182 | -0.804671962 | 0.000108066 |
| ENSCLMG00005007503 | -0.804495894 | 0.027767533 |
| ENSCLMG00005015503 | -0.804443061 | 2.49E-05 |
| ENSCLMG00005014413 | -0.804306943 | 0.000366677 |
| ENSCLMG00005011554 | -0.804305759 | 4.29E-05 |
| ENSCLMG00005006734 | -0.804295972 | 0.03581259 |
| ENSCLMG00005008336 | -0.804059624 | 0.014036131 |
| ENSCLMG00005021386 | -0.801443809 | 0.003200067 |
| ENSCLMG00005019376 | -0.801414873 | 5.84E-05 |
| ENSCLMG00005018751 | -0.801377804 | 0.002459595 |
| ENSCLMG00005003154 | -0.801341085 | 0.006967397 |
| ENSCLMG00005020393 | -0.801143232 | 0.000925942 |
| ENSCLMG00005021563 | -0.801007758 | 0.000529819 |
| ENSCLMG00005011857 | -0.800694792 | 0.002593966 |
| ENSCLMG00005016590 | -0.800002816 | 0.017381839 |
| ENSCLMG00005003241 | -0.799744963 | 0.018453145 |
| ENSCLMG00005012878 | -0.79941744 | 0.001931143 |
| ENSCLMG00005023039 | -0.799369738 | 0.004168385 |
| ENSCLMG00005011115 | -0.798732166 | 0.000674632 |
| ENSCLMG00005021610 | -0.798697779 | 0.001466862 |
| ENSCLMG00005015808 | -0.798359052 | 0.000113186 |
| ENSCLMG00005016419 | -0.795958097 | 0.002751309 |
| ENSCLMG00005015021 | -0.795699662 | 0.00592888 |
| ENSCLMG00005000499 | -0.79495399 | 3.52E-05 |
| ENSCLMG00005014378 | -0.793695839 | 0.000487086 |
| ENSCLMG00005020506 | -0.792639451 | 0.00013755 |
| ENSCLMG00005010280 | -0.792547314 | 0.01844864 |
| ENSCLMG00005011775 | -0.791885645 | 0.002087159 |
| ENSCLMG00005012653 | -0.790120607 | 0.02538587 |
| ENSCLMG00005010714 | -0.788472255 | 0.04555699 |
| ENSCLMG00005015236 | -0.788151818 | 0.008011851 |
| ENSCLMG00005002412 | -0.787210247 | 0.017879774 |
| ENSCLMG00005005127 | -0.786669507 | 0.004125079 |
| ENSCLMG00005017021 | -0.786182783 | 7.00E-06 |
| ENSCLMG00005014176 | -0.782193415 | 0.00825242 |
| ENSCLMG00005020135 | -0.781970602 | 1.08E-05 |
| ENSCLMG00005023021 | -0.781915878 | 0.000583426 |
| ENSCLMG00005018845 | -0.781658923 | 0.01269884 |
| ENSCLMG00005007791 | -0.781031667 | 0.043343115 |
| ENSCLMG00005000321 | -0.779854328 | 0.000740168 |
| ENSCLMG00005007084 | -0.779710162 | 0.010973489 |
| ENSCLMG00005022240 | -0.778784936 | 0.023473861 |
| ENSCLMG00005001021 | -0.778694631 | 0.001299807 |
| ENSCLMG00005014420 | -0.778503956 | 0.000589713 |
| ENSCLMG00005013312 | -0.778094315 | 0.001816274 |
| ENSCLMG00005017854 | -0.77786154 | 3.14E-05 |
| ENSCLMG00005021216 | -0.777233376 | 0.00063358 |
| ENSCLMG00005006613 | -0.777178103 | 0.000993131 |
| ENSCLMG00005001321 | -0.776373486 | 0.001307527 |
| ENSCLMG00005015511 | -0.775996955 | 0.015212202 |
| ENSCLMG00005020958 | -0.775295721 | 0.04467932 |
| ENSCLMG00005021965 | -0.774863663 | 0.036496108 |
| ENSCLMG00005000673 | -0.774713307 | 0.001916161 |
| ENSCLMG00005001729 | -0.773902137 | 0.011095184 |
| ENSCLMG00005013460 | -0.772328033 | 0.000180611 |
| ENSCLMG00005011368 | -0.771301734 | 2.30E-05 |
| ENSCLMG00005020866 | -0.771010583 | 0.003700348 |
| ENSCLMG00005016006 | -0.770202976 | 0.000403348 |
| ENSCLMG00005009963 | -0.767535756 | 0.002832912 |
| ENSCLMG00005020586 | -0.767271758 | 3.95E-05 |
| ENSCLMG00005001585 | -0.766274348 | 3.19E-05 |
| ENSCLMG00005001869 | -0.765468497 | 0.027053359 |
| ENSCLMG00005004862 | -0.765315005 | 0.00230487 |
| ENSCLMG00005012284 | -0.764141019 | 0.003906636 |
| ENSCLMG00005009239 | -0.763888136 | 0.012950815 |
| ENSCLMG00005009597 | -0.763708493 | 0.000102429 |
| ENSCLMG00005016774 | -0.762015251 | 0.00010325 |
| ENSCLMG00005013530 | -0.76172249 | 0.000689763 |
| ENSCLMG00005016076 | -0.759696239 | 0.001585927 |
| ENSCLMG00005022536 | -0.759422222 | 0.000908833 |
| ENSCLMG00005010442 | -0.759167876 | 0.004446686 |
| ENSCLMG00005005853 | -0.758179294 | 0.000136835 |
| ENSCLMG00005022489 | -0.757429158 | 0.013214928 |
| ENSCLMG00005020078 | -0.756197009 | 0.000142702 |
| ENSCLMG00005013020 | -0.75618069 | 0.002560605 |
| ENSCLMG00005009154 | -0.756115114 | 0.036400468 |
| ENSCLMG00005000150 | -0.753656346 | 0.012759072 |
| ENSCLMG00005021618 | -0.753417367 | 0.000217052 |
| ENSCLMG00005000893 | -0.753302135 | 0.000641016 |
| ENSCLMG00005010549 | -0.753137581 | 0.017255042 |
| ENSCLMG00005000455 | -0.752761995 | 0.020512424 |
| ENSCLMG00005021809 | -0.752327071 | 0.00356606 |
| ENSCLMG00005003193 | -0.751913966 | 0.011511659 |
| ENSCLMG00005011167 | -0.750914328 | 0.000640507 |
| ENSCLMG00005016534 | -0.750868427 | 0.000778896 |
| ENSCLMG00005014970 | -0.749910188 | 0.001276319 |
| ENSCLMG00005013665 | -0.74872211 | 0.002816981 |
| ENSCLMG00005020007 | -0.748601479 | 3.46E-05 |
| ENSCLMG00005021159 | -0.748440229 | 0.009750315 |
| ENSCLMG00005002297 | -0.747650698 | 0.033847065 |
| ENSCLMG00005022295 | -0.747492421 | 0.029224556 |
| ENSCLMG00005009666 | -0.746792923 | 0.005118909 |
| ENSCLMG00005006340 | -0.746663962 | 0.023493118 |
| ENSCLMG00005006319 | -0.744122623 | 0.017274163 |
| ENSCLMG00005017483 | -0.74361796 | 8.99E-05 |
| ENSCLMG00005008803 | -0.742693003 | 0.004135591 |
| ENSCLMG00005019063 | -0.742118563 | 0.001883404 |
| ENSCLMG00005022706 | -0.741666728 | 0.011602774 |
| ENSCLMG00005005840 | -0.741619574 | 0.001551567 |
| ENSCLMG00005019768 | -0.74159703 | 0.001130671 |
| ENSCLMG00005003774 | -0.739842768 | 0.03891644 |
| ENSCLMG00005020722 | -0.738408384 | 0.001129605 |
| ENSCLMG00005019637 | -0.738373587 | 0.00731809 |
| ENSCLMG00005016064 | -0.735938546 | 0.038165777 |
| ENSCLMG00005001489 | -0.735789622 | 0.008876755 |
| ENSCLMG00005022138 | -0.735667159 | 0.04878416 |
| ENSCLMG00005005136 | -0.735562156 | 0.022169796 |
| ENSCLMG00005020784 | -0.734843276 | 0.022717765 |
| ENSCLMG00005018963 | -0.73478997 | 0.028337289 |
| ENSCLMG00005021319 | -0.73359161 | 0.007581058 |
| ENSCLMG00005012495 | -0.733457484 | 0.030381754 |
| ENSCLMG00005021761 | -0.73251625 | 1.69E-05 |
| ENSCLMG00005007312 | -0.73226617 | 0.002515159 |
| ENSCLMG00005018600 | -0.732077342 | 0.002044678 |
| ENSCLMG00005022234 | -0.732039375 | 0.040662085 |
| ENSCLMG00005001062 | -0.731537967 | 0.035695402 |
| ENSCLMG00005003071 | -0.730024439 | 0.002311395 |
| ENSCLMG00005016863 | -0.729641929 | 0.002858256 |
| ENSCLMG00005019636 | -0.729575864 | 0.007785618 |
| ENSCLMG00005008216 | -0.729478228 | 0.035750648 |
| ENSCLMG00005014820 | -0.729150542 | 0.047175469 |
| ENSCLMG00005001935 | -0.728682042 | 0.000548436 |
| ENSCLMG00005014198 | -0.728175903 | 0.032634238 |
| ENSCLMG00005019657 | -0.727143382 | 0.001805831 |
| ENSCLMG00005017361 | -0.727066108 | 0.005127128 |
| ENSCLMG00005007043 | -0.724896958 | 0.007041434 |
| ENSCLMG00005011993 | -0.724207144 | 0.025397969 |
| ENSCLMG00005002038 | -0.723684051 | 0.0002459 |
| ENSCLMG00005014236 | -0.723323301 | 0.002917141 |
| ENSCLMG00005004830 | -0.722797358 | 0.015591482 |
| ENSCLMG00005012037 | -0.722678939 | 0.006506222 |
| ENSCLMG00005007940 | -0.721886457 | 0.002882472 |
| ENSCLMG00005006400 | -0.721584301 | 0.003733612 |
| ENSCLMG00005011747 | -0.720933405 | 0.003963243 |
| ENSCLMG00005020809 | -0.720833231 | 0.006628814 |
| ENSCLMG00005010024 | -0.72058946 | 0.01210906 |
| ENSCLMG00005010219 | -0.719137793 | 0.000151156 |
| ENSCLMG00005012268 | -0.719071778 | 0.002883657 |
| ENSCLMG00005020800 | -0.71863503 | 0.00593576 |
| ENSCLMG00005010557 | -0.718081159 | 0.024691171 |
| ENSCLMG00005014346 | -0.717925792 | 0.014537385 |
| ENSCLMG00005015918 | -0.717710202 | 0.008596838 |
| ENSCLMG00005007426 | -0.716418932 | 0.001322701 |
| ENSCLMG00005020582 | -0.716394646 | 0.005709436 |
| ENSCLMG00005018005 | -0.716020994 | 0.015445534 |
| ENSCLMG00005022453 | -0.715858442 | 0.000366677 |
| ENSCLMG00005006494 | -0.714313463 | 0.004217931 |
| ENSCLMG00005003066 | -0.712732237 | 0.016737167 |
| ENSCLMG00005020340 | -0.712399908 | 0.00709425 |
| ENSCLMG00005010889 | -0.711629508 | 0.019989515 |
| ENSCLMG00005016156 | -0.711152202 | 0.01103911 |
| ENSCLMG00005005185 | -0.709753706 | 0.000528026 |
| ENSCLMG00005010095 | -0.709044466 | 0.043486557 |
| ENSCLMG00005010849 | -0.708870536 | 0.004799596 |
| ENSCLMG00005003289 | -0.708818591 | 0.023580985 |
| ENSCLMG00005015530 | -0.708358562 | 0.012869582 |
| ENSCLMG00005014227 | -0.708246156 | 0.000906 |
| ENSCLMG00005016660 | -0.707247689 | 0.016962406 |
| ENSCLMG00005015323 | -0.70662383 | 0.00099507 |
| ENSCLMG00005001803 | -0.706531772 | 0.015993275 |
| ENSCLMG00005005574 | -0.70542659 | 0.005719054 |
| ENSCLMG00005018507 | -0.705230812 | 0.023189173 |
| ENSCLMG00005005002 | -0.704778007 | 0.005807333 |
| ENSCLMG00005015164 | -0.704062331 | 0.019073666 |
| ENSCLMG00005016396 | -0.702343324 | 0.016999366 |
| ENSCLMG00005007310 | -0.701684141 | 0.000452082 |
| ENSCLMG00005012236 | -0.700948466 | 0.009961221 |
| ENSCLMG00005011289 | -0.700717013 | 0.035560411 |
| ENSCLMG00005003177 | -0.700596611 | 0.035255184 |
| ENSCLMG00005020380 | -0.700254021 | 0.027855509 |
| ENSCLMG00005003308 | -0.699494503 | 0.047695752 |
| ENSCLMG00005015538 | -0.698738782 | 0.000385967 |
| ENSCLMG00005004805 | -0.698126331 | 0.000580414 |
| ENSCLMG00005000492 | -0.697462554 | 0.001441848 |
| ENSCLMG00005000395 | -0.696242746 | 0.000588756 |
| ENSCLMG00005017851 | -0.694939145 | 0.006996461 |
| ENSCLMG00005014197 | -0.694550441 | 0.000588756 |
| ENSCLMG00005010698 | -0.693892917 | 0.00797653 |
| ENSCLMG00005003086 | -0.693717607 | 0.008723097 |
| ENSCLMG00005020842 | -0.691901199 | 0.027855509 |
| ENSCLMG00005008252 | -0.691840312 | 0.00347471 |
| ENSCLMG00005016127 | -0.691815439 | 0.017348373 |
| ENSCLMG00005016547 | -0.691389671 | 0.007759581 |
| ENSCLMG00005014673 | -0.690772124 | 0.010710342 |
| ENSCLMG00005012986 | -0.687591676 | 0.013966149 |
| ENSCLMG00005017945 | -0.687483057 | 0.008595321 |
| ENSCLMG00005012100 | -0.686664504 | 0.005670513 |
| ENSCLMG00005005454 | -0.686462474 | 0.009484039 |
| ENSCLMG00005022325 | -0.686002217 | 0.011363483 |
| ENSCLMG00005002569 | -0.685890648 | 0.002798287 |
| ENSCLMG00005000302 | -0.685447128 | 0.000485608 |
| ENSCLMG00005021516 | -0.684463007 | 0.027220327 |
| ENSCLMG00005022182 | -0.684312668 | 0.009146662 |
| ENSCLMG00005013535 | -0.684077255 | 0.010746843 |
| ENSCLMG00005014961 | -0.682506445 | 0.023580985 |
| ENSCLMG00005015095 | -0.682505066 | 0.000259256 |
| ENSCLMG00005018910 | -0.681923588 | 0.001921319 |
| ENSCLMG00005003496 | -0.680560911 | 0.036994062 |
| ENSCLMG00005015494 | -0.680350472 | 0.003221133 |
| ENSCLMG00005012964 | -0.679223294 | 0.010818403 |
| ENSCLMG00005001453 | -0.679146276 | 0.022451016 |
| ENSCLMG00005012221 | -0.679123518 | 0.017286974 |
| ENSCLMG00005013566 | -0.678743655 | 0.010992709 |
| ENSCLMG00005008841 | -0.677258712 | 0.002958692 |
| ENSCLMG00005005697 | -0.676710822 | 0.041878131 |
| ENSCLMG00005020523 | -0.67667234 | 0.048165768 |
| ENSCLMG00005006394 | -0.676601334 | 0.005800606 |
| ENSCLMG00005013600 | -0.676501143 | 0.008471341 |
| ENSCLMG00005000599 | -0.67616161 | 0.013639219 |
| ENSCLMG00005013858 | -0.675319526 | 0.000659497 |
| ENSCLMG00005013863 | -0.674547897 | 0.014358311 |
| ENSCLMG00005010715 | -0.673119212 | 0.004104622 |
| ENSCLMG00005015613 | -0.672325876 | 0.012570601 |
| ENSCLMG00005009304 | -0.671009779 | 0.004041089 |
| ENSCLMG00005018126 | -0.670523432 | 0.018670784 |
| ENSCLMG00005003604 | -0.670058382 | 0.020260511 |
| ENSCLMG00005017177 | -0.669967526 | 0.000877292 |
| ENSCLMG00005007232 | -0.66936971 | 0.001199939 |
| ENSCLMG00005009833 | -0.669074532 | 0.04467932 |
| ENSCLMG00005012391 | -0.668445918 | 0.011268764 |
| ENSCLMG00005020871 | -0.668317008 | 0.022253022 |
| ENSCLMG00005000407 | -0.668233899 | 0.005806365 |
| ENSCLMG00005014988 | -0.66816264 | 0.007112037 |
| ENSCLMG00005016423 | -0.665494425 | 0.013135399 |
| ENSCLMG00005018483 | -0.664400391 | 0.009234056 |
| ENSCLMG00005002387 | -0.664097689 | 0.007753474 |
| ENSCLMG00005018318 | -0.663847502 | 0.002078825 |
| ENSCLMG00005022982 | -0.662193457 | 0.002457045 |
| ENSCLMG00005002583 | -0.661345686 | 0.018525086 |
| ENSCLMG00005018056 | -0.660113593 | 0.047809775 |
| ENSCLMG00005015102 | -0.6595663 | 0.012954978 |
| ENSCLMG00005009136 | -0.658656296 | 0.033757314 |
| ENSCLMG00005003287 | -0.657737259 | 0.013135399 |
| ENSCLMG00005017438 | -0.656952203 | 0.008761525 |
| ENSCLMG00005018854 | -0.656481138 | 0.001009979 |
| ENSCLMG00005019379 | -0.655788571 | 0.011312348 |
| ENSCLMG00005018619 | -0.654843472 | 0.019594341 |
| ENSCLMG00005016159 | -0.65229981 | 0.003001392 |
| ENSCLMG00005021872 | -0.651677744 | 0.018453145 |
| ENSCLMG00005005506 | -0.650897702 | 0.006926788 |
| ENSCLMG00005019171 | -0.649819988 | 0.016469705 |
| ENSCLMG00005019222 | -0.649156489 | 0.00227811 |
| ENSCLMG00005007974 | -0.649062658 | 0.03989403 |
| ENSCLMG00005016508 | -0.648008397 | 0.014197954 |
| ENSCLMG00005013353 | -0.64749695 | 0.010765126 |
| ENSCLMG00005002561 | -0.6468432 | 0.017255042 |
| ENSCLMG00005018581 | -0.645471657 | 0.014169293 |
| ENSCLMG00005012925 | -0.645074402 | 0.008786835 |
| ENSCLMG00005007586 | -0.645043214 | 0.014142103 |
| ENSCLMG00005021295 | -0.643475924 | 0.038636495 |
| ENSCLMG00005022701 | -0.642423152 | 0.048030482 |
| ENSCLMG00005020658 | -0.64236527 | 0.046926445 |
| ENSCLMG00005013414 | -0.642073953 | 0.033847065 |
| ENSCLMG00005017954 | -0.641531033 | 0.001717707 |
| ENSCLMG00005021500 | -0.641413369 | 0.029064905 |
| ENSCLMG00005013884 | -0.640787787 | 0.000728393 |
| ENSCLMG00005001775 | -0.639908068 | 0.008769539 |
| ENSCLMG00005009505 | -0.639312418 | 0.005719054 |
| ENSCLMG00005019976 | -0.639254353 | 0.028518208 |
| ENSCLMG00005010218 | -0.638099061 | 0.02096354 |
| ENSCLMG00005008207 | -0.637739129 | 0.038289182 |
| ENSCLMG00005003922 | -0.636811674 | 0.009623942 |
| ENSCLMG00005003130 | -0.636654107 | 0.003907933 |
| ENSCLMG00005022409 | -0.636125235 | 0.026182393 |
| ENSCLMG00005009977 | -0.63543585 | 0.01118974 |
| ENSCLMG00005020485 | -0.634990734 | 0.006637706 |
| ENSCLMG00005004011 | -0.633137936 | 0.020023639 |
| ENSCLMG00005002402 | -0.633080667 | 0.013966149 |
| ENSCLMG00005012407 | -0.632233748 | 0.002530446 |
| ENSCLMG00005010916 | -0.630944095 | 0.017140042 |
| ENSCLMG00005013660 | -0.630756608 | 0.019830676 |
| ENSCLMG00005022307 | -0.630623579 | 0.00344875 |
| ENSCLMG00005000523 | -0.630186991 | 0.004144058 |
| ENSCLMG00005022198 | -0.629249159 | 0.002493479 |
| ENSCLMG00005022605 | -0.629129917 | 0.031425659 |
| ENSCLMG00005006192 | -0.628621247 | 0.015108141 |
| ENSCLMG00005014742 | -0.628582242 | 0.004457362 |
| ENSCLMG00005007685 | -0.627396233 | 0.006683194 |
| ENSCLMG00005012182 | -0.627258276 | 0.021456834 |
| ENSCLMG00005008486 | -0.626300359 | 0.011281072 |
| ENSCLMG00005009578 | -0.626080341 | 0.015805174 |
| ENSCLMG00005005155 | -0.625672115 | 0.004007699 |
| ENSCLMG00005015076 | -0.624679813 | 0.029855365 |
| ENSCLMG00005011724 | -0.622360624 | 0.007909595 |
| ENSCLMG00005019431 | -0.621958471 | 0.016440184 |
| ENSCLMG00005002098 | -0.620396825 | 0.04356795 |
| ENSCLMG00005009318 | -0.619140088 | 0.007716887 |
| ENSCLMG00005006431 | -0.618386706 | 0.030058965 |
| ENSCLMG00005002364 | -0.617741282 | 0.001144175 |
| ENSCLMG00005012799 | -0.614690767 | 0.038800618 |
| ENSCLMG00005001361 | -0.613340749 | 0.047434674 |
| ENSCLMG00005012522 | -0.612558678 | 0.046133762 |
| ENSCLMG00005016661 | -0.612157829 | 0.043554958 |
| ENSCLMG00005019192 | -0.611917333 | 0.005895799 |
| ENSCLMG00005009233 | -0.61159262 | 0.018449175 |
| ENSCLMG00005001849 | -0.611488862 | 0.030354858 |
| ENSCLMG00005008899 | -0.611354509 | 0.026821742 |
| ENSCLMG00005011732 | -0.610660059 | 0.044562541 |
| ENSCLMG00005015351 | -0.610440386 | 0.020159383 |
| ENSCLMG00005015659 | -0.61037125 | 0.014174972 |
| ENSCLMG00005020742 | -0.610370201 | 0.045467749 |
| ENSCLMG00005004273 | -0.609552933 | 0.030745822 |
| ENSCLMG00005019012 | -0.608882385 | 0.000727456 |
| ENSCLMG00005019318 | -0.608539322 | 0.006656882 |
| ENSCLMG00005019661 | -0.607351126 | 0.005902518 |
| ENSCLMG00005010340 | -0.607007905 | 0.030193988 |
| ENSCLMG00005022649 | -0.606868057 | 0.038754307 |
| ENSCLMG00005005943 | -0.606736525 | 0.03901191 |
| ENSCLMG00005013287 | -0.605944957 | 0.025739829 |
| ENSCLMG00005014853 | -0.605487146 | 0.04356795 |
| ENSCLMG00005005602 | -0.603777274 | 0.003806883 |
| ENSCLMG00005017352 | -0.603451616 | 0.02413407 |
| ENSCLMG00005004927 | -0.602872853 | 0.014233588 |
| ENSCLMG00005001741 | -0.602749045 | 0.033969786 |
| ENSCLMG00005007817 | -0.602368525 | 0.024708436 |
| ENSCLMG00005007617 | -0.602359849 | 0.012106537 |
| ENSCLMG00005019758 | -0.602329981 | 0.004081415 |
| ENSCLMG00005012524 | -0.602031367 | 0.004472957 |
| ENSCLMG00005019017 | -0.601502925 | 0.025739829 |
| ENSCLMG00005020520 | -0.600540319 | 0.023189173 |
| ENSCLMG00005009533 | -0.599564447 | 0.013574 |
| ENSCLMG00005005787 | -0.599277921 | 0.036473916 |
| ENSCLMG00005015821 | -0.599114002 | 0.011503734 |
| ENSCLMG00005012497 | -0.598669129 | 0.012355119 |
| ENSCLMG00005006811 | -0.598582999 | 0.003221133 |
| ENSCLMG00005006716 | -0.598543654 | 0.046100172 |
| ENSCLMG00005013883 | -0.597162078 | 0.011373451 |
| ENSCLMG00005016589 | -0.596610174 | 0.010623302 |
| ENSCLMG00005002519 | -0.595789861 | 0.013966149 |
| ENSCLMG00005006725 | -0.595242616 | 0.025290207 |
| ENSCLMG00005009283 | -0.595044057 | 0.012810381 |
| ENSCLMG00005023089 | -0.593831666 | 0.02413407 |
| ENSCLMG00005004794 | -0.59367306 | 0.009525508 |
| ENSCLMG00005020617 | -0.593468586 | 0.046786667 |
| ENSCLMG00005019289 | -0.592506282 | 0.049189177 |
| ENSCLMG00005008728 | -0.592284842 | 0.041612165 |
| ENSCLMG00005000484 | -0.591859142 | 0.03196652 |
| ENSCLMG00005012011 | -0.591297608 | 0.003603635 |
| ENSCLMG00005008280 | -0.591172738 | 0.022308741 |
| ENSCLMG00005008606 | -0.591162045 | 0.019875807 |
| ENSCLMG00005018709 | -0.590966994 | 0.008723097 |
| ENSCLMG00005021356 | -0.590873576 | 0.015003318 |
| ENSCLMG00005001180 | -0.59071141 | 0.02329227 |
| ENSCLMG00005020076 | -0.590523874 | 0.009848744 |
| ENSCLMG00005019672 | -0.589388764 | 0.04898499 |
| ENSCLMG00005009854 | -0.589298155 | 0.008397905 |
| ENSCLMG00005014973 | -0.588105466 | 0.020921819 |
| ENSCLMG00005015371 | -0.587776435 | 0.003662914 |
| ENSCLMG00005016142 | -0.587121629 | 0.012954978 |
| ENSCLMG00005011049 | -0.587020252 | 0.041874189 |
| ENSCLMG00005022490 | -0.586735602 | 0.011968377 |
| ENSCLMG00005014295 | -0.586733882 | 0.036859823 |
| ENSCLMG00005000726 | -0.585464369 | 0.003574784 |
| ENSCLMG00005007796 | -0.58368431 | 0.032654376 |
| ENSCLMG00005016400 | -0.583577408 | 0.013355535 |
| ENSCLMG00005002717 | -0.581958593 | 0.016910759 |
| ENSCLMG00005005600 | -0.581071277 | 0.016661315 |
| ENSCLMG00005022737 | -0.580907251 | 0.024374976 |
| ENSCLMG00005012095 | -0.58065774 | 0.042526179 |
| ENSCLMG00005011025 | -0.579730599 | 0.046242287 |
| ENSCLMG00005011156 | -0.579038124 | 0.008576523 |
| ENSCLMG00005006966 | -0.577606545 | 0.041149203 |
| ENSCLMG00005009901 | -0.577405386 | 0.0034298 |
| ENSCLMG00005006580 | -0.577168648 | 0.018408493 |
| ENSCLMG00005010321 | -0.577114781 | 0.013966149 |
| ENSCLMG00005000041 | -0.57577327 | 0.008845346 |
| ENSCLMG00005009917 | -0.575293583 | 0.030193988 |
| ENSCLMG00005002677 | -0.575147519 | 0.032654376 |
| ENSCLMG00005013338 | -0.574509315 | 0.034583015 |
| ENSCLMG00005008203 | -0.573720987 | 0.013961828 |
| ENSCLMG00005014532 | -0.573499825 | 0.014097013 |
| ENSCLMG00005004845 | -0.572018542 | 0.032928638 |
| ENSCLMG00005016888 | -0.571271795 | 0.010518299 |
| ENSCLMG00005021381 | -0.570747074 | 0.043353582 |
| ENSCLMG00005021522 | -0.569476034 | 0.031510066 |
| ENSCLMG00005002042 | -0.567972164 | 0.003940819 |
| ENSCLMG00005006320 | -0.563471071 | 0.009553438 |
| ENSCLMG00005000336 | -0.56211924 | 0.031916061 |
| ENSCLMG00005019790 | -0.558769071 | 0.004680015 |
| ENSCLMG00005003729 | -0.558718648 | 0.041952211 |
| ENSCLMG00005020664 | -0.557687393 | 0.013627633 |
| ENSCLMG00005002053 | -0.55702312 | 0.027855509 |
| ENSCLMG00005015760 | -0.556980009 | 0.041700901 |
| ENSCLMG00005019769 | -0.55613941 | 0.029819037 |
| ENSCLMG00005019303 | -0.554531979 | 0.021456834 |
| ENSCLMG00005009640 | -0.553487146 | 0.022003128 |
| ENSCLMG00005021014 | -0.553243081 | 0.012943571 |
| ENSCLMG00005019549 | -0.552977964 | 0.015899435 |
| ENSCLMG00005013407 | -0.552332079 | 0.020376024 |
| ENSCLMG00005014201 | -0.551875775 | 0.038514574 |
| ENSCLMG00005019019 | -0.550311894 | 0.006685453 |
| ENSCLMG00005000607 | -0.549870258 | 0.033872615 |
| ENSCLMG00005012066 | -0.548728734 | 0.015822328 |
| ENSCLMG00005003598 | -0.548719537 | 0.025035806 |
| ENSCLMG00005003094 | -0.545675182 | 0.005969012 |
| ENSCLMG00005022526 | -0.544785504 | 0.032876392 |
| ENSCLMG00005017836 | -0.544201056 | 0.037505895 |
| ENSCLMG00005013152 | -0.543496787 | 0.033872615 |
| ENSCLMG00005017774 | -0.541778029 | 0.039931551 |
| ENSCLMG00005022714 | -0.540655212 | 0.041670135 |
| ENSCLMG00005014940 | -0.539549895 | 0.040586156 |
| ENSCLMG00005006189 | -0.538958923 | 0.014320552 |
| ENSCLMG00005010950 | -0.537908901 | 0.034682441 |
| ENSCLMG00005003270 | -0.536509533 | 0.011599912 |
| ENSCLMG00005017505 | -0.536348261 | 0.034031314 |
| ENSCLMG00005021409 | -0.535421221 | 0.007054537 |
| ENSCLMG00005002085 | -0.5351456 | 0.013024977 |
| ENSCLMG00005009135 | -0.534351553 | 0.033521442 |
| ENSCLMG00005003027 | -0.53324934 | 0.025587373 |
| ENSCLMG00005020016 | -0.531243376 | 0.016596657 |
| ENSCLMG00005006090 | -0.529926928 | 0.007774663 |
| ENSCLMG00005005878 | -0.529841708 | 0.019690186 |
| ENSCLMG00005006418 | -0.529754904 | 0.049908308 |
| ENSCLMG00005010631 | -0.52868187 | 0.011705132 |
| ENSCLMG00005001273 | -0.527297044 | 0.015461254 |
| ENSCLMG00005019603 | -0.527039139 | 0.027646682 |
| ENSCLMG00005015237 | -0.527015703 | 0.048272094 |
| ENSCLMG00005000923 | -0.526261966 | 0.04528917 |
| ENSCLMG00005009030 | -0.525678077 | 0.018832846 |
| ENSCLMG00005004790 | -0.52521381 | 0.023584241 |
| ENSCLMG00005014561 | -0.524620836 | 0.031075621 |
| ENSCLMG00005009281 | -0.523695932 | 0.027929985 |
| ENSCLMG00005006939 | -0.522946252 | 0.018905969 |
| ENSCLMG00005016066 | -0.522351397 | 0.005085979 |
| ENSCLMG00005018449 | -0.520655286 | 0.023580985 |
| ENSCLMG00005009315 | -0.52002119 | 0.021179836 |
| ENSCLMG00005002142 | -0.517382952 | 0.037269027 |
| ENSCLMG00005015532 | -0.516856493 | 0.04467932 |
| ENSCLMG00005010560 | -0.516717177 | 0.041465539 |
| ENSCLMG00005006221 | -0.515759361 | 0.049277441 |
| ENSCLMG00005017845 | -0.514558718 | 0.046911509 |
| ENSCLMG00005001964 | -0.511876745 | 0.012810381 |
| ENSCLMG00005021519 | -0.511283911 | 0.03901191 |
| ENSCLMG00005023013 | -0.510746732 | 0.025483779 |
| ENSCLMG00005004124 | -0.510276303 | 0.010415597 |
| ENSCLMG00005011397 | -0.508741619 | 0.03232208 |
| ENSCLMG00005015948 | -0.508709159 | 0.026062942 |
| ENSCLMG00005015390 | -0.507678671 | 0.033273237 |
| ENSCLMG00005016782 | -0.506845175 | 0.013690105 |
| ENSCLMG00005010941 | -0.505771836 | 0.020237161 |
| ENSCLMG00005010019 | -0.504815086 | 0.03264202 |
| ENSCLMG00005016880 | -0.502180813 | 0.031411052 |
| ENSCLMG00005008274 | -0.500534332 | 0.025587373 |
| ENSCLMG00005004062 | -0.49932102 | 0.023580985 |
| ENSCLMG00005004909 | -0.498041136 | 0.023062055 |
| ENSCLMG00005008380 | -0.497225842 | 0.024613662 |
| ENSCLMG00005005458 | -0.496908242 | 0.02606272 |
| ENSCLMG00005021957 | -0.496233354 | 0.017228155 |
| ENSCLMG00005007420 | -0.496148848 | 0.024567032 |
| ENSCLMG00005020167 | -0.496126014 | 0.010818403 |
| ENSCLMG00005019534 | -0.49577075 | 0.025687879 |
| ENSCLMG00005013577 | -0.49512468 | 0.021060299 |
| ENSCLMG00005007845 | -0.493799652 | 0.024396114 |
| ENSCLMG00005019258 | -0.493522897 | 0.023584241 |
| ENSCLMG00005006776 | -0.493115945 | 0.04467932 |
| ENSCLMG00005011007 | -0.491366268 | 0.01097495 |
| ENSCLMG00005022202 | -0.490336023 | 0.018525086 |
| ENSCLMG00005016616 | -0.489461736 | 0.049651952 |
| ENSCLMG00005015192 | -0.487537884 | 0.010818403 |
| ENSCLMG00005014213 | -0.487429568 | 0.028105755 |
| ENSCLMG00005011989 | -0.487228697 | 0.03288395 |
| ENSCLMG00005020417 | -0.485616226 | 0.034760015 |
| ENSCLMG00005014270 | -0.48409533 | 0.047175469 |
| ENSCLMG00005004163 | -0.478226348 | 0.027721766 |
| ENSCLMG00005010136 | -0.477362174 | 0.04356795 |
| ENSCLMG00005006304 | -0.475269302 | 0.039779026 |
| ENSCLMG00005020305 | -0.474704453 | 0.023556 |
| ENSCLMG00005005209 | -0.473448343 | 0.014217891 |
| ENSCLMG00005005680 | -0.473397986 | 0.041700901 |
| ENSCLMG00005021315 | -0.468666082 | 0.042073022 |
| ENSCLMG00005010067 | -0.459015617 | 0.026598023 |
| ENSCLMG00005018948 | -0.457461176 | 0.04396763 |
| ENSCLMG00005020981 | -0.449686701 | 0.032468627 |
| ENSCLMG00005011992 | -0.448877185 | 0.040586156 |
| ENSCLMG00005011816 | -0.447523104 | 0.038098817 |
| ENSCLMG00005005860 | -0.432375926 | 0.031414774 |
| ENSCLMG00005018532 | -0.430658251 | 0.040707017 |
| ENSCLMG00005007633 | -0.430657056 | 0.028429423 |
| ENSCLMG00005020956 | -0.425972415 | 0.049280313 |
| ENSCLMG00005007452 | -0.415385067 | 0.049568816 |
| ENSCLMG00005010522 | -0.397677756 | 0.04537054 |
| ENSCLMG00005013836 | 0.390586239 | 0.046577093 |
| ENSCLMG00005018390 | 0.403187654 | 0.047521741 |
| ENSCLMG00005001846 | 0.406026351 | 0.043212962 |
| ENSCLMG00005006183 | 0.407124772 | 0.048209271 |
| ENSCLMG00005010004 | 0.421571319 | 0.0438349 |
| ENSCLMG00005018686 | 0.434693596 | 0.043404118 |
| ENSCLMG00005019606 | 0.43519984 | 0.04537054 |
| ENSCLMG00005016548 | 0.437155437 | 0.042225051 |
| ENSCLMG00005021325 | 0.437593753 | 0.033202214 |
| ENSCLMG00005012149 | 0.440113398 | 0.039517663 |
| ENSCLMG00005018719 | 0.444746486 | 0.019758739 |
| ENSCLMG00005007507 | 0.445610382 | 0.04002961 |
| ENSCLMG00005002236 | 0.453941622 | 0.031595845 |
| ENSCLMG00005007459 | 0.455609512 | 0.042073022 |
| ENSCLMG00005020570 | 0.45709865 | 0.012301216 |
| ENSCLMG00005011423 | 0.460829022 | 0.033417703 |
| ENSCLMG00005015153 | 0.462312278 | 0.031575983 |
| ENSCLMG00005009720 | 0.464017678 | 0.019646526 |
| ENSCLMG00005010548 | 0.464446718 | 0.028098657 |
| ENSCLMG00005005357 | 0.466630342 | 0.042797277 |
| ENSCLMG00005012190 | 0.466997623 | 0.033273237 |
| ENSCLMG00005000400 | 0.467649456 | 0.030745822 |
| ENSCLMG00005009609 | 0.472592163 | 0.04467932 |
| ENSCLMG00005011507 | 0.47355758 | 0.04467932 |
| ENSCLMG00005006124 | 0.4739716 | 0.015822328 |
| ENSCLMG00005008249 | 0.474290468 | 0.031575983 |
| ENSCLMG00005021940 | 0.474816749 | 0.030745822 |
| ENSCLMG00005011113 | 0.477479575 | 0.038936228 |
| ENSCLMG00005022756 | 0.477595626 | 0.025442843 |
| ENSCLMG00005017628 | 0.477839014 | 0.024889152 |
| ENSCLMG00005004881 | 0.479991822 | 0.036283804 |
| ENSCLMG00005006572 | 0.480005033 | 0.017110385 |
| ENSCLMG00005006483 | 0.4807888 | 0.021012433 |
| ENSCLMG00005018188 | 0.481623245 | 0.013690679 |
| ENSCLMG00005006545 | 0.481692277 | 0.033838222 |
| ENSCLMG00005021499 | 0.483689065 | 0.045827201 |
| ENSCLMG00005022394 | 0.484080771 | 0.042073022 |
| ENSCLMG00005002375 | 0.486171926 | 0.009635003 |
| ENSCLMG00005013608 | 0.486728325 | 0.017286974 |
| ENSCLMG00005014130 | 0.48784236 | 0.041759363 |
| ENSCLMG00005020897 | 0.488331173 | 0.031573541 |
| ENSCLMG00005003234 | 0.48888582 | 0.039688744 |
| ENSCLMG00005003831 | 0.489305779 | 0.019653652 |
| ENSCLMG00005012765 | 0.490122932 | 0.032654376 |
| ENSCLMG00005014180 | 0.490328831 | 0.035255184 |
| ENSCLMG00005002786 | 0.491299781 | 0.013024977 |
| ENSCLMG00005005983 | 0.491595782 | 0.042073022 |
| ENSCLMG00005018254 | 0.493856282 | 0.038682923 |
| ENSCLMG00005010006 | 0.494985867 | 0.048909372 |
| ENSCLMG00005009111 | 0.495982391 | 0.027156982 |
| ENSCLMG00005012184 | 0.496033655 | 0.015328953 |
| ENSCLMG00005000213 | 0.496407169 | 0.029063097 |
| ENSCLMG00005001706 | 0.497578297 | 0.030614333 |
| ENSCLMG00005012186 | 0.498231661 | 0.039636664 |
| ENSCLMG00005019401 | 0.499520744 | 0.029862605 |
| ENSCLMG00005022598 | 0.501572628 | 0.041149203 |
| ENSCLMG00005006451 | 0.50217356 | 0.03913388 |
| ENSCLMG00005006434 | 0.504440304 | 0.049067696 |
| ENSCLMG00005007794 | 0.504994795 | 0.007494133 |
| ENSCLMG00005016095 | 0.505312652 | 0.016669708 |
| ENSCLMG00005022382 | 0.505600361 | 0.04368901 |
| ENSCLMG00005000314 | 0.50780648 | 0.016491662 |
| ENSCLMG00005001155 | 0.508026332 | 0.038681391 |
| ENSCLMG00005000554 | 0.508666352 | 0.045055339 |
| ENSCLMG00005008212 | 0.508972592 | 0.00584669 |
| ENSCLMG00005013908 | 0.50926685 | 0.015477207 |
| ENSCLMG00005017163 | 0.510349897 | 0.014639738 |
| ENSCLMG00005020153 | 0.511709049 | 0.02766261 |
| ENSCLMG00005003849 | 0.512034848 | 0.010765126 |
| ENSCLMG00005010216 | 0.51247002 | 0.013917787 |
| ENSCLMG00005022501 | 0.512838801 | 0.007509148 |
| ENSCLMG00005003848 | 0.513695697 | 0.026349568 |
| ENSCLMG00005010766 | 0.51507061 | 0.037244892 |
| ENSCLMG00005002712 | 0.515073591 | 0.036417677 |
| ENSCLMG00005004155 | 0.516270404 | 0.022708114 |
| ENSCLMG00005018278 | 0.518019803 | 0.019622743 |
| ENSCLMG00005016448 | 0.519374808 | 0.014305775 |
| ENSCLMG00005017504 | 0.519790516 | 0.047220523 |
| ENSCLMG00005016851 | 0.521445657 | 0.038700667 |
| ENSCLMG00005017568 | 0.521510676 | 0.030143088 |
| ENSCLMG00005009064 | 0.521688536 | 0.041878131 |
| ENSCLMG00005017229 | 0.523083814 | 0.035836755 |
| ENSCLMG00005022554 | 0.524211469 | 0.045126126 |
| ENSCLMG00005000939 | 0.527714326 | 0.047010307 |
| ENSCLMG00005011909 | 0.528721821 | 0.015445534 |
| ENSCLMG00005016724 | 0.529147514 | 0.02620533 |
| ENSCLMG00005010531 | 0.529929685 | 0.02600899 |
| ENSCLMG00005022774 | 0.530435481 | 0.030614333 |
| ENSCLMG00005016857 | 0.531248513 | 0.012869582 |
| ENSCLMG00005000735 | 0.531692869 | 0.033202214 |
| ENSCLMG00005018814 | 0.533959398 | 0.016186595 |
| ENSCLMG00005014986 | 0.534719334 | 0.04878416 |
| ENSCLMG00005002134 | 0.53544508 | 0.048165768 |
| ENSCLMG00005006813 | 0.535558467 | 0.00347471 |
| ENSCLMG00005014720 | 0.535813273 | 0.00867461 |
| ENSCLMG00005005763 | 0.537821713 | 0.015003318 |
| ENSCLMG00005000279 | 0.537853259 | 0.037496742 |
| ENSCLMG00005003170 | 0.538152089 | 0.048128133 |
| ENSCLMG00005015196 | 0.538570606 | 0.009159771 |
| ENSCLMG00005011271 | 0.53904863 | 0.003395802 |
| ENSCLMG00005021374 | 0.539845987 | 0.028426358 |
| ENSCLMG00005007549 | 0.540355103 | 0.040061774 |
| ENSCLMG00005004166 | 0.540546803 | 0.01761675 |
| ENSCLMG00005021165 | 0.541867277 | 0.032654376 |
| ENSCLMG00005014212 | 0.542201034 | 0.005714692 |
| ENSCLMG00005018010 | 0.542629185 | 0.005175047 |
| ENSCLMG00005016655 | 0.543340092 | 0.003567591 |
| ENSCLMG00005000056 | 0.543857106 | 0.004007699 |
| ENSCLMG00005013611 | 0.544024077 | 0.023325574 |
| ENSCLMG00005006170 | 0.546055735 | 0.0438349 |
| ENSCLMG00005022622 | 0.547582832 | 0.028966646 |
| ENSCLMG00005011960 | 0.549129356 | 0.047220523 |
| ENSCLMG00005002573 | 0.551247327 | 0.008197977 |
| ENSCLMG00005022319 | 0.551507626 | 0.016795961 |
| ENSCLMG00005008822 | 0.5521017 | 0.027017809 |
| ENSCLMG00005008417 | 0.55241105 | 0.034760015 |
| ENSCLMG00005021047 | 0.553090275 | 0.006828288 |
| ENSCLMG00005012293 | 0.554392363 | 0.024385974 |
| ENSCLMG00005001068 | 0.554527513 | 0.013347545 |
| ENSCLMG00005018425 | 0.555797895 | 0.025106001 |
| ENSCLMG00005022432 | 0.556490943 | 0.042625663 |
| ENSCLMG00005006171 | 0.556592638 | 0.038560119 |
| ENSCLMG00005022615 | 0.55698669 | 0.034346875 |
| ENSCLMG00005009317 | 0.557866398 | 0.002237119 |
| ENSCLMG00005002091 | 0.558249157 | 0.007913737 |
| ENSCLMG00005001141 | 0.558772322 | 0.035289617 |
| ENSCLMG00005001484 | 0.559557187 | 0.02701094 |
| ENSCLMG00005020936 | 0.559766795 | 0.035843813 |
| ENSCLMG00005016642 | 0.560228409 | 0.022623133 |
| ENSCLMG00005002296 | 0.561057131 | 0.046133762 |
| ENSCLMG00005002832 | 0.561510027 | 0.021173165 |
| ENSCLMG00005018678 | 0.561520356 | 0.004174036 |
| ENSCLMG00005006690 | 0.561569024 | 0.018914685 |
| ENSCLMG00005017699 | 0.561601978 | 0.024224284 |
| ENSCLMG00005002968 | 0.56231413 | 0.036670763 |
| ENSCLMG00005007381 | 0.562537066 | 0.020185066 |
| ENSCLMG00005010465 | 0.562596723 | 0.001369141 |
| ENSCLMG00005009104 | 0.56305653 | 0.036805973 |
| ENSCLMG00005006543 | 0.564850082 | 0.026526623 |
| ENSCLMG00005014203 | 0.565076532 | 0.049465632 |
| ENSCLMG00005020659 | 0.565264414 | 0.040520513 |
| ENSCLMG00005008192 | 0.565714513 | 0.040662085 |
| ENSCLMG00005013676 | 0.566129174 | 0.011614112 |
| ENSCLMG00005014398 | 0.566300982 | 0.04253107 |
| ENSCLMG00005021712 | 0.566399442 | 0.002845585 |
| ENSCLMG00005006742 | 0.56779371 | 0.044120603 |
| ENSCLMG00005013126 | 0.567973999 | 0.008222199 |
| ENSCLMG00005019391 | 0.568735987 | 0.004394569 |
| ENSCLMG00005015325 | 0.56994254 | 0.042073022 |
| ENSCLMG00005018382 | 0.570064493 | 0.023871425 |
| ENSCLMG00005015536 | 0.570505255 | 0.036682179 |
| ENSCLMG00005005029 | 0.571777862 | 0.014097013 |
| ENSCLMG00005010739 | 0.572035857 | 0.042191041 |
| ENSCLMG00005007910 | 0.572349415 | 0.015983679 |
| ENSCLMG00005010097 | 0.57246883 | 0.020257844 |
| ENSCLMG00005009452 | 0.572862672 | 0.009848744 |
| ENSCLMG00005005290 | 0.573324401 | 0.02014252 |
| ENSCLMG00005021095 | 0.574159772 | 0.04868373 |
| ENSCLMG00005019854 | 0.574169459 | 0.028098657 |
| ENSCLMG00005013227 | 0.576116966 | 0.014163024 |
| ENSCLMG00005019607 | 0.576478005 | 0.034954399 |
| ENSCLMG00005014493 | 0.577222449 | 0.011503734 |
| ENSCLMG00005002946 | 0.577491606 | 0.032083038 |
| ENSCLMG00005000662 | 0.577586984 | 0.033173834 |
| ENSCLMG00005002701 | 0.577661377 | 0.016882653 |
| ENSCLMG00005002292 | 0.577880896 | 0.049067696 |
| ENSCLMG00005008211 | 0.580350696 | 0.00978678 |
| ENSCLMG00005017103 | 0.580637262 | 0.015983679 |
| ENSCLMG00005013597 | 0.581144261 | 0.012263675 |
| ENSCLMG00005001144 | 0.581321394 | 0.031346389 |
| ENSCLMG00005005439 | 0.581566608 | 0.025397969 |
| ENSCLMG00005012948 | 0.582317476 | 0.049057244 |
| ENSCLMG00005006095 | 0.582794057 | 0.012775965 |
| ENSCLMG00005020248 | 0.583716823 | 0.005875956 |
| ENSCLMG00005019845 | 0.583917749 | 0.008010893 |
| ENSCLMG00005001720 | 0.584610649 | 0.0438349 |
| ENSCLMG00005006666 | 0.585189676 | 0.001955151 |
| ENSCLMG00005016016 | 0.586165287 | 0.001784601 |
| ENSCLMG00005005909 | 0.586627783 | 0.003457875 |
| ENSCLMG00005003509 | 0.587580304 | 0.020376024 |
| ENSCLMG00005016510 | 0.587801471 | 0.004628802 |
| ENSCLMG00005010788 | 0.588159307 | 0.027855509 |
| ENSCLMG00005007071 | 0.588787976 | 0.049952027 |
| ENSCLMG00005000569 | 0.590094481 | 0.000906391 |
| ENSCLMG00005017043 | 0.590155003 | 0.007848704 |
| ENSCLMG00005017868 | 0.591504799 | 0.00818592 |
| ENSCLMG00005018051 | 0.591887273 | 0.003355454 |
| ENSCLMG00005002466 | 0.592844307 | 0.00211615 |
| ENSCLMG00005017183 | 0.593265276 | 0.012592769 |
| ENSCLMG00005016960 | 0.593827393 | 0.025982737 |
| ENSCLMG00005015546 | 0.594052379 | 0.013966149 |
| ENSCLMG00005021032 | 0.594406868 | 0.008401291 |
| ENSCLMG00005004936 | 0.594573613 | 0.028429423 |
| ENSCLMG00005003127 | 0.595550942 | 0.028169183 |
| ENSCLMG00005003515 | 0.596043382 | 0.020068902 |
| ENSCLMG00005007044 | 0.596236702 | 0.022872338 |
| ENSCLMG00005008157 | 0.596635976 | 0.002184001 |
| ENSCLMG00005014429 | 0.597944548 | 0.034604578 |
| ENSCLMG00005014423 | 0.598478639 | 0.016669708 |
| ENSCLMG00005007815 | 0.599368708 | 0.004742371 |
| ENSCLMG00005015124 | 0.599415973 | 0.020753898 |
| ENSCLMG00005004818 | 0.599885148 | 0.007314635 |
| ENSCLMG00005021229 | 0.600037164 | 0.002251016 |
| ENSCLMG00005019032 | 0.600296113 | 0.002550077 |
| ENSCLMG00005009610 | 0.601304611 | 0.002796939 |
| ENSCLMG00005017303 | 0.602252021 | 0.036719173 |
| ENSCLMG00005006667 | 0.602916998 | 0.015462524 |
| ENSCLMG00005005972 | 0.603118567 | 0.028576645 |
| ENSCLMG00005012849 | 0.603887353 | 0.029224556 |
| ENSCLMG00005004173 | 0.604178574 | 0.020880183 |
| ENSCLMG00005000528 | 0.604740315 | 0.005523137 |
| ENSCLMG00005007996 | 0.605157523 | 0.012497709 |
| ENSCLMG00005023147 | 0.60530451 | 0.028361065 |
| ENSCLMG00005021109 | 0.606073039 | 0.008949649 |
| ENSCLMG00005007561 | 0.60646322 | 0.027767533 |
| ENSCLMG00005000835 | 0.606495193 | 0.041749641 |
| ENSCLMG00005021818 | 0.606698552 | 0.012598262 |
| ENSCLMG00005020880 | 0.607712047 | 0.00094699 |
| ENSCLMG00005017301 | 0.608396513 | 0.011705132 |
| ENSCLMG00005013557 | 0.609084241 | 0.012452782 |
| ENSCLMG00005004168 | 0.609570873 | 0.005103422 |
| ENSCLMG00005017711 | 0.609623562 | 0.012869582 |
| ENSCLMG00005005119 | 0.610861849 | 0.003221133 |
| ENSCLMG00005020431 | 0.611363216 | 0.014405412 |
| ENSCLMG00005005168 | 0.611536552 | 0.049841619 |
| ENSCLMG00005012165 | 0.612975171 | 0.027662285 |
| ENSCLMG00005015813 | 0.613214234 | 0.004560815 |
| ENSCLMG00005020933 | 0.614245399 | 0.005131664 |
| ENSCLMG00005015080 | 0.615902464 | 0.001641076 |
| ENSCLMG00005022816 | 0.617829292 | 0.026831941 |
| ENSCLMG00005003995 | 0.619157635 | 0.000805613 |
| ENSCLMG00005001997 | 0.619582691 | 0.020824492 |
| ENSCLMG00005000008 | 0.620508791 | 0.015717155 |
| ENSCLMG00005002093 | 0.62175321 | 0.020174362 |
| ENSCLMG00005003311 | 0.622226737 | 0.023189173 |
| ENSCLMG00005009107 | 0.622733399 | 0.007133863 |
| ENSCLMG00005014697 | 0.623033162 | 0.026221838 |
| ENSCLMG00005010440 | 0.623254608 | 0.042167186 |
| ENSCLMG00005004145 | 0.624602686 | 0.009156861 |
| ENSCLMG00005018379 | 0.624884978 | 0.015445534 |
| ENSCLMG00005020732 | 0.625800916 | 0.025646171 |
| ENSCLMG00005018238 | 0.626690963 | 0.007586893 |
| ENSCLMG00005000529 | 0.627124862 | 0.020661945 |
| ENSCLMG00005021877 | 0.627173692 | 0.016700843 |
| ENSCLMG00005001116 | 0.627986363 | 0.002400079 |
| ENSCLMG00005012602 | 0.628391706 | 0.005277222 |
| ENSCLMG00005005069 | 0.628481396 | 0.018348582 |
| ENSCLMG00005008748 | 0.628501072 | 0.028813056 |
| ENSCLMG00005022387 | 0.628702074 | 0.004832378 |
| ENSCLMG00005000637 | 0.629211561 | 0.034165208 |
| ENSCLMG00005009042 | 0.630469566 | 0.023123726 |
| ENSCLMG00005007123 | 0.630842135 | 0.014197954 |
| ENSCLMG00005012917 | 0.631791198 | 0.021950188 |
| ENSCLMG00005018892 | 0.631943521 | 0.016061128 |
| ENSCLMG00005003001 | 0.632207413 | 0.001974515 |
| ENSCLMG00005003766 | 0.632220837 | 0.023977558 |
| ENSCLMG00005013866 | 0.634049247 | 0.041149203 |
| ENSCLMG00005010954 | 0.634256022 | 0.003671432 |
| ENSCLMG00005011295 | 0.634955858 | 0.01756296 |
| ENSCLMG00005008424 | 0.635257702 | 0.008876755 |
| ENSCLMG00005008758 | 0.635694282 | 0.034031314 |
| ENSCLMG00005022320 | 0.636024993 | 0.013239713 |
| ENSCLMG00005022915 | 0.639284072 | 0.028105755 |
| ENSCLMG00005014788 | 0.639535425 | 0.023493118 |
| ENSCLMG00005002359 | 0.640111759 | 0.01268528 |
| ENSCLMG00005019124 | 0.640681664 | 0.011705132 |
| ENSCLMG00005020053 | 0.644915134 | 0.045779089 |
| ENSCLMG00005018995 | 0.645388435 | 0.001296021 |
| ENSCLMG00005016203 | 0.645862523 | 0.000222738 |
| ENSCLMG00005018811 | 0.64658411 | 0.017255042 |
| ENSCLMG00005015258 | 0.646775855 | 0.009630641 |
| ENSCLMG00005002745 | 0.647149109 | 0.042136179 |
| ENSCLMG00005008957 | 0.647608941 | 0.00142373 |
| ENSCLMG00005014629 | 0.647985698 | 0.035081901 |
| ENSCLMG00005005584 | 0.648078433 | 0.027915094 |
| ENSCLMG00005000754 | 0.648153774 | 0.015993275 |
| ENSCLMG00005007335 | 0.648357194 | 0.012847264 |
| ENSCLMG00005002675 | 0.648449892 | 0.039300728 |
| ENSCLMG00005017426 | 0.648665721 | 0.000634687 |
| ENSCLMG00005016061 | 0.648942804 | 0.002422411 |
| ENSCLMG00005001564 | 0.648991583 | 0.040273046 |
| ENSCLMG00005012383 | 0.649829994 | 0.034460903 |
| ENSCLMG00005019917 | 0.650354091 | 0.00075235 |
| ENSCLMG00005012267 | 0.655976769 | 0.005947982 |
| ENSCLMG00005005686 | 0.656068237 | 0.040465877 |
| ENSCLMG00005001513 | 0.656580668 | 0.014639738 |
| ENSCLMG00005014727 | 0.657693706 | 0.002237722 |
| ENSCLMG00005013059 | 0.657901738 | 0.009593097 |
| ENSCLMG00005002279 | 0.65838444 | 0.023553011 |
| ENSCLMG00005002101 | 0.658903619 | 0.001252119 |
| ENSCLMG00005010853 | 0.659134085 | 0.018851452 |
| ENSCLMG00005002266 | 0.659168722 | 0.007585705 |
| ENSCLMG00005004000 | 0.659490177 | 0.022127507 |
| ENSCLMG00005022523 | 0.65978923 | 0.025245586 |
| ENSCLMG00005014542 | 0.659904462 | 0.009735763 |
| ENSCLMG00005017848 | 0.660124406 | 0.047521741 |
| ENSCLMG00005005189 | 0.660501684 | 0.000748384 |
| ENSCLMG00005008811 | 0.660560806 | 0.025091047 |
| ENSCLMG00005022468 | 0.660730157 | 0.010765126 |
| ENSCLMG00005005482 | 0.661247032 | 0.001504013 |
| ENSCLMG00005019235 | 0.661409793 | 0.009221134 |
| ENSCLMG00005005147 | 0.661624101 | 0.013024977 |
| ENSCLMG00005007711 | 0.662113096 | 0.010115159 |
| ENSCLMG00005008687 | 0.662597469 | 0.000919433 |
| ENSCLMG00005010731 | 0.662723279 | 0.004427322 |
| ENSCLMG00005020388 | 0.663633522 | 0.023580985 |
| ENSCLMG00005014439 | 0.66418448 | 0.000740378 |
| ENSCLMG00005015400 | 0.664866136 | 0.008344255 |
| ENSCLMG00005014631 | 0.665057624 | 0.036129483 |
| ENSCLMG00005002942 | 0.666474969 | 0.014038174 |
| ENSCLMG00005001991 | 0.667648501 | 0.008353139 |
| ENSCLMG00005022495 | 0.668441675 | 0.04090772 |
| ENSCLMG00005015333 | 0.669175671 | 0.000161918 |
| ENSCLMG00005022901 | 0.669406002 | 6.91E-05 |
| ENSCLMG00005009628 | 0.669407789 | 0.000399695 |
| ENSCLMG00005008738 | 0.670438839 | 0.042643731 |
| ENSCLMG00005006605 | 0.670878123 | 0.01210906 |
| ENSCLMG00005015523 | 0.671313003 | 0.035099496 |
| ENSCLMG00005017603 | 0.67174779 | 0.000108995 |
| ENSCLMG00005006781 | 0.672043108 | 0.040472109 |
| ENSCLMG00005018384 | 0.6731363 | 0.011822034 |
| ENSCLMG00005003830 | 0.673185629 | 0.001271784 |
| ENSCLMG00005000360 | 0.673874983 | 0.007304432 |
| ENSCLMG00005010580 | 0.674222673 | 0.005026051 |
| ENSCLMG00005018247 | 0.674338181 | 0.003420627 |
| ENSCLMG00005016389 | 0.67490093 | 0.022436016 |
| ENSCLMG00005007577 | 0.675651174 | 0.048314665 |
| ENSCLMG00005008824 | 0.675730463 | 0.002589123 |
| ENSCLMG00005010264 | 0.675806418 | 0.011823663 |
| ENSCLMG00005008924 | 0.677323653 | 0.00418801 |
| ENSCLMG00005008162 | 0.678128441 | 0.013966149 |
| ENSCLMG00005009444 | 0.678515597 | 0.002853319 |
| ENSCLMG00005021732 | 0.678967975 | 0.002273116 |
| ENSCLMG00005005358 | 0.67977368 | 0.027944384 |
| ENSCLMG00005008346 | 0.681029672 | 0.000440442 |
| ENSCLMG00005015769 | 0.681375412 | 0.003557558 |
| ENSCLMG00005011099 | 0.682986893 | 0.002311395 |
| ENSCLMG00005006134 | 0.683025565 | 0.001760941 |
| ENSCLMG00005007279 | 0.683074931 | 0.020936095 |
| ENSCLMG00005020901 | 0.683572687 | 0.032654376 |
| ENSCLMG00005003089 | 0.683730341 | 0.008043456 |
| ENSCLMG00005002096 | 0.68389072 | 0.001434034 |
| ENSCLMG00005014607 | 0.684096609 | 0.005066802 |
| ENSCLMG00005011707 | 0.684913715 | 0.008507429 |
| ENSCLMG00005020748 | 0.685139332 | 0.006983308 |
| ENSCLMG00005017456 | 0.686351168 | 0.006258249 |
| ENSCLMG00005011301 | 0.68675361 | 0.003350506 |
| ENSCLMG00005022101 | 0.687183111 | 0.047521741 |
| ENSCLMG00005019332 | 0.687636507 | 0.004519145 |
| ENSCLMG00005008562 | 0.688202176 | 0.003018125 |
| ENSCLMG00005013323 | 0.68844688 | 0.030923365 |
| ENSCLMG00005004089 | 0.690384874 | 0.004247548 |
| ENSCLMG00005010476 | 0.690913527 | 0.009905919 |
| ENSCLMG00005016771 | 0.691605956 | 0.017110385 |
| ENSCLMG00005017481 | 0.691724651 | 0.005085979 |
| ENSCLMG00005002357 | 0.691991974 | 0.046729811 |
| ENSCLMG00005004744 | 0.694549021 | 0.048030482 |
| ENSCLMG00005002201 | 0.695066697 | 0.016149246 |
| ENSCLMG00005015620 | 0.695474273 | 0.005443763 |
| ENSCLMG00005008695 | 0.695722942 | 0.016367549 |
| ENSCLMG00005019101 | 0.695870433 | 0.000244922 |
| ENSCLMG00005000667 | 0.696715384 | 0.03957188 |
| ENSCLMG00005022422 | 0.697280662 | 0.000583703 |
| ENSCLMG00005000320 | 0.698780109 | 0.011390969 |
| ENSCLMG00005007839 | 0.698859563 | 0.002672401 |
| ENSCLMG00005000772 | 0.699620015 | 0.00043231 |
| ENSCLMG00005002370 | 0.700604357 | 0.005277222 |
| ENSCLMG00005000972 | 0.701448926 | 0.044071255 |
| ENSCLMG00005022528 | 0.701930782 | 0.000973579 |
| ENSCLMG00005012365 | 0.702361001 | 0.028429423 |
| ENSCLMG00005017197 | 0.703692948 | 0.022003128 |
| ENSCLMG00005002587 | 0.706552007 | 0.04467932 |
| ENSCLMG00005000440 | 0.706646303 | 0.01093176 |
| ENSCLMG00005015618 | 0.707013411 | 0.00020655 |
| ENSCLMG00005013943 | 0.707970852 | 0.004832378 |
| ENSCLMG00005010121 | 0.709261928 | 0.023716977 |
| ENSCLMG00005022920 | 0.71016969 | 0.028658916 |
| ENSCLMG00005001701 | 0.711706108 | 0.033170207 |
| ENSCLMG00005009476 | 0.711774593 | 0.020574503 |
| ENSCLMG00005018759 | 0.711790778 | 0.007422668 |
| ENSCLMG00005010081 | 0.711998892 | 0.005995336 |
| ENSCLMG00005017742 | 0.712188224 | 7.80E-05 |
| ENSCLMG00005012295 | 0.714216408 | 0.020053888 |
| ENSCLMG00005004896 | 0.714634575 | 4.30E-05 |
| ENSCLMG00005019846 | 0.714664809 | 0.011766245 |
| ENSCLMG00005019467 | 0.715439352 | 9.53E-05 |
| ENSCLMG00005000586 | 0.715781557 | 0.007441607 |
| ENSCLMG00005022681 | 0.716096364 | 0.045816522 |
| ENSCLMG00005014812 | 0.717544184 | 0.000452082 |
| ENSCLMG00005002413 | 0.718750438 | 0.006617716 |
| ENSCLMG00005001575 | 0.719300203 | 0.002830204 |
| ENSCLMG00005008781 | 0.719681005 | 0.004007699 |
| ENSCLMG00005005959 | 0.719819629 | 0.02096354 |
| ENSCLMG00005016765 | 0.719936366 | 0.000846347 |
| ENSCLMG00005016444 | 0.720685943 | 0.024050173 |
| ENSCLMG00005011291 | 0.720912711 | 0.00026894 |
| ENSCLMG00005012510 | 0.721322922 | 0.027361976 |
| ENSCLMG00005005749 | 0.721493075 | 0.001060785 |
| ENSCLMG00005011537 | 0.721564629 | 0.010765126 |
| ENSCLMG00005007901 | 0.7231073 | 0.027855509 |
| ENSCLMG00005008975 | 0.723632119 | 0.000151634 |
| ENSCLMG00005002431 | 0.724725744 | 0.000305834 |
| ENSCLMG00005022600 | 0.724777187 | 0.040662085 |
| ENSCLMG00005019816 | 0.724819392 | 0.012950815 |
| ENSCLMG00005015157 | 0.724894898 | 0.00825242 |
| ENSCLMG00005013049 | 0.724911365 | 0.024050173 |
| ENSCLMG00005007128 | 0.725447878 | 0.015591482 |
| ENSCLMG00005008836 | 0.726462646 | 0.049444485 |
| ENSCLMG00005003585 | 0.726924315 | 0.008455342 |
| ENSCLMG00005001938 | 0.727192199 | 0.000610066 |
| ENSCLMG00005023016 | 0.727933244 | 0.006133971 |
| ENSCLMG00005002356 | 0.728649815 | 0.000133919 |
| ENSCLMG00005003537 | 0.728677224 | 0.009249594 |
| ENSCLMG00005017751 | 0.72905227 | 0.009623942 |
| ENSCLMG00005001230 | 0.730573557 | 0.016669708 |
| ENSCLMG00005005028 | 0.731550502 | 0.041612165 |
| ENSCLMG00005003264 | 0.731849203 | 0.001743128 |
| ENSCLMG00005001362 | 0.731968085 | 0.02845931 |
| ENSCLMG00005005495 | 0.732328255 | 0.006001735 |
| ENSCLMG00005005831 | 0.733716023 | 0.033847065 |
| ENSCLMG00005003958 | 0.733743478 | 0.048803277 |
| ENSCLMG00005011758 | 0.737039518 | 0.005085979 |
| ENSCLMG00005003876 | 0.737367559 | 0.000966412 |
| ENSCLMG00005020941 | 0.738390946 | 0.004472957 |
| ENSCLMG00005021392 | 0.738972234 | 0.001475508 |
| ENSCLMG00005021817 | 0.739112208 | 0.000566117 |
| ENSCLMG00005005650 | 0.739405373 | 0.037496742 |
| ENSCLMG00005002063 | 0.741436374 | 0.029224556 |
| ENSCLMG00005018772 | 0.741575676 | 0.002475354 |
| ENSCLMG00005007917 | 0.742190556 | 0.012516094 |
| ENSCLMG00005009002 | 0.742797965 | 0.003077945 |
| ENSCLMG00005002112 | 0.743074803 | 0.001080363 |
| ENSCLMG00005017436 | 0.744406384 | 0.048934785 |
| ENSCLMG00005020392 | 0.744847728 | 6.95E-05 |
| ENSCLMG00005007287 | 0.744876117 | 0.004485996 |
| ENSCLMG00005012188 | 0.745179557 | 0.016333422 |
| ENSCLMG00005005825 | 0.746108682 | 0.042073022 |
| ENSCLMG00005003750 | 0.746500072 | 0.018832846 |
| ENSCLMG00005001158 | 0.746650539 | 0.001902188 |
| ENSCLMG00005010915 | 0.74709431 | 0.001244544 |
| ENSCLMG00005002994 | 0.747317271 | 0.003958663 |
| ENSCLMG00005011069 | 0.74828576 | 0.000247309 |
| ENSCLMG00005006607 | 0.749349275 | 0.004978606 |
| ENSCLMG00005007487 | 0.750206668 | 0.008320423 |
| ENSCLMG00005008521 | 0.750466393 | 0.023580985 |
| ENSCLMG00005022862 | 0.750685332 | 0.010973489 |
| ENSCLMG00005008579 | 0.751394432 | 0.012463886 |
| ENSCLMG00005020386 | 0.751584274 | 0.011525092 |
| ENSCLMG00005021910 | 0.751953511 | 0.018323401 |
| ENSCLMG00005017094 | 0.752679339 | 0.005103422 |
| ENSCLMG00005012118 | 0.752905724 | 0.00549926 |
| ENSCLMG00005017961 | 0.753722759 | 0.03905893 |
| ENSCLMG00005007423 | 0.754067789 | 0.003839147 |
| ENSCLMG00005011946 | 0.754526003 | 0.023482441 |
| ENSCLMG00005012958 | 0.755011063 | 0.003620293 |
| ENSCLMG00005020204 | 0.755050065 | 0.000201708 |
| ENSCLMG00005014389 | 0.755150692 | 0.000290923 |
| ENSCLMG00005008759 | 0.755381349 | 0.007753474 |
| ENSCLMG00005008825 | 0.755686261 | 0.000340329 |
| ENSCLMG00005012523 | 0.756396072 | 0.000188093 |
| ENSCLMG00005003893 | 0.758071993 | 0.002858129 |
| ENSCLMG00005001820 | 0.760305633 | 0.005026051 |
| ENSCLMG00005007453 | 0.760784366 | 0.000880012 |
| ENSCLMG00005013441 | 0.761069019 | 0.002589123 |
| ENSCLMG00005003522 | 0.761077164 | 0.000826216 |
| ENSCLMG00005006253 | 0.76183468 | 0.008964727 |
| ENSCLMG00005012022 | 0.764243464 | 0.009973335 |
| ENSCLMG00005014011 | 0.764365014 | 0.002956665 |
| ENSCLMG00005000514 | 0.764517639 | 0.01610654 |
| ENSCLMG00005003550 | 0.764731743 | 0.018159649 |
| ENSCLMG00005002196 | 0.766225623 | 0.012270428 |
| ENSCLMG00005008893 | 0.766447052 | 0.000161918 |
| ENSCLMG00005011384 | 0.767636263 | 5.08E-05 |
| ENSCLMG00005015054 | 0.770004439 | 0.000644675 |
| ENSCLMG00005015452 | 0.770126109 | 0.027945725 |
| ENSCLMG00005001860 | 0.770981255 | 0.009525508 |
| ENSCLMG00005001276 | 0.771077672 | 0.003274215 |
| ENSCLMG00005007770 | 0.771893901 | 0.001317272 |
| ENSCLMG00005006074 | 0.772815596 | 0.01752059 |
| ENSCLMG00005011661 | 0.772840121 | 0.003556366 |
| ENSCLMG00005011015 | 0.773192686 | 0.013425508 |
| ENSCLMG00005014278 | 0.773459416 | 0.004268641 |
| ENSCLMG00005017663 | 0.77472698 | 0.00418801 |
| ENSCLMG00005013395 | 0.774990371 | 0.011958621 |
| ENSCLMG00005010667 | 0.775228233 | 0.000916706 |
| ENSCLMG00005019371 | 0.775409985 | 0.00885549 |
| ENSCLMG00005007915 | 0.777116603 | 0.010307913 |
| ENSCLMG00005008199 | 0.779561594 | 0.003471639 |
| ENSCLMG00005008332 | 0.779965166 | 0.007814925 |
| ENSCLMG00005010217 | 0.780243217 | 0.004446686 |
| ENSCLMG00005013819 | 0.781241039 | 0.000501487 |
| ENSCLMG00005020083 | 0.781608261 | 0.001297209 |
| ENSCLMG00005000936 | 0.783302612 | 0.000231035 |
| ENSCLMG00005022675 | 0.785417051 | 7.29E-06 |
| ENSCLMG00005006264 | 0.785636841 | 0.007041434 |
| ENSCLMG00005008385 | 0.788157119 | 0.012759072 |
| ENSCLMG00005006530 | 0.788900249 | 0.027855509 |
| ENSCLMG00005019239 | 0.789012763 | 0.006885803 |
| ENSCLMG00005019284 | 0.789172837 | 0.021731568 |
| ENSCLMG00005001759 | 0.789863691 | 0.041115018 |
| ENSCLMG00005000571 | 0.78992005 | 0.001680098 |
| ENSCLMG00005018702 | 0.790027478 | 0.007031913 |
| ENSCLMG00005014719 | 0.790377635 | 0.001433871 |
| ENSCLMG00005008612 | 0.790938942 | 0.000314671 |
| ENSCLMG00005009601 | 0.792664879 | 0.004364433 |
| ENSCLMG00005012364 | 0.792802054 | 0.042625663 |
| ENSCLMG00005013105 | 0.793291068 | 0.048614612 |
| ENSCLMG00005022239 | 0.794464422 | 0.000149829 |
| ENSCLMG00005009851 | 0.794745756 | 0.000548605 |
| ENSCLMG00005014336 | 0.794755551 | 0.011421894 |
| ENSCLMG00005010778 | 0.79665057 | 0.03434511 |
| ENSCLMG00005022660 | 0.797021543 | 0.002226735 |
| ENSCLMG00005019125 | 0.797504292 | 0.004642971 |
| ENSCLMG00005003877 | 0.797657845 | 0.000198503 |
| ENSCLMG00005003396 | 0.798851666 | 0.000679806 |
| ENSCLMG00005007269 | 0.798980286 | 0.036318594 |
| ENSCLMG00005003486 | 0.800063083 | 0.000641016 |
| ENSCLMG00005009351 | 0.800111485 | 0.008375693 |
| ENSCLMG00005017306 | 0.800470441 | 0.004579414 |
| ENSCLMG00005010845 | 0.800582954 | 0.038560119 |
| ENSCLMG00005004148 | 0.800727978 | 0.021420178 |
| ENSCLMG00005019093 | 0.800823808 | 0.000101241 |
| ENSCLMG00005015676 | 0.801865825 | 0.012954978 |
| ENSCLMG00005012784 | 0.802213724 | 0.002866668 |
| ENSCLMG00005010317 | 0.80338535 | 9.07E-05 |
| ENSCLMG00005003435 | 0.803432237 | 0.001183412 |
| ENSCLMG00005018183 | 0.80347854 | 0.000740028 |
| ENSCLMG00005012508 | 0.803688571 | 0.000261454 |
| ENSCLMG00005015858 | 0.804339677 | 0.01389749 |
| ENSCLMG00005006488 | 0.805487415 | 0.000259068 |
| ENSCLMG00005020152 | 0.805579942 | 0.005670513 |
| ENSCLMG00005017295 | 0.807162675 | 1.97E-05 |
| ENSCLMG00005010173 | 0.807667541 | 0.00056691 |
| ENSCLMG00005018158 | 0.808287427 | 2.80E-06 |
| ENSCLMG00005007583 | 0.81015261 | 0.005806365 |
| ENSCLMG00005002903 | 0.811065848 | 0.007206647 |
| ENSCLMG00005016892 | 0.811390483 | 0.001130664 |
| ENSCLMG00005011239 | 0.812468618 | 0.009382566 |
| ENSCLMG00005000897 | 0.815527933 | 0.000134071 |
| ENSCLMG00005000804 | 0.81687158 | 0.000111588 |
| ENSCLMG00005017091 | 0.818423066 | 0.005014441 |
| ENSCLMG00005004085 | 0.818523464 | 0.000299239 |
| ENSCLMG00005020755 | 0.821585748 | 0.000595986 |
| ENSCLMG00005016081 | 0.822250465 | 0.000222965 |
| ENSCLMG00005011154 | 0.823007024 | 0.005794378 |
| ENSCLMG00005002241 | 0.823149606 | 0.017431848 |
| ENSCLMG00005018501 | 0.823189896 | 0.000208498 |
| ENSCLMG00005006798 | 0.823227954 | 0.010270623 |
| ENSCLMG00005000717 | 0.823389872 | 0.008587263 |
| ENSCLMG00005000452 | 0.823448836 | 0.004798001 |
| ENSCLMG00005004318 | 0.823797082 | 6.59E-05 |
| ENSCLMG00005008550 | 0.824502272 | 0.004731818 |
| ENSCLMG00005011232 | 0.824715354 | 3.80E-05 |
| ENSCLMG00005005305 | 0.825013759 | 0.000962493 |
| ENSCLMG00005019486 | 0.825717459 | 0.003027856 |
| ENSCLMG00005020166 | 0.826503793 | 0.021092287 |
| ENSCLMG00005019279 | 0.827602524 | 0.049178806 |
| ENSCLMG00005008275 | 0.827984294 | 0.003129434 |
| ENSCLMG00005015716 | 0.82859637 | 0.0232191 |
| ENSCLMG00005015235 | 0.828988632 | 5.17E-05 |
| ENSCLMG00005007150 | 0.829121286 | 0.035698968 |
| ENSCLMG00005017451 | 0.830378504 | 0.000993131 |
| ENSCLMG00005015977 | 0.830536603 | 0.001051892 |
| ENSCLMG00005001667 | 0.831129819 | 9.10E-05 |
| ENSCLMG00005012340 | 0.831197941 | 0.00324108 |
| ENSCLMG00005015086 | 0.832123116 | 0.017255042 |
| ENSCLMG00005008062 | 0.832507795 | 0.044185459 |
| ENSCLMG00005010562 | 0.835007351 | 0.00324108 |
| ENSCLMG00005011922 | 0.835048225 | 0.00160349 |
| ENSCLMG00005001707 | 0.835531417 | 2.60E-05 |
| ENSCLMG00005018926 | 0.835804056 | 0.000176975 |
| ENSCLMG00005015786 | 0.836117993 | 0.005137491 |
| ENSCLMG00005000864 | 0.838534947 | 3.86E-05 |
| ENSCLMG00005018357 | 0.840598944 | 0.000835716 |
| ENSCLMG00005002790 | 0.840821497 | 0.000172543 |
| ENSCLMG00005011347 | 0.841016943 | 0.005142064 |
| ENSCLMG00005008083 | 0.84224391 | 0.004446686 |
| ENSCLMG00005002546 | 0.842248783 | 9.52E-05 |
| ENSCLMG00005011865 | 0.843191821 | 3.70E-06 |
| ENSCLMG00005011380 | 0.843571247 | 0.000533049 |
| ENSCLMG00005011221 | 0.845158374 | 0.016449047 |
| ENSCLMG00005007263 | 0.84627711 | 0.000132063 |
| ENSCLMG00005003470 | 0.846417533 | 0.043404118 |
| ENSCLMG00005017610 | 0.847426625 | 0.000170287 |
| ENSCLMG00005003808 | 0.847768879 | 1.96E-06 |
| ENSCLMG00005008058 | 0.847776308 | 0.001712775 |
| ENSCLMG00005020201 | 0.848915484 | 0.011503734 |
| ENSCLMG00005012343 | 0.849446126 | 8.96E-05 |
| ENSCLMG00005010775 | 0.849785112 | 0.00493754 |
| ENSCLMG00005020229 | 0.850145522 | 0.003723586 |
| ENSCLMG00005003843 | 0.850209234 | 6.02E-05 |
| ENSCLMG00005000797 | 0.850598345 | 0.046911509 |
| ENSCLMG00005011331 | 0.852295412 | 0.00056241 |
| ENSCLMG00005013906 | 0.852351003 | 0.038766643 |
| ENSCLMG00005000956 | 0.85312336 | 0.000813791 |
| ENSCLMG00005006135 | 0.853551901 | 0.005822788 |
| ENSCLMG00005003964 | 0.856401647 | 0.002126943 |
| ENSCLMG00005011146 | 0.857662425 | 0.006671321 |
| ENSCLMG00005013821 | 0.857667048 | 0.007640079 |
| ENSCLMG00005012807 | 0.858294023 | 4.88E-06 |
| ENSCLMG00005002523 | 0.85907484 | 0.006028027 |
| ENSCLMG00005008111 | 0.859934756 | 0.005390988 |
| ENSCLMG00005018696 | 0.860571074 | 0.000107931 |
| ENSCLMG00005005685 | 0.862000991 | 0.005069915 |
| ENSCLMG00005014433 | 0.862101738 | 0.001929598 |
| ENSCLMG00005015976 | 0.862595432 | 0.044913916 |
| ENSCLMG00005003159 | 0.863217206 | 0.009249594 |
| ENSCLMG00005004243 | 0.86351588 | 0.015179661 |
| ENSCLMG00005002282 | 0.864386309 | 0.017841395 |
| ENSCLMG00005005250 | 0.864871856 | 0.010115159 |
| ENSCLMG00005006073 | 0.865031239 | 0.000176476 |
| ENSCLMG00005011645 | 0.86536969 | 0.000555671 |
| ENSCLMG00005008414 | 0.867376601 | 0.048634934 |
| ENSCLMG00005015914 | 0.870662229 | 5.69E-05 |
| ENSCLMG00005019942 | 0.87075871 | 0.010272273 |
| ENSCLMG00005012663 | 0.871453333 | 0.000622498 |
| ENSCLMG00005003501 | 0.871731341 | 0.011602774 |
| ENSCLMG00005006836 | 0.872565061 | 0.00133318 |
| ENSCLMG00005018800 | 0.874109648 | 0.035180954 |
| ENSCLMG00005019113 | 0.874236038 | 0.002078825 |
| ENSCLMG00005018090 | 0.875601762 | 0.005014441 |
| ENSCLMG00005010756 | 0.876990546 | 0.000812297 |
| ENSCLMG00005005588 | 0.877026836 | 0.016410792 |
| ENSCLMG00005020869 | 0.877054866 | 5.26E-05 |
| ENSCLMG00005014138 | 0.877257528 | 0.002550077 |
| ENSCLMG00005002234 | 0.877809516 | 0.008456157 |
| ENSCLMG00005006662 | 0.878254183 | 0.000700411 |
| ENSCLMG00005005050 | 0.880979584 | 0.005220036 |
| ENSCLMG00005019028 | 0.882494362 | 0.010225476 |
| ENSCLMG00005007429 | 0.883261776 | 0.003637923 |
| ENSCLMG00005013141 | 0.883634824 | 0.00017874 |
| ENSCLMG00005022815 | 0.884761852 | 0.000761077 |
| ENSCLMG00005001159 | 0.885584895 | 0.001282838 |
| ENSCLMG00005011668 | 0.886291502 | 0.000993131 |
| ENSCLMG00005016468 | 0.887388032 | 0.002418156 |
| ENSCLMG00005003546 | 0.888000284 | 0.004749688 |
| ENSCLMG00005000381 | 0.889092538 | 0.003653564 |
| ENSCLMG00005009093 | 0.889997817 | 0.048854177 |
| ENSCLMG00005017896 | 0.8911953 | 9.11E-06 |
| ENSCLMG00005006506 | 0.893622275 | 0.028169183 |
| ENSCLMG00005022585 | 0.894569203 | 0.000197576 |
| ENSCLMG00005020708 | 0.89499738 | 4.72E-05 |
| ENSCLMG00005011910 | 0.895772834 | 0.014002538 |
| ENSCLMG00005002421 | 0.895842728 | 0.003256741 |
| ENSCLMG00005022968 | 0.895914897 | 1.38E-06 |
| ENSCLMG00005020155 | 0.896867332 | 6.79E-05 |
| ENSCLMG00005017310 | 0.896891903 | 0.01500895 |
| ENSCLMG00005004985 | 0.897097062 | 0.000843307 |
| ENSCLMG00005006132 | 0.897647828 | 0.000483502 |
| ENSCLMG00005016469 | 0.89931222 | 0.000544807 |
| ENSCLMG00005002189 | 0.900053493 | 0.009249594 |
| ENSCLMG00005011751 | 0.901419736 | 0.001439752 |
| ENSCLMG00005019426 | 0.902773948 | 0.008344682 |
| ENSCLMG00005015060 | 0.905973006 | 0.025246918 |
| ENSCLMG00005019972 | 0.906140052 | 0.022302991 |
| ENSCLMG00005006133 | 0.906173018 | 0.003647008 |
| ENSCLMG00005009487 | 0.906194171 | 9.12E-06 |
| ENSCLMG00005010084 | 0.907068154 | 0.001515141 |
| ENSCLMG00005021465 | 0.907137791 | 0.002200025 |
| ENSCLMG00005000880 | 0.908644443 | 0.001572276 |
| ENSCLMG00005008608 | 0.912395942 | 0.000287948 |
| ENSCLMG00005005198 | 0.914129537 | 3.14E-05 |
| ENSCLMG00005019618 | 0.914464393 | 9.82E-06 |
| ENSCLMG00005008352 | 0.915205804 | 0.028681184 |
| ENSCLMG00005011200 | 0.915308582 | 0.007018286 |
| ENSCLMG00005020443 | 0.916099997 | 8.60E-05 |
| ENSCLMG00005001139 | 0.916690735 | 0.000843307 |
| ENSCLMG00005001201 | 0.91739379 | 0.029876523 |
| ENSCLMG00005006086 | 0.920773008 | 3.46E-05 |
| ENSCLMG00005010380 | 0.92132023 | 2.00E-05 |
| ENSCLMG00005013401 | 0.921353747 | 0.029925703 |
| ENSCLMG00005003770 | 0.921996474 | 9.06E-07 |
| ENSCLMG00005005362 | 0.922291609 | 0.00050989 |
| ENSCLMG00005001165 | 0.922527176 | 0.004393599 |
| ENSCLMG00005011644 | 0.922877422 | 0.027661338 |
| ENSCLMG00005011042 | 0.923229486 | 1.48E-06 |
| ENSCLMG00005017318 | 0.924170195 | 0.02845931 |
| ENSCLMG00005003783 | 0.924311393 | 3.21E-06 |
| ENSCLMG00005002956 | 0.926828112 | 6.85E-05 |
| ENSCLMG00005014630 | 0.928702779 | 2.40E-05 |
| ENSCLMG00005005220 | 0.929824706 | 7.92E-05 |
| ENSCLMG00005005460 | 0.93064181 | 0.001397975 |
| ENSCLMG00005020257 | 0.930819195 | 1.37E-05 |
| ENSCLMG00005010604 | 0.930953633 | 5.00E-05 |
| ENSCLMG00005012035 | 0.933367323 | 0.016682247 |
| ENSCLMG00005020724 | 0.934244581 | 0.004487716 |
| ENSCLMG00005010646 | 0.934668842 | 0.017999835 |
| ENSCLMG00005022016 | 0.936987976 | 4.14E-06 |
| ENSCLMG00005016919 | 0.937057412 | 0.012205166 |
| ENSCLMG00005019755 | 0.937906069 | 0.023772932 |
| ENSCLMG00005010787 | 0.938214041 | 0.000651592 |
| ENSCLMG00005003048 | 0.938748315 | 0.006532369 |
| ENSCLMG00005021680 | 0.938921327 | 0.000758754 |
| ENSCLMG00005001206 | 0.939721143 | 0.031837844 |
| ENSCLMG00005006729 | 0.941652535 | 0.000309561 |
| ENSCLMG00005012369 | 0.941722658 | 5.46E-07 |
| ENSCLMG00005005706 | 0.941755788 | 0.012661857 |
| ENSCLMG00005019804 | 0.941991367 | 0.018348582 |
| ENSCLMG00005017849 | 0.942602655 | 7.67E-05 |
| ENSCLMG00005008577 | 0.943264604 | 0.006380742 |
| ENSCLMG00005006686 | 0.943354372 | 6.43E-05 |
| ENSCLMG00005001164 | 0.944688945 | 0.012397108 |
| ENSCLMG00005010633 | 0.944979932 | 0.006004292 |
| ENSCLMG00005021352 | 0.947059785 | 0.016076559 |
| ENSCLMG00005002043 | 0.947534825 | 0.000584278 |
| ENSCLMG00005015350 | 0.947772666 | 0.000687009 |
| ENSCLMG00005007222 | 0.947969257 | 0.015998504 |
| ENSCLMG00005000339 | 0.949415891 | 7.06E-06 |
| ENSCLMG00005006821 | 0.950528029 | 0.00030068 |
| ENSCLMG00005006643 | 0.950667738 | 0.004865064 |
| ENSCLMG00005006174 | 0.952526063 | 0.00080808 |
| ENSCLMG00005001212 | 0.95472466 | 0.016491662 |
| ENSCLMG00005005958 | 0.955917423 | 0.006153232 |
| ENSCLMG00005018044 | 0.956442966 | 1.27E-07 |
| ENSCLMG00005008390 | 0.957840085 | 1.53E-05 |
| ENSCLMG00005008753 | 0.960338797 | 0.00067382 |
| ENSCLMG00005004760 | 0.960594011 | 0.00312481 |
| ENSCLMG00005015908 | 0.961273479 | 0.000164878 |
| ENSCLMG00005001818 | 0.961499879 | 0.000106315 |
| ENSCLMG00005006655 | 0.963230267 | 0.000406591 |
| ENSCLMG00005001796 | 0.96427105 | 0.001282838 |
| ENSCLMG00005004828 | 0.964571121 | 5.21E-06 |
| ENSCLMG00005011059 | 0.965387325 | 0.000293974 |
| ENSCLMG00005012446 | 0.965641116 | 0.041009661 |
| ENSCLMG00005012371 | 0.966267461 | 0.000167953 |
| ENSCLMG00005014979 | 0.966304954 | 0.000164878 |
| ENSCLMG00005022979 | 0.966408682 | 0.004579414 |
| ENSCLMG00005013624 | 0.967317692 | 0.022293563 |
| ENSCLMG00005009827 | 0.968003911 | 0.000217365 |
| ENSCLMG00005003078 | 0.968370449 | 0.000437594 |
| ENSCLMG00005003333 | 0.96840859 | 0.005400941 |
| ENSCLMG00005006142 | 0.968784579 | 0.00041282 |
| ENSCLMG00005016549 | 0.969633716 | 0.006028027 |
| ENSCLMG00005001941 | 0.972059215 | 0.002866668 |
| ENSCLMG00005011367 | 0.972756824 | 0.004798212 |
| ENSCLMG00005014319 | 0.973535002 | 1.52E-05 |
| ENSCLMG00005017685 | 0.973649651 | 1.10E-05 |
| ENSCLMG00005016111 | 0.975000325 | 0.003544265 |
| ENSCLMG00005006159 | 0.975731067 | 1.46E-05 |
| ENSCLMG00005015641 | 0.976883503 | 0.038618207 |
| ENSCLMG00005013255 | 0.977095586 | 4.08E-06 |
| ENSCLMG00005003992 | 0.977326955 | 0.022872338 |
| ENSCLMG00005015986 | 0.977491028 | 0.00184429 |
| ENSCLMG00005010398 | 0.979176108 | 0.02118385 |
| ENSCLMG00005012375 | 0.979715117 | 0.000216157 |
| ENSCLMG00005010133 | 0.981684408 | 0.007012734 |
| ENSCLMG00005017376 | 0.98227946 | 0.003968409 |
| ENSCLMG00005010700 | 0.982340063 | 2.00E-05 |
| ENSCLMG00005010276 | 0.982425647 | 7.05E-05 |
| ENSCLMG00005019533 | 0.982800117 | 0.003490759 |
| ENSCLMG00005006143 | 0.985335942 | 2.18E-05 |
| ENSCLMG00005018635 | 0.987393569 | 0.000672293 |
| ENSCLMG00005004530 | 0.987398618 | 0.002005592 |
| ENSCLMG00005006594 | 0.990356108 | 0.003224337 |
| ENSCLMG00005000127 | 0.991031533 | 0.001060348 |
| ENSCLMG00005020168 | 0.99199448 | 2.20E-09 |
| ENSCLMG00005012274 | 0.992263575 | 6.52E-06 |
| ENSCLMG00005013812 | 0.99374372 | 3.39E-07 |
| ENSCLMG00005015907 | 0.994475245 | 0.002043119 |
| ENSCLMG00005018138 | 0.996568354 | 0.0443406 |
| ENSCLMG00005021685 | 0.997076044 | 0.003606265 |
| ENSCLMG00005010117 | 0.998269087 | 3.80E-05 |
| ENSCLMG00005011218 | 0.999350181 | 9.36E-05 |
| ENSCLMG00005001008 | 0.999573522 | 4.99E-05 |
| ENSCLMG00005021514 | 1.000467663 | 7.98E-07 |
| ENSCLMG00005005674 | 1.002663711 | 0.001359122 |
| ENSCLMG00005008103 | 1.003166794 | 6.38E-05 |
| ENSCLMG00005000149 | 1.003213983 | 0.000220083 |
| ENSCLMG00005015128 | 1.006292209 | 1.24E-05 |
| ENSCLMG00005007636 | 1.006316623 | 0.001070018 |
| ENSCLMG00005008778 | 1.008115526 | 1.98E-06 |
| ENSCLMG00005017463 | 1.009540519 | 0.000403884 |
| ENSCLMG00005019338 | 1.009738795 | 1.95E-05 |
| ENSCLMG00005007237 | 1.010103458 | 0.0003366 |
| ENSCLMG00005020056 | 1.010195588 | 1.96E-05 |
| ENSCLMG00005003184 | 1.011888525 | 1.73E-06 |
| ENSCLMG00005000534 | 1.014362683 | 0.001774695 |
| ENSCLMG00005010719 | 1.01467738 | 0.000632769 |
| ENSCLMG00005004866 | 1.015387994 | 1.61E-06 |
| ENSCLMG00005014515 | 1.015836602 | 2.92E-05 |
| ENSCLMG00005002392 | 1.01718362 | 0.003466546 |
| ENSCLMG00005018488 | 1.018709351 | 5.51E-06 |
| ENSCLMG00005020825 | 1.020713723 | 1.10E-05 |
| ENSCLMG00005019480 | 1.021268822 | 0.026813249 |
| ENSCLMG00005015895 | 1.022322074 | 0.000921267 |
| ENSCLMG00005015151 | 1.023103883 | 1.87E-05 |
| ENSCLMG00005014861 | 1.023180682 | 0.001486199 |
| ENSCLMG00005000875 | 1.025051941 | 0.001158993 |
| ENSCLMG00005009622 | 1.02510879 | 0.001475508 |
| ENSCLMG00005005382 | 1.026493707 | 0.000110646 |
| ENSCLMG00005010868 | 1.028125265 | 1.13E-05 |
| ENSCLMG00005009623 | 1.029002224 | 2.05E-06 |
| ENSCLMG00005008253 | 1.029592892 | 0.012950815 |
| ENSCLMG00005014632 | 1.030049324 | 2.97E-06 |
| ENSCLMG00005013555 | 1.030928292 | 0.000432966 |
| ENSCLMG00005013713 | 1.032231488 | 2.82E-05 |
| ENSCLMG00005009781 | 1.032396334 | 0.001712775 |
| ENSCLMG00005011670 | 1.032680546 | 9.92E-05 |
| ENSCLMG00005009037 | 1.032934835 | 4.09E-05 |
| ENSCLMG00005004511 | 1.036334807 | 0.002717629 |
| ENSCLMG00005016710 | 1.037290999 | 0.00069295 |
| ENSCLMG00005001271 | 1.038169957 | 4.79E-05 |
| ENSCLMG00005008451 | 1.039266101 | 0.03957188 |
| ENSCLMG00005006786 | 1.040242602 | 0.001851054 |
| ENSCLMG00005013811 | 1.043536182 | 3.26E-05 |
| ENSCLMG00005001170 | 1.045716151 | 0.020195529 |
| ENSCLMG00005011641 | 1.046278004 | 0.037043908 |
| ENSCLMG00005020385 | 1.046551745 | 0.00063338 |
| ENSCLMG00005005064 | 1.047984745 | 4.43E-07 |
| ENSCLMG00005013817 | 1.048142676 | 3.62E-05 |
| ENSCLMG00005007094 | 1.049194348 | 0.003544142 |
| ENSCLMG00005001127 | 1.049263755 | 3.28E-07 |
| ENSCLMG00005003993 | 1.050676939 | 0.002351625 |
| ENSCLMG00005014462 | 1.051328238 | 8.76E-05 |
| ENSCLMG00005010830 | 1.053078924 | 0.012165869 |
| ENSCLMG00005020269 | 1.056638521 | 0.011503734 |
| ENSCLMG00005015309 | 1.057099892 | 0.003349293 |
| ENSCLMG00005016338 | 1.05880431 | 0.018939968 |
| ENSCLMG00005000303 | 1.059887367 | 0.002898438 |
| ENSCLMG00005002197 | 1.060339152 | 0.016669708 |
| ENSCLMG00005018950 | 1.060745256 | 0.000426796 |
| ENSCLMG00005005117 | 1.061679841 | 5.26E-05 |
| ENSCLMG00005019733 | 1.061841976 | 7.84E-07 |
| ENSCLMG00005001171 | 1.06196248 | 0.001034722 |
| ENSCLMG00005004143 | 1.064556058 | 8.09E-06 |
| ENSCLMG00005004955 | 1.06467762 | 3.70E-05 |
| ENSCLMG00005002066 | 1.064895533 | 0.002514556 |
| ENSCLMG00005004111 | 1.065037089 | 5.15E-08 |
| ENSCLMG00005010600 | 1.065704608 | 1.55E-08 |
| ENSCLMG00005008037 | 1.06615494 | 0.000740168 |
| ENSCLMG00005019582 | 1.070130382 | 0.00237982 |
| ENSCLMG00005010763 | 1.070710231 | 0.000129953 |
| ENSCLMG00005006563 | 1.07129643 | 2.17E-06 |
| ENSCLMG00005003775 | 1.072029422 | 1.26E-05 |
| ENSCLMG00005019432 | 1.072441083 | 0.000499111 |
| ENSCLMG00005011063 | 1.074347024 | 1.43E-05 |
| ENSCLMG00005001221 | 1.075757664 | 0.000249947 |
| ENSCLMG00005011328 | 1.079908374 | 2.66E-05 |
| ENSCLMG00005020297 | 1.079983883 | 0.001984547 |
| ENSCLMG00005011149 | 1.081439893 | 0.000516015 |
| ENSCLMG00005016994 | 1.084856092 | 0.004575631 |
| ENSCLMG00005009336 | 1.085195104 | 0.000244922 |
| ENSCLMG00005019421 | 1.086083757 | 0.013127429 |
| ENSCLMG00005013085 | 1.086772536 | 0.004076591 |
| ENSCLMG00005012003 | 1.087202044 | 3.55E-05 |
| ENSCLMG00005015975 | 1.088996262 | 0.003221133 |
| ENSCLMG00005003454 | 1.089318296 | 0.000646615 |
| ENSCLMG00005021200 | 1.089697054 | 0.000119308 |
| ENSCLMG00005005354 | 1.092826132 | 0.000403844 |
| ENSCLMG00005008713 | 1.093858464 | 1.00E-05 |
| ENSCLMG00005000275 | 1.094681817 | 3.87E-05 |
| ENSCLMG00005011783 | 1.094898015 | 0.003054501 |
| ENSCLMG00005001140 | 1.10071231 | 0.013811383 |
| ENSCLMG00005007990 | 1.102545607 | 0.006880676 |
| ENSCLMG00005001398 | 1.104429802 | 4.00E-07 |
| ENSCLMG00005001477 | 1.106693882 | 1.82E-05 |
| ENSCLMG00005010387 | 1.106973423 | 0.006947331 |
| ENSCLMG00005004093 | 1.1076756 | 3.49E-06 |
| ENSCLMG00005017344 | 1.107773707 | 0.008341293 |
| ENSCLMG00005010622 | 1.110900275 | 3.58E-05 |
| ENSCLMG00005014478 | 1.112613767 | 0.029995441 |
| ENSCLMG00005008023 | 1.114238395 | 2.90E-05 |
| ENSCLMG00005021798 | 1.114268758 | 4.44E-06 |
| ENSCLMG00005014634 | 1.115650961 | 0.000434213 |
| ENSCLMG00005010682 | 1.115696342 | 0.014134238 |
| ENSCLMG00005014379 | 1.117505463 | 0.013226279 |
| ENSCLMG00005002471 | 1.119169845 | 6.46E-05 |
| ENSCLMG00005010942 | 1.121200606 | 0.000484715 |
| ENSCLMG00005015486 | 1.121899817 | 3.84E-05 |
| ENSCLMG00005018107 | 1.122856395 | 6.00E-05 |
| ENSCLMG00005020165 | 1.12292111 | 0.002864965 |
| ENSCLMG00005022423 | 1.124797478 | 1.35E-06 |
| ENSCLMG00005002315 | 1.126272664 | 0.032333003 |
| ENSCLMG00005017809 | 1.126722529 | 9.02E-07 |
| ENSCLMG00005016467 | 1.127690359 | 0.003069157 |
| ENSCLMG00005004165 | 1.127751929 | 7.61E-07 |
| ENSCLMG00005010357 | 1.128349143 | 1.06E-07 |
| ENSCLMG00005010985 | 1.12928434 | 1.34E-07 |
| ENSCLMG00005022952 | 1.130133128 | 1.83E-08 |
| ENSCLMG00005013160 | 1.131849322 | 2.12E-05 |
| ENSCLMG00005013862 | 1.132580273 | 0.016511571 |
| ENSCLMG00005016134 | 1.135786815 | 0.032171642 |
| ENSCLMG00005017224 | 1.137003057 | 0.007236517 |
| ENSCLMG00005017508 | 1.137700597 | 0.00190981 |
| ENSCLMG00005018675 | 1.138022849 | 2.38E-05 |
| ENSCLMG00005016299 | 1.141282291 | 4.33E-05 |
| ENSCLMG00005018198 | 1.142943002 | 2.83E-05 |
| ENSCLMG00005012342 | 1.144720611 | 0.008672941 |
| ENSCLMG00005013040 | 1.14519992 | 1.10E-05 |
| ENSCLMG00005001305 | 1.145226571 | 0.000349212 |
| ENSCLMG00005013682 | 1.145260878 | 0.001136035 |
| ENSCLMG00005016424 | 1.145938187 | 0.000416784 |
| ENSCLMG00005012323 | 1.146346698 | 2.25E-07 |
| ENSCLMG00005010396 | 1.146919899 | 0.001348299 |
| ENSCLMG00005012547 | 1.149772701 | 0.003968409 |
| ENSCLMG00005002448 | 1.151920991 | 0.000305834 |
| ENSCLMG00005020253 | 1.152226639 | 5.83E-06 |
| ENSCLMG00005014855 | 1.152511928 | 6.79E-05 |
| ENSCLMG00005018775 | 1.153836133 | 0.005806365 |
| ENSCLMG00005014976 | 1.156056739 | 0.027783143 |
| ENSCLMG00005019669 | 1.156475752 | 0.000553472 |
| ENSCLMG00005018178 | 1.159029325 | 0.001174144 |
| ENSCLMG00005010235 | 1.159681479 | 0.00329261 |
| ENSCLMG00005011093 | 1.160744033 | 3.81E-05 |
| ENSCLMG00005006642 | 1.161281132 | 5.42E-06 |
| ENSCLMG00005001094 | 1.162554212 | 0.045467749 |
| ENSCLMG00005000077 | 1.163093122 | 0.000909872 |
| ENSCLMG00005004863 | 1.164021294 | 0.017015779 |
| ENSCLMG00005017027 | 1.164090568 | 6.88E-08 |
| ENSCLMG00005002277 | 1.164514014 | 0.000987454 |
| ENSCLMG00005002654 | 1.168624332 | 3.28E-06 |
| ENSCLMG00005015281 | 1.169444921 | 3.08E-05 |
| ENSCLMG00005004456 | 1.173508884 | 3.52E-07 |
| ENSCLMG00005022078 | 1.174364453 | 0.000172973 |
| ENSCLMG00005016915 | 1.178395858 | 0.012020683 |
| ENSCLMG00005019470 | 1.182422895 | 0.013135399 |
| ENSCLMG00005007267 | 1.183948377 | 1.10E-05 |
| ENSCLMG00005017834 | 1.185447979 | 0.006457946 |
| ENSCLMG00005018084 | 1.186061029 | 0.022467051 |
| ENSCLMG00005003165 | 1.186274273 | 0.002295998 |
| ENSCLMG00005008864 | 1.191987804 | 2.31E-05 |
| ENSCLMG00005010031 | 1.194323414 | 5.09E-08 |
| ENSCLMG00005000940 | 1.199467689 | 0.001249974 |
| ENSCLMG00005020026 | 1.199938358 | 3.20E-09 |
| ENSCLMG00005015860 | 1.204933022 | 0.018824477 |
| ENSCLMG00005011233 | 1.205373589 | 2.01E-05 |
| ENSCLMG00005017421 | 1.205384035 | 0.00308292 |
| ENSCLMG00005005569 | 1.207469397 | 0.000516015 |
| ENSCLMG00005011771 | 1.20914915 | 5.90E-05 |
| ENSCLMG00005017158 | 1.210183262 | 0.000169667 |
| ENSCLMG00005012049 | 1.21364039 | 6.49E-06 |
| ENSCLMG00005002218 | 1.22093999 | 0.006390128 |
| ENSCLMG00005007995 | 1.221365898 | 0.02606272 |
| ENSCLMG00005002901 | 1.223457553 | 0.002683392 |
| ENSCLMG00005005030 | 1.224894781 | 4.14E-06 |
| ENSCLMG00005010400 | 1.226614222 | 2.24E-06 |
| ENSCLMG00005000562 | 1.227425402 | 8.71E-07 |
| ENSCLMG00005017280 | 1.229029377 | 3.93E-08 |
| ENSCLMG00005011524 | 1.230157256 | 0.016540252 |
| ENSCLMG00005008821 | 1.230304027 | 0.000676146 |
| ENSCLMG00005006658 | 1.232413984 | 7.92E-10 |
| ENSCLMG00005016500 | 1.233839668 | 0.031376851 |
| ENSCLMG00005023005 | 1.238230423 | 0.04016266 |
| ENSCLMG00005013558 | 1.239383312 | 0.012263675 |
| ENSCLMG00005012944 | 1.240056612 | 3.31E-05 |
| ENSCLMG00005006723 | 1.2442706 | 0.015108141 |
| ENSCLMG00005007422 | 1.246736384 | 0.021046227 |
| ENSCLMG00005016089 | 1.255075183 | 0.003085113 |
| ENSCLMG00005002153 | 1.257657429 | 7.22E-14 |
| ENSCLMG00005012138 | 1.272575559 | 2.57E-05 |
| ENSCLMG00005004068 | 1.272656628 | 0.000108344 |
| ENSCLMG00005017950 | 1.274437977 | 0.000993131 |
| ENSCLMG00005007581 | 1.275657188 | 2.86E-08 |
| ENSCLMG00005010925 | 1.275846776 | 2.17E-06 |
| ENSCLMG00005008978 | 1.276637957 | 2.35E-08 |
| ENSCLMG00005012331 | 1.280490701 | 0.006772604 |
| ENSCLMG00005011558 | 1.283844712 | 2.36E-09 |
| ENSCLMG00005002319 | 1.291190493 | 3.26E-07 |
| ENSCLMG00005011101 | 1.291523935 | 0.000740168 |
| ENSCLMG00005004405 | 1.295312041 | 2.59E-05 |
| ENSCLMG00005017369 | 1.296639262 | 0.014012796 |
| ENSCLMG00005012816 | 1.298693161 | 8.48E-07 |
| ENSCLMG00005011275 | 1.299531597 | 0.002164361 |
| ENSCLMG00005004997 | 1.299614065 | 0.000269542 |
| ENSCLMG00005007516 | 1.300452917 | 4.67E-08 |
| ENSCLMG00005011008 | 1.302667341 | 2.15E-07 |
| ENSCLMG00005007892 | 1.302715772 | 1.85E-16 |
| ENSCLMG00005011262 | 1.303660897 | 4.94E-10 |
| ENSCLMG00005010036 | 1.305620486 | 4.06E-05 |
| ENSCLMG00005022647 | 1.310927873 | 0.006901707 |
| ENSCLMG00005022113 | 1.317077884 | 0.000340329 |
| ENSCLMG00005000478 | 1.317735083 | 0.000124032 |
| ENSCLMG00005015897 | 1.322736106 | 8.64E-07 |
| ENSCLMG00005015305 | 1.325409731 | 0.003607772 |
| ENSCLMG00005004895 | 1.327246772 | 7.89E-05 |
| ENSCLMG00005008690 | 1.327433327 | 5.87E-14 |
| ENSCLMG00005007358 | 1.328386892 | 9.69E-06 |
| ENSCLMG00005022940 | 1.33060534 | 0.005462644 |
| ENSCLMG00005011195 | 1.332135041 | 6.72E-05 |
| ENSCLMG00005018187 | 1.332442271 | 2.14E-12 |
| ENSCLMG00005007326 | 1.336156579 | 2.24E-05 |
| ENSCLMG00005019963 | 1.336677726 | 0.009245736 |
| ENSCLMG00005011526 | 1.337112793 | 0.002827478 |
| ENSCLMG00005003242 | 1.337168575 | 0.008355704 |
| ENSCLMG00005018050 | 1.340766117 | 0.007386715 |
| ENSCLMG00005000081 | 1.346325178 | 0.031566791 |
| ENSCLMG00005020164 | 1.349264792 | 2.75E-07 |
| ENSCLMG00005014297 | 1.349282732 | 0.026570822 |
| ENSCLMG00005015894 | 1.352512816 | 5.27E-06 |
| ENSCLMG00005013954 | 1.355819545 | 0.034830535 |
| ENSCLMG00005009372 | 1.355843767 | 2.07E-07 |
| ENSCLMG00005008655 | 1.355990913 | 4.04E-05 |
| ENSCLMG00005018701 | 1.358390809 | 1.13E-06 |
| ENSCLMG00005022527 | 1.361198042 | 3.95E-05 |
| ENSCLMG00005018042 | 1.362717521 | 2.85E-13 |
| ENSCLMG00005010239 | 1.363658357 | 1.72E-09 |
| ENSCLMG00005018355 | 1.363895125 | 4.72E-05 |
| ENSCLMG00005005560 | 1.367604436 | 3.07E-06 |
| ENSCLMG00005001108 | 1.371677497 | 0.007589066 |
| ENSCLMG00005014867 | 1.372914403 | 4.44E-05 |
| ENSCLMG00005002425 | 1.377465682 | 9.78E-09 |
| ENSCLMG00005009067 | 1.377471364 | 1.27E-05 |
| ENSCLMG00005022875 | 1.382715799 | 0.004272197 |
| ENSCLMG00005011061 | 1.387440923 | 8.53E-05 |
| ENSCLMG00005006527 | 1.396780053 | 2.92E-05 |
| ENSCLMG00005006518 | 1.401998133 | 1.18E-06 |
| ENSCLMG00005015085 | 1.403567043 | 9.27E-15 |
| ENSCLMG00005021928 | 1.404039126 | 0.01236461 |
| ENSCLMG00005013464 | 1.404970783 | 5.02E-07 |
| ENSCLMG00005002148 | 1.40586235 | 0.031837844 |
| ENSCLMG00005016954 | 1.409374118 | 0.000259256 |
| ENSCLMG00005003238 | 1.410713141 | 2.15E-09 |
| ENSCLMG00005013250 | 1.410728387 | 1.77E-07 |
| ENSCLMG00005004906 | 1.411454062 | 5.95E-06 |
| ENSCLMG00005011106 | 1.411849947 | 8.53E-05 |
| ENSCLMG00005018956 | 1.41203553 | 7.16E-05 |
| ENSCLMG00005019251 | 1.416922587 | 0.016682247 |
| ENSCLMG00005022205 | 1.417961279 | 7.73E-08 |
| ENSCLMG00005003547 | 1.421520137 | 0.005067734 |
| ENSCLMG00005003426 | 1.421701111 | 9.35E-14 |
| ENSCLMG00005007460 | 1.423917005 | 0.033609496 |
| ENSCLMG00005008230 | 1.424868223 | 2.29E-09 |
| ENSCLMG00005014628 | 1.430569922 | 0.000267131 |
| ENSCLMG00005014809 | 1.43495189 | 0.005663454 |
| ENSCLMG00005003533 | 1.441000851 | 1.00E-17 |
| ENSCLMG00005008351 | 1.441262614 | 2.90E-07 |
| ENSCLMG00005012330 | 1.444533408 | 0.014174972 |
| ENSCLMG00005020890 | 1.448673023 | 0.000666542 |
| ENSCLMG00005007674 | 1.449588982 | 0.026270718 |
| ENSCLMG00005000908 | 1.451401863 | 0.006417926 |
| ENSCLMG00005012006 | 1.453123537 | 1.73E-05 |
| ENSCLMG00005021634 | 1.455218869 | 0.041115018 |
| ENSCLMG00005006539 | 1.457973302 | 1.06E-07 |
| ENSCLMG00005011559 | 1.458790392 | 1.93E-06 |
| ENSCLMG00005008901 | 1.461058108 | 0.047599848 |
| ENSCLMG00005015635 | 1.465544909 | 7.73E-08 |
| ENSCLMG00005000202 | 1.466545018 | 5.58E-08 |
| ENSCLMG00005006654 | 1.467535339 | 2.15E-08 |
| ENSCLMG00005010185 | 1.468780855 | 4.40E-06 |
| ENSCLMG00005008917 | 1.47420298 | 5.96E-08 |
| ENSCLMG00005008554 | 1.485557363 | 7.19E-06 |
| ENSCLMG00005013808 | 1.486426694 | 0.043486557 |
| ENSCLMG00005011586 | 1.492159579 | 4.09E-05 |
| ENSCLMG00005012946 | 1.49316763 | 0.002118467 |
| ENSCLMG00005010248 | 1.496788987 | 0.016440184 |
| ENSCLMG00005020913 | 1.511185678 | 5.15E-08 |
| ENSCLMG00005006648 | 1.522013688 | 2.85E-08 |
| ENSCLMG00005018988 | 1.522406603 | 7.06E-10 |
| ENSCLMG00005000728 | 1.52437976 | 6.49E-09 |
| ENSCLMG00005002041 | 1.529140313 | 0.000145494 |
| ENSCLMG00005011138 | 1.529702196 | 0.015425151 |
| ENSCLMG00005001151 | 1.538389149 | 0.005085979 |
| ENSCLMG00005006037 | 1.540111676 | 4.14E-06 |
| ENSCLMG00005012337 | 1.54477269 | 2.86E-06 |
| ENSCLMG00005002557 | 1.546808468 | 1.31E-14 |
| ENSCLMG00005013032 | 1.548011264 | 7.08E-13 |
| ENSCLMG00005021066 | 1.551036515 | 1.13E-07 |
| ENSCLMG00005001142 | 1.55555296 | 4.90E-20 |
| ENSCLMG00005005881 | 1.568451636 | 3.11E-05 |
| ENSCLMG00005013603 | 1.568567463 | 0.048272094 |
| ENSCLMG00005010045 | 1.579112218 | 0.031171214 |
| ENSCLMG00005001172 | 1.579207105 | 8.88E-10 |
| ENSCLMG00005011064 | 1.585233049 | 0.000990322 |
| ENSCLMG00005017366 | 1.586748105 | 0.000234716 |
| ENSCLMG00005006699 | 1.587298045 | 0.001421415 |
| ENSCLMG00005012742 | 1.590019535 | 0.000102893 |
| ENSCLMG00005022364 | 1.591419789 | 0.000878451 |
| ENSCLMG00005008667 | 1.592978041 | 1.21E-10 |
| ENSCLMG00005004446 | 1.595832019 | 4.26E-08 |
| ENSCLMG00005007053 | 1.597495177 | 0.030067758 |
| ENSCLMG00005010432 | 1.597719688 | 0.001394426 |
| ENSCLMG00005006687 | 1.604573398 | 1.33E-10 |
| ENSCLMG00005021314 | 1.606065202 | 1.38E-21 |
| ENSCLMG00005017069 | 1.608685542 | 4.86E-07 |
| ENSCLMG00005011915 | 1.609163648 | 0.00022846 |
| ENSCLMG00005011538 | 1.617537301 | 0.000237815 |
| ENSCLMG00005008241 | 1.621725121 | 0.018505198 |
| ENSCLMG00005010094 | 1.622614223 | 0.00026052 |
| ENSCLMG00005009654 | 1.629899603 | 1.38E-09 |
| ENSCLMG00005012335 | 1.644488238 | 9.67E-13 |
| ENSCLMG00005021470 | 1.655786934 | 2.58E-24 |
| ENSCLMG00005020694 | 1.661546699 | 0.006129808 |
| ENSCLMG00005014654 | 1.661587744 | 5.09E-05 |
| ENSCLMG00005005091 | 1.663856466 | 0.023580985 |
| ENSCLMG00005013031 | 1.665520042 | 1.64E-07 |
| ENSCLMG00005008154 | 1.67308211 | 1.82E-06 |
| ENSCLMG00005019130 | 1.677466582 | 5.55E-16 |
| ENSCLMG00005015339 | 1.680976588 | 3.64E-18 |
| ENSCLMG00005016741 | 1.68218223 | 2.11E-12 |
| ENSCLMG00005021834 | 1.689388532 | 8.88E-10 |
| ENSCLMG00005005911 | 1.689719483 | 3.27E-08 |
| ENSCLMG00005016649 | 1.704583397 | 0.010754182 |
| ENSCLMG00005005470 | 1.708894792 | 0.003652413 |
| ENSCLMG00005005085 | 1.71536074 | 1.35E-06 |
| ENSCLMG00005013191 | 1.715824554 | 0.003471978 |
| ENSCLMG00005013683 | 1.717348985 | 0.000153153 |
| ENSCLMG00005012699 | 1.723232189 | 2.54E-06 |
| ENSCLMG00005012916 | 1.729744865 | 0.029257975 |
| ENSCLMG00005003430 | 1.731317544 | 4.67E-08 |
| ENSCLMG00005001899 | 1.737340191 | 0.000890946 |
| ENSCLMG00005014604 | 1.745052743 | 1.64E-11 |
| ENSCLMG00005022243 | 1.747437326 | 1.59E-11 |
| ENSCLMG00005022181 | 1.759309885 | 0.04537054 |
| ENSCLMG00005017101 | 1.760272159 | 0.000366677 |
| ENSCLMG00005017368 | 1.766209856 | 2.27E-06 |
| ENSCLMG00005012662 | 1.773376764 | 1.05E-06 |
| ENSCLMG00005020364 | 1.781656168 | 0.001080147 |
| ENSCLMG00005012098 | 1.792032979 | 2.93E-15 |
| ENSCLMG00005002390 | 1.796411803 | 0.00035486 |
| ENSCLMG00005020475 | 1.800133095 | 0.023542294 |
| ENSCLMG00005000557 | 1.806959678 | 2.45E-12 |
| ENSCLMG00005010855 | 1.808551715 | 2.24E-14 |
| ENSCLMG00005009643 | 1.815932478 | 0.032296024 |
| ENSCLMG00005017225 | 1.817803386 | 0.012048589 |
| ENSCLMG00005007988 | 1.836938353 | 0.013743725 |
| ENSCLMG00005015340 | 1.837767556 | 9.95E-17 |
| ENSCLMG00005005104 | 1.847657362 | 1.56E-16 |
| ENSCLMG00005011639 | 1.847801468 | 4.59E-05 |
| ENSCLMG00005021804 | 1.858154689 | 6.63E-12 |
| ENSCLMG00005015554 | 1.86665823 | 2.51E-06 |
| ENSCLMG00005012729 | 1.867459629 | 0.006006217 |
| ENSCLMG00005000344 | 1.87020065 | 5.52E-27 |
| ENSCLMG00005006908 | 1.88438061 | 0.007585705 |
| ENSCLMG00005006139 | 1.896833747 | 0.001713361 |
| ENSCLMG00005003586 | 1.925374498 | 0.001503952 |
| ENSCLMG00005004947 | 1.927983499 | 3.63E-11 |
| ENSCLMG00005014519 | 1.942434284 | 5.46E-07 |
| ENSCLMG00005011463 | 1.957618997 | 0.027898146 |
| ENSCLMG00005013947 | 1.958298845 | 0.002956665 |
| ENSCLMG00005007260 | 1.968570292 | 7.34E-07 |
| ENSCLMG00005002078 | 1.977712464 | 2.24E-14 |
| ENSCLMG00005000260 | 1.979895302 | 2.64E-10 |
| ENSCLMG00005014776 | 2.008468914 | 4.01E-11 |
| ENSCLMG00005018745 | 2.017791466 | 5.21E-11 |
| ENSCLMG00005022149 | 2.061628287 | 2.93E-06 |
| ENSCLMG00005019568 | 2.079651685 | 1.33E-08 |
| ENSCLMG00005018774 | 2.089724263 | 5.98E-10 |
| ENSCLMG00005003924 | 2.091033595 | 3.47E-20 |
| ENSCLMG00005005791 | 2.103345236 | 4.86E-08 |
| ENSCLMG00005020820 | 2.108472102 | 5.50E-08 |
| ENSCLMG00005010634 | 2.118675843 | 1.68E-07 |
| ENSCLMG00005000572 | 2.152497704 | 0.002364881 |
| ENSCLMG00005008328 | 2.159652698 | 8.15E-10 |
| ENSCLMG00005019050 | 2.160876714 | 8.88E-33 |
| ENSCLMG00005017332 | 2.171612206 | 4.45E-26 |
| ENSCLMG00005012727 | 2.193491328 | 0.000330959 |
| ENSCLMG00005018786 | 2.203891237 | 1.30E-10 |
| ENSCLMG00005017665 | 2.217550819 | 8.54E-10 |
| ENSCLMG00005005864 | 2.219472095 | 4.55E-10 |
| ENSCLMG00005013894 | 2.22116634 | 3.93E-09 |
| ENSCLMG00005004333 | 2.225194111 | 0.007454198 |
| ENSCLMG00005008453 | 2.228751327 | 0.002295998 |
| ENSCLMG00005006548 | 2.236656268 | 3.44E-16 |
| ENSCLMG00005010455 | 2.261681816 | 4.59E-05 |
| ENSCLMG00005007333 | 2.262634456 | 5.87E-11 |
| ENSCLMG00005012382 | 2.273887164 | 0.000561477 |
| ENSCLMG00005002320 | 2.276527642 | 0.000407687 |
| ENSCLMG00005022242 | 2.27906513 | 3.74E-13 |
| ENSCLMG00005002202 | 2.292224348 | 0.002413356 |
| ENSCLMG00005018683 | 2.313191398 | 0.015975101 |
| ENSCLMG00005011140 | 2.330181213 | 3.78E-20 |
| ENSCLMG00005016633 | 2.336727542 | 2.15E-09 |
| ENSCLMG00005006636 | 2.339473712 | 2.93E-20 |
| ENSCLMG00005002493 | 2.372886684 | 8.82E-09 |
| ENSCLMG00005013471 | 2.37604707 | 0.005289975 |
| ENSCLMG00005002713 | 2.393303923 | 0.000817305 |
| ENSCLMG00005011190 | 2.400041457 | 9.79E-14 |
| ENSCLMG00005002498 | 2.419168329 | 2.08E-07 |
| ENSCLMG00005008800 | 2.429920921 | 2.27E-07 |
| ENSCLMG00005007006 | 2.436586198 | 4.27E-19 |
| ENSCLMG00005023053 | 2.439798367 | 2.03E-16 |
| ENSCLMG00005012570 | 2.444596389 | 1.25E-30 |
| ENSCLMG00005010468 | 2.44566804 | 1.68E-26 |
| ENSCLMG00005010671 | 2.455349809 | 0.00168004 |
| ENSCLMG00005021978 | 2.457424837 | 0.02845931 |
| ENSCLMG00005013104 | 2.461897033 | 0.000129089 |
| ENSCLMG00005020206 | 2.470104741 | 5.76E-20 |
| ENSCLMG00005017105 | 2.478759897 | 9.27E-05 |
| ENSCLMG00005010180 | 2.484175205 | 4.93E-17 |
| ENSCLMG00005020470 | 2.507910752 | 3.54E-21 |
| ENSCLMG00005004874 | 2.528914308 | 3.34E-27 |
| ENSCLMG00005017857 | 2.532410397 | 6.07E-17 |
| ENSCLMG00005022332 | 2.5354226 | 6.54E-12 |
| ENSCLMG00005011685 | 2.554237201 | 0.045779089 |
| ENSCLMG00005011543 | 2.576432791 | 7.92E-40 |
| ENSCLMG00005011060 | 2.583669707 | 2.34E-06 |
| ENSCLMG00005011530 | 2.59490353 | 2.02E-29 |
| ENSCLMG00005007521 | 2.628344041 | 2.03E-16 |
| ENSCLMG00005007365 | 2.631865948 | 0.012919315 |
| ENSCLMG00005020785 | 2.683726541 | 2.36E-11 |
| ENSCLMG00005007330 | 2.694557462 | 2.07E-18 |
| ENSCLMG00005011464 | 2.698663261 | 0.003741082 |
| ENSCLMG00005018101 | 2.726649167 | 2.57E-30 |
| ENSCLMG00005008700 | 2.777369491 | 5.98E-22 |
| ENSCLMG00005019487 | 2.790327572 | 1.41E-46 |
| ENSCLMG00005014224 | 2.809228262 | 6.41E-30 |
| ENSCLMG00005008807 | 2.827122181 | 8.03E-47 |
| ENSCLMG00005011806 | 2.828966312 | 2.50E-69 |
| ENSCLMG00005019688 | 2.84829637 | 9.41E-19 |
| ENSCLMG00005020912 | 2.893946975 | 1.64E-35 |
| ENSCLMG00005023096 | 2.894699282 | 6.30E-18 |
| ENSCLMG00005022588 | 2.899724881 | 0.00012883 |
| ENSCLMG00005015861 | 2.95933201 | 0.000965401 |
| ENSCLMG00005008744 | 2.971879956 | 0.000217052 |
| ENSCLMG00005002258 | 2.972169763 | 1.01E-40 |
| ENSCLMG00005018843 | 3.016002149 | 7.88E-12 |
| ENSCLMG00005007328 | 3.037983795 | 1.68E-23 |
| ENSCLMG00005004838 | 3.058530097 | 0.00039552 |
| ENSCLMG00005015335 | 3.154969289 | 4.84E-53 |
| ENSCLMG00005004123 | 3.165480961 | 1.80E-24 |
| ENSCLMG00005014615 | 3.268600054 | 4.08E-06 |
| ENSCLMG00005019424 | 3.324869979 | 3.11E-46 |
| ENSCLMG00005015451 | 3.386842113 | 3.31E-35 |
| ENSCLMG00005014627 | 3.396527901 | 5.98E-10 |
| ENSCLMG00005011973 | 3.401044316 | 8.78E-06 |
| ENSCLMG00005011975 | 3.424501867 | 1.04E-29 |
| ENSCLMG00005007096 | 3.457054019 | 2.68E-24 |
| ENSCLMG00005011189 | 3.530723511 | 1.90E-23 |
| ENSCLMG00005010666 | 3.555904637 | 4.33E-05 |
| ENSCLMG00005007189 | 3.599643354 | 1.79E-65 |
| ENSCLMG00005013890 | 3.610662842 | 1.47E-28 |
| ENSCLMG00005011248 | 3.630813717 | 7.62E-14 |
| ENSCLMG00005003609 | 3.633921041 | 9.82E-56 |
| ENSCLMG00005013994 | 3.75098412 | 7.80E-08 |
| ENSCLMG00005015682 | 3.83780374 | 0.003603123 |
| ENSCLMG00005011921 | 3.934564654 | 0.001095535 |
| ENSCLMG00005020000 | 4.045385508 | 2.05E-05 |
| ENSCLMG00005001377 | 4.17852357 | 1.89E-45 |
| ENSCLMG00005008813 | 4.207326727 | 1.18E-06 |
| ENSCLMG00005017999 | 4.305814597 | 1.49E-22 |
| ENSCLMG00005001410 | 4.326425301 | 5.30E-41 |
| ENSCLMG00005014884 | 4.405138202 | 8.59E-34 |
| ENSCLMG00005019686 | 4.471324532 | 1.41E-11 |
| ENSCLMG00005020865 | 4.513946832 | 4.74E-54 |
| ENSCLMG00005012127 | 4.558195283 | 1.20E-36 |
| ENSCLMG00005015980 | 4.666615429 | 4.41E-106 |
| ENSCLMG00005012378 | 4.719199832 | 5.15E-06 |
| ENSCLMG00005018435 | 4.802818962 | 2.76E-05 |
| ENSCLMG00005001984 | 4.925509488 | 4.59E-05 |
| ENSCLMG00005015292 | 5.377559986 | 0.000992587 |
| ENSCLMG00005003581 | 5.484248535 | 3.08E-169 |
| ENSCLMG00005017102 | 5.636333638 | 1.86E-69 |
| ENSCLMG00005018145 | 6.471140087 | 6.03E-136 |
| ENSCLMG00005002149 | 6.475661162 | 9.58E-06 |
| ENSCLMG00005012320 | 6.486906462 | 1.47E-05 |
| ENSCLMG00005003574 | 6.618134686 | 2.60E-05 |
| ENSCLMG00005011065 | 6.664883368 | 4.96E-06 |
| ENSCLMG00005018119 | 6.668856885 | 1.46E-05 |
| ENSCLMG00005012324 | 6.677721752 | 8.93E-16 |
| ENSCLMG00005011066 | 7.636576602 | 8.87E-19 |
| ENSCLMG00005001985 | 7.830246305 | 1.24E-08 |

**Most significantly regulated genes in TI analysis**

| **ID** | **log2FoldChange** | **P_value(adj)** |
| --- | --- | --- |
| ENSCLMG00005012324 | 6.149161347 | 2.87E-26 |
| ENSCLMG00005012320 | 5.91093733 | 5.25E-09 |
| ENSCLMG00005011066 | 5.828324919 | 5.91E-21 |
| ENSCLMG00005018119 | 5.617872922 | 1.20E-07 |
| ENSCLMG00005018145 | 5.518747688 | 1.92E-54 |
| ENSCLMG00005011065 | 5.123796997 | 6.91E-09 |
| ENSCLMG00005011193 | 4.894134221 | 3.71E-05 |
| ENSCLMG00005001985 | 4.708234389 | 0.000787811 |
| ENSCLMG00005018746 | 4.585331377 | 5.17E-05 |
| ENSCLMG00005003574 | 4.535058841 | 1.50E-05 |
| ENSCLMG00005002149 | 4.490865554 | 5.95E-07 |
| ENSCLMG00005001377 | 4.477013489 | 9.09E-59 |
| ENSCLMG00005019686 | 4.371227975 | 1.59E-36 |
| ENSCLMG00005001984 | 4.319854876 | 8.92E-09 |
| ENSCLMG00005001410 | 4.104830069 | 8.89E-42 |
| ENSCLMG00005015980 | 4.038639247 | 6.67E-52 |
| ENSCLMG00005015292 | 3.942768444 | 0.001125374 |
| ENSCLMG00005017245 | 3.816879513 | 0.001753246 |
| ENSCLMG00005005311 | 3.703138815 | 0.001999802 |
| ENSCLMG00005004398 | 3.647040652 | 0.00375721 |
| ENSCLMG00005010991 | 3.483339361 | 0.001985396 |
| ENSCLMG00005014884 | 3.457778683 | 3.09E-08 |
| ENSCLMG00005011248 | 3.445098679 | 2.78E-13 |
| ENSCLMG00005017999 | 3.433527542 | 1.97E-18 |
| ENSCLMG00005000798 | 3.421387612 | 6.81E-05 |
| ENSCLMG00005015682 | 3.359054401 | 0.000489962 |
| ENSCLMG00005018435 | 3.338139112 | 5.29E-07 |
| ENSCLMG00005017102 | 3.337695335 | 0.001219219 |
| ENSCLMG00005020000 | 3.323632246 | 4.14E-05 |
| ENSCLMG00005005312 | 3.271771879 | 0.006957853 |
| ENSCLMG00005011685 | 3.197072119 | 0.022454293 |
| ENSCLMG00005018843 | 3.041125484 | 1.15E-18 |
| ENSCLMG00005015335 | 3.001055809 | 8.17E-91 |
| ENSCLMG00005018677 | -2.938387874 | 0.044780523 |
| ENSCLMG00005012378 | 2.920056357 | 0.00040531 |
| ENSCLMG00005013890 | 2.847435627 | 1.49E-16 |
| ENSCLMG00005010671 | 2.787309729 | 2.20E-11 |
| ENSCLMG00005004838 | 2.724966777 | 1.08E-05 |
| ENSCLMG00005010047 | -2.703369455 | 6.95E-06 |
| ENSCLMG00005004874 | 2.686146472 | 6.50E-40 |
| ENSCLMG00005013994 | 2.628780603 | 0.001144084 |
| ENSCLMG00005008813 | 2.607011677 | 0.000720176 |
| ENSCLMG00005022588 | 2.605723797 | 5.81E-07 |
| ENSCLMG00005011543 | 2.596389426 | 1.23E-56 |
| ENSCLMG00005001873 | 2.553389555 | 0.006321258 |
| ENSCLMG00005008867 | 2.54016415 | 0.0019306 |
| ENSCLMG00005006281 | 2.482613169 | 0.03049602 |
| ENSCLMG00005003609 | 2.479701526 | 4.98E-10 |
| ENSCLMG00005002258 | 2.455081239 | 2.23E-19 |
| ENSCLMG00005007273 | 2.441925707 | 0.029012402 |
| ENSCLMG00005006306 | -2.373363483 | 6.91E-29 |
| ENSCLMG00005005864 | 2.34157431 | 1.82E-20 |
| ENSCLMG00005012727 | 2.301596456 | 1.59E-06 |
| ENSCLMG00005005309 | -2.265291649 | 3.06E-07 |
| ENSCLMG00005007189 | 2.247467706 | 5.15E-07 |
| ENSCLMG00005011189 | 2.246075239 | 3.84E-05 |
| ENSCLMG00005001043 | 2.242444787 | 0.001117603 |
| ENSCLMG00005006299 | -2.214305322 | 2.13E-13 |
| ENSCLMG00005011921 | 2.208401928 | 0.007068201 |
| ENSCLMG00005003866 | -2.203900787 | 1.60E-10 |
| ENSCLMG00005021738 | -2.198103213 | 0.001503832 |
| ENSCLMG00005000029 | -2.195810255 | 4.52E-23 |
| ENSCLMG00005014628 | 2.18638367 | 2.61E-07 |
| ENSCLMG00005007096 | 2.1829368 | 4.98E-05 |
| ENSCLMG00005000035 | -2.164274914 | 1.11E-23 |
| ENSCLMG00005012028 | -2.160514959 | 2.47E-20 |
| ENSCLMG00005020274 | -2.159148933 | 3.41E-32 |
| ENSCLMG00005008800 | 2.138917285 | 4.78E-16 |
| ENSCLMG00005011114 | 2.137759674 | 0.000155796 |
| ENSCLMG00005008569 | -2.043941948 | 2.46E-08 |
| ENSCLMG00005013429 | -2.038397805 | 1.06E-08 |
| ENSCLMG00005000447 | -2.025646674 | 1.95E-15 |
| ENSCLMG00005010703 | -2.020976352 | 1.20E-09 |
| ENSCLMG00005010666 | 2.009506364 | 0.004837435 |
| ENSCLMG00005020206 | 1.994213277 | 5.04E-16 |
| ENSCLMG00005014615 | 1.98223345 | 0.041988113 |
| ENSCLMG00005002493 | 1.976133421 | 1.70E-12 |
| ENSCLMG00005010468 | 1.965595244 | 1.01E-11 |
| ENSCLMG00005004333 | 1.963743662 | 0.007068201 |
| ENSCLMG00005018745 | 1.959694006 | 2.75E-08 |
| ENSCLMG00005008853 | -1.947366534 | 0.003909452 |
| ENSCLMG00005019424 | 1.946008817 | 8.18E-05 |
| ENSCLMG00005011538 | 1.936224926 | 4.66E-08 |
| ENSCLMG00005010393 | -1.927302411 | 0.004172632 |
| ENSCLMG00005006548 | 1.924895468 | 3.70E-16 |
| ENSCLMG00005000948 | -1.915531339 | 4.16E-12 |
| ENSCLMG00005023053 | 1.91456466 | 1.13E-08 |
| ENSCLMG00005018959 | -1.907713423 | 0.02898379 |
| ENSCLMG00005002230 | -1.889373811 | 1.19E-30 |
| ENSCLMG00005014809 | 1.874216279 | 3.09E-08 |
| ENSCLMG00005005054 | -1.87301385 | 0.049630198 |
| ENSCLMG00005005053 | -1.869945297 | 6.01E-05 |
| ENSCLMG00005006368 | 1.853524006 | 0.038333258 |
| ENSCLMG00005015653 | 1.852032878 | 1.32E-05 |
| ENSCLMG00005019688 | 1.848757529 | 2.38E-05 |
| ENSCLMG00005014627 | 1.836537076 | 0.034531975 |
| ENSCLMG00005016663 | 1.83379564 | 0.03965787 |
| ENSCLMG00005008908 | -1.833638598 | 1.41E-05 |
| ENSCLMG00005010180 | 1.830587423 | 6.35E-09 |
| ENSCLMG00005002202 | 1.809108397 | 2.29E-05 |
| ENSCLMG00005010745 | -1.794434263 | 0.03965787 |
| ENSCLMG00005013592 | -1.794223303 | 3.55E-11 |
| ENSCLMG00005007521 | 1.789118874 | 7.25E-06 |
| ENSCLMG00005010794 | -1.788403535 | 1.09E-49 |
| ENSCLMG00005020820 | 1.783799663 | 2.32E-10 |
| ENSCLMG00005020785 | 1.771708108 | 0.00046804 |
| ENSCLMG00005004519 | -1.769696668 | 3.42E-33 |
| ENSCLMG00005013433 | 1.767877854 | 0.00645778 |
| ENSCLMG00005011465 | 1.761347673 | 0.036446879 |
| ENSCLMG00005002325 | -1.756801003 | 1.55E-06 |
| ENSCLMG00005006297 | -1.756483605 | 9.00E-06 |
| ENSCLMG00005021280 | -1.742352338 | 0.020130506 |
| ENSCLMG00005013248 | -1.714647433 | 3.23E-27 |
| ENSCLMG00005004947 | 1.710727713 | 3.32E-16 |
| ENSCLMG00005008834 | -1.702586318 | 6.08E-50 |
| ENSCLMG00005016649 | 1.701845755 | 8.38E-06 |
| ENSCLMG00005012191 | -1.700562465 | 1.31E-05 |
| ENSCLMG00005011806 | 1.696793676 | 1.86E-05 |
| ENSCLMG00005020464 | -1.685387722 | 0.017289828 |
| ENSCLMG00005008461 | -1.682918758 | 0.000185908 |
| ENSCLMG00005006358 | -1.676165993 | 1.04E-09 |
| ENSCLMG00005011922 | 1.675237785 | 3.27E-05 |
| ENSCLMG00005010384 | -1.666245855 | 1.68E-06 |
| ENSCLMG00005020912 | 1.6657805 | 0.000205431 |
| ENSCLMG00005022781 | -1.664440386 | 0.000889177 |
| ENSCLMG00005006912 | -1.661476365 | 1.04E-16 |
| ENSCLMG00005017816 | -1.659676703 | 2.19E-05 |
| ENSCLMG00005015707 | -1.658548197 | 0.001602627 |
| ENSCLMG00005016458 | 1.657683964 | 0.041138935 |
| ENSCLMG00005003586 | 1.629176346 | 0.000251953 |
| ENSCLMG00005011306 | -1.627392654 | 5.26E-10 |
| ENSCLMG00005015621 | -1.614684888 | 4.10E-10 |
| ENSCLMG00005004123 | 1.599055784 | 0.013949156 |
| ENSCLMG00005005640 | -1.596927877 | 1.37E-11 |
| ENSCLMG00005019050 | 1.592388194 | 6.06E-11 |
| ENSCLMG00005015694 | -1.592306548 | 4.27E-11 |
| ENSCLMG00005000260 | 1.590621154 | 1.14E-08 |
| ENSCLMG00005011530 | 1.589645345 | 0.000250593 |
| ENSCLMG00005017289 | -1.587899887 | 6.34E-06 |
| ENSCLMG00005014967 | -1.582957308 | 1.13E-18 |
| ENSCLMG00005021340 | -1.580337724 | 0.026304784 |
| ENSCLMG00005007006 | 1.579110145 | 2.38E-05 |
| ENSCLMG00005017225 | 1.578482356 | 0.000388732 |
| ENSCLMG00005010499 | -1.576630667 | 2.00E-11 |
| ENSCLMG00005018786 | 1.575710505 | 3.63E-07 |
| ENSCLMG00005007328 | 1.575372429 | 0.017289828 |
| ENSCLMG00005010553 | -1.573308319 | 1.55E-08 |
| ENSCLMG00005013254 | -1.568207338 | 1.21E-16 |
| ENSCLMG00005007333 | 1.552638559 | 0.000103466 |
| ENSCLMG00005002713 | 1.550707424 | 0.000149735 |
| ENSCLMG00005005204 | -1.548063833 | 4.95E-09 |
| ENSCLMG00005013572 | -1.54001567 | 0.015956277 |
| ENSCLMG00005017332 | 1.539993799 | 6.23E-08 |
| ENSCLMG00005013464 | 1.536064159 | 5.18E-11 |
| ENSCLMG00005005851 | -1.531121861 | 7.09E-05 |
| ENSCLMG00005017857 | 1.530790134 | 0.003956655 |
| ENSCLMG00005008930 | -1.530000481 | 7.06E-31 |
| ENSCLMG00005006624 | -1.529757217 | 1.70E-22 |
| ENSCLMG00005009492 | -1.528859513 | 0.030656877 |
| ENSCLMG00005011190 | 1.525991414 | 0.000142372 |
| ENSCLMG00005014224 | 1.520846089 | 0.014459713 |
| ENSCLMG00005015696 | -1.514589534 | 1.26E-23 |
| ENSCLMG00005015554 | 1.511011148 | 2.72E-08 |
| ENSCLMG00005021035 | -1.510884524 | 6.88E-16 |
| ENSCLMG00005018052 | -1.509326585 | 4.95E-09 |
| ENSCLMG00005017281 | -1.508878282 | 0.016713999 |
| ENSCLMG00005008700 | 1.508571913 | 0.002630182 |
| ENSCLMG00005012330 | 1.502542847 | 0.000477045 |
| ENSCLMG00005001142 | 1.499868362 | 5.90E-38 |
| ENSCLMG00005011799 | -1.49678898 | 9.48E-06 |
| ENSCLMG00005013626 | 1.490163906 | 0.00134533 |
| ENSCLMG00005017209 | -1.4901169 | 7.09E-10 |
| ENSCLMG00005017069 | 1.489867479 | 4.93E-06 |
| ENSCLMG00005011140 | 1.489665738 | 3.56E-05 |
| ENSCLMG00005014519 | 1.488673 | 0.00045698 |
| ENSCLMG00005016290 | -1.483963695 | 1.12E-05 |
| ENSCLMG00005009629 | -1.483491852 | 5.33E-10 |
| ENSCLMG00005012382 | 1.482219083 | 0.002301553 |
| ENSCLMG00005019230 | -1.479031586 | 0.031723693 |
| ENSCLMG00005017832 | -1.47096597 | 8.11E-09 |
| ENSCLMG00005010501 | -1.466273566 | 6.54E-11 |
| ENSCLMG00005016731 | -1.465933049 | 7.62E-07 |
| ENSCLMG00005022364 | 1.465332449 | 0.001463557 |
| ENSCLMG00005020627 | -1.462467904 | 8.47E-19 |
| ENSCLMG00005001743 | -1.461066169 | 0.001139067 |
| ENSCLMG00005017401 | -1.459837235 | 2.35E-10 |
| ENSCLMG00005014745 | -1.45883049 | 3.28E-12 |
| ENSCLMG00005022242 | 1.458650371 | 0.000801189 |
| ENSCLMG00005001818 | 1.456072016 | 4.37E-08 |
| ENSCLMG00005012570 | 1.455294732 | 0.000406906 |
| ENSCLMG00005022149 | 1.448854647 | 1.37E-05 |
| ENSCLMG00005019162 | -1.446067778 | 6.75E-10 |
| ENSCLMG00005009999 | -1.445865182 | 2.38E-06 |
| ENSCLMG00005018101 | 1.445434192 | 0.005719093 |
| ENSCLMG00005022205 | 1.445349832 | 5.87E-11 |
| ENSCLMG00005021455 | -1.444325297 | 0.008095553 |
| ENSCLMG00005002498 | 1.442760799 | 0.013584031 |
| ENSCLMG00005008901 | 1.440341468 | 0.020104871 |
| ENSCLMG00005014178 | -1.436616568 | 1.23E-07 |
| ENSCLMG00005023096 | 1.435571868 | 0.02159915 |
| ENSCLMG00005013808 | 1.431558702 | 0.005624306 |
| ENSCLMG00005017511 | -1.425462197 | 0.00025704 |
| ENSCLMG00005000774 | -1.425191775 | 1.59E-07 |
| ENSCLMG00005013683 | 1.422412182 | 1.82E-05 |
| ENSCLMG00005009954 | -1.419129235 | 3.70E-12 |
| ENSCLMG00005010420 | -1.417278574 | 9.83E-11 |
| ENSCLMG00005012916 | 1.415038045 | 0.008331235 |
| ENSCLMG00005020188 | -1.408301933 | 1.62E-05 |
| ENSCLMG00005008240 | -1.406554841 | 0.000143508 |
| ENSCLMG00005014474 | -1.405298647 | 5.27E-22 |
| ENSCLMG00005016269 | -1.403411695 | 1.08E-05 |
| ENSCLMG00005003496 | -1.403261992 | 0.001364316 |
| ENSCLMG00005004136 | -1.403143911 | 0.017964408 |
| ENSCLMG00005003037 | -1.40153766 | 0.009160455 |
| ENSCLMG00005010855 | 1.397877902 | 1.49E-05 |
| ENSCLMG00005006790 | 1.396530294 | 0.000254912 |
| ENSCLMG00005008807 | 1.395734203 | 0.013036839 |
| ENSCLMG00005016233 | -1.394416341 | 0.001085553 |
| ENSCLMG00005007179 | -1.393438412 | 7.81E-08 |
| ENSCLMG00005011743 | 1.388378076 | 0.008774572 |
| ENSCLMG00005021888 | -1.387876863 | 0.006999995 |
| ENSCLMG00005007168 | -1.387798849 | 8.63E-05 |
| ENSCLMG00005019589 | -1.386701132 | 0.000489962 |
| ENSCLMG00005007620 | -1.386612926 | 7.53E-25 |
| ENSCLMG00005018963 | -1.381515054 | 0.000262396 |
| ENSCLMG00005020488 | -1.381176553 | 3.14E-10 |
| ENSCLMG00005020975 | -1.380335529 | 0.021818192 |
| ENSCLMG00005010949 | -1.376617393 | 2.56E-13 |
| ENSCLMG00005018520 | -1.374810128 | 0.025746078 |
| ENSCLMG00005009114 | -1.373432799 | 1.32E-07 |
| ENSCLMG00005023042 | -1.371701616 | 0.003144 |
| ENSCLMG00005017049 | -1.370908417 | 9.64E-13 |
| ENSCLMG00005004954 | -1.370298844 | 0.008346481 |
| ENSCLMG00005008016 | -1.363814655 | 2.95E-07 |
| ENSCLMG00005014776 | 1.363726347 | 6.38E-05 |
| ENSCLMG00005012662 | 1.356992436 | 2.94E-05 |
| ENSCLMG00005018626 | -1.352135512 | 3.45E-19 |
| ENSCLMG00005005289 | -1.35196398 | 8.11E-09 |
| ENSCLMG00005017156 | -1.350755382 | 0.006059201 |
| ENSCLMG00005010362 | 1.348081306 | 0.013526671 |
| ENSCLMG00005002041 | 1.347468073 | 0.018444996 |
| ENSCLMG00005003659 | -1.343876058 | 0.033973278 |
| ENSCLMG00005022745 | -1.343180888 | 1.76E-54 |
| ENSCLMG00005015369 | -1.340347951 | 0.008117052 |
| ENSCLMG00005017830 | -1.334648742 | 8.48E-06 |
| ENSCLMG00005002505 | -1.33231568 | 0.006521713 |
| ENSCLMG00005002153 | 1.330376544 | 1.62E-40 |
| ENSCLMG00005022511 | -1.32970421 | 2.12E-06 |
| ENSCLMG00005022462 | -1.329484957 | 0.005456562 |
| ENSCLMG00005021834 | 1.329406711 | 1.61E-09 |
| ENSCLMG00005019731 | -1.327926909 | 0.002785826 |
| ENSCLMG00005011138 | 1.326450142 | 0.006012615 |
| ENSCLMG00005021928 | 1.326204889 | 0.000274029 |
| ENSCLMG00005012749 | -1.326081258 | 0.021075874 |
| ENSCLMG00005012671 | -1.32412693 | 0.029166425 |
| ENSCLMG00005016777 | -1.323741914 | 1.03E-12 |
| ENSCLMG00005019487 | 1.321478434 | 0.027300207 |
| ENSCLMG00005015340 | 1.319564826 | 1.93E-07 |
| ENSCLMG00005013100 | -1.31356799 | 5.69E-17 |
| ENSCLMG00005001740 | -1.310751099 | 0.006248249 |
| ENSCLMG00005007092 | -1.310553711 | 5.23E-09 |
| ENSCLMG00005011639 | 1.306795992 | 0.000950546 |
| ENSCLMG00005017470 | -1.306706171 | 0.007201015 |
| ENSCLMG00005021452 | -1.30619809 | 8.41E-06 |
| ENSCLMG00005016592 | -1.300804456 | 2.36E-16 |
| ENSCLMG00005008894 | -1.299922797 | 1.22E-10 |
| ENSCLMG00005016339 | -1.298835132 | 0.002722456 |
| ENSCLMG00005006474 | -1.29592535 | 2.15E-06 |
| ENSCLMG00005017224 | 1.294788255 | 2.90E-07 |
| ENSCLMG00005022380 | -1.294161558 | 1.29E-07 |
| ENSCLMG00005005186 | -1.293820735 | 0.00178627 |
| ENSCLMG00005021303 | -1.293719385 | 7.71E-12 |
| ENSCLMG00005007622 | -1.293691296 | 8.87E-07 |
| ENSCLMG00005009734 | -1.289679283 | 5.26E-07 |
| ENSCLMG00005006636 | 1.285949227 | 0.003963317 |
| ENSCLMG00005020364 | 1.284041412 | 0.00965328 |
| ENSCLMG00005020164 | 1.28318139 | 1.15E-09 |
| ENSCLMG00005013894 | 1.283011037 | 0.006711867 |
| ENSCLMG00005018392 | -1.282015547 | 3.61E-14 |
| ENSCLMG00005003430 | 1.280232747 | 0.000346399 |
| ENSCLMG00005008241 | 1.279014791 | 0.002053691 |
| ENSCLMG00005007821 | -1.273959647 | 1.24E-06 |
| ENSCLMG00005020470 | 1.271386499 | 0.013855702 |
| ENSCLMG00005004298 | -1.271287367 | 8.11E-12 |
| ENSCLMG00005019259 | -1.27066185 | 5.04E-10 |
| ENSCLMG00005015693 | -1.269535637 | 0.010901822 |
| ENSCLMG00005001168 | -1.26894781 | 0.000149735 |
| ENSCLMG00005006687 | 1.267108403 | 8.19E-10 |
| ENSCLMG00005006149 | -1.266538482 | 8.56E-05 |
| ENSCLMG00005006686 | 1.261920494 | 1.28E-07 |
| ENSCLMG00005000743 | 1.261775518 | 0.004203995 |
| ENSCLMG00005015160 | -1.260794152 | 0.006390988 |
| ENSCLMG00005000572 | 1.260676245 | 0.006521713 |
| ENSCLMG00005013868 | -1.259506832 | 2.76E-10 |
| ENSCLMG00005014881 | -1.254814322 | 2.31E-09 |
| ENSCLMG00005004828 | 1.253984283 | 2.38E-06 |
| ENSCLMG00005003924 | 1.253675304 | 0.007621065 |
| ENSCLMG00005004422 | -1.249476958 | 5.22E-05 |
| ENSCLMG00005007373 | -1.249099709 | 4.91E-14 |
| ENSCLMG00005017458 | -1.248493216 | 0.006179908 |
| ENSCLMG00005012500 | -1.246767323 | 0.01824357 |
| ENSCLMG00005019273 | -1.245704796 | 0.008590896 |
| ENSCLMG00005011840 | -1.244242006 | 0.000296654 |
| ENSCLMG00005001899 | 1.241967684 | 0.001024173 |
| ENSCLMG00005001707 | 1.241671786 | 1.93E-07 |
| ENSCLMG00005008328 | 1.241553794 | 0.006890442 |
| ENSCLMG00005011064 | 1.241121405 | 0.001998376 |
| ENSCLMG00005009787 | -1.236610497 | 1.56E-08 |
| ENSCLMG00005010662 | -1.235105404 | 1.25E-14 |
| ENSCLMG00005005791 | 1.2336524 | 0.009089119 |
| ENSCLMG00005014826 | -1.233443576 | 1.29E-06 |
| ENSCLMG00005018936 | -1.232438521 | 9.30E-14 |
| ENSCLMG00005019355 | -1.227958122 | 4.19E-06 |
| ENSCLMG00005009333 | -1.226444137 | 5.58E-10 |
| ENSCLMG00005020358 | -1.222962586 | 3.49E-25 |
| ENSCLMG00005008314 | -1.221848976 | 0.000309437 |
| ENSCLMG00005020602 | -1.220873832 | 3.61E-14 |
| ENSCLMG00005006294 | -1.218537256 | 8.28E-05 |
| ENSCLMG00005008778 | 1.218381558 | 0.000116667 |
| ENSCLMG00005021558 | -1.216710818 | 5.03E-14 |
| ENSCLMG00005021470 | 1.216209905 | 2.62E-10 |
| ENSCLMG00005008864 | 1.214246142 | 4.74E-05 |
| ENSCLMG00005020313 | -1.213522553 | 0.000778861 |
| ENSCLMG00005018683 | 1.212839101 | 0.012053214 |
| ENSCLMG00005022682 | -1.212250137 | 0.000148035 |
| ENSCLMG00005012944 | 1.209931036 | 2.35E-10 |
| ENSCLMG00005002032 | 1.208315765 | 0.011913198 |
| ENSCLMG00005013128 | -1.205297538 | 1.87E-17 |
| ENSCLMG00005018126 | -1.202855411 | 1.68E-05 |
| ENSCLMG00005009165 | 1.202317006 | 0.025264815 |
| ENSCLMG00005001398 | 1.199730365 | 6.35E-13 |
| ENSCLMG00005021113 | -1.198628713 | 1.39E-16 |
| ENSCLMG00005009468 | -1.198010047 | 3.83E-12 |
| ENSCLMG00005012087 | -1.198001862 | 0.037513063 |
| ENSCLMG00005014559 | -1.197539534 | 1.01E-08 |
| ENSCLMG00005001888 | -1.197040734 | 0.000495136 |
| ENSCLMG00005012561 | -1.192008176 | 4.20E-06 |
| ENSCLMG00005016702 | -1.190272362 | 0.040100518 |
| ENSCLMG00005000659 | -1.190267754 | 0.00040096 |
| ENSCLMG00005021375 | -1.190190508 | 7.43E-08 |
| ENSCLMG00005015692 | -1.188740382 | 1.38E-14 |
| ENSCLMG00005000047 | -1.188421293 | 1.82E-06 |
| ENSCLMG00005003189 | -1.184214021 | 0.006460103 |
| ENSCLMG00005022113 | 1.183348163 | 3.35E-05 |
| ENSCLMG00005012285 | -1.181208981 | 0.023628571 |
| ENSCLMG00005010248 | 1.180870218 | 0.005514386 |
| ENSCLMG00005006816 | -1.180532628 | 0.003575421 |
| ENSCLMG00005018853 | -1.176760088 | 1.46E-05 |
| ENSCLMG00005015366 | 1.176523761 | 0.000326859 |
| ENSCLMG00005004984 | -1.176198505 | 1.05E-09 |
| ENSCLMG00005015809 | -1.172277233 | 9.07E-16 |
| ENSCLMG00005002060 | -1.171705763 | 4.46E-05 |
| ENSCLMG00005016330 | -1.171459062 | 3.09E-34 |
| ENSCLMG00005019645 | -1.17085726 | 0.000386918 |
| ENSCLMG00005009039 | -1.170701384 | 0.002008635 |
| ENSCLMG00005013072 | -1.170107902 | 0.045450563 |
| ENSCLMG00005005269 | -1.169431249 | 2.38E-06 |
| ENSCLMG00005018845 | -1.169045993 | 0.00181549 |
| ENSCLMG00005001087 | -1.168828821 | 2.98E-06 |
| ENSCLMG00005020640 | -1.167997338 | 1.10E-07 |
| ENSCLMG00005009881 | 1.166098847 | 0.028373782 |
| ENSCLMG00005000049 | -1.162508288 | 4.45E-18 |
| ENSCLMG00005007235 | -1.161449987 | 0.001998376 |
| ENSCLMG00005021644 | -1.16079224 | 9.67E-08 |
| ENSCLMG00005005949 | -1.160534752 | 0.000429535 |
| ENSCLMG00005007162 | -1.157406467 | 3.21E-05 |
| ENSCLMG00005012313 | -1.156313346 | 0.033564639 |
| ENSCLMG00005002412 | -1.155986418 | 3.12E-05 |
| ENSCLMG00005011407 | -1.155268422 | 0.003071368 |
| ENSCLMG00005018283 | -1.15409987 | 0.009756565 |
| ENSCLMG00005019669 | 1.153607579 | 1.26E-06 |
| ENSCLMG00005008432 | -1.152070648 | 1.33E-19 |
| ENSCLMG00005022038 | -1.150491745 | 4.84E-17 |
| ENSCLMG00005008998 | -1.14860723 | 6.09E-07 |
| ENSCLMG00005012163 | -1.148556267 | 1.91E-09 |
| ENSCLMG00005015339 | 1.148189464 | 3.62E-05 |
| ENSCLMG00005008690 | 1.146262743 | 0.000650596 |
| ENSCLMG00005012822 | -1.145075885 | 0.000128997 |
| ENSCLMG00005008246 | -1.144788739 | 1.05E-23 |
| ENSCLMG00005001331 | -1.144075729 | 5.15E-07 |
| ENSCLMG00005016423 | -1.142561937 | 0.001375927 |
| ENSCLMG00005005697 | -1.141159016 | 0.003122323 |
| ENSCLMG00005013909 | -1.139681819 | 1.20E-10 |
| ENSCLMG00005011441 | -1.138668769 | 0.000141039 |
| ENSCLMG00005013947 | 1.137714335 | 0.03165504 |
| ENSCLMG00005020589 | -1.136236752 | 2.76E-10 |
| ENSCLMG00005021654 | -1.136074529 | 0.016637619 |
| ENSCLMG00005009633 | -1.134255548 | 0.00178627 |
| ENSCLMG00005000847 | -1.132377146 | 0.011647239 |
| ENSCLMG00005010027 | -1.132183545 | 0.000725441 |
| ENSCLMG00005012047 | -1.132067415 | 0.024293906 |
| ENSCLMG00005006128 | -1.129873297 | 8.03E-14 |
| ENSCLMG00005016223 | -1.129265498 | 9.90E-13 |
| ENSCLMG00005000701 | -1.127825789 | 0.001911665 |
| ENSCLMG00005004419 | -1.127719055 | 0.001535167 |
| ENSCLMG00005016528 | -1.127473181 | 0.049372288 |
| ENSCLMG00005006099 | -1.125369256 | 0.004247195 |
| ENSCLMG00005014297 | 1.12520545 | 0.010035447 |
| ENSCLMG00005019545 | -1.12200049 | 8.87E-05 |
| ENSCLMG00005013580 | -1.121698592 | 7.78E-06 |
| ENSCLMG00005015108 | -1.12119007 | 0.000754601 |
| ENSCLMG00005018353 | -1.120899031 | 5.43E-07 |
| ENSCLMG00005011182 | -1.11988832 | 9.38E-16 |
| ENSCLMG00005017410 | -1.118524954 | 1.07E-13 |
| ENSCLMG00005004992 | -1.118069704 | 7.64E-10 |
| ENSCLMG00005001907 | -1.11770567 | 0.00264712 |
| ENSCLMG00005021794 | -1.115979707 | 7.08E-05 |
| ENSCLMG00005022098 | -1.113617236 | 1.08E-06 |
| ENSCLMG00005019998 | -1.113533688 | 5.34E-09 |
| ENSCLMG00005000496 | -1.112567891 | 6.11E-11 |
| ENSCLMG00005019647 | -1.112362031 | 0.007068201 |
| ENSCLMG00005001940 | -1.109748493 | 1.01E-08 |
| ENSCLMG00005012699 | 1.108987223 | 0.03118507 |
| ENSCLMG00005019895 | -1.10870934 | 1.83E-08 |
| ENSCLMG00005000168 | -1.108047002 | 7.93E-08 |
| ENSCLMG00005006897 | -1.107721727 | 0.002512717 |
| ENSCLMG00005010185 | 1.106186249 | 0.000292331 |
| ENSCLMG00005010209 | -1.105620639 | 0.017427764 |
| ENSCLMG00005018707 | -1.105528739 | 0.001647632 |
| ENSCLMG00005018788 | -1.105391658 | 2.27E-16 |
| ENSCLMG00005021414 | -1.105079676 | 0.006549245 |
| ENSCLMG00005007326 | 1.104127963 | 0.002570217 |
| ENSCLMG00005006235 | -1.104109654 | 0.046149746 |
| ENSCLMG00005012129 | -1.100216246 | 0.000722704 |
| ENSCLMG00005013032 | 1.100062246 | 2.47E-07 |
| ENSCLMG00005012335 | 1.098725913 | 3.52E-05 |
| ENSCLMG00005014806 | -1.098118356 | 3.95E-24 |
| ENSCLMG00005008446 | -1.097978665 | 0.000128997 |
| ENSCLMG00005013257 | -1.097392609 | 0.001104777 |
| ENSCLMG00005003088 | -1.096457794 | 1.72E-10 |
| ENSCLMG00005006748 | -1.092362004 | 3.13E-13 |
| ENSCLMG00005020721 | -1.09144032 | 1.97E-18 |
| ENSCLMG00005022529 | -1.090907192 | 8.03E-06 |
| ENSCLMG00005019354 | -1.090263685 | 1.46E-11 |
| ENSCLMG00005003054 | 1.089834923 | 0.034718379 |
| ENSCLMG00005004093 | 1.089296348 | 3.25E-12 |
| ENSCLMG00005014625 | -1.088261982 | 2.46E-09 |
| ENSCLMG00005018619 | -1.08793634 | 0.000445326 |
| ENSCLMG00005011867 | -1.085201914 | 2.47E-18 |
| ENSCLMG00005020890 | 1.084824557 | 0.001631367 |
| ENSCLMG00005008854 | -1.084795014 | 0.000621328 |
| ENSCLMG00005003201 | 1.084175463 | 2.56E-05 |
| ENSCLMG00005000720 | -1.083440081 | 6.90E-05 |
| ENSCLMG00005018682 | 1.082931999 | 0.023454063 |
| ENSCLMG00005012454 | -1.0822422 | 1.90E-08 |
| ENSCLMG00005015892 | -1.079220072 | 1.34E-19 |
| ENSCLMG00005015649 | -1.078966594 | 1.71E-22 |
| ENSCLMG00005020075 | -1.075850801 | 0.00069443 |
| ENSCLMG00005013856 | -1.073889146 | 1.65E-08 |
| ENSCLMG00005002524 | -1.073050562 | 2.51E-49 |
| ENSCLMG00005007504 | -1.072959308 | 3.36E-05 |
| ENSCLMG00005013521 | -1.07088029 | 2.33E-14 |
| ENSCLMG00005004895 | 1.070609802 | 2.06E-05 |
| ENSCLMG00005003304 | -1.070575826 | 0.000516984 |
| ENSCLMG00005011137 | -1.068907252 | 1.23E-07 |
| ENSCLMG00005017287 | -1.068717685 | 1.53E-06 |
| ENSCLMG00005011106 | 1.065855257 | 0.008448291 |
| ENSCLMG00005011304 | 1.06405474 | 0.006227042 |
| ENSCLMG00005015654 | -1.062178153 | 0.004667727 |
| ENSCLMG00005011775 | -1.061603649 | 1.68E-08 |
| ENSCLMG00005000163 | 1.061566466 | 0.002442903 |
| ENSCLMG00005012629 | 1.060631463 | 0.031899132 |
| ENSCLMG00005020224 | -1.060217522 | 0.008512251 |
| ENSCLMG00005011573 | -1.059673267 | 0.001161607 |
| ENSCLMG00005001543 | -1.058721854 | 3.34E-11 |
| ENSCLMG00005005837 | 1.057978019 | 0.000178245 |
| ENSCLMG00005000848 | -1.057868856 | 0.00081775 |
| ENSCLMG00005000644 | -1.05753548 | 2.60E-05 |
| ENSCLMG00005000713 | -1.056123347 | 9.45E-11 |
| ENSCLMG00005009886 | -1.055071019 | 2.92E-08 |
| ENSCLMG00005021886 | -1.054483887 | 4.95E-09 |
| ENSCLMG00005016135 | -1.052426686 | 0.000178311 |
| ENSCLMG00005021066 | 1.052244027 | 0.00151452 |
| ENSCLMG00005003636 | 1.051146394 | 0.0188233 |
| ENSCLMG00005017598 | -1.050630952 | 0.028892052 |
| ENSCLMG00005021226 | -1.047875767 | 1.22E-11 |
| ENSCLMG00005015569 | -1.047309828 | 0.000128703 |
| ENSCLMG00005010042 | -1.046903632 | 1.29E-15 |
| ENSCLMG00005013380 | -1.04447106 | 0.001098516 |
| ENSCLMG00005020652 | -1.044165845 | 2.78E-13 |
| ENSCLMG00005011149 | 1.044089207 | 4.81E-06 |
| ENSCLMG00005007892 | 1.042436579 | 1.89E-08 |
| ENSCLMG00005002492 | -1.041680941 | 4.35E-06 |
| ENSCLMG00005002394 | -1.041254478 | 0.003649883 |
| ENSCLMG00005005104 | 1.040797689 | 0.003575421 |
| ENSCLMG00005002557 | 1.039321999 | 1.28E-05 |
| ENSCLMG00005013031 | 1.037068393 | 0.003300919 |
| ENSCLMG00005014488 | 1.034804171 | 0.002106318 |
| ENSCLMG00005015635 | 1.034349172 | 0.016951091 |
| ENSCLMG00005003219 | -1.033862641 | 0.000268842 |
| ENSCLMG00005016914 | -1.033811098 | 0.005499998 |
| ENSCLMG00005022526 | -1.033746555 | 0.001406045 |
| ENSCLMG00005022562 | -1.033389278 | 0.003706558 |
| ENSCLMG00005004176 | -1.031665678 | 2.34E-09 |
| ENSCLMG00005000214 | -1.030699784 | 3.36E-13 |
| ENSCLMG00005005026 | -1.029484864 | 4.75E-07 |
| ENSCLMG00005004892 | -1.027591407 | 2.31E-12 |
| ENSCLMG00005009885 | -1.026697733 | 0.003921115 |
| ENSCLMG00005015917 | -1.026230414 | 0.022404846 |
| ENSCLMG00005016876 | -1.02587187 | 0.048539562 |
| ENSCLMG00005018555 | -1.024928179 | 8.42E-17 |
| ENSCLMG00005002615 | -1.024620849 | 5.70E-08 |
| ENSCLMG00005001510 | 1.023360789 | 0.01090294 |
| ENSCLMG00005012892 | -1.022089717 | 2.62E-10 |
| ENSCLMG00005012742 | 1.021989359 | 0.009762711 |
| ENSCLMG00005020165 | 1.018319112 | 0.000261839 |
| ENSCLMG00005010569 | -1.016475724 | 0.000171659 |
| ENSCLMG00005000344 | 1.014361163 | 0.005966723 |
| ENSCLMG00005011085 | -1.012832525 | 0.015106873 |
| ENSCLMG00005002013 | -1.012020839 | 0.004356358 |
| ENSCLMG00005006372 | -1.010780167 | 9.78E-09 |
| ENSCLMG00005004349 | -1.009344392 | 0.014354131 |
| ENSCLMG00005020739 | -1.008753694 | 2.38E-05 |
| ENSCLMG00005010593 | 1.007209159 | 0.019708461 |
| ENSCLMG00005017981 | -1.004988079 | 0.000328977 |
| ENSCLMG00005021804 | 1.003837386 | 0.013813669 |
| ENSCLMG00005015326 | -1.003141373 | 2.71E-05 |
| ENSCLMG00005000892 | -1.001675805 | 1.14E-06 |
| ENSCLMG00005000557 | 1.00063335 | 0.008831315 |
| ENSCLMG00005005193 | -1.000512763 | 2.60E-07 |
| ENSCLMG00005000053 | -0.999570212 | 0.000106759 |
| ENSCLMG00005002511 | -0.998236533 | 0.00765609 |
| ENSCLMG00005012941 | -0.998120685 | 0.024273393 |
| ENSCLMG00005022870 | -0.997194523 | 3.61E-08 |
| ENSCLMG00005010500 | -0.996456388 | 6.03E-07 |
| ENSCLMG00005015395 | -0.995711934 | 9.77E-06 |
| ENSCLMG00005019088 | -0.995422113 | 0.005163994 |
| ENSCLMG00005009721 | -0.994538122 | 1.91E-05 |
| ENSCLMG00005001904 | -0.993382835 | 0.016307151 |
| ENSCLMG00005010044 | -0.99214622 | 0.004796718 |
| ENSCLMG00005006609 | -0.991311878 | 4.43E-06 |
| ENSCLMG00005010978 | -0.989037406 | 3.87E-07 |
| ENSCLMG00005010618 | -0.988470953 | 8.11E-09 |
| ENSCLMG00005015785 | -0.98717788 | 9.07E-05 |
| ENSCLMG00005018848 | -0.985555108 | 0.000329466 |
| ENSCLMG00005013333 | -0.984901018 | 0.005590204 |
| ENSCLMG00005015699 | -0.984632478 | 0.0092141 |
| ENSCLMG00005019624 | -0.983540331 | 0.004356358 |
| ENSCLMG00005016399 | 0.983364273 | 0.012478244 |
| ENSCLMG00005017834 | 0.983165976 | 0.015161328 |
| ENSCLMG00005011563 | 0.982669267 | 6.55E-05 |
| ENSCLMG00005009037 | 0.981476609 | 7.75E-05 |
| ENSCLMG00005021199 | -0.980725242 | 3.14E-05 |
| ENSCLMG00005005274 | -0.980348445 | 0.032286403 |
| ENSCLMG00005014295 | -0.980275397 | 0.000262228 |
| ENSCLMG00005022214 | -0.980069303 | 1.72E-08 |
| ENSCLMG00005005941 | -0.978506618 | 0.002768731 |
| ENSCLMG00005006870 | -0.978001378 | 6.01E-05 |
| ENSCLMG00005008216 | -0.977822042 | 9.55E-05 |
| ENSCLMG00005011923 | 0.977794112 | 0.010052302 |
| ENSCLMG00005006041 | -0.977757522 | 9.00E-13 |
| ENSCLMG00005013512 | -0.977037528 | 2.46E-08 |
| ENSCLMG00005018202 | -0.97694088 | 0.004274377 |
| ENSCLMG00005010463 | -0.976582512 | 0.011647239 |
| ENSCLMG00005014420 | -0.974773537 | 6.85E-12 |
| ENSCLMG00005011526 | 0.971472336 | 0.035427721 |
| ENSCLMG00005015336 | -0.97132983 | 0.001432139 |
| ENSCLMG00005019063 | -0.970927331 | 4.84E-07 |
| ENSCLMG00005010239 | 0.970059593 | 0.000108586 |
| ENSCLMG00005014955 | -0.968966255 | 0.000667342 |
| ENSCLMG00005019945 | -0.968966014 | 3.36E-11 |
| ENSCLMG00005014286 | -0.968365186 | 0.015339325 |
| ENSCLMG00005006988 | -0.967133733 | 3.64E-11 |
| ENSCLMG00005009415 | -0.966449411 | 0.002391566 |
| ENSCLMG00005002434 | -0.966439682 | 3.86E-05 |
| ENSCLMG00005019627 | -0.96496835 | 0.011781567 |
| ENSCLMG00005006494 | -0.964068859 | 1.17E-09 |
| ENSCLMG00005021756 | -0.963186921 | 1.51E-11 |
| ENSCLMG00005012284 | -0.962576842 | 4.58E-07 |
| ENSCLMG00005019972 | 0.961511827 | 1.12E-05 |
| ENSCLMG00005016293 | -0.960887302 | 0.020391819 |
| ENSCLMG00005013256 | -0.959825316 | 9.77E-06 |
| ENSCLMG00005012663 | 0.959736318 | 7.85E-08 |
| ENSCLMG00005005085 | 0.958451697 | 0.03440195 |
| ENSCLMG00005002226 | -0.958263559 | 6.91E-09 |
| ENSCLMG00005017576 | -0.956277728 | 7.81E-08 |
| ENSCLMG00005007934 | -0.955478694 | 3.91E-06 |
| ENSCLMG00005002037 | -0.952980529 | 2.61E-09 |
| ENSCLMG00005013101 | -0.951270694 | 0.000445326 |
| ENSCLMG00005022960 | -0.951204049 | 0.000287311 |
| ENSCLMG00005006036 | -0.950847311 | 9.42E-05 |
| ENSCLMG00005005943 | -0.950675448 | 0.001328511 |
| ENSCLMG00005004982 | -0.949901459 | 0.004356358 |
| ENSCLMG00005010698 | -0.949231387 | 2.27E-07 |
| ENSCLMG00005020490 | -0.948718269 | 3.69E-05 |
| ENSCLMG00005002464 | -0.947877965 | 1.01E-12 |
| ENSCLMG00005012453 | -0.947606098 | 2.24E-12 |
| ENSCLMG00005018099 | 0.947395652 | 0.004576595 |
| ENSCLMG00005017132 | -0.946840547 | 0.002219796 |
| ENSCLMG00005010280 | -0.946057002 | 4.32E-07 |
| ENSCLMG00005004744 | 0.945522543 | 0.005888951 |
| ENSCLMG00005017601 | -0.944626936 | 7.78E-09 |
| ENSCLMG00005017639 | -0.943846416 | 0.001277525 |
| ENSCLMG00005016228 | -0.943616034 | 1.01E-11 |
| ENSCLMG00005006574 | -0.942185698 | 0.000298488 |
| ENSCLMG00005011586 | 0.939875004 | 0.020104871 |
| ENSCLMG00005014299 | -0.939575873 | 0.037513063 |
| ENSCLMG00005005378 | -0.939549679 | 0.016672444 |
| ENSCLMG00005002729 | -0.937244118 | 2.24E-12 |
| ENSCLMG00005020889 | -0.936649088 | 1.07E-05 |
| ENSCLMG00005012847 | -0.936116908 | 0.00229621 |
| ENSCLMG00005010428 | -0.935820333 | 0.003973983 |
| ENSCLMG00005013273 | -0.935713626 | 2.00E-11 |
| ENSCLMG00005008966 | -0.934594485 | 0.000227667 |
| ENSCLMG00005000559 | -0.934435201 | 1.60E-09 |
| ENSCLMG00005020747 | -0.934413496 | 2.31E-08 |
| ENSCLMG00005005154 | -0.934404912 | 0.000173764 |
| ENSCLMG00005005185 | -0.934176419 | 3.52E-11 |
| ENSCLMG00005019754 | -0.934078581 | 0.012977415 |
| ENSCLMG00005007940 | -0.933736386 | 8.87E-07 |
| ENSCLMG00005019766 | -0.933402093 | 0.005456562 |
| ENSCLMG00005018870 | -0.929823594 | 7.35E-06 |
| ENSCLMG00005016396 | -0.929704048 | 9.77E-09 |
| ENSCLMG00005019881 | -0.929446067 | 0.002774787 |
| ENSCLMG00005010942 | 0.927822696 | 0.000611413 |
| ENSCLMG00005011968 | -0.927693406 | 0.002383965 |
| ENSCLMG00005022243 | 0.926970835 | 0.025463421 |
| ENSCLMG00005006584 | -0.925040931 | 0.006521713 |
| ENSCLMG00005020220 | -0.923668742 | 1.01E-11 |
| ENSCLMG00005020256 | -0.922650046 | 0.033195783 |
| ENSCLMG00005014743 | -0.922443459 | 0.017978766 |
| ENSCLMG00005015598 | -0.922439506 | 1.09E-05 |
| ENSCLMG00005003079 | -0.920674692 | 9.92E-07 |
| ENSCLMG00005003612 | -0.919494013 | 0.000446725 |
| ENSCLMG00005020366 | -0.918470514 | 0.008448291 |
| ENSCLMG00005013703 | -0.918443173 | 3.10E-10 |
| ENSCLMG00005011547 | -0.91804489 | 4.61E-05 |
| ENSCLMG00005004111 | 0.917470965 | 4.50E-07 |
| ENSCLMG00005021314 | 0.917303149 | 0.001901819 |
| ENSCLMG00005007562 | -0.915652033 | 5.53E-15 |
| ENSCLMG00005009083 | -0.915357163 | 2.31E-11 |
| ENSCLMG00005022603 | -0.915351103 | 8.78E-06 |
| ENSCLMG00005021809 | -0.91528163 | 0.000495136 |
| ENSCLMG00005012874 | -0.91348481 | 0.006906751 |
| ENSCLMG00005003888 | -0.912714606 | 0.009727339 |
| ENSCLMG00005012790 | -0.91105654 | 2.88E-10 |
| ENSCLMG00005014520 | 0.911040686 | 0.040529542 |
| ENSCLMG00005020007 | -0.910206885 | 2.78E-15 |
| ENSCLMG00005021887 | -0.909983793 | 3.83E-12 |
| ENSCLMG00005004068 | 0.907228294 | 0.001554474 |
| ENSCLMG00005005360 | -0.906982125 | 3.13E-08 |
| ENSCLMG00005003770 | 0.905155569 | 7.21E-10 |
| ENSCLMG00005003242 | 0.904861033 | 0.029012402 |
| ENSCLMG00005009974 | -0.904572229 | 1.17E-05 |
| ENSCLMG00005010704 | -0.903946745 | 0.004354633 |
| ENSCLMG00005009925 | -0.903283384 | 2.89E-08 |
| ENSCLMG00005001218 | -0.902448308 | 0.007068201 |
| ENSCLMG00005010544 | -0.902338464 | 7.87E-06 |
| ENSCLMG00005020277 | -0.90034896 | 0.003194574 |
| ENSCLMG00005016698 | -0.900225679 | 0.000714646 |
| ENSCLMG00005012628 | -0.899548148 | 0.030381134 |
| ENSCLMG00005021898 | -0.899512367 | 4.86E-07 |
| ENSCLMG00005000455 | -0.898785485 | 5.06E-08 |
| ENSCLMG00005002437 | -0.896861849 | 0.01882421 |
| ENSCLMG00005000010 | -0.89632414 | 1.23E-11 |
| ENSCLMG00005002316 | -0.895828419 | 1.23E-07 |
| ENSCLMG00005022086 | -0.894690362 | 7.35E-06 |
| ENSCLMG00005006540 | -0.894512518 | 3.62E-11 |
| ENSCLMG00005007056 | -0.894111035 | 1.91E-11 |
| ENSCLMG00005000867 | -0.893706276 | 0.003300919 |
| ENSCLMG00005006658 | 0.892436627 | 9.00E-06 |
| ENSCLMG00005018200 | -0.892102536 | 3.66E-05 |
| ENSCLMG00005001117 | -0.89195236 | 0.000158176 |
| ENSCLMG00005015021 | -0.891895868 | 7.67E-05 |
| ENSCLMG00005017865 | -0.891840493 | 0.003345271 |
| ENSCLMG00005012098 | 0.891616926 | 0.032419119 |
| ENSCLMG00005003437 | -0.890835529 | 0.005920843 |
| ENSCLMG00005021892 | -0.890828892 | 0.000280659 |
| ENSCLMG00005007691 | -0.890823202 | 0.00510779 |
| ENSCLMG00005003514 | -0.888002553 | 2.33E-07 |
| ENSCLMG00005022647 | 0.887211215 | 0.039702214 |
| ENSCLMG00005001963 | -0.886584793 | 0.016675809 |
| ENSCLMG00005012006 | 0.88625777 | 0.016504002 |
| ENSCLMG00005005105 | -0.88505832 | 1.09E-08 |
| ENSCLMG00005000008 | 0.885013361 | 0.016094382 |
| ENSCLMG00005011559 | 0.884837853 | 0.044543371 |
| ENSCLMG00005019376 | -0.883575234 | 5.04E-13 |
| ENSCLMG00005020192 | -0.883417073 | 4.61E-05 |
| ENSCLMG00005010792 | 0.883221793 | 0.012936357 |
| ENSCLMG00005014242 | -0.87994011 | 0.006806073 |
| ENSCLMG00005022223 | -0.877797637 | 0.000260482 |
| ENSCLMG00005013566 | -0.877376888 | 2.01E-06 |
| ENSCLMG00005000745 | -0.87610899 | 1.35E-07 |
| ENSCLMG00005004090 | -0.87534808 | 2.03E-05 |
| ENSCLMG00005008577 | 0.874686672 | 0.000113825 |
| ENSCLMG00005003292 | -0.874483104 | 0.001385789 |
| ENSCLMG00005012538 | -0.872580226 | 8.95E-10 |
| ENSCLMG00005008919 | -0.872566952 | 9.89E-13 |
| ENSCLMG00005005184 | -0.870759865 | 2.08E-08 |
| ENSCLMG00005014976 | 0.870701889 | 0.019296976 |
| ENSCLMG00005002319 | 0.869161987 | 0.000549707 |
| ENSCLMG00005013704 | -0.868444645 | 0.000430727 |
| ENSCLMG00005003287 | -0.867905 | 8.56E-07 |
| ENSCLMG00005022220 | -0.86749466 | 0.001478119 |
| ENSCLMG00005002085 | -0.865545089 | 0.004488034 |
| ENSCLMG00005003454 | 0.863669668 | 0.007895242 |
| ENSCLMG00005020804 | -0.863108189 | 0.00012605 |
| ENSCLMG00005015459 | -0.862656396 | 0.006890442 |
| ENSCLMG00005017945 | -0.8622391 | 0.005904351 |
| ENSCLMG00005019356 | -0.861883768 | 0.00478448 |
| ENSCLMG00005020913 | 0.860987857 | 0.014511219 |
| ENSCLMG00005015202 | -0.860144439 | 0.005127476 |
| ENSCLMG00005022868 | -0.860009615 | 0.004733225 |
| ENSCLMG00005007995 | 0.859940255 | 0.041704017 |
| ENSCLMG00005004906 | 0.857454253 | 0.010717486 |
| ENSCLMG00005020722 | -0.855792956 | 9.66E-11 |
| ENSCLMG00005021668 | -0.855682207 | 0.004982602 |
| ENSCLMG00005005781 | -0.855649429 | 0.006340988 |
| ENSCLMG00005010786 | -0.855465321 | 0.002936046 |
| ENSCLMG00005019486 | 0.854897029 | 0.000114003 |
| ENSCLMG00005015486 | 0.854887758 | 0.006497468 |
| ENSCLMG00005017915 | -0.854881217 | 7.30E-09 |
| ENSCLMG00005003212 | -0.854729787 | 0.000900014 |
| ENSCLMG00005020742 | -0.854195885 | 1.17E-07 |
| ENSCLMG00005003459 | -0.852703486 | 2.82E-07 |
| ENSCLMG00005008252 | -0.852105966 | 9.82E-07 |
| ENSCLMG00005021559 | -0.849386525 | 3.52E-20 |
| ENSCLMG00005012219 | -0.848032337 | 2.67E-05 |
| ENSCLMG00005016046 | -0.847365203 | 0.006518149 |
| ENSCLMG00005022065 | -0.846795795 | 1.34E-08 |
| ENSCLMG00005008706 | 0.845051791 | 0.044584981 |
| ENSCLMG00005007220 | -0.844775088 | 0.021633782 |
| ENSCLMG00005002066 | 0.844629117 | 0.006251894 |
| ENSCLMG00005019480 | 0.844055802 | 0.047561488 |
| ENSCLMG00005020346 | -0.842922257 | 0.000148169 |
| ENSCLMG00005005136 | -0.842785121 | 0.000132757 |
| ENSCLMG00005020767 | -0.842720079 | 0.002264368 |
| ENSCLMG00005001703 | -0.84253669 | 0.029450418 |
| ENSCLMG00005018136 | -0.841878203 | 0.000138111 |
| ENSCLMG00005012986 | -0.841007701 | 2.82E-07 |
| ENSCLMG00005005202 | -0.840952125 | 1.87E-09 |
| ENSCLMG00005011175 | -0.840428 | 0.007993267 |
| ENSCLMG00005012273 | -0.839755655 | 0.000227667 |
| ENSCLMG00005003426 | 0.83846963 | 0.003435905 |
| ENSCLMG00005003956 | -0.837631245 | 8.71E-12 |
| ENSCLMG00005021761 | -0.835538625 | 2.37E-19 |
| ENSCLMG00005009597 | -0.835431817 | 2.51E-15 |
| ENSCLMG00005022579 | -0.835089014 | 0.020330291 |
| ENSCLMG00005013457 | 0.834688797 | 0.049654546 |
| ENSCLMG00005010757 | -0.83420523 | 0.003110767 |
| ENSCLMG00005020519 | -0.833625774 | 6.47E-05 |
| ENSCLMG00005017861 | -0.830920893 | 0.010900036 |
| ENSCLMG00005016638 | -0.829788882 | 2.52E-07 |
| ENSCLMG00005014951 | -0.82879942 | 0.000262887 |
| ENSCLMG00005014451 | -0.828786961 | 4.66E-05 |
| ENSCLMG00005005000 | -0.828622233 | 1.80E-05 |
| ENSCLMG00005005301 | -0.827959815 | 0.002390689 |
| ENSCLMG00005010024 | -0.827918468 | 0.000757543 |
| ENSCLMG00005004608 | -0.826514496 | 0.005094909 |
| ENSCLMG00005013682 | 0.825326938 | 0.006251322 |
| ENSCLMG00005001490 | -0.8245592 | 3.78E-06 |
| ENSCLMG00005011641 | 0.824111562 | 0.04286351 |
| ENSCLMG00005009928 | -0.823944367 | 0.000661967 |
| ENSCLMG00005010736 | -0.822721162 | 0.000511243 |
| ENSCLMG00005007178 | -0.822131413 | 5.51E-11 |
| ENSCLMG00005004240 | -0.820452828 | 7.99E-05 |
| ENSCLMG00005014658 | -0.820324545 | 0.011282858 |
| ENSCLMG00005013807 | -0.817993087 | 0.000539785 |
| ENSCLMG00005004931 | -0.817437826 | 0.001144084 |
| ENSCLMG00005015231 | -0.816720163 | 0.00178627 |
| ENSCLMG00005015473 | 0.815307684 | 0.034437154 |
| ENSCLMG00005014445 | -0.814043381 | 0.022404846 |
| ENSCLMG00005015776 | -0.812770836 | 0.006321258 |
| ENSCLMG00005010826 | -0.812425335 | 0.042886982 |
| ENSCLMG00005005288 | -0.812364062 | 0.042612349 |
| ENSCLMG00005020774 | -0.812225244 | 0.044085972 |
| ENSCLMG00005014990 | -0.811903392 | 0.033058257 |
| ENSCLMG00005000865 | -0.811801839 | 0.002944519 |
| ENSCLMG00005003193 | -0.811701727 | 0.038345745 |
| ENSCLMG00005009594 | -0.811689542 | 3.19E-06 |
| ENSCLMG00005020888 | 0.809325244 | 0.000402332 |
| ENSCLMG00005019490 | -0.80901084 | 0.00424301 |
| ENSCLMG00005009372 | 0.808880701 | 0.009759052 |
| ENSCLMG00005015129 | -0.808298837 | 0.014896913 |
| ENSCLMG00005011558 | 0.807010915 | 0.022274246 |
| ENSCLMG00005016064 | -0.806030169 | 0.040444226 |
| ENSCLMG00005012020 | -0.805224301 | 0.002654972 |
| ENSCLMG00005017528 | -0.804450349 | 3.62E-06 |
| ENSCLMG00005003033 | -0.804275682 | 7.35E-06 |
| ENSCLMG00005002059 | -0.803759934 | 0.024536647 |
| ENSCLMG00005018428 | -0.801853565 | 0.019387899 |
| ENSCLMG00005007453 | 0.80171212 | 3.30E-05 |
| ENSCLMG00005018042 | 0.800727847 | 0.004412434 |
| ENSCLMG00005009997 | -0.799373314 | 0.007363568 |
| ENSCLMG00005010390 | -0.798827845 | 0.002263335 |
| ENSCLMG00005018187 | 0.79745886 | 0.002072497 |
| ENSCLMG00005009181 | -0.797413327 | 0.031369136 |
| ENSCLMG00005020393 | -0.797147431 | 3.05E-09 |
| ENSCLMG00005006463 | -0.797030627 | 2.79E-05 |
| ENSCLMG00005001084 | -0.796984649 | 1.01E-11 |
| ENSCLMG00005022067 | -0.796464998 | 1.57E-07 |
| ENSCLMG00005010877 | -0.794675888 | 0.003241348 |
| ENSCLMG00005015128 | 0.794177968 | 0.015052951 |
| ENSCLMG00005016950 | -0.793835666 | 2.59E-06 |
| ENSCLMG00005009666 | -0.793246955 | 1.75E-06 |
| ENSCLMG00005014730 | -0.792778055 | 6.15E-08 |
| ENSCLMG00005022468 | 0.792530126 | 0.000635722 |
| ENSCLMG00005014413 | -0.792437535 | 1.51E-06 |
| ENSCLMG00005017643 | -0.792103883 | 0.002225097 |
| ENSCLMG00005000499 | -0.790651572 | 6.09E-08 |
| ENSCLMG00005008336 | -0.790096812 | 0.003832349 |
| ENSCLMG00005011438 | -0.789861255 | 2.41E-05 |
| ENSCLMG00005016127 | -0.789599954 | 2.84E-06 |
| ENSCLMG00005003878 | -0.789384342 | 0.009560267 |
| ENSCLMG00005004894 | -0.788921835 | 8.94E-06 |
| ENSCLMG00005012035 | 0.788390835 | 0.031591918 |
| ENSCLMG00005001677 | -0.787924569 | 0.00199482 |
| ENSCLMG00005016527 | -0.787163593 | 0.020621751 |
| ENSCLMG00005015532 | -0.786381317 | 4.86E-05 |
| ENSCLMG00005006613 | -0.785199679 | 1.89E-08 |
| ENSCLMG00005007563 | -0.784797029 | 0.000688818 |
| ENSCLMG00005011632 | -0.783809557 | 0.000227315 |
| ENSCLMG00005010144 | -0.782887376 | 0.00608941 |
| ENSCLMG00005004927 | -0.782867274 | 1.71E-07 |
| ENSCLMG00005000614 | -0.781695622 | 0.008346481 |
| ENSCLMG00005000221 | -0.781025219 | 3.07E-05 |
| ENSCLMG00005019116 | -0.780006265 | 0.007270833 |
| ENSCLMG00005011233 | 0.778981614 | 0.013360124 |
| ENSCLMG00005020177 | 0.778893898 | 0.015380873 |
| ENSCLMG00005006706 | -0.778356938 | 1.83E-08 |
| ENSCLMG00005018122 | -0.777501896 | 1.41E-07 |
| ENSCLMG00005000776 | -0.776796001 | 0.012542899 |
| ENSCLMG00005002654 | 0.775587894 | 0.001428854 |
| ENSCLMG00005023017 | -0.77347143 | 9.68E-05 |
| ENSCLMG00005021798 | 0.771950787 | 0.007194945 |
| ENSCLMG00005003028 | -0.771063761 | 0.041821106 |
| ENSCLMG00005007097 | -0.769975147 | 1.18E-06 |
| ENSCLMG00005000682 | -0.76941069 | 0.003300919 |
| ENSCLMG00005021922 | -0.768120852 | 0.000559183 |
| ENSCLMG00005018910 | -0.768064957 | 3.99E-10 |
| ENSCLMG00005004997 | 0.767159271 | 0.043404871 |
| ENSCLMG00005016803 | -0.767107928 | 0.027816166 |
| ENSCLMG00005007491 | -0.766167504 | 0.035427721 |
| ENSCLMG00005008713 | 0.765423136 | 0.000423206 |
| ENSCLMG00005013530 | -0.765366765 | 0.001440444 |
| ENSCLMG00005021186 | -0.765207614 | 0.030326682 |
| ENSCLMG00005008952 | -0.763958124 | 0.004887391 |
| ENSCLMG00005017334 | -0.763667782 | 0.001051542 |
| ENSCLMG00005018670 | -0.763526589 | 0.028285397 |
| ENSCLMG00005016475 | -0.763021375 | 0.001721914 |
| ENSCLMG00005007094 | 0.76193806 | 0.012797624 |
| ENSCLMG00005016424 | 0.761168851 | 0.049617191 |
| ENSCLMG00005019713 | 0.760956527 | 0.001655802 |
| ENSCLMG00005011289 | -0.76008453 | 0.008590896 |
| ENSCLMG00005013270 | -0.760036984 | 3.17E-10 |
| ENSCLMG00005018831 | -0.759789168 | 0.00089054 |
| ENSCLMG00005019530 | -0.756214291 | 0.028492101 |
| ENSCLMG00005021386 | -0.755892088 | 0.001382314 |
| ENSCLMG00005015934 | -0.752091352 | 9.53E-06 |
| ENSCLMG00005011167 | -0.752038278 | 4.22E-07 |
| ENSCLMG00005004008 | -0.750817799 | 0.003285948 |
| ENSCLMG00005007123 | 0.750352133 | 1.91E-05 |
| ENSCLMG00005006078 | -0.749747889 | 7.13E-09 |
| ENSCLMG00005002197 | 0.749416158 | 0.028952827 |
| ENSCLMG00005022591 | -0.749273183 | 0.04126427 |
| ENSCLMG00005003159 | 0.748029893 | 0.000550515 |
| ENSCLMG00005009727 | -0.747707365 | 0.007068201 |
| ENSCLMG00005009896 | -0.747343534 | 0.013228516 |
| ENSCLMG00005000666 | -0.746836258 | 0.002832535 |
| ENSCLMG00005017734 | -0.746422493 | 4.37E-09 |
| ENSCLMG00005002136 | -0.746289145 | 0.004633072 |
| ENSCLMG00005008781 | 0.745354838 | 1.17E-06 |
| ENSCLMG00005008209 | -0.745305648 | 0.001061764 |
| ENSCLMG00005013922 | -0.744275931 | 0.013891068 |
| ENSCLMG00005000993 | -0.743156151 | 0.003729175 |
| ENSCLMG00005006773 | -0.742782378 | 0.049231128 |
| ENSCLMG00005006090 | -0.742027306 | 0.000716176 |
| ENSCLMG00005011063 | 0.741979368 | 0.004088674 |
| ENSCLMG00005010763 | 0.741904752 | 0.046039038 |
| ENSCLMG00005016406 | -0.741496 | 0.005175819 |
| ENSCLMG00005021019 | -0.741190287 | 0.037298918 |
| ENSCLMG00005016063 | -0.74094675 | 0.000780777 |
| ENSCLMG00005001869 | -0.739609161 | 0.004356358 |
| ENSCLMG00005008242 | -0.738947432 | 0.001528832 |
| ENSCLMG00005005506 | -0.738556162 | 9.82E-07 |
| ENSCLMG00005015503 | -0.738182591 | 8.82E-10 |
| ENSCLMG00005000415 | -0.737937564 | 3.86E-05 |
| ENSCLMG00005003850 | -0.73759132 | 0.005337434 |
| ENSCLMG00005009781 | 0.737539197 | 0.022119628 |
| ENSCLMG00005002660 | 0.737337756 | 0.006873977 |
| ENSCLMG00005021610 | -0.736848143 | 0.005143734 |
| ENSCLMG00005019258 | -0.735158807 | 0.006918996 |
| ENSCLMG00005000150 | -0.734686064 | 0.007215849 |
| ENSCLMG00005007358 | 0.734550212 | 0.047128007 |
| ENSCLMG00005021506 | -0.733856895 | 0.007621834 |
| ENSCLMG00005000140 | -0.733448694 | 0.001991337 |
| ENSCLMG00005023036 | -0.732943727 | 0.009934409 |
| ENSCLMG00005018036 | -0.732745329 | 0.004661312 |
| ENSCLMG00005020754 | -0.732607307 | 0.007559437 |
| ENSCLMG00005015806 | -0.729458882 | 0.030260218 |
| ENSCLMG00005011747 | -0.728789197 | 8.89E-06 |
| ENSCLMG00005020586 | -0.728768088 | 2.76E-05 |
| ENSCLMG00005020588 | 0.727278699 | 0.009391836 |
| ENSCLMG00005004804 | -0.726677743 | 0.009189892 |
| ENSCLMG00005014996 | -0.726342603 | 0.018210884 |
| ENSCLMG00005008767 | -0.72591328 | 7.89E-07 |
| ENSCLMG00005005800 | 0.724883477 | 0.000116203 |
| ENSCLMG00005007799 | -0.724457564 | 0.015271679 |
| ENSCLMG00005021818 | 0.724295952 | 0.000159999 |
| ENSCLMG00005017684 | -0.724225333 | 0.001490227 |
| ENSCLMG00005012864 | -0.724077783 | 0.011647239 |
| ENSCLMG00005007194 | -0.723172281 | 4.54E-05 |
| ENSCLMG00005012202 | -0.721012999 | 0.032370616 |
| ENSCLMG00005004866 | 0.720569236 | 0.001753246 |
| ENSCLMG00005017375 | -0.720236955 | 0.0001482 |
| ENSCLMG00005011313 | -0.718661668 | 0.026279655 |
| ENSCLMG00005005835 | -0.71862208 | 0.001104777 |
| ENSCLMG00005003308 | -0.718291419 | 0.00010032 |
| ENSCLMG00005014979 | 0.717593634 | 0.000454837 |
| ENSCLMG00005011368 | -0.717574125 | 4.63E-05 |
| ENSCLMG00005000844 | -0.715995273 | 0.005456562 |
| ENSCLMG00005015225 | -0.715548134 | 1.68E-07 |
| ENSCLMG00005019698 | -0.715457861 | 0.009174983 |
| ENSCLMG00005000057 | -0.714963281 | 4.40E-05 |
| ENSCLMG00005017854 | -0.712622515 | 3.54E-13 |
| ENSCLMG00005003533 | 0.712182537 | 0.038121898 |
| ENSCLMG00005020340 | -0.712000562 | 0.00126261 |
| ENSCLMG00005021514 | 0.711967992 | 0.000722613 |
| ENSCLMG00005007680 | -0.711854887 | 0.008116101 |
| ENSCLMG00005000407 | -0.711620163 | 1.32E-05 |
| ENSCLMG00005010743 | -0.710813711 | 0.044584981 |
| ENSCLMG00005007974 | -0.710606184 | 4.20E-05 |
| ENSCLMG00005013713 | 0.710370628 | 0.001987427 |
| ENSCLMG00005006811 | -0.707850953 | 0.000326859 |
| ENSCLMG00005010717 | -0.707525722 | 0.007375734 |
| ENSCLMG00005009996 | -0.706322042 | 0.014102923 |
| ENSCLMG00005021749 | -0.703687957 | 5.86E-08 |
| ENSCLMG00005006805 | -0.702330683 | 0.005753677 |
| ENSCLMG00005003550 | 0.70134152 | 0.000651993 |
| ENSCLMG00005018239 | -0.70049159 | 0.015726685 |
| ENSCLMG00005014452 | -0.700117704 | 0.020331055 |
| ENSCLMG00005004009 | -0.700074272 | 0.000528587 |
| ENSCLMG00005009993 | -0.697475334 | 0.000250484 |
| ENSCLMG00005010219 | -0.696425046 | 2.13E-12 |
| ENSCLMG00005006676 | -0.694798094 | 0.048756891 |
| ENSCLMG00005014257 | -0.692789965 | 0.001522523 |
| ENSCLMG00005011644 | 0.692130049 | 0.049231128 |
| ENSCLMG00005022457 | -0.689073666 | 0.013891068 |
| ENSCLMG00005007101 | -0.688762798 | 0.024197103 |
| ENSCLMG00005020385 | 0.688169761 | 0.01193774 |
| ENSCLMG00005012100 | -0.687705233 | 3.88E-05 |
| ENSCLMG00005001941 | 0.686648078 | 0.030049394 |
| ENSCLMG00005003596 | -0.686278696 | 0.020988143 |
| ENSCLMG00005000864 | 0.686016462 | 0.003395247 |
| ENSCLMG00005020265 | -0.682984855 | 0.007602518 |
| ENSCLMG00005004862 | -0.682599603 | 3.90E-06 |
| ENSCLMG00005002717 | -0.681005719 | 2.29E-06 |
| ENSCLMG00005017962 | -0.68049257 | 0.002707068 |
| ENSCLMG00005022211 | -0.677757735 | 0.006039473 |
| ENSCLMG00005007354 | -0.677236772 | 0.000897585 |
| ENSCLMG00005022475 | -0.676983952 | 0.004203995 |
| ENSCLMG00005011554 | -0.675976738 | 1.20E-10 |
| ENSCLMG00005000562 | 0.675686843 | 0.047070228 |
| ENSCLMG00005014419 | -0.675499061 | 0.001980787 |
| ENSCLMG00005016419 | -0.675424635 | 0.000930425 |
| ENSCLMG00005016468 | 0.674747697 | 0.013673829 |
| ENSCLMG00005011059 | 0.674482672 | 0.012680807 |
| ENSCLMG00005004146 | -0.674277214 | 0.011798835 |
| ENSCLMG00005010862 | -0.674041243 | 0.000583406 |
| ENSCLMG00005001062 | -0.673136141 | 0.038615177 |
| ENSCLMG00005006136 | -0.672877653 | 0.000860359 |
| ENSCLMG00005003045 | -0.672400891 | 0.015631639 |
| ENSCLMG00005008913 | -0.671656375 | 0.017062414 |
| ENSCLMG00005015091 | -0.670493162 | 0.018712636 |
| ENSCLMG00005003598 | -0.669458087 | 3.51E-06 |
| ENSCLMG00005003633 | -0.668674752 | 0.001253804 |
| ENSCLMG00005010031 | 0.668339323 | 0.023885681 |
| ENSCLMG00005016249 | -0.668259985 | 7.99E-05 |
| ENSCLMG00005002999 | -0.668215536 | 1.71E-05 |
| ENSCLMG00005007938 | -0.668086758 | 0.035481831 |
| ENSCLMG00005018568 | 0.665341246 | 0.002630182 |
| ENSCLMG00005005573 | -0.665101153 | 0.008135408 |
| ENSCLMG00005007707 | -0.664755529 | 0.007068201 |
| ENSCLMG00005005602 | -0.664571246 | 8.55E-09 |
| ENSCLMG00005023007 | -0.663148803 | 0.001216679 |
| ENSCLMG00005020134 | -0.663062299 | 0.016652233 |
| ENSCLMG00005004512 | -0.662430252 | 7.02E-05 |
| ENSCLMG00005019379 | -0.662302655 | 0.000213517 |
| ENSCLMG00005016655 | 0.661461144 | 5.91E-05 |
| ENSCLMG00005005809 | -0.660974267 | 0.004111544 |
| ENSCLMG00005002790 | 0.660574735 | 0.012190677 |
| ENSCLMG00005019677 | -0.659122346 | 0.004208954 |
| ENSCLMG00005022952 | 0.65865793 | 0.027965826 |
| ENSCLMG00005022917 | -0.658249238 | 0.025697531 |
| ENSCLMG00005015718 | -0.657962647 | 1.12E-05 |
| ENSCLMG00005007971 | -0.657742144 | 0.000262228 |
| ENSCLMG00005002803 | -0.657412314 | 0.009759052 |
| ENSCLMG00005002029 | -0.655221793 | 0.038805886 |
| ENSCLMG00005022536 | -0.653627676 | 0.000467666 |
| ENSCLMG00005010941 | -0.652528038 | 5.40E-07 |
| ENSCLMG00005011857 | -0.651614695 | 0.000244745 |
| ENSCLMG00005009639 | -0.651151889 | 0.005192469 |
| ENSCLMG00005023039 | -0.650744979 | 0.033332502 |
| ENSCLMG00005019636 | -0.65042901 | 0.001935904 |
| ENSCLMG00005020431 | 0.649175612 | 0.001022708 |
| ENSCLMG00005019315 | -0.648822712 | 0.027354732 |
| ENSCLMG00005019827 | -0.64836042 | 0.01686411 |
| ENSCLMG00005008809 | -0.648266985 | 0.038137771 |
| ENSCLMG00005015951 | 0.648226358 | 0.017964408 |
| ENSCLMG00005009221 | -0.647966041 | 0.000304855 |
| ENSCLMG00005003117 | -0.646821226 | 0.046692376 |
| ENSCLMG00005012834 | -0.645401608 | 0.00232497 |
| ENSCLMG00005018962 | -0.643931744 | 0.000852399 |
| ENSCLMG00005017954 | -0.643908096 | 6.80E-09 |
| ENSCLMG00005001786 | -0.642169631 | 0.001183397 |
| ENSCLMG00005022812 | -0.640923228 | 0.013656946 |
| ENSCLMG00005020658 | -0.639674237 | 0.009733576 |
| ENSCLMG00005021563 | -0.63952591 | 0.020621751 |
| ENSCLMG00005013721 | -0.636972829 | 0.020988143 |
| ENSCLMG00005017642 | 0.636261011 | 0.013855702 |
| ENSCLMG00005013921 | -0.634266593 | 0.009499488 |
| ENSCLMG00005014798 | -0.634145897 | 0.007068201 |
| ENSCLMG00005003071 | -0.633542778 | 0.017252647 |
| ENSCLMG00005005920 | -0.632985694 | 0.039116067 |
| ENSCLMG00005007871 | -0.632636463 | 0.027618731 |
| ENSCLMG00005015908 | 0.63152464 | 0.041138935 |
| ENSCLMG00005000726 | -0.631479433 | 7.93E-08 |
| ENSCLMG00005007010 | -0.631408846 | 0.006314057 |
| ENSCLMG00005017896 | 0.630180537 | 0.009169408 |
| ENSCLMG00005017021 | -0.630139151 | 7.65E-05 |
| ENSCLMG00005016912 | -0.629989108 | 0.031374994 |
| ENSCLMG00005014535 | 0.626668596 | 0.005103163 |
| ENSCLMG00005012423 | -0.626660618 | 0.000634422 |
| ENSCLMG00005011427 | -0.626494462 | 0.007483092 |
| ENSCLMG00005014493 | 0.625987834 | 4.42E-06 |
| ENSCLMG00005015237 | -0.625177684 | 4.38E-06 |
| ENSCLMG00005003435 | 0.624306409 | 0.039277314 |
| ENSCLMG00005020582 | -0.62332429 | 0.016713999 |
| ENSCLMG00005005087 | -0.62219063 | 0.000242407 |
| ENSCLMG00005007312 | -0.621011355 | 0.00099368 |
| ENSCLMG00005006320 | -0.620742264 | 3.04E-08 |
| ENSCLMG00005007685 | -0.619540414 | 0.002768731 |
| ENSCLMG00005020809 | -0.61929436 | 0.003205089 |
| ENSCLMG00005007272 | -0.618268404 | 0.041211903 |
| ENSCLMG00005022453 | -0.617878676 | 0.000142846 |
| ENSCLMG00005010095 | -0.616689271 | 0.001100571 |
| ENSCLMG00005008103 | 0.614581669 | 0.028860766 |
| ENSCLMG00005005567 | 0.613467407 | 0.014371525 |
| ENSCLMG00005014227 | -0.611654397 | 0.000669755 |
| ENSCLMG00005011156 | -0.611348638 | 1.59E-08 |
| ENSCLMG00005015986 | 0.610996495 | 0.029561545 |
| ENSCLMG00005020659 | 0.61054861 | 0.042344139 |
| ENSCLMG00005000130 | -0.610316703 | 0.016901947 |
| ENSCLMG00005019373 | -0.609016394 | 0.013656946 |
| ENSCLMG00005020825 | 0.607965728 | 0.018939517 |
| ENSCLMG00005019431 | -0.60752651 | 3.86E-05 |
| ENSCLMG00005003130 | -0.606709237 | 2.14E-07 |
| ENSCLMG00005018784 | 0.606145039 | 0.001307959 |
| ENSCLMG00005006174 | 0.605139417 | 0.020820641 |
| ENSCLMG00005013590 | -0.604377217 | 0.039770121 |
| ENSCLMG00005010461 | -0.604156013 | 0.034580392 |
| ENSCLMG00005011256 | -0.604031343 | 0.002581292 |
| ENSCLMG00005020866 | -0.603905238 | 0.013988431 |
| ENSCLMG00005015196 | 0.602960802 | 0.004576595 |
| ENSCLMG00005017663 | 0.60289169 | 0.001159734 |
| ENSCLMG00005017742 | 0.602344148 | 3.27E-07 |
| ENSCLMG00005006394 | -0.602178737 | 8.87E-05 |
| ENSCLMG00005015016 | -0.601355658 | 0.002383965 |
| ENSCLMG00005009659 | -0.599625287 | 0.041821106 |
| ENSCLMG00005018600 | -0.598736626 | 0.000100722 |
| ENSCLMG00005007917 | 0.597749321 | 0.034531625 |
| ENSCLMG00005021872 | -0.597743752 | 0.000535484 |
| ENSCLMG00005015538 | -0.597461755 | 1.57E-06 |
| ENSCLMG00005009963 | -0.595541677 | 0.001276267 |
| ENSCLMG00005019390 | -0.595262469 | 0.02013237 |
| ENSCLMG00005015808 | -0.594836783 | 0.015822582 |
| ENSCLMG00005019012 | -0.593644442 | 2.19E-10 |
| ENSCLMG00005022595 | -0.59248627 | 0.004237695 |
| ENSCLMG00005012397 | -0.59130483 | 0.003791076 |
| ENSCLMG00005008500 | 0.590604638 | 0.029335599 |
| ENSCLMG00005000673 | -0.589299424 | 0.00126261 |
| ENSCLMG00005000862 | -0.589227713 | 0.011825149 |
| ENSCLMG00005014961 | -0.589048188 | 6.15E-05 |
| ENSCLMG00005006939 | -0.588557553 | 1.02E-05 |
| ENSCLMG00005018971 | -0.586231927 | 0.002526453 |
| ENSCLMG00005016774 | -0.585926247 | 0.004579758 |
| ENSCLMG00005016508 | -0.58590122 | 0.042068093 |
| ENSCLMG00005014176 | -0.584146224 | 0.024971811 |
| ENSCLMG00005004995 | -0.58390577 | 0.031671178 |
| ENSCLMG00005015769 | 0.583629797 | 0.006521713 |
| ENSCLMG00005018846 | 0.583492829 | 0.000281527 |
| ENSCLMG00005005574 | -0.583464275 | 0.009623629 |
| ENSCLMG00005005853 | -0.583293965 | 0.000465636 |
| ENSCLMG00005011025 | -0.582339254 | 0.001773274 |
| ENSCLMG00005000043 | -0.582275664 | 0.018712636 |
| ENSCLMG00005011724 | -0.581537289 | 8.42E-08 |
| ENSCLMG00005003241 | -0.581431664 | 0.02736094 |
| ENSCLMG00005003950 | -0.58104957 | 0.049883649 |
| ENSCLMG00005013323 | 0.580864846 | 0.01048779 |
| ENSCLMG00005008211 | 0.57984457 | 0.007194945 |
| ENSCLMG00005014378 | -0.579321169 | 0.001102644 |
| ENSCLMG00005000599 | -0.577568559 | 0.003300919 |
| ENSCLMG00005012133 | 0.577136505 | 0.00236475 |
| ENSCLMG00005001935 | -0.575456671 | 0.001957536 |
| ENSCLMG00005015546 | 0.574227018 | 0.000101693 |
| ENSCLMG00005006221 | -0.573698366 | 0.008378487 |
| ENSCLMG00005010121 | 0.572971054 | 0.01048779 |
| ENSCLMG00005003305 | -0.572797296 | 0.027014158 |
| ENSCLMG00005019716 | 0.572228156 | 0.000386104 |
| ENSCLMG00005012469 | -0.571557063 | 0.049296417 |
| ENSCLMG00005005223 | -0.57150765 | 0.026798316 |
| ENSCLMG00005020803 | -0.570429847 | 0.04349325 |
| ENSCLMG00005003584 | -0.569145586 | 0.008863081 |
| ENSCLMG00005013821 | 0.568646419 | 0.027713745 |
| ENSCLMG00005013460 | -0.568350364 | 0.000640865 |
| ENSCLMG00005014197 | -0.568183843 | 0.001071916 |
| ENSCLMG00005020732 | 0.56763034 | 0.0119366 |
| ENSCLMG00005021159 | -0.56687887 | 0.004175263 |
| ENSCLMG00005018158 | 0.56613517 | 0.000178311 |
| ENSCLMG00005000183 | -0.564083715 | 0.034844189 |
| ENSCLMG00005009371 | -0.562934081 | 0.011450272 |
| ENSCLMG00005008196 | -0.562747084 | 0.006657986 |
| ENSCLMG00005008841 | -0.562213944 | 8.87E-05 |
| ENSCLMG00005022618 | -0.562209166 | 0.002722456 |
| ENSCLMG00005021216 | -0.562204892 | 0.000893765 |
| ENSCLMG00005009854 | -0.561680381 | 0.029012402 |
| ENSCLMG00005007144 | -0.560326497 | 0.028892052 |
| ENSCLMG00005017173 | -0.560185413 | 0.028311962 |
| ENSCLMG00005022178 | 0.559561062 | 0.030074451 |
| ENSCLMG00005010600 | 0.55915816 | 0.03848102 |
| ENSCLMG00005005884 | -0.558508747 | 0.00123854 |
| ENSCLMG00005019085 | -0.558013528 | 0.049069503 |
| ENSCLMG00005015051 | 0.555815216 | 0.035427721 |
| ENSCLMG00005014236 | -0.555419882 | 6.33E-05 |
| ENSCLMG00005001840 | -0.552973391 | 0.028892052 |
| ENSCLMG00005022234 | -0.552971641 | 0.02047743 |
| ENSCLMG00005021618 | -0.55182924 | 0.035541193 |
| ENSCLMG00005015371 | -0.551081175 | 2.77E-05 |
| ENSCLMG00005020167 | -0.550015885 | 1.72E-07 |
| ENSCLMG00005015062 | -0.549029575 | 0.003300919 |
| ENSCLMG00005012510 | 0.548791635 | 0.021396166 |
| ENSCLMG00005015724 | -0.548520739 | 0.017964408 |
| ENSCLMG00005005600 | -0.547900178 | 7.12E-05 |
| ENSCLMG00005008207 | -0.547860545 | 0.003436264 |
| ENSCLMG00005014742 | -0.547348214 | 0.000107642 |
| ENSCLMG00005020523 | -0.547193753 | 0.018210884 |
| ENSCLMG00005008486 | -0.546285245 | 0.000287311 |
| ENSCLMG00005015659 | -0.546256552 | 0.000191628 |
| ENSCLMG00005002591 | -0.54458013 | 0.006179908 |
| ENSCLMG00005007994 | 0.544050918 | 0.033281148 |
| ENSCLMG00005013884 | -0.544005347 | 0.005590204 |
| ENSCLMG00005002176 | -0.543978876 | 0.038073728 |
| ENSCLMG00005003922 | -0.542138425 | 0.000495017 |
| ENSCLMG00005003783 | 0.541541167 | 0.037688311 |
| ENSCLMG00005006496 | -0.541300276 | 0.008906188 |
| ENSCLMG00005001571 | -0.540570738 | 0.000987479 |
| ENSCLMG00005006069 | -0.540222824 | 0.000359669 |
| ENSCLMG00005016060 | -0.538332416 | 0.018159724 |
| ENSCLMG00005010950 | -0.536791894 | 0.002099378 |
| ENSCLMG00005018501 | 0.535622785 | 0.007769428 |
| ENSCLMG00005006304 | -0.534533327 | 0.000229201 |
| ENSCLMG00005000772 | 0.533870171 | 0.000145395 |
| ENSCLMG00005015523 | 0.533728655 | 0.00373115 |
| ENSCLMG00005022455 | -0.532473059 | 0.039702214 |
| ENSCLMG00005020386 | 0.530781197 | 0.036918849 |
| ENSCLMG00005022198 | -0.530469606 | 3.24E-05 |
| ENSCLMG00005021500 | -0.53009541 | 0.003832349 |
| ENSCLMG00005014016 | 0.52913155 | 0.035640421 |
| ENSCLMG00005010604 | 0.527865221 | 0.046638488 |
| ENSCLMG00005012508 | 0.526111226 | 0.017449057 |
| ENSCLMG00005017361 | -0.523229814 | 0.0254429 |
| ENSCLMG00005005458 | -0.519503755 | 0.00023187 |
| ENSCLMG00005000336 | -0.518419628 | 0.00964029 |
| ENSCLMG00005011007 | -0.517900478 | 4.75E-10 |
| ENSCLMG00005007334 | 0.517668198 | 0.030074451 |
| ENSCLMG00005018483 | -0.517558043 | 0.035640421 |
| ENSCLMG00005018709 | -0.517481088 | 4.83E-05 |
| ENSCLMG00005001991 | 0.517266593 | 0.005719093 |
| ENSCLMG00005018507 | -0.516342595 | 0.047210474 |
| ENSCLMG00005018854 | -0.516304993 | 0.00051204 |
| ENSCLMG00005002583 | -0.516114475 | 0.00725741 |
| ENSCLMG00005022323 | -0.51420111 | 0.04993326 |
| ENSCLMG00005003396 | 0.513622623 | 0.032883109 |
| ENSCLMG00005005878 | -0.512051586 | 0.000111841 |
| ENSCLMG00005020664 | -0.511239445 | 0.013228516 |
| ENSCLMG00005000118 | 0.510663181 | 0.016295524 |
| ENSCLMG00005007423 | 0.51039646 | 0.038855644 |
| ENSCLMG00005006479 | -0.510162252 | 0.017678621 |
| ENSCLMG00005020629 | -0.50997424 | 0.0152058 |
| ENSCLMG00005016444 | 0.509669495 | 0.045293114 |
| ENSCLMG00005014727 | 0.509322191 | 0.001834165 |
| ENSCLMG00005000487 | 0.508595551 | 0.009640045 |
| ENSCLMG00005008902 | 0.50400129 | 0.033564639 |
| ENSCLMG00005015530 | -0.503957919 | 0.045886381 |
| ENSCLMG00005012066 | -0.50345063 | 0.004023778 |
| ENSCLMG00005015494 | -0.502919373 | 0.023795976 |
| ENSCLMG00005021522 | -0.499685905 | 0.001797642 |
| ENSCLMG00005011992 | -0.499254207 | 4.19E-06 |
| ENSCLMG00005019768 | -0.499175016 | 0.044931048 |
| ENSCLMG00005019318 | -0.498221477 | 0.021482954 |
| ENSCLMG00005002531 | -0.493158143 | 0.047766142 |
| ENSCLMG00005012963 | -0.491959195 | 0.001178378 |
| ENSCLMG00005005680 | -0.491661504 | 8.02E-06 |
| ENSCLMG00005002220 | -0.49140316 | 0.030375122 |
| ENSCLMG00005000380 | -0.490558156 | 0.020339583 |
| ENSCLMG00005016006 | -0.49055796 | 0.036712107 |
| ENSCLMG00005007815 | 0.489676733 | 0.000266614 |
| ENSCLMG00005021033 | -0.489425514 | 0.02282202 |
| ENSCLMG00005006418 | -0.488440546 | 0.047680477 |
| ENSCLMG00005005454 | -0.487429203 | 0.039472101 |
| ENSCLMG00005013964 | 0.48609594 | 0.003985982 |
| ENSCLMG00005009281 | -0.483635659 | 0.000244902 |
| ENSCLMG00005012493 | 0.482752804 | 0.006261062 |
| ENSCLMG00005004124 | -0.48098551 | 3.45E-09 |
| ENSCLMG00005003848 | 0.480707333 | 0.013891068 |
| ENSCLMG00005019650 | -0.480560342 | 0.015790607 |
| ENSCLMG00005012492 | -0.478337726 | 0.022307707 |
| ENSCLMG00005008687 | 0.478276408 | 0.001602627 |
| ENSCLMG00005005652 | 0.476944714 | 0.045102285 |
| ENSCLMG00005011222 | -0.4765604 | 0.039770121 |
| ENSCLMG00005013535 | -0.475948747 | 0.008796854 |
| ENSCLMG00005022713 | -0.475743682 | 0.006390988 |
| ENSCLMG00005003849 | 0.474691555 | 0.000111649 |
| ENSCLMG00005007426 | -0.474142718 | 0.006460103 |
| ENSCLMG00005005577 | 0.473151918 | 0.009930249 |
| ENSCLMG00005014833 | 0.471719166 | 0.041178168 |
| ENSCLMG00005008630 | -0.471608665 | 0.002880171 |
| ENSCLMG00005004805 | -0.470841686 | 0.00156066 |
| ENSCLMG00005000939 | 0.469799967 | 0.021242079 |
| ENSCLMG00005021017 | 0.469196807 | 0.020517474 |
| ENSCLMG00005017986 | -0.469076025 | 0.004175263 |
| ENSCLMG00005007218 | -0.467826526 | 0.009007663 |
| ENSCLMG00005017303 | 0.467338093 | 0.041988113 |
| ENSCLMG00005012677 | -0.465668687 | 0.000227315 |
| ENSCLMG00005020951 | -0.465251281 | 0.041623533 |
| ENSCLMG00005017483 | -0.462442453 | 0.047561488 |
| ENSCLMG00005001258 | -0.462241447 | 0.037146425 |
| ENSCLMG00005006010 | -0.460643159 | 0.002755747 |
| ENSCLMG00005017911 | 0.458669418 | 0.001999802 |
| ENSCLMG00005016203 | 0.456919191 | 0.045293114 |
| ENSCLMG00005013717 | 0.456641216 | 0.024838138 |
| ENSCLMG00005000796 | -0.456590936 | 0.020554477 |
| ENSCLMG00005006966 | -0.456340247 | 0.002798757 |
| ENSCLMG00005013880 | 0.45628506 | 0.017289828 |
| ENSCLMG00005005377 | -0.454595805 | 0.021559998 |
| ENSCLMG00005010631 | -0.452461602 | 0.003171762 |
| ENSCLMG00005012141 | -0.451561492 | 0.011406332 |
| ENSCLMG00005005126 | -0.450963769 | 0.032034743 |
| ENSCLMG00005013287 | -0.450923939 | 0.020621751 |
| ENSCLMG00005018177 | 0.450491745 | 0.014779123 |
| ENSCLMG00005023089 | -0.450448048 | 0.013913451 |
| ENSCLMG00005021497 | -0.450172873 | 0.004418325 |
| ENSCLMG00005011436 | -0.447435695 | 0.017289828 |
| ENSCLMG00005004834 | -0.447417729 | 0.020477984 |
| ENSCLMG00005008825 | 0.447189868 | 0.031560661 |
| ENSCLMG00005010921 | -0.446727201 | 0.028380283 |
| ENSCLMG00005015948 | -0.443588937 | 0.049231128 |
| ENSCLMG00005012757 | -0.439315275 | 0.001538658 |
| ENSCLMG00005003091 | -0.438648308 | 0.009793348 |
| ENSCLMG00005014973 | -0.437702983 | 0.041297671 |
| ENSCLMG00005001484 | 0.43766572 | 0.044753912 |
| ENSCLMG00005019690 | 0.436247034 | 0.038364329 |
| ENSCLMG00005022737 | -0.435066285 | 0.033253095 |
| ENSCLMG00005022662 | -0.434146868 | 0.006895287 |
| ENSCLMG00005022499 | -0.433941443 | 1.51E-05 |
| ENSCLMG00005006478 | -0.433616146 | 0.03554126 |
| ENSCLMG00005016672 | 0.432650794 | 0.007883809 |
| ENSCLMG00005010131 | -0.431148003 | 0.021787749 |
| ENSCLMG00005006325 | -0.429607435 | 0.009798435 |
| ENSCLMG00005009772 | 0.428840282 | 0.001525576 |
| ENSCLMG00005008975 | 0.427004737 | 0.021381645 |
| ENSCLMG00005008014 | -0.423142036 | 0.041054951 |
| ENSCLMG00005021732 | 0.422376177 | 0.040904525 |
| ENSCLMG00005001592 | 0.421375866 | 0.042068093 |
| ENSCLMG00005016142 | -0.417543278 | 0.024883154 |
| ENSCLMG00005002340 | -0.417203862 | 0.02159915 |
| ENSCLMG00005014398 | 0.417187103 | 0.049936866 |
| ENSCLMG00005021067 | -0.415649806 | 0.038615177 |
| ENSCLMG00005020302 | 0.413979468 | 0.035226508 |
| ENSCLMG00005021288 | -0.413842062 | 0.015938219 |
| ENSCLMG00005002053 | -0.411592983 | 0.040416099 |
| ENSCLMG00005003996 | 0.407310724 | 0.017995769 |
| ENSCLMG00005014848 | -0.406568335 | 0.00349345 |
| ENSCLMG00005008157 | 0.404472343 | 0.007486459 |
| ENSCLMG00005019606 | 0.397015869 | 6.53E-05 |
| ENSCLMG00005003831 | 0.396451959 | 0.001528832 |
| ENSCLMG00005022460 | -0.396039289 | 0.037302943 |
| ENSCLMG00005005293 | -0.393262213 | 0.016599648 |
| ENSCLMG00005017646 | 0.387674549 | 0.009398996 |
| ENSCLMG00005016349 | -0.383463764 | 0.025697531 |
| ENSCLMG00005005139 | 0.383067809 | 0.034437154 |
| ENSCLMG00005007705 | -0.382735287 | 0.019488011 |
| ENSCLMG00005003504 | -0.377108092 | 0.028876093 |
| ENSCLMG00005001068 | 0.375864651 | 0.034147033 |
| ENSCLMG00005016616 | -0.37563799 | 0.029166425 |
| ENSCLMG00005009901 | -0.374332019 | 0.027508264 |
| ENSCLMG00005002849 | -0.37429671 | 0.019978733 |
| ENSCLMG00005015490 | -0.373121517 | 0.016951091 |
| ENSCLMG00005002466 | 0.372518396 | 0.023074396 |
| ENSCLMG00005018358 | 0.367163644 | 0.024343385 |
| ENSCLMG00005015192 | -0.366872689 | 0.019342792 |
| ENSCLMG00005001026 | -0.361425652 | 0.003279043 |
| ENSCLMG00005018916 | -0.360144638 | 0.040904525 |
| ENSCLMG00005023062 | -0.350943662 | 0.017289828 |
| ENSCLMG00005009303 | -0.350544712 | 0.006053833 |
| ENSCLMG00005021771 | -0.342326132 | 0.048848662 |
| ENSCLMG00005013577 | -0.341234018 | 0.021396166 |
| ENSCLMG00005008977 | 0.33894751 | 0.015726685 |
| ENSCLMG00005000338 | -0.338916039 | 0.008448291 |
| ENSCLMG00005012189 | 0.329065774 | 0.044925491 |
| ENSCLMG00005015588 | 0.328375995 | 0.047561488 |
| ENSCLMG00005000314 | 0.327963896 | 0.025803324 |
| ENSCLMG00005012691 | 0.321991968 | 0.01513934 |
| ENSCLMG00005009102 | -0.321522591 | 0.029279872 |
| ENSCLMG00005009109 | 0.318431556 | 0.002835837 |
| ENSCLMG00005020902 | 0.317376437 | 0.023754055 |
| ENSCLMG00005008544 | -0.317310725 | 0.016543707 |
| ENSCLMG00005015442 | -0.316744893 | 0.017548461 |
| ENSCLMG00005007015 | 0.316066252 | 0.044543371 |
| ENSCLMG00005022193 | 0.315027907 | 0.006251894 |
| ENSCLMG00005003299 | 0.313903249 | 0.004528709 |
| ENSCLMG00005008182 | -0.30041319 | 0.03965787 |
| ENSCLMG00005000866 | 0.300321653 | 0.012855234 |
| ENSCLMG00005010980 | 0.298135114 | 0.024080941 |
| ENSCLMG00005022849 | 0.296223556 | 0.004137189 |
| ENSCLMG00005015197 | 0.28990403 | 0.003809188 |
| ENSCLMG00005013056 | -0.289783336 | 0.027665921 |
| ENSCLMG00005019863 | 0.286768916 | 0.046692376 |
| ENSCLMG00005016844 | 0.264894205 | 0.004166975 |
| ENSCLMG00005018984 | -0.262554321 | 0.0152058 |
| ENSCLMG00005018297 | -0.257839087 | 0.027416505 |
| ENSCLMG00005010293 | 0.255172489 | 0.047903292 |
| ENSCLMG00005014731 | -0.244603689 | 0.008863081 |
| ENSCLMG00005016976 | 0.238820852 | 0.045293114 |

* Positive logFC values= upregulated transcripts, negative logFC values=down-regulated transcripts
