# Supplementary material for: Transcriptome-wide analyses of early immune responses in lumpfish leukocytes upon stimulation with poly(I:C)
Source: Front Immunol. 2023 Jun 14;14:1198211. doi: 10.3389/fimmu.2023.1198211 (PMC10300353; doi:10.3389/fimmu.2023.1198211)
Supplement: Supplementary file 4 [file Table_2.docx]

**Supplemental Table 2.** GO terms in the time independent analyses.

**GO term: Immune system processes (GO:0002376). Gene ratio 58/676. Adjusted p-value: 0.0331**

| **Gene stable ID** | **GO term accession** | **GO term name** | **Gene name** | **Gene description** |
| --- | --- | --- | --- | --- |
| ENSCLMG00005000130 | GO:0002244 | hematopoietic prog. cell differentiation | brd8 | bromodomain containing 8 |
| ENSCLMG00005000163 | GO:1990266 | neutrophil migration | nox5 | NADPH oxidase, EF-hand calcium binding domain 5 |
| ENSCLMG00005001068 | GO:0019885 | antigen processing and presentation of endogenous peptide antigen via MHC class I | erap1b | endoplasmic reticulum aminopeptidase 1b |
| ENSCLMG00005001818 | GO:0006955 | immune response | TNF14-L | tumor necrosis factor ligand 14-like* |
| ENSCLMG00005001873 | GO:0006955 | immune response | CXC10 | C-X-C motif chemokine 10-like* |
| ENSCLMG00005002136 | GO:0035855 | megakaryocyte development | npc1 | Niemann-Pick disease, type C1 |
| ENSCLMG00005002437 | GO:0006958 | complement activation, classical pathway | C1a | Complement C1a subcomponent subunit A* |
| ENSCLMG00005002498 | GO:0050776 | regulation of immune response | HCST | hematopoietic cell signal transducer isoform X1* |
| ENSCLMG00005002557 | GO:0060334 | regulation of interferon-gamma-mediated signaling pathway | socs1b | suppressor of cytokine signaling 1b |
| ENSCLMG00005002654 | GO:0019885 | antigen processing and presentation of endogenous peptide antigen via MHC class I | tapbpl | TAP binding protein like |
| ENSCLMG00005005791 | GO:0035162 | embryonic hemopoiesis | socs1a | suppressor of cytokine signaling 1a |
|  | GO:0060334 | regulation of interferon-gamma-mediated signaling pathway |  |  |
|  | GO:0060336 | negative regulation of interferon-gamma-mediated signaling pathway |  |  |
|  | GO:2000406 | positive regulation of T cell migration |  |  |
| ENSCLMG00005005853 | GO:0030220 | platelet formation | kdsr | 3-ketodihydrosphingosine reductase |
| ENSCLMG00005006128 | GO:0030223 | neutrophil differentiation | zbtb11 | zinc finger and BTB domain containing 11 |
| ENSCLMG00005006372 | GO:0090024 | negative regulation of neutrophil chemotaxis | inpp5d | inositol polyphosphate-5-phosphatase D |
| ENSCLMG00005006636 | GO:0002376 | immune system process | psmb9a | proteasome 20S subunit beta 9a |
| ENSCLMG00005006658 | GO:0019885 | antigen processing and presentation of endogenous peptide antigen via MHC class I | tapbp.2 | TAP binding protein (tapasin), tandem duplicate 2 |
| ENSCLMG00005007453 | GO:0006955 | immune response | Cstf3 | Colony stimulating factor 3* |
| ENSCLMG00005008690 | GO:0006955 | immune response | CCL35 | C-C motif chemokine 35 |
| ENSCLMG00005008778 | GO:0006955 | immune response | CXC-CK3 | CC chemokine CK3 |
| ENSCLMG00005009114 | GO:0030097 | hemopoiesis | pdia5 | protein disulfide isomerase family A, member 5 |
| ENSCLMG00005010631 | GO:0050776 | regulation of immune response | SPPL2A | signal peptide peptidase like 2A |
| ENSCLMG00005010826 | GO:0060338 | regulation of type I interferon-mediated signaling pathway | cav1 | caveolin 1 |
| ENSCLMG00005011065 | GO:0006955 | immune response | CCL13 | C-C motif chemokine 13 |
| ENSCLMG00005011066 | GO:0006955 | immune response | CCL19 | C-C motif chemokine 19 |
| ENSCLMG00005011085 | GO:0002224 | toll-like receptor signaling pathway | TLR3 | toll-like receptor 13 |
|  | GO:0006955 | immune response | TLR3 | toll-like receptor 13 |
| ENSCLMG00005011304 | GO:0060217 | hemangioblast cell differentiation | etv5a | ETS variant transcription factor 5a |
| ENSCLMG00005012219 | GO:0002244 | hematopoietic progenitor cell differentiation | smarcd1 | SWI/SNF related |
|  | GO:0060216 | definitive hemopoiesis |  |  |
| ENSCLMG00005012330 | GO:0006955 | immune response | TNF-β | tumor necrosis factor beta |
| ENSCLMG00005012790 | GO:0006959 | humoral immune response | tfe3b | transcription factor binding to IGHM enhancer 3b |
|  | GO:0045670 | regulation of osteoclast differentiation |  |  |
| ENSCLMG00005013572 | GO:0060216 | definitive hemopoiesis | lpl | lipoprotein lipase |
| ENSCLMG00005013626 | GO:0002244 | hematopoietic progenitor cell differentiation | ptger4a | prostaglandin E receptor 4 (subtype EP4) a |
|  | GO:0033077 | T cell differentiation in thymus |  |  |
| ENSCLMG00005013682 | GO:0048821 | erythrocyte development | etv7 | ETS variant transcription factor 7 |
| ENSCLMG00005013884 | GO:0060319 | primitive erythrocyte differentiation | cicb | capicua transcriptional repressor b |
| ENSCLMG00005013894 | GO:0060217 | hemangioblast cell differentiation | snrkb | SNF related kinase b |
| ENSCLMG00005014176 | GO:0002221 | pattern recognition receptor signaling pathway | tlr21 | toll-like receptor 21 |
|  | GO:0002224 | toll-like receptor signaling pathway |  |  |
|  | GO:0006955 | immune response |  |  |
|  | GO:0019731 | antibacterial humoral response |  |  |
| ENSCLMG00005014178 | GO:0002281 | macrophage activation involved in immune response | cxcr3.2 | chemokine (C-X-C motif) receptor 3, tandem duplicate 2 |
|  | GO:0002522 | leukocyte migration involved in immune response |  |  |
|  | GO:0048246 | macrophage chemotaxis |  |  |
| ENSCLMG00005015196 | GO:0030098 | lymphocyte differentiation | stat5a | signal transducer and activator of transcription 5a |
|  | GO:0030218 | erythrocyte differentiation |  |  |
|  | GO:0045639 | positive regulation of myeloid cell differentiation |  |  |
|  | GO:0048821 | erythrocyte development |  |  |
| ENSCLMG00005015340 | GO:0050776 | regulation of immune response | irf1b | interferon regulatory factor 1b |
| ENSCLMG00005015486 | GO:0002224 | toll-like receptor signaling pathway | tlr7 | toll-like receptor 7 |
|  | GO:0006955 | immune response |  |  |
| ENSCLMG00005015724 | GO:0030097 | hemopoiesis | rcor1 | REST corepressor 1 |
| ENSCLMG00005015934 | GO:0060218 | hematopoietic stem cell differentiation | snrnp70 | small nuclear ribonucleoprotein 70 (U1) |
| ENSCLMG00005015980 | GO:0006955 | immune response | CXCL10 | C-X-C motif chemokine 10 * |
| ENSCLMG00005016663 | GO:0006955 | immune response | il12a | interleukin 12a |
| ENSCLMG00005018959 | GO:0006955 | immune response | tnfsf12 | TNF superfamily member 12 |
| ENSCLMG00005019424 | GO:0002218 | activation of innate immune response | sting1 | stimulator of interferon response cGAMP interactor 1 |
|  | GO:0045087 | innate immune response |  |  |
| ENSCLMG00005019998 | GO:0002574 | thrombocyte differentiation | brd4 | bromodomain containing 4 |
| ENSCLMG00005020000 | GO:0045647 | negative regulation of erythrocyte differentiation | lpar2a | lysophosphatidic acid receptor 2a |
| ENSCLMG00005020007 | GO:0060216 | definitive hemopoiesis | myef2 | myelin expression factor 2 |
| ENSCLMG00005020809 | GO:0045576 | mast cell activation | ndrg1a | N-myc downstream regulated 1a |
| ENSCLMG00005021280 | GO:1903707 | negative regulation of hemopoiesis | gpr65 | G protein-coupled receptor 65 |
| ENSCLMG00005021558 | GO:0043249 | erythrocyte maturation | klf6a | Kruppel-like factor 6a |
|  | GO:1902033 | regulation of hematopoietic stem cell proliferation |  |  |
| ENSCLMG00005021732 | GO:0019885 | antigen processing and presentation of endogenous peptide antigen via MHC class I | erap2 | endoplasmic reticulum aminopeptidase 2 |
| ENSCLMG00005022205 | GO:0006955 | immune response | IL-6 | interleukin-6-like* |
|  | GO:0072540 | T-helper 17 cell lineage commitment |  |  |
| ENSCLMG00005022603 | GO:0006955 | immune response | TNFSF10 | TNF superfamily member 10 |
| ENSCLMG00005022952 | GO:0030097 | hemopoiesis | irf7 | interferon regulatory factor 7 |
|  | GO:0045580 | regulation of T cell differentiation |  |  |
|  | GO:1902037 | negative regulation of hematopoietic stem cell differentiation |  |  |

*= annotation based on blastp search

**GO term: Immune response (GO:0006955). Gene ratio 27/676. Adjusted p-value: 0.0049**

| **Gene stable ID** | **GO term accession** | **GO term name** | **Gene name** | **Gene description** |
| --- | --- | --- | --- | --- |
| ENSCLMG00005001818 | GO:0006955 | immune response | TNF14-L | tumor necrosis factor ligand 14-like* |
| ENSCLMG00005001873 | GO:0006955 | immune response | CXCL10 | C-X-C motif chemokine 10* |
| ENSCLMG00005002437 | GO:0006958 | complement activation, classical pathway | C1a | Complement C1a subcomponent subunit A* |
| ENSCLMG00005002498 | GO:0050776 | regulation of immune response | HCST | hematopoietic cell signal transducer isoform X1* |
| ENSCLMG00005002557 | GO:0060334 | regulation of interferon-gamma-mediated signaling pathway | socs1b | suppressor of cytokine signaling 1b |
| ENSCLMG00005005791 | GO:0060334 | regulation of interferon-gamma-mediated signaling pathway | socs1a | suppressor of cytokine signaling 1a |
| ENSCLMG00005005791 | GO:0060336 | negative regulation of interferon-gamma-mediated signaling pathway | socs1a | suppressor of cytokine signaling 1a |
| ENSCLMG00005007453 | GO:0006955 | immune response | Cstf3 | Colony stimulating factor 3* |
| ENSCLMG00005008690 | GO:0006955 | immune response | CCL35 | C-C motif chemokine 35 |
| ENSCLMG00005008778 | GO:0006955 | immune response | CXC-CK3 | CC chemokine CK3* |
| ENSCLMG00005010631 | GO:0050776 | regulation of immune response | SPPL2A | signal peptide peptidase like 2A |
| ENSCLMG00005010826 | GO:0060338 | regulation of type I interferon-mediated signaling pathway | cav1 | caveolin 1 |
| ENSCLMG00005011065 | GO:0006955 | immune response | CCL13 | C-C motif chemokine 13* |
| ENSCLMG00005011066 | GO:0006955 | immune response | CCL19 | C-C motif chemokine 19* |
| ENSCLMG00005011085 | GO:0002224 | toll-like receptor signaling pathway | TLR3 | toll-like receptor 13* |
| ENSCLMG00005011085 | GO:0006955 | immune response | TLR3 | toll-like receptor 13* |
| ENSCLMG00005012330 | GO:0006955 | immune response | TNF-β | tumor necrosis factor beta* |
| ENSCLMG00005012790 | GO:0006959 | humoral immune response | tfe3b | transcription factor binding to IGHM enhancer 3b |
| ENSCLMG00005014176 | GO:0002221 | pattern recognition receptor signaling pathway | tlr21 | toll-like receptor 21 |
| ENSCLMG00005014176 | GO:0002224 | toll-like receptor signaling pathway | tlr21 | toll-like receptor 21 |
| ENSCLMG00005014176 | GO:0006955 | immune response | tlr21 | toll-like receptor 21 |
| ENSCLMG00005014176 | GO:0019731 | antibacterial humoral response | tlr21 | toll-like receptor 21 |
| ENSCLMG00005014178 | GO:0002281 | macrophage activation involved in immune response | cxcr3.2 | chemokine (C-X-C motif) receptor 3, tandem duplicate 2 |
| ENSCLMG00005014178 | GO:0002522 | leukocyte migration involved in immune response | cxcr3.2 | chemokine (C-X-C motif) receptor 3, tandem duplicate 2 |
| ENSCLMG00005015340 | GO:0050776 | regulation of immune response | irf1b | interferon regulatory factor 1b |
| ENSCLMG00005015486 | GO:0002224 | toll-like receptor signaling pathway | tlr7 | toll-like receptor 7 |
| ENSCLMG00005015486 | GO:0006955 | immune response | tlr7 | toll-like receptor 7 |
| ENSCLMG00005015980 | GO:0006955 | immune response | CXCL10 | C-X-C motif chemokine 10 * |
| ENSCLMG00005016663 | GO:0006955 | immune response | il12a | interleukin 12a |
| ENSCLMG00005018959 | GO:0006955 | immune response | tnfsf12 | TNF superfamily member 12 |
| ENSCLMG00005019424 | GO:0002218 | activation of innate immune response | sting1 | stimulator of interferon response cGAMP interactor 1 |
| ENSCLMG00005019424 | GO:0045087 | innate immune response | sting1 | stimulator of interferon response cGAMP interactor 1 |
| ENSCLMG00005022205 | GO:0006955 | immune response |  | IL-6 interleukin-6-like* |
| ENSCLMG00005022205 | GO:0072540 | T-helper 17 cell lineage commitment |  |  |
| ENSCLMG00005022603 | GO:0006955 | immune response | TNFSF10 | TNF superfamily member 10 |

*= annotation based on blastp search

**GO term: Cytokine receptor binding (GO:0005126). Gene ratio 14/867. Adjusted p-value: 0.0372**

| **Gene stable ID** | **GO term accession** | **GO term name** | **Gene name** | **Gene description** |
| --- | --- | --- | --- | --- |
| ENSCLMG00005001818 | GO:0005164 | tumor necrosis factor receptor binding | TNFSF14 | tumor necrosis factor ligand superfamily member 14 |
| ENSCLMG00005001873 | GO:0008009 | chemokine activity | CXCL10 | C-X-C motif chemokine 10* |
| ENSCLMG00005008690 | GO:0008009 | chemokine activity | CCL35 | C-C motif chemokine 35* |
| ENSCLMG00005008778 | GO:0008009 | chemokine activity | CXC-CK3 | CC chemokine CK3* |
| ENSCLMG00005011065 | GO:0008009 | chemokine activity | CCL13 | C-C motif chemokine 13* |
| ENSCLMG00005011066 | GO:0008009 | chemokine activity | CCL19 | C-C motif chemokine 19* |
| ENSCLMG00005012320 | GO:0005126 | cytokine receptor binding | ifn3 | interferon phi 3* |
| ENSCLMG00005012324 | GO:0005126 | cytokine receptor binding | ifni | interferon phi 1* |
| ENSCLMG00005012324 | GO:0005132 | type I interferon receptor binding |  |  |
| ENSCLMG00005012330 | GO:0005164 | tumor necrosis factor receptor binding | TNF-β | tumor necrosis factor beta* |
| ENSCLMG00005015980 | GO:0008009 | chemokine activity | CXCL10 | C-X-C motif chemokine 10* |
| ENSCLMG00005016663 | GO:0005143 | interleukin-12 receptor binding | il12a | interleukin 12a |
| ENSCLMG00005018959 | GO:0005164 | tumor necrosis factor receptor binding | tnfsf12 | TNF superfamily member 12 |
| ENSCLMG00005022205 | GO:0005138 | interleukin-6 receptor binding | IL-6 | interleukin-6-like* |
| ENSCLMG00005022603 | GO:0005164 | tumor necrosis factor receptor binding | TNFSF10 | TNF superfamily member 10 |

*= annotation based on blastp search
