# Supplementary material for: Transcriptome-wide analyses of early immune responses in lumpfish leukocytes upon stimulation with poly(I:C)
Source: Front Immunol. 2023 Jun 14;14:1198211. doi: 10.3389/fimmu.2023.1198211 (PMC10300353; doi:10.3389/fimmu.2023.1198211)
Supplement: Supplementary file 5 [file Table_3.docx]

**Supplemental Table 3.** GO terms in the upset plot

**GO terms: Subset 66**

| **GO term accession** | **GO term name** | **p-value** |
| --- | --- | --- |
| GO:1901575 | tRNA metabolic process | 0.00269636 |
| GO:1901565 | tRNA aminoacylation for protein translation | 0.0001134 |
| GO:0072522 | tRNA aminoacylation | 0.00301487 |
| GO:0050776 | threonine-type peptidase activity | 0.00282064 |
| GO:0048872 | small molecule metabolic process | 0.00137265 |
| GO:0048534 | ribose phosphate metabolic process | 0.00015738 |
| GO:0046390 | ribose phosphate biosynthetic process | 0.00106081 |
| GO:0046034 | ribonucleotide metabolic process | 1.88748E-0 |
| GO:0045333 | ribonucleotide biosynthetic process | 0.00057879 |
| GO:0044281 | ribonucleoside triphosphate metabolic process | 0.00089341 |
| GO:0043648 | ribonucleoside triphosphate biosynthetic process | 0.00049525 |
| GO:0043039 | ribonucleoprotein complex | 5.35841E-06 |
| GO:0043038 | response to stress | 5.35841E-06 |
| GO:0042775 | response to external stimulus | 8.841E-05 |
| GO:0042773 | respiratory electron transport chain | 3.70922E-05 |
| GO:0034660 | respiratory chain complex | 0.00029936 |
| GO:0034101 | respirasome | 0.00027904 |
| GO:0030218 | regulation of immune response | 0.00023663 |
| GO:0030163 | purine-containing compound biosynthetic process | 0.00149709 |
| GO:0030099 | purine ribonucleotide metabolic process | 0.0027352 |
| GO:0030097 | purine ribonucleotide biosynthetic process | 0.0002250 |
| GO:0022904 | purine ribonucleoside triphosphate metabolic process | 9.65523E-05 |
| GO:0022900 | purine ribonucleoside triphosphate biosynthetic process | 1.26667E-05 |
| GO:0019693 | purine nucleotide biosynthetic process | 0.00273523 |
| GO:0019646 | purine nucleoside triphosphate metabolic process | 8.841E-05 |
| GO:0015980 | purine nucleoside triphosphate biosynthetic process | 0.002468483 |
| GO:0009605 | proton-transporting two-sector ATPase complex | 0.000621101 |
| GO:0009260 | proton transmembrane transporter activity | 0.001060814 |
| GO:0009259 | protein catabolic process | 0.00273523 |
| GO:0009206 | oxidoreduction-driven active transmembrane transporter activity | 8.841E-05 |
| GO:0009205 | oxidative phosphorylation | 8.841E-05 |
| GO:0009201 | organonitrogen compound catabolic process | 0.000169803 |
| GO:0009199 | organic substance catabolic process | 0.000169803 |
| GO:0009152 | nucleoside triphosphate metabolic process | 0.000583485 |
| GO:0009150 | nucleoside triphosphate biosynthetic process | 0.001820734 |
| GO:0009145 | ncRNA metabolic process | 8.841E-05 |
| GO:0009144 | myeloid cell homeostasis | 8.841E-05 |
| GO:0009142 | myeloid cell differentiation | 0.000229522 |
| GO:0009141 | mitochondrial respirasome | 0.000520936 |
| GO:0009060 | mitochondrial matrix | 0.000266456 |
| GO:0009056 | mitochondrial ATP synthesis coupled electron transport | 0.001146063 |
| GO:0006954 | ligase activity, forming carbon-oxygen bonds | 0.002415249 |
| GO:0006950 | ligase activity | 0.002855914 |
| GO:0006754 | inflammatory response | 4.28295E-06 |
| GO:0006520 | immune system development | 0.000935011 |
| GO:0006418 | homeostasis of number of cells | 3.88876E-06 |
| GO:0006399 | hemopoiesis | 1.68054E-05 |
| GO:0006164 | hematopoietic or lymphoid organ development | 0.00122146 |
| GO:0006119 | generation of precursor metabolites and energy | 7.12592E-05 |
| GO:0006091 | erythrocyte homeostasis | 0.000297765 |
| GO:0002520 | erythrocyte differentiation | 0.000170558 |
| GO:0002262 | energy derivation by oxidation of organic compounds | 0.000383804 |
| GO:1990904 | electron transport chain | 3.2143E-05 |
| GO:0098803 | dicarboxylic acid metabolic process | 0.00063333 |
| GO:0070469 | cellular respiration | 0.000178757 |
| GO:0016469 | cellular amino acid metabolic process | 0.002717579 |
| GO:0005759 | catalytic activity, acting on RNA | 0.00017379 |
| GO:0005746 | catalytic activity, acting on a tRNA | 9.37068E-05 |
| GO:0140101 | catabolic process | 1.44981E-05 |
| GO:0140098 | ATP synthesis coupled electron transport | 6.75738E-06 |
| GO:0070003 | ATP metabolic process | 3.16045E-05 |
| GO:0016875 | ATP biosynthetic process | 6.47816E-06 |
| GO:0016874 | aminoacyl-tRNA ligase activity | 1.49591E-07 |
| GO:0015453 | amino acid activation | 0.000344359 |
| GO:0015078 | aerobic respiration | 8.79309E-05 |
| GO:0004812 | aerobic electron transport chain | 6.47816E-06 |

**GO terms: Subset 9**

| **GO term accession** | **GO term name** | **p-value** |
| --- | --- | --- |
| GO:1903131 | regulation of programmed cell death | 0.00091785 |
| GO:0046649 | regulation of cell death | 0.00133773 |
| GO:0043067 | regulation of apoptotic process | 0.00092660 |
| GO:0042981 | programmed cell death | 0.00082537 |
| GO:0030098 | mononuclear cell differentiation | 0.00060054 |
| GO:0012501 | lymphocyte differentiation | 2.70656E-05 |
| GO:0010941 | lymphocyte activation | 0.00129312 |
| GO:0008219 | cell death | 4.62186E-05 |
| GO:0006915 | apoptotic process | 2.24694E-05 |

**GO terms: Subset 1**

| **GO term accession** | **GO term name** | **p-value** |
| --- | --- | --- |
| GO:0005615 | extracellular space | 0.00270 |

**GO terms: Subset 32**

| **GO term accession** | **GO term name** | **p-value** | |
| --- | --- | --- | --- |
|  |  | 24Hpe | 24Hpe_up |
| GO:0043604 | translation | 3.95081E-21 | 4.64602E-37 |
| GO:0043603 | structural molecule activity | 1.92111E-20 | 1.99363E-38 |
| GO:0043043 | structural constituent of ribosome | 1.51635E-22 | 6.2375E-39 |
| GO:0034645 | small ribosomal subunit | 6.94509E-13 | 1.27933E-23 |
| GO:0031347 | RNA binding | 8.85942E-05 | 8.04608E-06 |
| GO:0015986 | ribosome | 0.000474585 | 2.41947E-06 |
| GO:0006518 | ribosomal subunit | 5.52432E-22 | 2.69466E-39 |
| GO:0006412 | regulation of defense response | 2.12308E-22 | 1.55959E-38 |
| GO:1905369 | proton-transporting two-sector ATPase complex, catalytic domain | 7.71672E-09 | 4.71876E-10 |
| GO:1905368 | proton-transporting ATP synthase complex | 2.64313E-07 | 4.12596E-09 |
| GO:0140535 | proton motive force-driven ATP synthesis | 0.000405746 | 0.000293468 |
| GO:0098800 | proteasome core complex, alpha-subunit complex | 0.003723009 | 6.70711E-06 |
| GO:0098798 | proteasome core complex | 0.001127829 | 4.37435E-06 |
| GO:0045259 | proteasome complex | 0.00080886 | 1.61603E-05 |
| GO:0044391 | peptide metabolic process | 1.03087E-06 | 2.65634E-10 |
| GO:0033178 | peptide biosynthetic process | 0.003177696 | 0.0012681 |
| GO:0031975 | peptidase complex | 0.00125906 | 1.69446E-05 |
| GO:0031967 | organelle inner membrane | 0.00125906 | 1.69446E-05 |
| GO:0031966 | organelle envelope | 0.001382473 | 1.05633E-05 |
| GO:0019866 | mitochondrion | 0.003316621 | 0.000162089 |
| GO:0019773 | mitochondrial protein-containing complex | 0.001930098 | 0.001440084 |
| GO:0015935 | mitochondrial membrane | 0.000257755 | 4.54871E-06 |
| GO:0015934 | mitochondrial inner membrane | 0.001060297 | 1.55896E-05 |
| GO:0005840 | mitochondrial envelope | 2.92458E-24 | 1.73647E-38 |
| GO:0005839 | large ribosomal subunit | 4.56586E-09 | 4.98059E-11 |
| GO:0005743 | intracellular protein-containing complex | 0.002641172 | 5.46668E-05 |
| GO:0005740 | inner mitochondrial membrane protein complex | 0.000471321 | 6.9448E-06 |
| GO:0005739 | envelope | 2.43413E-06 | 3.37869E-09 |
| GO:0000502 | endopeptidase complex | 1.71708E-09 | 2.02542E-11 |
| GO:0003735 | cellular macromolecule biosynthetic process | 3.93561E-24 | 1.29741E-42 |
| GO:0003723 | cellular amide metabolic process | 0.00018113 | 0.00018113 |
| GO:0005198 | amide biosynthetic process | 5.86366E-12 | 1.27639E-29 |

**GO terms: Subset 4**

| **GO term accession** | **GO term name** | **p-value** | |
| --- | --- | --- | --- |
|  |  | **24Hpe** | **TI_UP** |
| GO:0098542 | defense response to other organism | 2.90951E-05 | 5.44557E-05 |
| GO:0019882 | antigen processing and presentation | 6.90775E-05 | 7.33187E-05 |
| GO:0009615 | response to virus | 0.000509531 | 0.000917853 |
| GO:0001664 | G protein-coupled receptor binding | 0.001219868 | 0.000473618 |

**GO terms: Subset 2**

| **GO term accession** | **GO term name** | **p-value** | |
| --- | --- | --- | --- |
|  |  | **6Hpe_UP** | **TI_UP** |
| GO:0005576 | extracellular region | 2.94E-05 | 7.30E-07 |
| GO:0005102 | signaling receptor binding | 0.000616093 | 7.41E-06 |

**GO terms: Subset 7**

| **GO term accession** | **GO term name** | **p-value** | | |
| --- | --- | --- | --- | --- |
|  |  | **24Hpe** | **24Hpe_up** | **TI_up** |
| GO:0006952 | response to other organism | 1.13062E-05 | 4.93096E-09 | 2.82681E-06 |
| GO:0051707 | response to external biotic stimulus | 5.00266E-06 | 2.80688E-08 | 2.82681E-06 |
| GO:0044419 | response to biotic stimulus | 7.20661E-06 | 1.00074E-07 | 5.71282E-06 |
| GO:0043207 | regulation of response to biotic stimulus | 5.00266E-06 | 2.80688E-08 | 2.82681E-06 |
| GO:0009607 | regulation of immune system process | 6.35722E-06 | 3.49729E-08 | 3.13721E-06 |
| GO:0002831 | defense response | 0.000305944 | 3.40491E-05 | 0.000210869 |
| GO:0002682 | biological process involved in interspecies interaction between organisms | 0.000106657 | 0.000598137 | 0.000236049 |

**GO terms: Subset 6**

| **GO term accession** | **GO term name** | **p-value** | | |
| --- | --- | --- | --- | --- |
|  |  | **6Hpe_up** | **24Hpe_up** | **TI_up** |
| GO:0048018 | signaling receptor regulator activity | 9.98879E-05 | 0.001568693 | 2.05741E-06 |
| GO:0042379 | signaling receptor activator activity | 0.003823667 | 6.61636E-06 | 1.16874E-05 |
| GO:0030546 | receptor ligand activity | 9.98879E-05 | 0.001568693 | 2.05741E-06 |
| GO:0030545 | NAD+ ADP-ribosyltransferase activity | 0.000104673 | 0.001752001 | 2.30371E-06 |
| GO:0008009 | chemokine receptor binding | 0.003823667 | 6.61636E-06 | 1.16874E-05 |
| GO:0003950 | chemokine activity | 0.00276138 | 0.000223301 | 5.59174E-05 |

**GO terms: Subset 2**

| **GO term accession** | **GO term name** | **p-value** | | | |
| --- | --- | --- | --- | --- | --- |
|  |  | **24Hpe** | **24Hpe_up** | **TI_up** | **TI** |
| GO:0006955 | immune response | 3.39349E-07 | 1.96176E-09 | 2.79849E-09 | 2.61397E-05 |
| GO:0002376 | immune system process | 5.19091E-09 | 2.71594E-11 | 7.41041E-09 | 7.73733E-05 |

**GO terms: Subset 1**

| **GO term accession** | **GO term name** | **p-value** | | | |
| --- | --- | --- | --- | --- | --- |
|  |  | **6Hpe_up** | **24Hpe** | **24Hpe_up** | **TI_up** |
| GO:0005125 | cytokine activity | 1.53E-05 | 4.10E-05 | 7.28553E-09 | 9.39E-09 |

**GO terms: Subset 1**

| **GO term accession** | **GO term name** | **p-value** | | | | |
| --- | --- | --- | --- | --- | --- | --- |
|  |  | **6Hpe_up** | **24Hpe** | **24Hpe_up** | **TI** | **TI_up** |
| GO:0005126 | cytokine receptor binding | 4.28E-06 | 0.000127667 | 3.27777E-08 | 9.72E-05 | 8.07E-09 |
