# Supplementary material for: Transcriptome-wide analyses of early immune responses in lumpfish leukocytes upon stimulation with poly(I:C)
Source: Front Immunol. 2023 Jun 14;14:1198211. doi: 10.3389/fimmu.2023.1198211 (PMC10300353; doi:10.3389/fimmu.2023.1198211)
Supplement: Supplementary file 6 [file Table_4.docx]

| **Supplemental Table 4.** Abbreviations used in Fig. 4D and Fig. 7B, C. | | | |
| --- | --- | --- | --- |
| **Ensemble Number** | **Gene** | **Details** |  |
| ENSCLMG00005013429 | MARVELD | si:ch211-191a24.4 (Predicted: MAL and related proteins for vesicle trafficking and membrane link- domain containing 3) |  |
| ENSCLMG00005002149 | CX30.9 | connexin30.9 |  |
| ENSCLMG00005015292 | ALDH (1) | Predicted: aldehyde dehydrogenase 1 family, member |  |
| ENSCLMG00005018435 | TENM | Predicted: teneurin transmembrane protein (4) |  |
| ENSCLMG00005012378 | CAX1 | Predicted: cation/H+ exchanger protein 1 |  |
| ENSCLMG00005019686 | RSAD2 | radicalS-adenosylmethioninedomaincontaining2 |  |
| ENSCLMG00005017999 | PAPLNA | papilin a, proteoglycan-like sulfated glycoprotein |  |
| ENSCLMG00005020000 | LPAR2A | lysophosphatidicacidreceptor2a |  |
| ENSCLMG00005011248 | PARP11 | poly(ADP-ribose) polymerase family member 11 |  |
| ENSCLMG00005003939 | MX1 | MXdynaminlikeGTPase1 |  |
| ENSCLMG00005010666 | CPNE2 | copineII |  |
| ENSCLMG00005019424 | STING1 | stimulatorofinterferonresponsecGAMPinteractor1 |  |
| ENSCLMG00005015335 | LGP2/DHX58 | DEXH (Asp-Glu-X-His) box polypeptide 58 |  |
| ENSCLMG00005018843 | ACOD1 | aconitate decarboxylase 1 |  |
| ENSCLMG00005002258 | IRF3 | interferonregulatoryfactor3 |  |
| ENSCLMG00005011806 | MOV10 | Mov10RISCcomplexRNAhelicase |  |
| ENSCLMG00005022332 | USP18 | ubiquitinspecificpeptidase18 |  |
| ENSCLMG00005017857 | OCLNA | occludina |  |
| ENSCLMG00005004398 | STEAP4 | STEAPfamilymember4 |  |
| ENSCLMG00005020206 | UBA7 | ubiquitin-likemodifieractivatingenzyme7 |  |
| ENSCLMG00005007273 | IGDCC3 | Immunoglobulin superfamily, DCC subclass,member3 |  |
| ENSCLMG00005017332 | IRF10 | interferonregulatoryfactor10 |  |
| ENSCLMG00005005791 | SOCS1A | suppressorofcytokinesignaling1a |  |
| ENSCLMG00005000344 | IFI35 | interferon-inducedprotein35 |  |
| ENSCLMG00005015340 | IRF1B | interferonregulatoryfactor1b |  |
| ENSCLMG00005010855 | NLRC5 | NLRfamilyCARDdomaincontaining5 |  |
| ENSCLMG00005015653 | SOX9B | SRY-boxtranscriptionfactor9b |  |
| ENSCLMG00005002557 | SOCS1B | suppressorofcytokinesignaling1b |  |
| ENSCLMG00005003533 | STAT1A | signaltransducerandactivatoroftranscription1a |  |
| ENSCLMG00005003426 | MDA5 | interferon induced with helicase C domain 1 |  |
| ENSCLMG00005010239 | STAT2 | signaltransducerandactivatoroftranscription2 |  |
| ENSCLMG00005022952 | IRF7 | interferonregulatoryfactor7 |  |
| ENSCLMG00005015486 | TLR7 | toll-likereceptor7 |  |
| ENSCLMG00005001707 | ADAR | Adenosine deaminase RNA specific |  |
| ENSCLMG00005004082 | TRIM25B | tripartite motif containing 25 |  |
| ENSCLMG00005004829 | IL12B | interleukin12B |  |
| ENSCLMG00005001276 | HSP70.1 | heatshockcognate70-kdprotein, tandemduplicate1 |  |
| ENSCLMG00005011099 | NDUFB6 | NADH: ubiquinone oxidoreductase subunit B |  |
| ENSCLMG00005000569 | UBA52 | ubiquitinA-52residueribosomalproteinfusionproduct1 |  |
| ENSCLMG00005009317 | RACK1 | receptorforactivatedCkinase1 |  |
| ENSCLMG00005015196 | STAT5A | signaltransducerandactivatoroftranscription5a |  |
| ENSCLMG00005009987 | STAT4 | signaltransducerandactivatoroftranscription4 |  |
| ENSCLMG00005004085 | TRIM25A | tripartite motif containing 25 |  |
| ENSCLMG00005000677 | TRAF6 | TNFreceptor-associatedfactor6 |  |
| ENSCLMG00005017466 | NFKBIL1 | nuclearfactorofkappalightpolypeptidegeneenhancerinB-cellsinhibitor-like1 |  |
| ENSCLMG00005009817 | IL10 | interleukin10 |  |
| ENSCLMG00005015156 | STAT3 | signaltransducerandactivatoroftranscription3(acute-phaseresponsefactor) |  |
| ENSCLMG00005015930 | TNIK | TRAF2andNCKinteractingkinase |  |
| ENSCLMG00005021957 | STAT6 | signaltransducerandactivatoroftranscription6, interleukin-4induced |  |
| ENSCLMG00005000407 | RNF126 | ringfingerprotein126 |  |
| ENSCLMG00005005110 | UBR7 | ubiquitinproteinligaseE3componentn-recognin7 |  |
| ENSCLMG00005017854 | MAP3K7 | mitogen-activatedproteinkinasekinasekinase7 |  |
| ENSCLMG00005014176 | TLR21 | toll-likereceptor21 |  |
| ENSCLMG00005005137 | TRIM8 | tripartitemotifcontaining8 |  |
| ENSCLMG00005005105 | PIK3R3 | phosphoinositide-3-kinase, regulatorysubunit3b(gamma) |  |
| ENSCLMG00005015654 | TRIM45 | tripartitemotifcontaining45 |  |
| ENSCLMG00005014826 | AKT2 | v-aktmurinethymomaviraloncogenehomolog2 |  |
| ENSCLMG00005020302 | CASP10 | caspase10, apoptosis-related cysteine epeptidase |  |
| ENSCLMG00005016663 | IL12A | interleukin12a |  |
| ENSCLMG00005003299 | RELA | v-rel reticuloendotheliosis viral oncogene homolog A |  |
| ENSCLMG00005002340 | CHUK | Component of inhibitor of nuclear factor kappa B kinase complex |  |
| ENSCLMG00005012066 | IKBKB | Inhibitor of nuclear factor kappa B kinase sub unit beta |  |
| ENSCLMG00005008841 | PIK3CB | phosphatidylinositol-4,5-bisphosphate3-kinase, catalytic subunit beta |  |
| ENSCLMG00005001703 | MAP2K6 | mitogen-activatedproteinkinasekinase6 |  |
| ENSCLMG00005015694 | MAPK1 | mitogen-activatedproteinkinase1 |  |
| ENSCLMG00005007263 | CTSK | Cathepsin K |  |
| ENSCLMG00005001158 | FADD | Fas(tnfrsf6)-associated via death domain |  |
| ENSCLMG00005008748 | JUN | Junproto-oncogene, AP-1transcriptionfactorsubunit |  |
| ENSCLMG00005005860 | MAPK3 | mitogen-activatedproteinkinase3 |  |
| ENSCLMG00005015839 | MAP2K7 | mitogen-activatedproteinkinasekinase7 |  |
| ENSCLMG00005004900 | PIK3CA | phosphatidylinositol-4,5-bisphosphate3-kinase, catalytic subunit alpha |  |
| ENSCLMG00005006159 | AZI2 | 5-azacytidineinduced2 |  |
