# Supplementary material for: Transcriptome-wide analyses of early immune responses in lumpfish leukocytes upon stimulation with poly(I:C)
Source: Front Immunol. 2023 Jun 14;14:1198211. doi: 10.3389/fimmu.2023.1198211 (PMC10300353; doi:10.3389/fimmu.2023.1198211)
Supplement: Supplementary file 7 [file Table_5.docx]

**Supplemental Table 5.** Accession no. used in the phylogenetic analysis.

| **RIG-I (DDX58)** |  |
| --- | --- |
| XP 007904799.1 | Callorhinchus milii |
| XP 006009599.1 | Latimeria chalumnae |
| XP 015201265.1 | Lepisosteus oculatus |
| XP 018594052.1 | Scleropages formosus |
| NP 001157171.1 | Salmo salar |
| NP 001265735.1 | Ictalurus punctatus |
| XP 017544078.1 | Pygocentrus nattereri |
| NP 001293024.1 | Danio rerio |
| AGN48011.1 | Danio rerio |
| AGN48008.1 | Danio rerio |
| CAY86112.1 | Cyprinidae sp. |
| AGH30714.1 | Ctenopharyngodon idella |
| ADC81089.2 | Ctenopharyngodon idella |
| AEN04472.1 | Carassius auratus |
| ADZ55452.1 | Cyprinus carpio |
| XP 018952401.1 | Cyprinus carpio |
| XP 016359901.1 | Sinocyclocheilus anshuiensis |
| XP 016405112.1 | Sinocyclocheilus rhinocerous |
|  |  |
| **LGP2 (DHX58)** |  |
| XP 019752532.1 | Hippocampus comes |
| XP 003964848.1 | Takifugu rubripes |
| XP 008327470.1 | Cynoglossus semilaevis |
| ADM18136.1 | Paralichthys olivaceus |
| ADI75503.1 | Paralichthys olivaceus |
| XP 019949839.1 | Paralichthys olivaceus |
| XP 018558212.1 | Lates calcarifer |
| AOV82294.1 | Lates calcarifer |
| XP 015809356.1 | Nothobranchius furzeri |
| XP 013878529.1 | Cyprinodontiformes |
| XP 017284942.1 | Kryptolebias marmoratus |
| XP 015253302.1 | Cyprinodon variegatus |
| XP 005796753.1 | Xiphophorus maculatus |
| XP 008415285.1 | Poecilia reticulata |
| XP 007558174.1 | Poecilia formosa |
| XP 014893336.1 | Poecilia latipinna |
| XP 014848824.1 | Poecilia mexicana |
| XP 012727447.1 | Fundulus heteroclitus |
| XP 004071339.1 | Oryzias latipes |
| XP 006798306.1 | Neolamprologus brichardi |
| XP 019213273.1 | Oreochromis niloticus |
| XP 005920418.1 | Haplochromis burtoni |
| XP 014263552.1 | Maylandia zebra |
| XP 019213271.1 | Oreochromis niloticus |
| XP 006798307.1 | Neolamprologus brichardi |
| XP 005726146.1 | Pundamilia nyererei |
| XP 005920417.1 | Stegastes partitus |
| XP 008283077.1 | Stegastes partitus |
| XP 020463090.1 | Monopterus albus |
| XP 010784105.1 | Notothenia coriiceps |
| XP 020488255.1 | Labrus bergylta |
| ENSCLMP00005032208.1 | Cyclopterus lumpus |
| ALE66118.1 | Lateolabrax japonicus |
| APU51302.1 | Miichthys miiuy |
| KKF29873.1 | Larimichthys crocea |
| AHX37213.1 | Oplegnathus fasciatus |
| XP 010873108.1 | Esox lucius |
| XP 020342088.1 | Oncorhynchus kisutch |
| XP 014059582.1 | Salmo salar |
| NP 001133649.1 | Salmo salar |
| XP 021480505.1 | Oncorhynchus mykiss |
| XP 012672250.1 | Clupea harengus |
| AKA09351.1 | Danio rerio |
| ANY28498.1 | Mylopharyngodon piceus |
| AFQ93565.1 | Ctenopharyngodon idella |
| AEN04474.1 | Carassius auratus |
| AIX47137.1 | Cyprinus carpio |
| XP 016395114.1 | Sinocyclocheilus rhinocerous |
| XP 016339635.1 | Sinocyclocheilus anshuiensis |
| KTG38443.1 | Cyprinus carpio |
| XP 016344075.1 | Sinocyclocheilus anshuiensis |
| XP 016427927.1 | Sinocyclocheilus rhinocerous |
| XP 017576716.1 | Pygocentrus nattereri |
| NP 001265738.1 | Ictalurus punctatus |
| XP 018619809.1 | Scleropages formosus |
|  |  |
| **MDA5** |  |
| XP 030199004.1 | Gadus morhua |
| XP 020784592.1 | Boleophthalmus pectinirostris |
| XP 019723743.1 | Hippocampus comes |
| XP 004081944.1 | Oryzias latipes |
| XP 015822058.1 | Nothobranchius furzeri |
| XP 017279885.1 | Kryptolebias marmoratus |
| XP 013856958.1 | Austrofundulus limnaeus |
| XP 014326614.1 | Xiphophorus maculatus |
| XP 008402244.1 | Poecilia reticulata |
| XP 014831621.1 | Poecilia mexicana |
| XP 007546654.1 | Poecilia formosa |
| XP 014905849.1 | Poecilia latipinna |
| XP 012729491.1 | Fundulus heteroclitus |
| XP 015225886.1 | Cyprinodon variegatus |
| XP 008286628.1 | Stegastes partitus |
| AIP84311.1 | Etroplus suratensis |
| XP 019208061.1 | Oreochromis niloticus |
| XP 006788592.1 | Neolamprologus brichardi |
| XP 005723472.1 | Pundamilia nyererei |
| XP 005918274.1 | Haplochromis burtoni |
| XP 014264522.1 | Maylandia zebra |
| AUN88445.1 | Oreochromis niloticus |
| XP 008326929.1 | Cynoglossus semilaevis |
| ADU87114.1 | Paralichthys olivaceus |
| XP 018557904.1 | Lates calcarifer |
| AOV82292.1 | Lates calcarifer |
| XP 042282966.1 | Thunnus maccoyii |
| XP 044222292.1 | Thunnus albacares |
| XP 020450399.1 | Monopterus albus |
| AKI07286.1 | Siniperca chuatsi |
| AHX37214.1 | Oplegnathus fasciatus |
| ANQ31758.1 | Larimichthys crocea |
| XP 051259083.1 | Dicentrarchus labrax |
| XP 035532757.1 | Morone saxatilis |
| AMW90927.1 | Lateolabrax japonicus |
| XP 011608567.1 | Takifugu rubripes |
| CAG09339.1 | Tetraodon nigroviridis |
| XP 020499807.1 | Labrus bergylta |
| ENSCLMP00005006804.1 | Cyclopterus lumpus |
| XP 010780897.1 | Notothenia coriiceps |
| AEX01716.1 | Epinephelus coioides |
| XP 010889175.2 | Esox lucius |
| XP 014019608.1 | Salmo salar |
| XP 014019608.1 | Salmo salar |
| XP 029559154.1 | Salmo trutta |
| XP 023836851.1 | Salvelinus alpinus |
| NP 001182108.1 | Oncorhynchus mykiss |
| XP 020316852.1 | Oncorhynchus kisutch |
| XP 024244677.1 | Oncorhynchus tshawytscha |
| XP 029540612.1 | Oncorhynchus nerka |
| XP 031414183.1 | Clupea harengus |
| NP 001295492.1 | Danio rerio |
| ACT68336.2 | Carassius auratus |
| AEN04473.1 | Carassius auratus |
| XP 051761287.1 | Ctenopharyngodon idella |
| ARO77472.1 | Mylopharyngodon piceus |
| APB09201.1 | Squaliobarbus curriculus |
| XP 050975681.1 | Labeo rohita |
| XP 026126943.1 | Carassius auratus |
| AIX47136.1 | Cyprinus carpio |
| XP 016396932.1 | Sinocyclocheilus rhinocerous |
| XP 016101519.1 | Sinocyclocheilus grahami |
| XP 016104280.1 | Sinocyclocheilus grahami |
| XP 016386882.1 | Sinocyclocheilus rhinocerous |
| XP 016324338.1 | Sinocyclocheilus anshuiensis |
| NP 001265741.1 | Ictalurus punctatus |
| XP 017547738.1 | Pygocentrus nattereri |
| XP 007230472.3 | Astyanax mexicanus |
| KPP78498.1 | Scleropages formosus |
| XP 018597147.1 | Scleropages formosus |
| XP 006636679.1 | Lepisosteus oculatus |
